# Microbial ecological instability and non-linear host interactions shape airway inflammation in HIV-associated COPD

**DOI:** 10.1038/s41467-026-78400-4

**Published:** 2026-10-06

**Authors:** Alex Kayongo, Theda Ulrike Patricia Bartolomaeus, Julia Hecker, Till Birkner, Eremire Vila, Moses Levi Ntayi, Lajos Markó, Ulrike Löber, Geoffrey Olweny, Edward Kyalo, Daisy Kyamulabi, Josephine Ndawula, Willy Ssengooba, Rose Nabatanzi, Samuel Kyobe, Faith Nassali, Edgar Kigozi, Robert Kalyesubula, Richard Munana, Jesca Namaganda, Caroline J. Musiime, Rogers Sekibira, Bernard S. Bagaya, David P. Kateete, Moses L. Joloba, Daudi Jjingo, John Lusiba, William Worodria, Harriet Mayanja-Kizza, Rejani Lalitha, Wasswa Ham, Ingvar Sanyu, Josephine Zawedde, Nicole M. Robertson, James Sande Obondo, Carl Weidinger, Desiree Kunkel, Bruce J. Kirenga, Trishul Siddharthan, John R. Hurst, William Checkley, William B. Cookson, Miriam F. Moffatt, Sofia K. Forslund-Startceva

**Affiliations:** 1 Department of Immunology and Molecular Biology, Makerere University College of Health Sciences, Kampala, Uganda; 2 Makerere University Lung Institute, Makerere University College of Health Sciences, Kampala, Uganda; 3 Berlin Institute of Health, Charité-Universitatsmedizin Berlin, corporate member of Freie Universität Berlin, Humboldt-Universität zu Berlin, Berlin, Germany; 4 Experimental and Clinical Research Center, a cooperation of Charité-Universitatsmedizin Berlin and Max Delbrück Center for Molecular Medicine, Berlin, Germany; 5 Max Delbrück Center for Molecular Medicine in the Helmholtz Association, Berlin, Germany; 6 German Centre for Cardiovascular Research, Partner Site, Berlin, Berlin, Germany; 7 Department of Gastroenterology, Infectious Diseases and Rheumatology, Campus Benjamin Franklin, Charité - Universitätsmedizin Berlin, corporate member of Freie Universität Berlin and Humboldt-Universität zu Berlin, Berlin, Germany; 8 Department of Dermatology, Medical University of Vienna, Vienna, Austria; 9 CeMM Research Center for Molecular Medicine, Austrian Academy of Sciences, Vienna, Austria; 10 Diagnostic and Research Institute of Hygiene, Microbiology and Environmental Medicine, Medical University of Graz, Graz, Austria; 11 Department of Medical Microbiology, Makerere University College of Health Sciences, Kampala, Uganda; 12 Department of Research, African Community Center for Social Sustainability (ACCESS), Nakaseke, Uganda; 13 Department of Physiology, Makerere University College of Health Sciences, Kampala, Uganda; 14 Department of Computer Science, College of Computing and Information Sciences, Makerere University, Kampala, Uganda; 15 African Center of Excellence in Bioinformatics and Data Science, Infectious Diseases Institute, Kampala, Uganda; 16 Department of Medicine, Makerere University College of Health Sciences, Kampala, Uganda; 17 Infectious Diseases Research Collaboration, IDRC, Kampala, Uganda; 18 Department of Anaesthesia, Makerere University College of Health Sciences, Kampala, Uganda; 19 Division of Pulmonary and Critical Care Medicine, School of Medicine, Johns Hopkins University, Baltimore, MD USA; 20 Flow & Mass Cytometry Core Facility, Berlin Institute of Health at Charité - Universitätsmedizin Berlin, Berlin, Germany; 21 Division of Pulmonary and Critical Care Medicine, School of Medicine, University of Miami, Miami, FL USA; 22 UCL Respiratory, University College London, London, UK; 23 Center for Non-Communicable Disease Research and Training, School of Medicine, Johns Hopkins University, Baltimore, MD USA; 24 Respiratory Genetics, National Heart and Lung Institute, Imperial College London, London, UK; 25 Structural and Computational Biology Unit, European Molecular Biology Laboratory, Heidelberg, Germany; 26 Cluster of Excellence ImmunoPreCept (EXC 3118), Charité - Universitätsmedizin Berlin, Berlin, Germany

**Keywords:** Classification and taxonomy, Molecular medicine

## Abstract

Chronic obstructive pulmonary disease (COPD) is increasingly recognized among people living with HIV in sub-Saharan Africa, yet the mechanisms linking airway microbial ecology to chronic inflammation remain poorly understood. Here, we study 200 adults stratified by HIV and COPD status, including 160 participants with two-year longitudinal follow-up, using an integrated multi-omics framework combining sputum 16S rRNA profiling, bacterial outer membrane vesicle quantification, airway cytokine profiling, CyTOF immune phenotyping, bulk transcriptomics of induced sputum, bronchoalveolar lavage and peripheral blood, and bronchoalveolar lavage single-cell RNA sequencing-guided deconvolution. We find that sputum microbial communities are shaped predominantly by temporal ecological turnover rather than HIV or COPD status. Although microbial diversity, microbiome instability, outer membrane vesicle burden and inferred airway cellular composition remain largely preserved, HIV-associated COPD exhibits disease-specific microbial signatures together with inflammatory transcriptional remodeling concentrated in induced sputum. Integrated analyses identify selective host-microbe interaction networks linking discrete microbial taxa with inflammatory, immune and transcriptional programmes.

## Abstract (web summary)

Here, the authors show that unstable airway microbial communities and their interactions with the immune system are linked to airway inflammation in people with HIV and COPD.

## Introduction

Chronic obstructive pulmonary disease (COPD) is a major global health burden, with more than 80% of cases arising in resource-limited settings^1,2^. Unlike the smoking-driven phenotype predominant in high-income countries, COPD in low- and middle-income countries (LMICs) frequently develops in the setting of biomass exposure, chronic respiratory infections, and other non-smoking environmental insults that contribute to persistent airway inflammation and structural lung injury^1,3^. In sub-Saharan Africa, these exposures frequently coexist with HIV infection, prior tuberculosis, and recurrent respiratory infections, creating a biologically distinct form of chronic airway disease that remains incompletely characterized^4–8^. Understanding the host and microbial factors associated with this non-smoking COPD phenotype is therefore an important global health priority^9^.

The interaction between HIV infection, airway immunity, and the airway microbiome remains incompletely understood. HIV profoundly alters mucosal immune homeostasis^10^ and has been associated with changes in airway microbial community composition^11–13^. However, whether these microbial alterations simply accompany HIV-associated immune dysfunction or are consistently associated with distinct inflammatory programs remains unresolved. HIV infection is also associated with increased susceptibility to pneumonia, colonization by opportunistic pathogens including *Pneumocystis jirovecii*, and accelerated decline in lung function^12,14,15^. Collectively, these observations suggest that HIV-associated COPD may reflect complex interactions between mucosal immunity and airway microbial ecology rather than disruption of either system alone. Experimental studies have shown that individual airway bacteria and microbial products can activate innate and adaptive immune pathways in vitro^16–18^. These experimental observations provide biological plausibility for host–microbe interactions but do not establish that analogous mechanisms operate in human HIV-associated COPD, although evidence is now emerging as is the case for interstitial pulmonary fibrosis^19^. Likewise, while bacterial outer membrane vesicles (OMVs) contain immunologically active molecules capable of stimulating inflammatory pathways experimentally, their relationship with airway immune states in humans remains largely unknown^20^. A major knowledge gap therefore concerns how microbial community composition, microbial-derived products, and mucosal immune responses are organized within HIV-associated COPD in humans. Existing studies are predominantly cross-sectional, involve relatively small cohorts, lack integrated longitudinal sampling, or examine only individual components of the airway ecosystem in isolation. Few studies have simultaneously evaluated airway ecology, inflammatory mediators, immune-cell composition, and host transcriptional programs, particularly in African populations where HIV-associated non-smoking COPD is common. Consequently, it remains unclear whether distinct microbial states consistently align with specific immune and transcriptional programs or whether HIV-associated immune remodeling predominates independent of microbial community structure.

In this work, we investigate relationships between airway microbial ecology and host inflammatory responses in adults with and without HIV and COPD in rural Uganda using a longitudinal multi-omics framework. We integrate sputum 16S rRNA microbiome profiling, bacterial outer membrane vesicle quantification, cytokine measurements, immune phenotyping by CyTOF, and transcriptomic analyses of blood, induced sputum, and bronchoalveolar lavage (BAL). We find that longitudinal sputum microbial communities are shaped predominantly by temporal ecological turnover rather than HIV or COPD status, while disease-associated differences involve selected bacterial taxa and host immune-transcriptional programmes. HIV-associated COPD is associated with transcriptional remodeling in induced sputum despite largely preserved microbial diversity and inferred airway cellular composition. Integrated analyses identify associations between selected microbial taxa and inflammatory, immune-cell and transcriptional programmes. Together, these findings indicate selective host–microbe relationships rather than broad disruption of the airway microbial community.

## Results

### Longitudinal trajectories of lung function according to HIV and COPD status

**Fig. 1:**
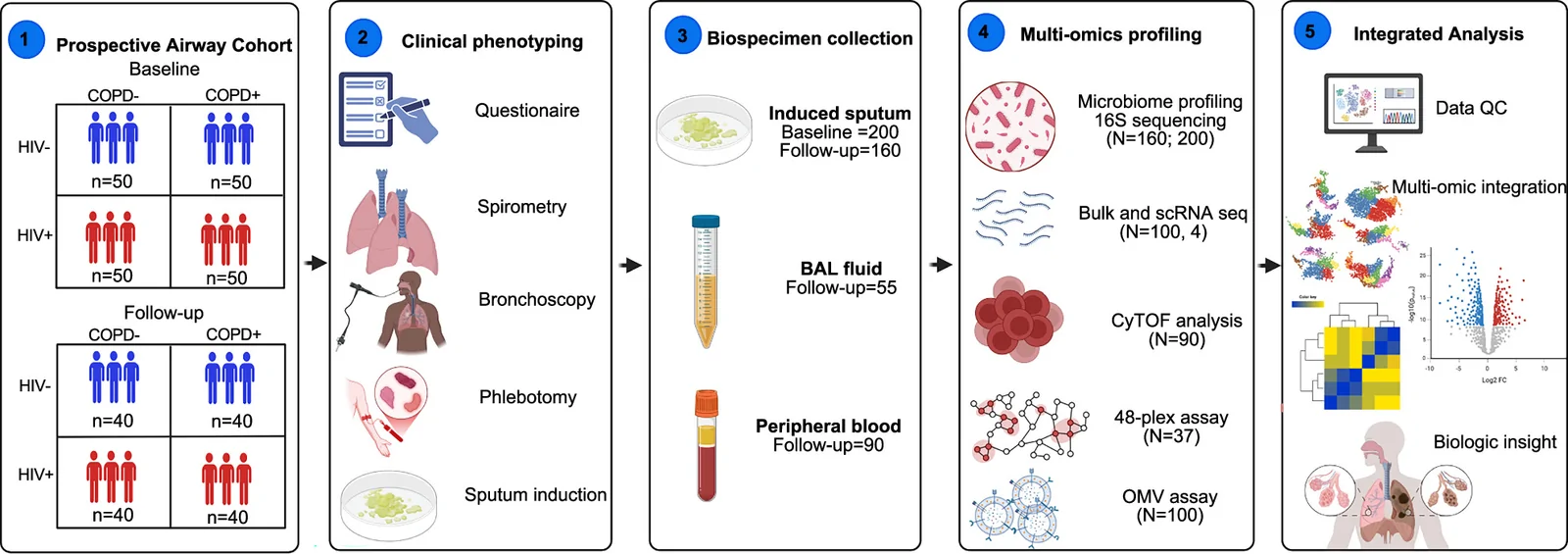
Study design and multi-omic profiling workflow. Participants were recruited from two prospective airway cohorts in the same geographic region and stratified into four clinical groups according to HIV and COPD status (HIV-/COPD-, HIV+/COPD-, HIV-/COPD+, and HIV+/COPD+; *n* = 50 participants per group; total *n* = 200 participants). Baseline assessments (from February 2018 to February 2021) included standardized clinical phenotyping (questionnaire, spirometry, sputum induction, bronchoscopy where applicable, and peripheral blood collection). Induced sputum underwent 16S rRNA gene sequencing targeting the V3-V4 hypervariable regions (*n* = 200 participants). Longitudinal follow-up (from April 2022 to January 2023) was completed for *n* = 160 participants, who underwent repeat spirometry and sputum microbiome profiling. Multi-omic analyses included sputum bulk RNA sequencing (*n* = 100), bronchoalveolar lavage (BAL) bulk RNA sequencing (*n* = 55), BAL single-cell RNA sequencing (*n* = 4), peripheral blood CyTOF immunophenotyping (*n* = 90), sputum cytokine profiling using a 48-plex Luminex assay (*n* = 37), and bacterial outer membrane vesicle (OMV) isolation with lipopolysaccharide (LPS) quantification (*n* = 100). Data from all platforms were integrated to investigate microbial, immunological, and transcriptional signatures associated with HIV, COPD, and their coexistence. Created in BioRender. Kayongo, A. (2026) https://BioRender.com/s66563t.

**Fig. 2:**
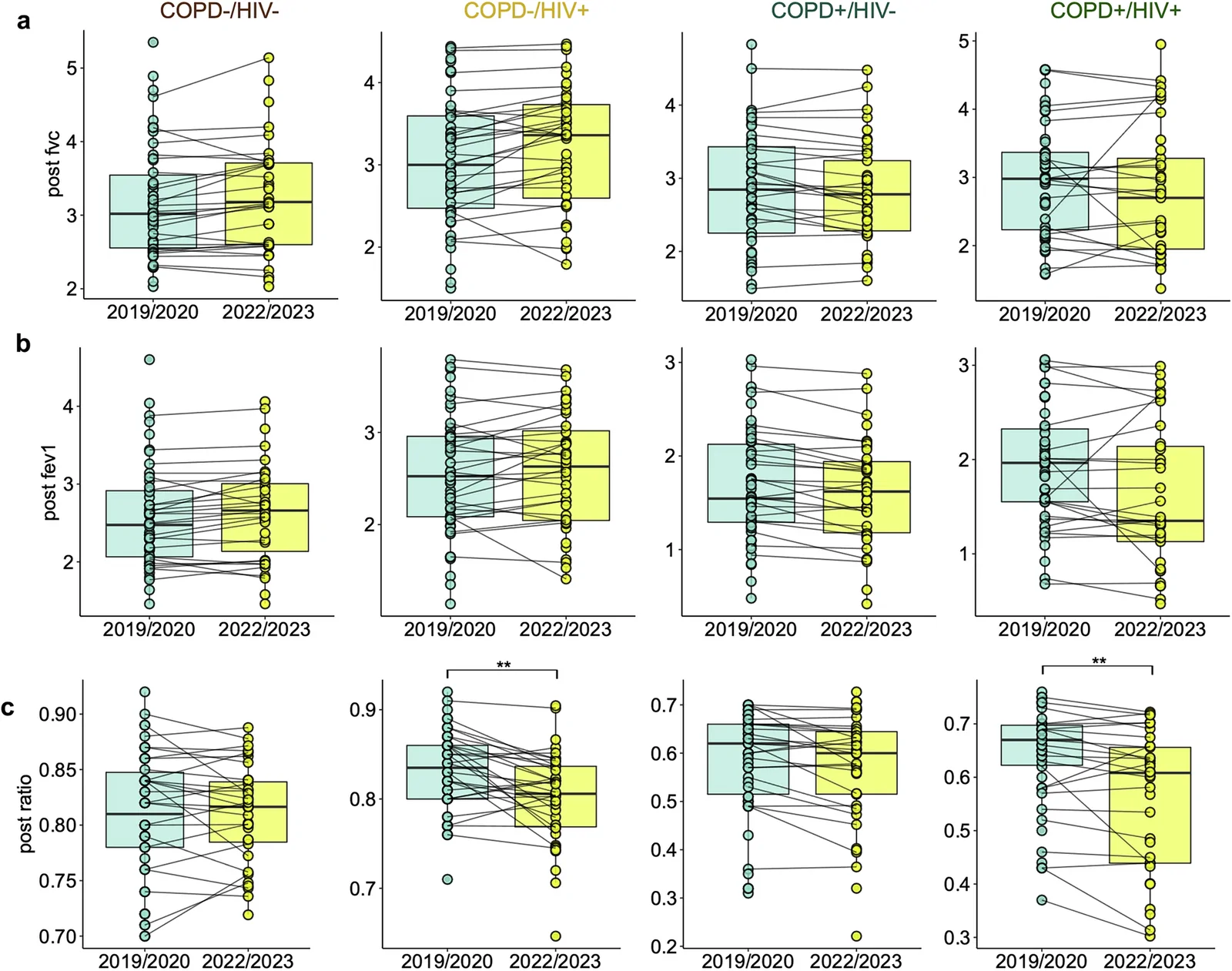
Longitudinal pulmonary function over a 2-year follow-up, stratified by clinical group. Boxplots show post-bronchodilator forced vital capacity (FVC; **a**), forced expiratory volume in 1 s (FEV₁; **b**), and FEV₁/FVC ratio (**c**) measured at baseline and follow-up. Analyses included *n* = 40 biologically independent participants with paired baseline and follow-up measurements in each clinical group (COPD-/HIV-, COPD-/HIV+, COPD+/HIV-, and COPD+/HIV+). Each participant constitutes the biological unit of study, and no technical replicates were treated as independent observations. Points represent individual participants, and gray lines connect repeated measurements from the same participant. Boxplots show the median (center line), interquartile range (25th–75th percentiles; box), and whiskers extending to the most extreme data points within 1.5 × the interquartile range. Statistical comparisons between baseline and follow-up measurements within each clinical group were performed using two-sided paired Wilcoxon signed-rank tests, with Benjamini–Hochberg false discovery rate (FDR) correction for multiple comparisons. Statistical significance is denoted as *P* < 0.05 (*), *P* < 0.01 (**), and *P* < 0.001 (***). Source data are provided as a Source Data file.

To characterize longitudinal lung function trajectories, we performed post-bronchodilator spirometry at baseline in 200 adults recruited between February 2018 and February 2021 and repeated spirometry in 160 participants after a median follow-up of approximately 2 years (April 2022–January 2023). The study design, participant flow, and multi-omics sampling strategy are summarized in Fig. 1. Baseline (visit 1) and follow-up (visit 2) clinical characteristics are presented in Tables 1 and 2, respectively. Table 3 shows the characteristics of participants contributing sputum, bronchoalveolar lavage (BAL), blood RNA-seq, CyTOF, and multiplex cytokine datasets. At baseline, COPD-positive participants were generally older, had lower body mass index (BMI), and more frequently reported respiratory medication use than COPD-negative participants, despite the cohort being predominantly non-smoking. Among individuals living with HIV, viral suppression was common, with most participants receiving highly active antiretroviral therapy, consistent with a clinically stable treated HIV population. A history of pulmonary tuberculosis remained more frequent among HIV+/COPD+ participants than among HIV+/COPD- participants. Longitudinal analysis of post-bronchodilator pulmonary function demonstrated within-group declines in the FEV₁/FVC ratio among HIV-positive participants, reaching statistical significance in the COPD-/HIV+ (raw *P* = 0.00343, BH *q* = 0.0137) and COPD+/HIV+ (raw *P* = 0.00822, BH *q* = 0.0164) groups (paired Wilcoxon signed-rank tests; Fig. 2); and non-significant in COPD-/HIV-: *P* = 0.684 and COPD+/HIV+: raw *P* = 0.0320, BH *q* = 0.0427. In contrast, post-bronchodilator FEV₁ and FVC remained relatively stable across all clinical groups. To determine whether these longitudinal changes differed according to clinical status, we modeled baseline-to-follow-up changes (Δ values) using multivariable linear regression adjusting for age, sex, BMI, and smoking status. After adjustment, no statistically significant between-group differences were identified for changes in post-bronchodilator FEV₁, FVC, or FEV₁/FVC ratio (Supplementary Figs. S1 and S2). These findings indicate that although HIV-positive groups exhibited significant within-group declines in airflow limitation over time, the magnitude of lung function change was not independently associated with HIV/COPD status after accounting for measured clinical covariates.

**Table 1:** Socio-demographic and clinical characteristics of the study participants at baseline

Cohort year of follow-up	2020 (*N* = 200)			
Characteristic	HIV+/COPD+ (*n* = 50)	HIV+/COPD- (*n* = 50)	HIV-/COPD+ (*n* = 50)	HIV-/COPD- (*n* = 50)
Sex	*P* = 0.2933			
Female	19 (38%)	25 (50%)	16 (32%)	22 (44%)
Male	31 (62%)	25 (50%)	34 (68%)	28 (56%)
Age group (Years)	*P* = 0.0002			
35–39	03 (06%)	04 (08%)	00 (00%)	01 (02%)
40–44	08 (16%)	10 (20%)	04 (08%)	08 (16%)
45–54	19 (38%)	21 (42%)	09 (18%)	27 (54%)
>55	20 (40%)	15 (30%)	37 (74%)	14 (28%)
Median age (IQR)	54.7 (47.9–61.5)	51.0 (46.9–56.8)	54.6 (48.4–63.7)	52.3 (47.3–61.3)
Body mass index (BMI: Kg/m^2^)	*P* = 0.00003			
<18.5	11 (22%)	01 (02%)	18 (36%)	02 (04%)
18.5–24.9	29 (58%)	37 (74%)	20 (40%)	29 (58%)
25.0–29.9	06 (12%)	07 (14%)	10 (20%)	16 (32%)
>30.0	04 (08%)	05 (10%)	02 (04%)	03 (06%)
Median BMI (IQR)	21.3 (19.3–24.8)	22.2 (20.5–26.6)	21.9 (19.1–25.7)	22.1 (20.0–26.6)
Smoking history	*P* = 0.00053			
Non-smoker	24 (48%)	35 (70%)	23 (46%)	44 (88%)
Daily smoker	03 (06%)	03 (06%)	02 (04%)	01 (02%)
Occasional smoker	22 (44%)	12 (24%)	25 (50%)	05 (10%)
Unknown	01 (02%)	00 (00%)	00 (00%)	00 (00%)
Pulmonary tuberculosis (TB)	*P* = 0.0002			
Yes	12 (24%)	04 (08%)	01 (02%)	01 (02%)
No	38 (76%)	46 (92%)	49 (98%)	49 (98%)
Biomass exposure	*P* = 0.61803			
Wood	43 (86%)	46 (92%)	47 (94%)	47 (94%)
Charcoal	05 (10%)	02 (04%)	02 (04%)	03 (06%)
Cow dung	02 (04%)	02 (04%)	01 (02%)	00 (00%)
Missing	00 (00%)	00 (00%)	00 (00%)	00 (00%)
Respiratory medication	*P*(antibiotics) = 0.00022; *P*(inhaled salbutamol) = 0.000057; *P*(oral prednisolone) = 0.0052			
Antibiotics >2weeks before study	05 (10%)	02 (04%)	16 (32%)	02 (04%)
Inhaled salbutamol use	05 (10%)	00 (00%)	11 (22%)	00 (00%)
Oral prednisone use	02 (04%)	02 (04%)	11 (22%)	00 (00%)
Lung function parameters				
Post BD FEV1 (L), Median (IQR)	1.97 (1.55–2.34)	2.53 (2.08–2.96)	1.55 (1.29–2.15)	2.48 (2.06–2.92)
Post BD FVC (L), Median (IQR)	2.96 (2.38–3.47)	3.00 (2.49–4.17)	2.98 (2.44–3.44)	2.95 (2.45–3.48)
Post BD ratio, Median (IQR)	0.69 (0.62–0.80)	0.79 (0.70–0.84)	0.69 (0.62–0.78)	0.79 (0.66–0.84)
FEV1 % predicted, Median (IQR)	89.50 (68.0–108.0)	103.0 (87.0–115.00)	88.50 (68.0–108.0)	103.0 (82.0–116.0)
FVC % predicted, Median (IQR)	104.0 (89.8–117.50)	108.5 (101.8–115.80)	102.5 (88.5–127.3)	110.0 (98.0–122.0)
Degree of airflow limitation				
No obstruction	00 (00%)	50 (100%)	00 (00%)	50 (100%)
Mild obstruction (GOLD 1)	28 (56%)	00 (00%)	21 (42%)	00 (00%)
Moderate obstruction (GOLD 2)	15 (30%)	00 (00%)	22 (44%)	00 (00%)
Severe obstruction (GOLD 3)	07 (14%)	00 (00%)	07 (14%)	00 (00%)
Very severe obstruction (GOLD 4)	00 (00%)	00 (00%)	00 (00%)	00 (00%)

Data are presented as *n* (%) for categorical variables and median (IQR) for continuous variables. Categorical variables were compared using two-sided Pearson’s chi-square tests. Continuous variables were compared across groups using two-sided Kruskal–Wallis tests. No adjustment for multiple comparisons was applied because these analyses were descriptive. Lung function parameters and degree of airflow limitation are presented descriptively without between-group hypothesis testing because COPD status was defined using spirometric measurements. Source data are provided as a Source Data file.

**Table 2:** Socio-demographic and clinical characteristics of the study participants at follow-up

Cohort year of follow-up	2022 (*N* = 160)			
Characteristic	HIV+/COPD+ (*n* = 40)	HIV+/COPD- (*n* = 40)	HIV-/COPD+ (*n* = 40)	HIV-/COPD- (*n* = 40)
Sex	*P* = 0.1175			
Female	18 (45%)	17 (43%)	10 (25%)	20 (50%)
Male	22 (55%)	23 (57%)	30 (75%)	20 (50%)
Age group (Years)	*P* = 0.0677			
35–39	02 (05%)	00 (00%)	00 (00%)	00 (00%)
40–44	03 (08%)	02 (05%)	02 (05%)	05 (13%)
45–54	13 (33%)	16 (40%)	06 (15%)	14 (35%)
>55	22 (55%)	22 (55%)	32 (80%)	21 (53%)
Median age (IQR)	55.5 (47.5–60.6)	56.7 (50.5–61.6)	65.1 (57.9–69.8)	55.8 (52.0–65.9)
Body mass index (BMI: Kg/m^2^)	*P* = 0.0001			
<18.5	17 (43%)	05 (13%)	15 (38%)	02 (05%)
18.5–24.9	18 (45%)	26 (65%)	23 (58%)	22 (55%)
25.0–29.9	04 (10%)	05 (13%)	01 (02%)	12 (30%)
>30.0	01 (02%)	04 (10%)	01 (02%)	04 (10%)
Median BMI (IQR)	19.7 (17.2–23.3)	21.2 (19.4–23.7)	19.0 (17.3–20.7)	23.5 (21.0–27.0)
Smoking history	*P* = 0.0062			
Non-smoker	28 (70%)	30 (75%)	22 (55%)	37 (93%)
Daily smoker	06 (15%)	08 (20%)	11 (28%)	03 (07%)
Occasional smoker	04 (10%)	02 (05%)	07 (17%)	00 (00%)
Unknown	02 (05%)	00 (00%)	00 (00%)	00 (00%)
Pulmonary tuberculosis (TB)	*P* = 0.0030			
Yes	10 (25%)	01 (03%)	03 (08%)	01 (02%)
No	30 (75%)	39 (97%)	37 (92%)	39 (93%)
Biomass exposure	*P* = 0.1057			
Wood	27 (68%)	35 (88%)	33 (83%)	37 (93%)
Charcoal	10 (25%)	05 (13%)	05 (13%)	02 (05%)
Cow dung	03 (08%)	00 (00%)	01 (03%)	00 (00%)
Missing	00 (00%)	00 (00%)	01 (03%)	01 (03%)
Respiratory medication	*P*(antibiotics) = 0.0004; *P*(inhaled salbutamol) = 0.5672; *P*(oral prednisolone) = 0.1044			
Antibiotics >2weeks before study	12 (30%)	01 (03%)	10 (25%)	00 (00%)
Inhaled salbutamol use	01 (03%)	00 (00%)	01 (03%)	00 (00%)
Oral prednisone use	03 (08%)	00 (00%)	01 (03%)	00 (00%)
Lung function parameters				
Post BD FEV1 (L), Median (IQR)	1.36 (1.13–2.21)	2.63 (2.03–3.04)	1.56 (1.18–1.88)	2.58 (2.01–2.97)
Post BD FVC (L), Median (IQR)	2.78 (1.95–3.52)	3.36 (2.52–3.75)	2.71 (2.26–3.14)	3.15 (2.58–3.71)
Post BD ratio, Median (IQR)	0.61 (0.44–0.66)	0.81 (0.77–0.84)	0.60 (0.51–0.64)	0.81 (0.76–0.84)
FEV1 % predicted, Median (IQR)	71.5 (43.5–88.0)	105 (94.0–115.5)	72.0 (54.0–84.0)	109.5 (98.5–120)
FVC % predicted, Median (IQR)	102 (71.3–113.0)	106 (99.0–117.5)	100 (85.5–118.8)	107.5 (96.25–115)
Degree of airflow limitation				
No obstruction	00 (00%)	40 (100%)	00 (00%)	40 (100%)
Mild obstruction (GOLD 1)	23 (58%)	00 (00%)	16 (40%)	00 (00%)
Moderate obstruction (GOLD 2)	07 (18%)	00 (00%)	16 (40%)	00 (00%)
Severe obstruction (GOLD 3)	09 (23%)	00 (00%)	06 (15%)	00 (00%)
Very severe obstruction (GOLD 4)	01 (03%)	00 (00%)	02 (05%)	00 (00%)

Data are presented as *n* (%) for categorical variables and median (IQR) for continuous variables. Categorical variables were compared using two-sided Pearson’s chi-square tests. Continuous variables were compared across groups using two-sided Kruskal–Wallis tests. No adjustment for multiple comparisons was applied because these analyses were descriptive. Lung function parameters and degree of airflow limitation are presented descriptively without between-group hypothesis testing because COPD status was defined using spirometric measurements. Source data are provided as a Source Data file.

**Table 3:** Characteristics of participants contributing sputum, bronchoalveolar lavage (BAL), blood RNA-seq, CyTOF and multiplex cytokine datasets

Characteristic	Sputum (*N* = 100)	BAL (*N* = 55)	Blood (*N* = 24)	CyTOF (*n* = 90)	48-Plex (*n* = 37)
Disease status	*P* = 0.7136				
HIV+/COPD+	26 (26%)	11 (20%)	11 (46%)	23 (26%)	08 (22%)
HIV-/COPD+	23 (23%)	15 (33%)	06 (25%)	26 (29%)	10 (27%)
HIV+/COPD-	26 (26%)	12 (22%)	03 (13%)	21 (23%)	10 (27%)
HIV-/COPD-	25 (25%)	17 (26%)	04 (17%)	20 (22%)	09 (24%)
Sex	*P* = 0.8824				
Female	39 (39%)	20 (36%)	08 (33%)	29 (32%)	12 (32%)
Male	61 (61%)	35 (64%)	16 (67%)	61 (68%)	25 (68%)
Age group (Years)	*P* = 0.7833				
35–39	02 (02%)	01 (02%)	00 (00%)	00 (00%)	00 (00%)
40–44	08 (08%)	04 (07%)	00 (00%)	05 (06%)	02 (05%)
45–54	22 (22%)	18 (33%)	08 (33.3%)	27 (30%)	12 (32%)
>55	68 (68%)	32 (58%)	16 (66.7%)	58 (64%)	23 (62%)
Median age (IQR)	58.7 (52.4–67.5)	57.2 (50.2–66.2)	57.6 (53.1–64.3)	58.5 (52.0–68.0)	57.9 (50–67.1)
Body mass index (BMI: Kg/m^2^)	*P* = 0.9893				
<18.5	25 (25%)	15 (27%)	06 (25%)	26 (29%)	09 (24%)
18.5–24.9	54 (54%)	27 (49%)	15 (63%)	47 (52%)	21 (57%)
25.0–29.9	12 (12%)	06 (11%)	02 (08%)	10 (11%)	05 (14%)
>30.0	09 (09%)	07 (13%)	01 (04%)	07 (8%)	02 (05%)
Median BMI (IQR)	20.52 (18.40–24.11)	20.12 (17.8–24.0)	20.3 (18.6–23.2)	20.1 (17.7–21)	20.2 (18.5–24.1)
Smoking history	*P* = 0.1208				
Non-smoker	79 (79%)	30 (54.6%)	13 (54%)	59 (66%)	24 (65%)
Daily smoker	12 (12%)	11 (20.0%)	06 (25%)	18 (20%)	06 (16%)
Occasional smoker	06 (6%)	10 (18.2%)	04 (17%)	11 (12%)	07 (19%)
Unknown	03 (3%)	04 (7.3%)	01 (04%)	02 (2%)	00 (00%)
Pulmonary tuberculosis (TB)	*P* = 0.5788				
Yes	11 (11%)	05 (09%)	02 (08%)	06 (7%)	01 (03%)
No	89 (89%)	50 (91%)	22 (92%)	84 (93%)	36 (97%)
Biomass exposure	*P* = 0.7071				
Wood	78 (78%)	44 (80.0%)	18 (75%)	77 (86%)	31 (84%)
Charcoal	15 (15%)	04 (7.27%)	04 (17%)	09 (10%)	03 (8%)
Cow dung	01 (01%)	01 (1.82%)	00 (00%)	01 (01%)	01 (03%)
Missing	06 (06%)	06 (10.9%)	02 (08%)	03 (03%)	02 (05%)
Respiratory medication					
Antibiotics >2weeks before study	01 (01%)	00 (00%)	01 (04%)	01 (01%)	01 (03%)
Inhaled salbutamol use	01 (01%)	01 (02%)	02 (08%)	02 (02%)	01 (03%)
Oral prednisone use	03 (03%)	01 (02%)	02 (08%)	04 (4.4%)	01 (03%)
Lung function parameters	*Kruskal–Wallis P(FEV1)* = *0.5671, P(FVC)* = *0.2395; P(FEV1/FVC)* = *0.5023; P(FEV1 % predicted)* = *0.8162; P(FVC% predicted)* = *0.7844)*				
Post BD FEV1 (L), Median (IQR)	1.99 (1.39–2.69)	2.16 (1.59–2.78)	2.01 (1.42–2.73)	2.01 (1.5–2.7)	2.1 (1.5–2.91)
Post BD FVC (L), Median (IQR)	2.88 (2.25–3.53)	3.04 (2.54–3.83)	3.23 (2.49–4.17)	3.0 (2.4–3.7)	3.0 (2.5–3.8)
Post BD ratio, Median (IQR)	0.73 (0.58–0.82)	0.72 (0.62–0.82)	0.64 (0.58–0.77)	0.69 (0.6–0.8)	0.7 (0.6–0.82)
FEV1 % predicted, Median (IQR)	97.0 (60.5–113.5)	88.06 (55.7–93.4)	86.0 (69.8–100.8)	85 (66–104.5)	90 (67–113)
FVC % predicted, Median (IQR)	105.0 (89.0–118.0)	102 (90.5–107.0)	113 (91.8–117.8)	103 (88–116)	105 (93–118)
Degree of airflow limitation	*P* = 0.7071				
No obstruction	51 (51%)	29 (53%)	07 (29%)	41 (46%)	19 (51%)
Mild obstruction (GOLD 1)	24 (24%)	12 (22%)	09 (38%)	19 (21%)	06 (16%)
Moderate obstruction (GOLD 2)	10 (10%)	10 (18%)	05 (21%)	17 (19%)	07 (19%)
Severe obstruction (GOLD 3)	13 (13%)	04 (07%)	03 (13%)	12 (13%)	05 (14%)
Very severe obstruction (GOLD 4)	02 (02%)	00 (00%)	00 (00%)	01 (01%)	00 (00%)

Data are presented as *n* (%) for categorical variables and median (interquartile range [IQR]) for continuous variables. Disease status was classified according to HIV and COPD status: HIV+/COPD+, HIV-/COPD+, HIV+/COPD-, and HIV-/COPD-. COPD severity was classified according to the Global Initiative for Chronic Obstructive Lung Disease (GOLD) criteria based on post-bronchodilator FEV₁ percent predicted: GOLD 1 (mild, FEV₁ ≥80%), GOLD 2 (moderate, FEV₁ 50–79%), GOLD 3 (severe, FEV₁ 30–49%), and GOLD 4 (very severe, FEV₁ <30%). Comparisons of categorical variables across datasets were performed using two-sided Pearson’s chi-square tests. Continuous variables were compared across datasets using two-sided Kruskal–Wallis tests. No adjustment for multiple comparisons was applied to these descriptive comparisons. Statistical significance was defined as *P* < 0.05. Source data are provided as a Source Data file.

### Longitudinal sputum microbiome dynamics are dominated by temporal ecological turnover rather than HIV or COPD status

**Fig. 3:**
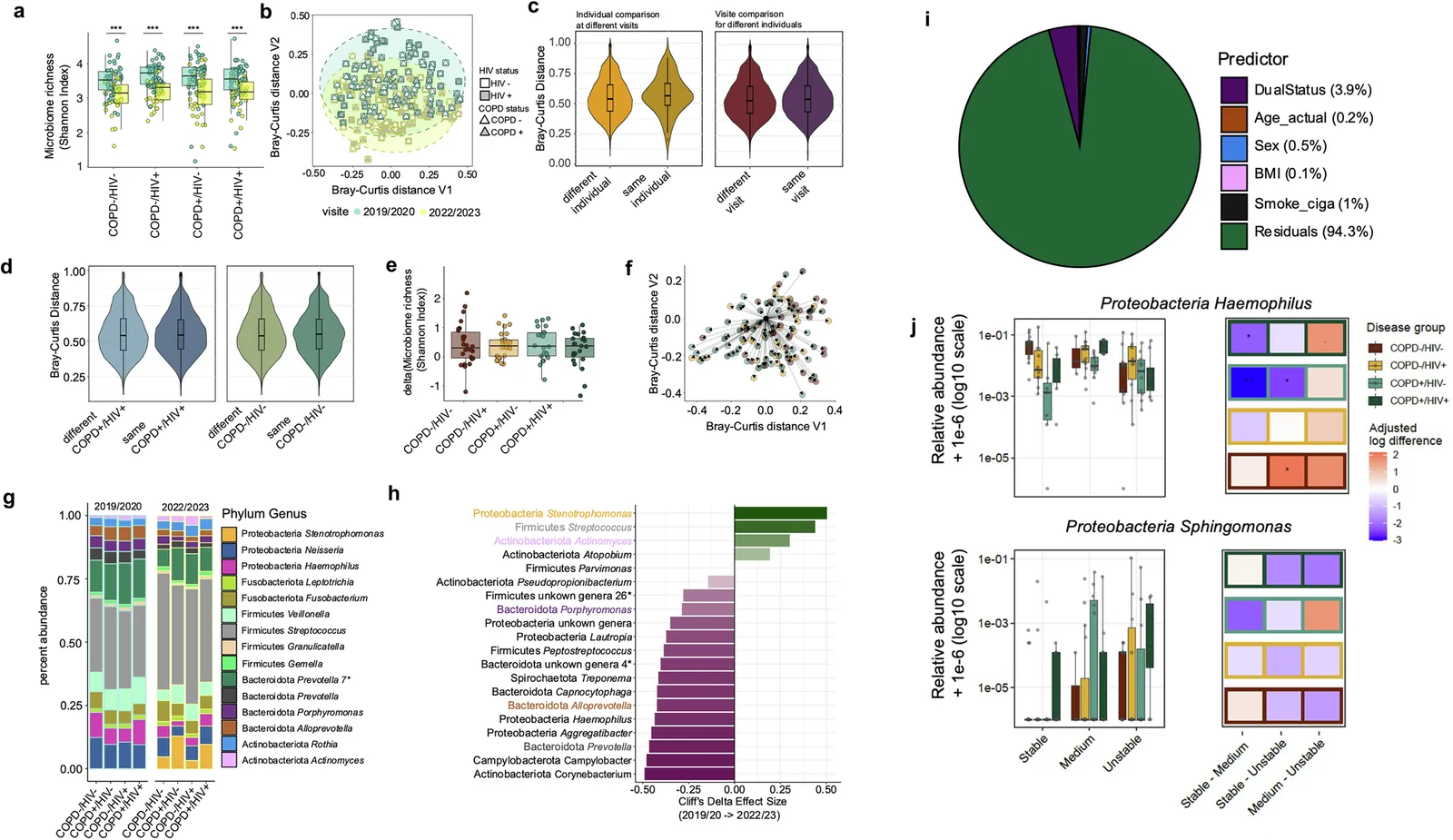
Longitudinal dynamics of the induced sputum microbiome and associations with HIV/COPD clinical status. **a** Shannon diversity at baseline and follow-up across the four clinical groups. Longitudinal differences were assessed using a linear mixed-effects model with participant ID as a random effect to account for repeated measurements. Statistical significance is denoted as *P* < 0.05 (*), *P* < 0.01 (**), and *P* < 0.001 (***). Shannon visit term raw *P* = 1.4e-12; BH across Shannon + Simpson ≈2.8e-12. **b** Principal coordinates analysis (PCoA) of Bray–Curtis dissimilarities at baseline and follow-up. The analysis included 356 samples: baseline, *n* = 50 per group; follow-up, COPD-/HIV- *n* = 36, COPD-/HIV+ *n* = 40, COPD+/HIV- *n* = 41 and COPD+/HIV+ *n* = 39. Community differences were assessed by PERMANOVA with permutations constrained by participant ID. **c**, **d** Bray–Curtis dissimilarities comparing samples within and between individuals and clinical groups; comparisons were performed using two-sided Wilcoxon rank-sum tests, with Benjamini–Hochberg false discovery rate (FDR) correction for multiple comparisons. **e** Change in Shannon diversity (ΔShannon = follow-up − baseline) across clinical groups. **f** PCoA of longitudinal trajectories; arrows connect paired samples from individual participants. **g** Relative abundances of the 15 most abundant bacterial genera by visit and clinical group. **h** Follow-up-associated bacterial taxa; bars show Cliff’s δ. **i** Variance in longitudinal Bray–Curtis distance explained by clinical and demographic variables. **c**–**i** Involving longitudinal paired analyses included *n* = 101 biologically independent participants: COPD-/HIV-, *n* = 26; COPD-/HIV+, *n* = 28; COPD+/HIV-, *n* = 24; and COPD+/HIV+, *n* = 23. **j** Genera with microbiome stability × clinical-group interactions (FDR < 0.10) among the same *n* = 101 participants, classified as stable (*n* = 34), medium (*n* = 34), or unstable (*n* = 33). Boxplots show the median (center line), interquartile range (box), and whiskers extending to 1.5 × the interquartile range; points represent individual participants. Heatmaps show FDR-adjusted pairwise comparisons. Each participant constitutes the biological unit of study; no technical replicates were treated as independent observations. Source data are provided as a Source Data file.

Longitudinal 16S rRNA gene sequencing demonstrated that sputum microbial community dynamics were driven predominantly by temporal ecological turnover rather than by HIV or COPD status. Alpha diversity declined between visits across all clinical groups (Fig. 3A). A linear mixed-effects model revealed significant visit effects on the Shannon index (Shannon visit term raw *P* = 1.4e-12; BH-adj *P* across Shannon + Simpson ≈2.8e-12), accounting for sex, age, BMI, and participant ID (random effect). Principal coordinate analysis of Bray–Curtis dissimilarities demonstrated a significant visit-associated shift in overall community composition. PERMANOVA analysis was performed, adjusting for patient ID, age, sex, and dual disease status. Results show a significant effect of visit on community composition (*F* = 2.001, *R*^2^ = 0.03251, *P* = 0.001), indicating temporal changes in the microbiome structure (Fig. 3B). Despite substantial temporal turnover, Bray–Curtis dissimilarity across visits was comparable between samples obtained from the same individual and those compared between different individuals, providing little evidence for preservation of individual-specific microbial signatures over the follow-up period (Fig. 3C). Statistical comparisons using the Wilcoxon rank-sum test, with Benjamini–Hochberg FDR correction, revealed a significant difference in sample similarity for time-point comparisons [*W* = 96, 706, 168, raw *P* = 4.37e-42; BH-adj (family of 3 candidates: same_visit/same_Dualdisease/same_healthy) = 1.31e-41], and for within-individual comparisons over time (*W* = 806, 63.5, raw *P* = 3.31e-4). After adjustment for age, sex, body mass index (BMI), smoking status, and disease group, neither overall alpha diversity nor longitudinal changes in Shannon diversity (ΔShannon) differed significantly according to HIV/COPD status (Fig. 3D–F). Temporal ecological turnover was accompanied by reproducible changes in the relative abundance of several bacterial genera. *Stenotrophomonas*, *Streptococcus*, *Actinomyces*, and *Atopobium* increased over time, whereas *Corynebacterium*, *Campylobacter, Haemophilus*, and *Prevotella* declined (Fig. 3G, H). Genus-level paired analyses identified multiple taxa exhibiting significant visit-to-visit abundance changes (|log₂FC| > 1; Supplementary Fig. S3), while linear mixed-effects models confirmed consistent population-wide increases in *Streptococcus* and decreases in *Kingella*, *Prevotella*, and *Veillonella* (Supplementary Fig. S4). Importantly, these temporal trajectories were similar across clinical groups. Only *Firmicutes Eubacterium nodatum* exhibited evidence of a disease-specific longitudinal pattern, increasing preferentially among HIV+/COPD+ participants, whereas all remaining taxa demonstrated comparable temporal behavior irrespective of HIV or COPD status (Supplementary Fig. S4).

### Characterization of shared longitudinal sputum microbiome compositional drift

To characterize longitudinal microbial changes independently of disease status, we first analyzed paired COPD⁻/HIV⁻ participants as internal sentinels of temporal compositional change (Supplementary Table 1). Between baseline and follow-up, genera including *Prevotella, Haemophilus, Aggregatibacter, Campylobacter*, and *Veillonella declined*, whereas *Actinomyces, Atopobium, Abiotrophia*, and *Shuttleworthia* increased (Supplementary Table 1). These compositional shifts were accompanied by reduced alpha diversity. Among paired healthy controls, Shannon diversity declined significantly between visits (paired t-test, *P* = 0.007), and linear mixed-effects modeling confirmed an independent effect of visit after adjustment for age (visit coefficient = − 0.49 ± 0.10 SE, *P* = 2.4 × 10⁻⁶), while age remained positively associated with baseline diversity (*β* = + 0.015, *P* = 0.005; Supplementary Table 2). To account for shared longitudinal compositional drift, we derived composite *bloomer* and *decreaser* covariates from genera exhibiting consistent temporal changes in healthy controls (Supplementary Fig. S5 and Supplementary Table 1) and incorporated these into subsequent longitudinal models. Adjustment attenuated, but did not eliminate, the visit effect (visit coefficient = − 0.33 ± 0.13 SE, *P* = 0.012), indicating that shared compositional drift explained part, but not all, of the observed temporal decline in diversity (Supplementary Table 2). Temporal trajectories were consistent across clinical groups. No visit × DualStatus interaction was detected for Shannon diversity (likelihood-ratio test, *χ*^2^ = 0.94, *df* = 3, *P* = 0.816), and no genus-level interaction remained significant following false-discovery correction across 96 genera (Supplementary Fig. S6 and Supplementary Table 3). Similarly, metadeconfoundR analyses identified no evidence that visit confounded disease-associated microbial differences across 375 comparisons (Supplementary Fig. S7). Together, these findings indicate that shared longitudinal compositional drift was adequately controlled for and did not account for the disease-associated microbiome patterns reported below.

### Disease-specific longitudinal behavior of individual taxa

To determine whether baseline sputum microbiome composition influenced subsequent ecological change, we performed baseline-delta modeling. Twelve genera demonstrated disease-modified baseline-follow-up relationships (FDR < 0.1), indicating that disease status selectively influenced the longitudinal behavior of individual taxa despite broadly conserved community trajectories. For example, *Staphylococcus* exhibited a positive baseline-delta relationship in HIV+/COPD+ participants, whereas *Moraxella, Gemella, and Aggregatibacter* displayed disease-dependent regression-to-the-mean patterns (Supplementary Fig. S8). Despite these taxon-specific differences, multivariable analyses showed no significant effect of HIV/COPD status on overall microbiome turnover. Bray–Curtis shift magnitude did not differ between clinical groups, and demographic variables together explained <4% of longitudinal variation. In contrast, visit (time) accounted for the largest proportion of explained variance (Model 1: *R*^2^ = 5.21%, *P* = 0.001), with age contributing a smaller independent effect, while sex, BMI, smoking status, and disease status explained little additional variation (Supplementary Figs. S9–S11 and Fig. 3I). Likewise, longitudinal changes in microbial diversity were not associated with contemporaneous changes in lung function after adjustment for clinical covariates (Supplementary Fig. S12). Together, these findings indicate that longitudinal sputum microbiome dynamics were dominated by shared temporal ecological turnover, with disease status influencing the trajectories of only a limited number of bacterial taxa. These observations motivated subsequent analyses examining whether clinically relevant microbial differences preferentially emerged during periods of ecological instability.

### Disease-specific taxonomic signatures characterize sputum microbiome instability states

To examine whether longitudinal ecological instability differed according to clinical status, participants with paired visit 1 and visit 2 microbiome profiles (*n* = 101) were stratified into stable (*n* = 34), medium (*n* = 34), and unstable (*n* = 33) groups according to tertiles of their within-individual Bray–Curtis dissimilarity. Although the magnitude of instability did not differ significantly among HIV/COPD groups, the taxa associated with increasing instability differed by disease group. At the OTU level, several taxa showed stability × DualStatus interactions (OTU255 *P* = 0.0117/0.0283; OTU25 *P* = 0.00206/0.00875), including OTU 255, which increased across the medium and unstable strata among COPD+/HIV+ participants, and OTU 25, which was enriched in the stable stratum among COPD-/HIV- controls (Supplementary Fig. S13). These analyses should be interpreted cautiously because some stability × DualStatus subgroups contained few participants. Following adjustment for visit-associated compositional drift, five of six representative OTUs retained statistical significance (Supplementary Table 4). Genus-level analyses identified *Haemophilus and Sphingomonas* as the only genera exhibiting significant microbiome stability-associated patterns after multiple-testing correction. *Haemophilus* was consistently enriched in medium and unstable microbiomes among HIV+/COPD+ participants, whereas *Sphingomonas* was most abundant in stable microbiomes, primarily reflecting a stability main effect rather than a disease-specific interaction (Fig. 3J and Supplementary Table 5). Post hoc estimated marginal means analyses confirmed these relationships, and the *Haemophilus* stability ×  DualStatus interaction remained significant after drift adjustment (Supplementary Table 5). Microbiome instability itself was not associated with clinical progression. Neither microbiome stability tertile nor continuous Bray–Curtis shift magnitude predicted 2-year changes in FEV₁, FVC, or FEV₁/FVC after adjustment for age, sex, BMI, and smoking (ΔFEV₁ *q* = 0.414; ΔFVC *q* = 0.559; Δratio *q* = 0.414; Supplementary Table 6). Together, these findings indicate that HIV-associated COPD is characterized not by greater microbiome instability, but by disease-specific taxonomic configurations within unstable communities. These observations identify candidate microbial states for subsequent integration with host immune and inflammatory phenotypes, prompting investigation of bacterial OMVs as potential microbial functional correlates.

### Sputum bacterial outer membrane vesicle abundance is not associated with microbial community structure

Across the cohort, sputum OMV concentrations were not associated with microbial alpha diversity (Supplementary Fig. S14A), HIV or COPD status (Supplementary Fig. S14B), or overall microbial community composition (PERMANOVA, *P* = 0.170/*P* = 0.186; Supplementary Fig. S14D). Consistent with these observations, multivariable models adjusting for age and sex identified no independent association between disease status and OMV abundance. Confounder-aware genus-level analyses similarly identified no bacterial genera significantly associated with OMV concentration after multiple-testing correction, although modest, non-significant trends were observed for *Prevotella*, *Stenotrophomonas*, and *Parvimonas* (Supplementary Fig. S14C). Together, these findings indicate that bulk sputum OMV burden was not consistently associated with microbial diversity, taxonomic composition, or overall community structure in this cohort. Although bacterial OMVs possess well-established immunomodulatory properties, our findings suggest that total OMV abundance alone does not explain the microbial ecological patterns observed here. These results do not exclude functional effects of OMVs produced by specific bacterial taxa or differences in OMV molecular cargo but indicate that bulk OMV burden is unlikely to account for the disease-associated microbial features identified in this study. We therefore next investigated whether discrete microbial states were more closely associated with airway and systemic immune responses.

### Peripheral immune-cell composition shows selective HIV- and COPD-associated differences and taxon-specific microbial associations

**Fig. 4:**
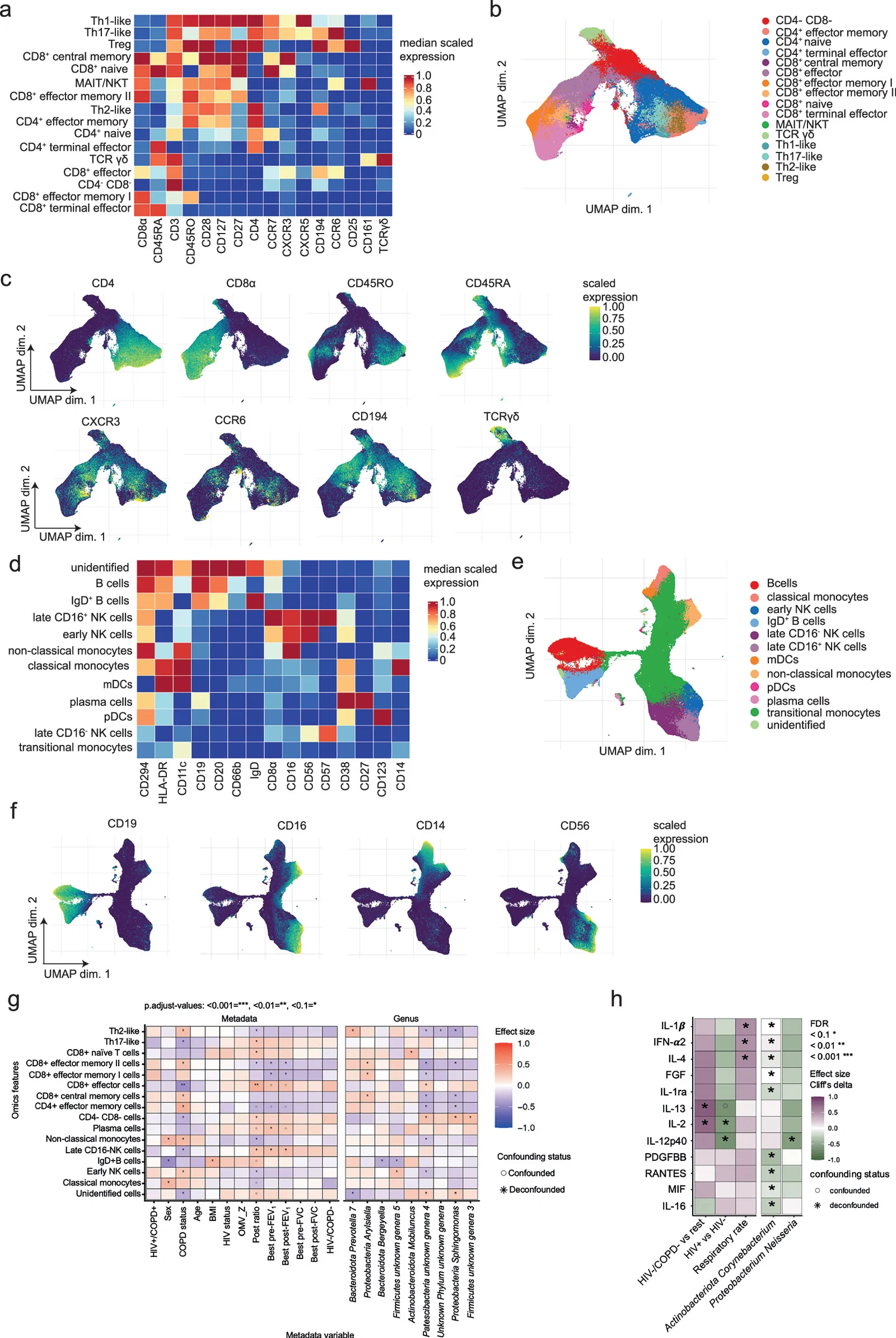
Airway immune landscape and associations with the induced sputum microbiome and inflammatory mediators. CyTOF characterization of T-cell populations showing normalized expression of 16 clustering markers (**a**), UMAP visualization of 16 T-cell clusters (**b**), and expression of representative lineage and activation markers (**c**). CyTOF characterization of non-T-cell populations showing normalized expression of 15 clustering markers (**d**), UMAP visualization of 13 non-T-cell clusters (**e**), and expression of representative lineage and functional markers (**f**). CyTOF analyses included *n* = 90 biologically independent participants. **g** Deconfounded associations between immune-cell populations and clinical variables or bacterial genera. **h** Deconfounded associations between sputum inflammatory mediators and clinical variables or bacterial genera; cytokine profiling included *n* = 37 biologically independent participants. In **g**, **h**, colors indicate Cliff’s δ effect sizes; open circles denote associations attenuated after adjustment for potential confounders and asterisks denote associations remaining significant after multivariable adjustment. Statistical significance was defined using Benjamini–Hochberg FDR correction (*FDR < 0.10; **FDR < 0.01*;* ***FDR < 0.001). Each participant constitutes the biological unit of study; no technical replicates were treated as independent observations. Source data are provided as a Source Data file.

High-dimensional CyTOF profiling identified eight major CD45⁺ immune compartments (Supplementary Fig. S15). Subcluster analysis resolved 16 T-cell subsets spanning naïve, memory, effector, regulatory, γδ, and helper-like phenotypes and 13 non-T cell subsets including different B cell, myeloid cell, and NK cell clusters (Fig. 4A–F). HIV-positive participants exhibited selective differences rather than broad redistribution of circulating immune populations, with lower frequencies of total NK cells, CD4⁺ effector-memory T cells, and total B cells, together with higher frequencies of IgD⁺ B cells and plasma cells. Most other immune populations were comparable between HIV groups (Supplementary Fig. S16). Similarly, COPD-positive participants had increased frequencies of total NK cells, CD4⁺ effector-memory T cells, Th1-like cells, regulatory T cells, and plasmacytoid dendritic cells, together with reduced CD8⁺ effector T cells and plasma cells, while the remaining populations showed no significant differences (Supplementary Fig. S17). Confounder-aware integration of immune-cell frequencies with sputum microbial genera identified discrete host–microbe association modules rather than widespread microbiota-immune relationships (Fig. 4G). Th2-like cells were positively associated with *Prevotella 7* and inversely associated with *Sphingomonas* and *Patescibacteria*-related taxa. Effector-memory T-cell populations also showed inverse associations with *Sphingomonas* and *Patescibacteria*, whereas naïve CD8⁺ T cells were associated with *Mobiluncus*. Additional taxon-specific associations involved double-negative T cells, non-classical monocytes, naïve B cells, and early NK cells. Several of these immune populations also correlated with post-bronchodilator FEV₁/FVC, linking microbial-associated immune phenotypes with airflow limitation (Fig. 4G). To further distinguish HIV- from COPD-associated effects, four additional confounder-aware subset analyses were performed comparing HIV and COPD strata separately (Supplementary Fig. S18). No immune-cell population remained independently associated with HIV status after multi-confounder adjustment. Where significant associations were observed, they involved COPD-related variables or specific microbial genera rather than HIV itself. This may reflect the predominantly treated and virologically suppressed nature of the cohort; however, the number of participants with both CyTOF and contemporaneous viral-load measurements was insufficient to directly test peripheral immune differences according to virological suppression status. Together, these findings indicate that HIV and COPD were associated with selective differences in peripheral immune-cell composition, while confounder-aware integration identified reproducible host–microbe-immune association modules linked to lung function. These observations are associative and hypothesis-generating and do not establish that airway microbial features directly remodel systemic immunity.

### Confounder-aware integration identifies selective associations between sputum microbial taxa and inflammatory cytokines

To investigate whether sputum microbial states were associated with local inflammatory programs, we quantified 48 cytokines in induced sputum and integrated cytokine concentrations with microbial genera and clinical variables using confounder-aware modeling (Fig. 4H). Clinical characteristics explained only a limited proportion of cytokine variation. HIV-positive participants had lower IL-12p40 and IL-2 concentrations than HIV-negative participants, whereas higher respiratory rate was associated with increased IL-4, IL-1β, and IFN-α2 concentrations. Healthy controls (HIV-/COPD-) also exhibited relatively higher IL-3 and IL-2 concentrations than the remaining clinical groups, while HIV/COPD dual positivity was not associated with a distinct global cytokine profile. Microbiome integration identified a limited number of reproducible taxon-specific associations. *Corynebacterium* abundance was inversely associated with multiple inflammatory mediators, including IL-6, MIF, RANTES, PDGF-BB, IL-1Ra, and IL-4, with additional borderline associations involving IL-1β, IFN-α2, and FGF (Fig. 4H). *Neisseria* abundance was inversely associated with IL-12p40, whereas no other bacterial genera remained significantly associated with cytokine concentrations after multiple-testing correction. Together, these findings indicate that airway microbial communities were associated with selective inflammatory pathways rather than generalized cytokine activation. Although these associations do not establish causality, they identify biologically coherent host–microbe interaction modules linking a limited number of bacterial genera with specific inflammatory mediators. We therefore next examined whether these selective inflammatory signatures were accompanied by coordinated changes in host airway gene expression.

### Airway compartment-specific transcriptomic analysis identifies induced sputum as the principal site of HIV-associated immune remodeling

**Fig. 5:**
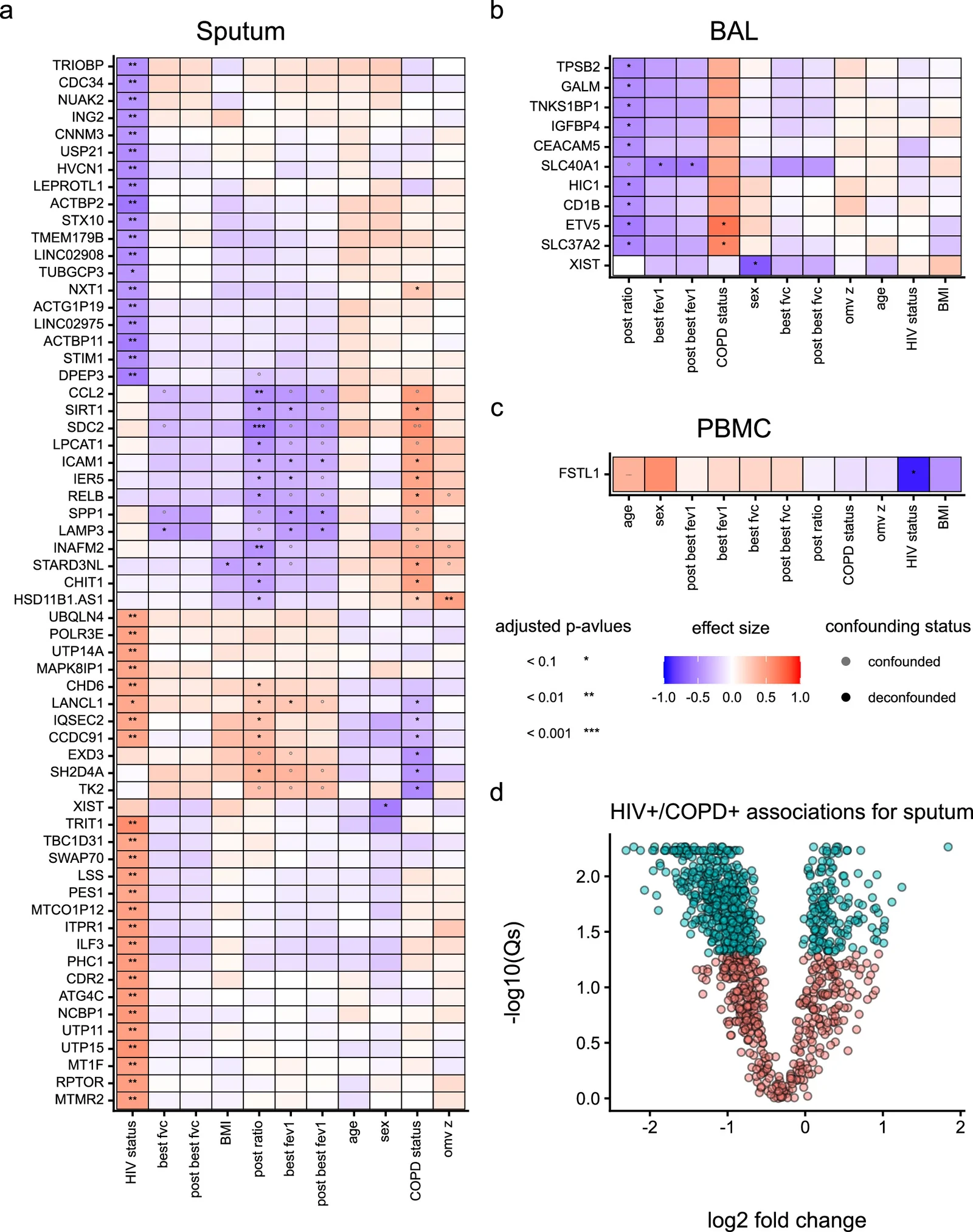
Compartment-specific transcriptional signatures and microbiome-host associations. Deconfounded associations between host gene expression and clinical characteristics in induced sputum (**a**; *n* = 80; HIV-, *n* = 39; HIV+, *n* = 41; COPD-, *n* = 42; COPD+, *n* = 38; COPD-/HIV-, *n* = 62; COPD+/HIV+, *n* = 18), bronchoalveolar lavage (**b**; *n* = 48; HIV-, *n* = 26; HIV+, *n* = 22; COPD-, *n* = 24; COPD+, *n* = 24; COPD-/HIV-, *n* = 38; COPD+/HIV+, *n* = 10), and peripheral blood mononuclear cells (**c**; *n* = 21; HIV-, *n* = 10; HIV+, *n* = 11; COPD-, *n* = 7; COPD+, *n* = 14; COPD-/HIV-, *n* = 13; COPD+/HIV+, *n* = 8). Sample sizes represent biologically independent participants. Associations were assessed using two-sided Wilcoxon tests for binary variables, Kruskal–Wallis tests for variables with more than two categories, and two-sided Spearman rank correlations for continuous variables, as appropriate. *P* values were adjusted for multiple testing using the Benjamini–Hochberg procedure. Colors indicate Cliff’s δ effect sizes. Open circles denote associations attenuated after adjustment for potential confounders, whereas asterisks denote associations remaining significant after multivariable adjustment (FDR < 0.10; *FDR < 0.01; **FDR < 0.001). **d** Differential gene-expression analysis of induced sputum (*n* = 80 biologically independent participants) was performed using DESeq2 with two-sided Wald tests, with *P* values adjusted for multiple testing using the Benjamini–Hochberg procedure. Each point represents a gene plotted according to log₂ fold change and -log₁₀ adjusted *P* value; differentially expressed genes were defined at FDR < 0.10. Each participant constitutes the biological unit of study; no technical replicates were treated as independent observations. Source data are provided as a Source Data file.

To determine whether the selective inflammatory signatures identified at the cytokine level extended to coordinated transcriptional programmes, we performed confounder-aware transcriptomic analyses of induced sputum (*n* = 80; HIV-, *n* = 39; HIV+, *n* = 41; COPD-, *n* = 42; COPD+, *n* = 38; Dualdisease-, *n* = 62; Dualdisease+, *n* = 18), BAL (*n* = 48; HIV-, *n* = 26; HIV+, *n* = 22; COPD-, *n* = 24; COPD+, *n* = 24; Dualdisease-, *n* = 38; Dualdisease+, *n* = 10), and PBMCs (*n* = 21; HIV-, *n* = 10; HIV+, *n* = 11; COPD-, *n* = 7; COPD+, *n* = 14; Dualdisease-, *n* = 13; Dualdisease+, *n* = 8) (Fig. 5). Among the three compartments, induced sputum exhibited more disease-associated transcriptional remodeling, whereas BAL and PBMCs showed comparatively limited changes. In induced sputum (Fig. 5A, D), HIV status was associated with broad inflammatory transcriptional programmes involving interferon signaling, lysosomal biology, antigen processing, and cellular stress responses. Multiple genes, including *LAMP3, CHIT1, RELB, IER5, HVCN1, SPP1, STARD3NL*, and *USP21*, remained significantly associated with HIV after adjustment for COPD status, lung function, age, sex, and BMI. COPD was associated with a more restricted transcriptional signature involving genes such as *SIRT1, ICAM1, IER5, RELB,* and *STARD3NL*, while declining post-bronchodilator FEV₁ and FEV₁/FVC were associated with increased expression of inflammatory genes including *CCL2, CHIT1, RELB, ICAM1*, and *SIRT1*. As expected, *XIST* expression was strongly associated with female sex, providing an internal biological control for the analytical framework. In contrast, BAL transcriptomes demonstrated relatively few disease-associated signals, with only a limited number of genes, including *TPSB2, GALM, CEACAM5, SLC40A1, CD1B*, and *ETV5*, associated with COPD or lung-function measures after confounder adjustment (Fig. 5B). PBMC transcriptomes showed even fewer significant associations, with *FSTL1* representing the principal transcript associated with HIV status (Fig. 5C). To isolate HIV-associated transcriptional effects within COPD-positive participants, we repeated the confounder-aware analysis comparing COPD+/HIV+ vs. COPD+/HIV- individuals. In sputum, 452 of 1127 genes remained significantly associated with HIV (BH-adjusted *q* < 0.1), including 11 genes at *q* < 0.01, and 97% (438/452) were classified as deconfounded (Supplementary Table 7). In contrast, no significant HIV-associated genes were identified in BAL or PBMCs after subsetting (Supplementary Tables 8 and 9), consistent with the reduced statistical power available in these smaller compartment-specific analyses.

### Selective microbial features align with coordinated airway transcriptional programmes

Having established induced sputum as the principal compartment exhibiting disease-associated transcriptional remodeling, we next asked whether these airway transcriptional programmes were associated with specific microbial features independently of HIV status, COPD status, lung function, and demographic characteristics. Using a confounder-aware integration approach, we identified a structured network of host–microbe associations centered on a limited number of bacterial genera rather than a widespread transcriptome-wide relationship (Supplementary Fig. S19). The strongest associations involved *Treponema, Peptostreptococcus, Gemella, Alloprevotella, Actinomyces*, and an unclassified *Patescibacteria lineage*. *Treponema-* and *Peptostreptococcus*-associated microbial states aligned with coordinated inflammatory gene modules, including genes involved in neutrophil activation, innate immune signaling, cytokine responses, and cellular stress pathways (for example, *SERPINE1, IFI27, CXCL16, PGLYRP1, CCR1, IL1RAP,* and *TREM1*). These microbial states were inversely associated with genes involved in RNA processing and cellular homeostasis, and they also aligned with lysosomal and autophagy-related pathways. The RNA features were prefiltered for dual-disease associations, and the microbial associations were then tested while controlling for the other metadata. Many of these associations remained significant after adjustment for HIV status, COPD status, lung function, age, sex, and BMI, indicating that they were not explained by the measured clinical covariates. Together, these findings identify reproducible host–microbe transcriptional modules linking a limited number of bacterial taxa with coordinated airway inflammatory programmes. These observational associations do not establish causality or directionality, but they provide biologically plausible targets for future mechanistic investigation. We therefore next examined whether these transcriptional programmes were accompanied by broader changes in microbial ecology and pathway activity across the clinical groups.

### Sputum immune-transcriptional remodeling occurs despite preserved overall microbial diversity

**Fig. 6:**
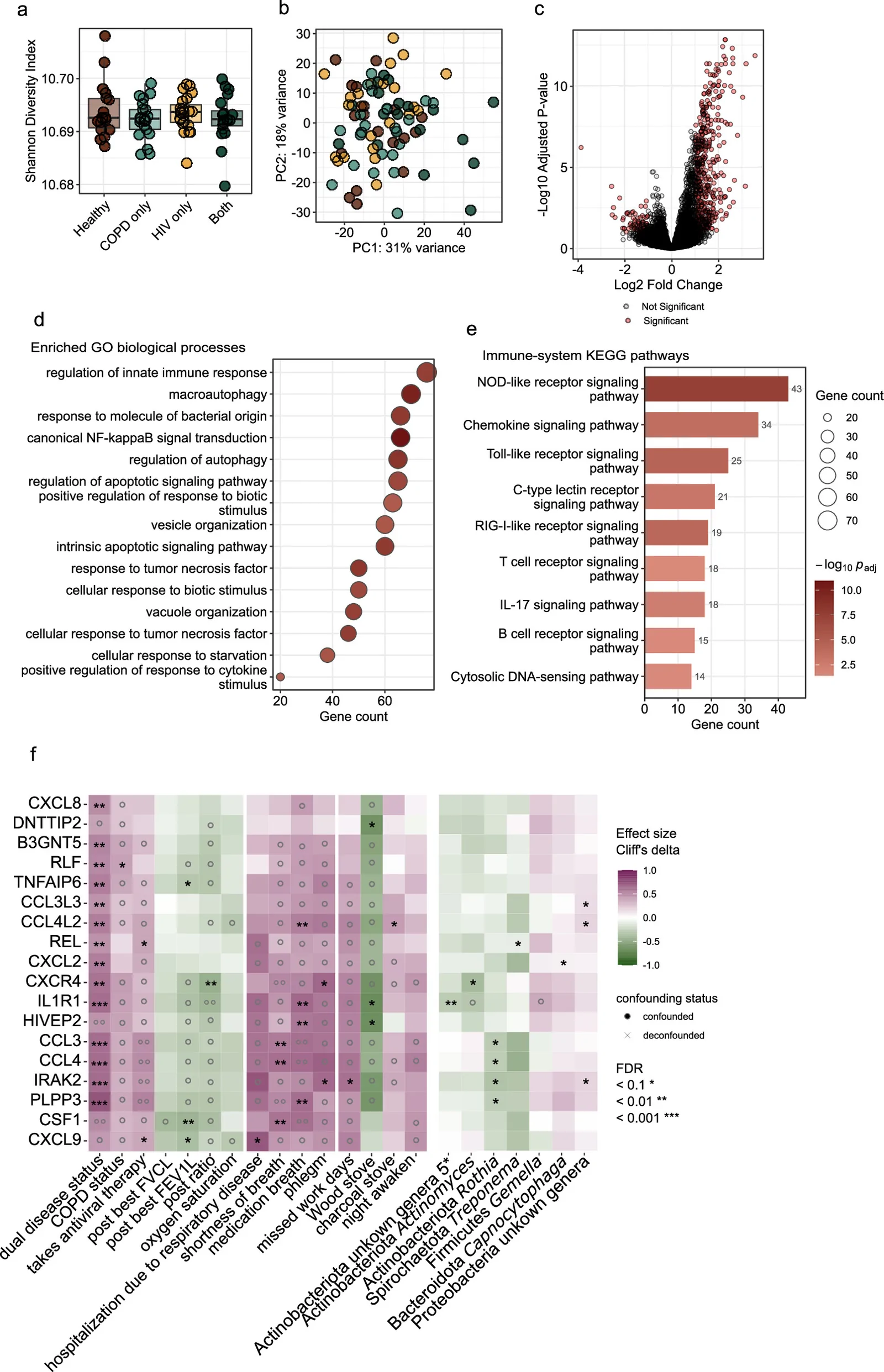
Integrated analysis of the induced-sputum microbiome and host transcriptome in HIV-associated COPD. **a** Sputum microbial Shannon diversity across clinical groups. Boxplots show the median (center line), interquartile range (box), and whiskers extending to 1.5 × the interquartile range; points represent individual participants. **b** Principal component analysis of induced-sputum bulk RNA-sequencing profiles across clinical groups; each point represents one participant. **a**, **b** Included *n* = 80 biologically independent participants: HIV-/COPD-, *n* = 19; HIV+/COPD-, *n* = 23; HIV-/COPD+, *n* = 20; and HIV+/COPD+, *n* = 18. **c** Differential gene-expression analysis comparing HIV+/COPD+ (*n* = 18) with HIV-/COPD- (*n* = 19) participants was performed using DESeq2 with two-sided Wald tests. *P* values were adjusted for multiple testing using the Benjamini–Hochberg procedure, and significant genes were defined at FDR < 0.10. Each point represents a gene. Gene Ontology Biological Process (**d**) and KEGG pathway (**e**) enrichment analyses of differentially expressed genes were performed using hypergeometric tests, with *P* values adjusted for multiple testing using the Benjamini–Hochberg procedure. Symbol size or bar length indicates gene count, and color indicates adjusted *P* value. **f** Deconfounded associations between inflammatory transcripts, clinical characteristics, and airway microbial genera in the induced-sputum analysis cohort (*n* = 80). Colors indicate Cliff’s δ effect sizes; open circles denote associations attenuated after adjustment for potential confounders and asterisks denote associations remaining significant after adjustment (FDR < 0.10; *FDR < 0.01; **FDR < 0.001). Each participant constitutes the biological unit of study; no technical replicates were treated as independent observations. Source data are provided as a Source Data file.

To determine whether the host–microbe transcriptional modules identified above reflected widespread disruption of sputum microbial ecology or occurred within relatively preserved microbial communities, we compared induced-sputum microbial diversity and transcriptomic profiles across clinical groups, focusing on participants with HIV-associated COPD (Fig. 6). Overall microbial alpha diversity was comparable between groups, with no significant differences in Shannon diversity (Fig. 6A), indicating that HIV-associated COPD was not accompanied by major changes in community diversity. In contrast, sputum transcriptomes exhibited distinct global expression patterns, with principal component analysis demonstrating separation of participants according to disease status (Fig. 6B), consistent with airway transcriptional remodeling, despite preserved global microbial diversity. Differential expression analysis identified numerous dysregulated genes in HIV+/COPD+ participants compared to HIV-/COPD- control group (Fig. 6C). Functional enrichment highlighted pathways related to immune activation, cytokine production, leukocyte migration, macrophage activation, interferon responses, and NF-κB signaling, together with enrichment of B-cell receptor, IL-17, Toll-like receptor, NOD-like receptor, RIG-I-like receptor, chemokine, and cytokine-cytokine receptor signaling pathways (Fig. 6D, E). To identify factors associated with these inflammatory programmes, we integrated airway gene expression with clinical variables and microbial genera using confounder-aware modeling (Fig. 6F). COPD status was positively associated with *RLF*, whereas antiretroviral therapy was associated with *CXCL9* expression. Lower post-bronchodilator FEV₁ was associated with increased *CXCL10, CSF1,* and *TNFAIP6* expression, while reduced FEV₁/FVC was associated with higher *TNFAIP6* expression. Additional inflammatory mediators, including *CXCL2, CXCR4, IL1R1, CCL3, CCL4,* and* CSF1*, were associated with dual HIV/COPD status or hospitalization history. After adjustment for demographic and clinical covariates, only a limited number of microbial genera remained associated with inflammatory gene expression, including positive associations of *Actinomyces, Rothia, and Treponema*, together with inverse associations involving *Capnocytophaga* and *an unclassified Proteobacteria lineage*. Together, these findings indicate that HIV-associated COPD is characterized by more airway immune-transcriptional remodeling despite preserved overall microbial diversity. Rather than broad disruption of the airway microbiome, a limited number of bacterial taxa consistently aligned with coordinated inflammatory transcriptional programmes, identifying reproducible host–microbe interaction modules for future mechanistic investigation.

### OMV-associated sputum transcriptional changes are subtle and enriched for metal-ion pathways

Participants were stratified into low, medium, and high sputum OMV groups to evaluate whether OMV burden was associated with microbial diversity or airway transcriptional responses. Shannon diversity did not differ across OMV strata (Supplementary Fig. S20A), and principal component analysis demonstrated no significant differences in global transcriptomic profiles (Supplementary Fig. S20B). Despite the absence of broad transcriptional differences, differential expression analysis identified a limited number of genes that differed between medium- and low-OMV groups, including *FTH1P20, CNMN3*, and *MTCYBP18* (Supplementary Fig. S20C). Gene ontology analysis showed enrichment of pathways related to metal-ion binding and intracellular metal-ion homeostasis (Supplementary Fig. S20D), while normalized expression profiles confirmed stepwise changes across OMV strata for *BOLA2B*, ENSG00000240103, and *MTCYBP18* (Supplementary Fig. S20E). Together, these findings indicate that variation in sputum OMV burden was associated with modest, pathway-specific transcriptional changes rather than broad alterations in microbial diversity or global airway gene expression. We therefore next investigated whether OMVs and microbial taxa were more closely associated with airway cellular composition using single-cell-guided deconvolution.

### Single-cell-guided deconvolution reveals limited and reference-sensitive variation in inferred airway cellular composition

To determine whether the immune-transcriptional programmes identified in induced sputum were accompanied by changes in airway cellular composition, we estimated cell-type proportions from bulk sputum RNA sequencing using reference-guided deconvolution. Our primary analysis used a BAL snRNA-seq reference comprising four donors from the same parent cohort that contributed induced-sputum samples and 5205 nuclei after quality control, annotated into 12 major cell populations (Table 4 and Supplementary Fig. S21). MuSiC-derived proportions showed no significant differences across either the four HIV/COPD groups (DualStatus) or OMV quartiles after correction for multiple testing (Supplementary Fig. S22). We additionally deconvolved the induced-sputum dataset using the Human Lung Cell Atlas (HLCA) as an independent external reference (Supplementary Fig. S23), allowing us to assess the sensitivity of inferred cellular composition to reference choice. We next formally tested disease-associated differences in individual inferred cell populations. In the HLCA analysis of the full induced-sputum cohort (*n* = 100), classical monocytes, conventional dendritic cells, and natural killer cells differed significantly across the four HIV/COPD groups after correction for multiple testing (classical monocyte *p*.adj = 0.00951 (**); conventional DC *p*.adj = 0.00951 (**); NK *p*.adj = 0.0495 (*); Supplementary Fig. S24). We then applied the same group-level testing framework to the BAL snRNA-seq/MuSiC estimates (*n* = 48). In contrast to the HLCA analysis, none of the 12 MuSiC-inferred cell populations differed significantly across HIV/COPD groups after multiple-testing correction (Supplementary Fig. S25). These findings indicate that disease-associated differences in individual inferred cell proportions were not uniformly reproduced across the two reference frameworks. We therefore extended the comparison to overall cellular composition (Supplementary Fig. S26). Using centered log-ratio (CLR)-transformed proportions, HLCA-derived composition differed significantly among HIV/COPD groups (PERMANOVA: *pseudo-F* = 2.286, *R*^2^ = 0.0667, *P* = 0.0063; 9999 permutations), without significant heterogeneity of multivariate dispersion (*P* = 0.0979). By contrast, BAL snRNA-seq/MuSiC-derived composition showed no significant global separation among HIV/COPD groups (*pseudo-F* = 0.988, *R*^2^ = 0.0631, *P* = 0.4641), with no evidence of heterogeneous dispersion (*P* = 0.9171). Notably, despite their different statistical significance, the proportion of compositional variance attributable to the HIV/COPD group was similar in magnitude between the HLCA and BAL/MuSiC analyses (*R*^2^ = 0.067 and 0.063, respectively). Thus, the reference-sensitivity analysis supports modest disease-associated compositional structure while showing that its statistical resolution depends on the deconvolution reference and available sample size, rather than reflecting large and consistently reproduced shifts in major airway cell populations.

**Table 4:** Clinical characteristics of participants in whom BAL single-cell RNAseq analysis was performed

SN	Sex	HIV	COPD	Post-FEV1	Post-FVC	Post ratio	% predicted FEV_1_
#1	M	−ve	−ve	3.49	4.20	0.831	107
#2	F	+ve	+ve	1.13	1.79	0.631	47
#3	M	−ve	+ve	1.72	2.55	0.675	49
#4	M	−ve	−ve	2.62	3.18	0.824	90

For subsequent OMV- and microbiome-cell association analyses, we retained the BAL/MuSiC framework as the primary deconvolution dataset. This choice was based on biological and study-design considerations. The BAL snRNA-seq reference was generated from participants within the same parent cohort as the induced-sputum samples and therefore provided a cohort-derived airway reference that preserves population-specific cellular states not necessarily represented in an external atlas. Moreover, MuSiC explicitly incorporates between-donor variation in the reference during estimation. The HLCA analysis was therefore used as an independent external reference-sensitivity analysis rather than as a replacement for the cohort-derived framework. Given differences in tissue source, cellular taxonomy, and reference construction, concordance of absolute cell fractions between the two approaches was not expected; instead, their comparison was used to determine the extent to which conclusions regarding cellular composition were sensitive to reference choice. Using this cohort-derived framework, we next examined whether BAL/MuSiC-inferred cellular proportions were related to OMV burden. Simple Spearman correlations between continuous OMV z-scores and individual inferred cell proportions did not remain significant after Benjamini–Hochberg correction (Supplementary Table 10; 12/12 cell types NS, *p*adj range 0.814–0.992). However, after adjustment for smoking, age, sex, and BMI in cell type-wise linear regression models, OMV burden was positively associated with the inferred proportion of proliferating alveolar macrophages (*β* = 0.0678, SE = 0.0251, *t* = 2.71, *P* = 0.0085, BH-adjusted *P* = 0.0085), whereas no significant adjusted associations were observed for the remaining inferred cell populations (Supplementary Table 11). Thus, although OMV burden was not associated with broad shifts in inferred cellular composition or differences across OMV quartiles, covariate-adjusted modeling identified a selective association with the proliferating alveolar macrophage compartment. We next tested whether individual microbial genera were associated with airway cell-type composition. Each CLR-transformed cell-type fraction was modeled against CLR-transformed genus abundance, adjusting for smoking, age, sex, BMI and OMV z-score, with disease dependence assessed through genus × DualStatus interactions. After correction, only one main-effect association and three DualStatus-dependent associations reached an FDR threshold of 10%, several involving unclassified genera. We therefore found little evidence for widespread or disease-dependent genus-cell associations and did not pursue these sparse signals further. Consistent with the primary BAL/MuSiC analyses, subsequent prediction and covariate-adjusted models provided no evidence for broad disease- or OMV-dependent restructuring of inferred airway cellular composition after correction for multiple testing (Supplementary Figs. S27–S30). These analyses supported the absence of widespread differences in major immune and epithelial cell proportions across HIV/COPD groups, while retaining the selective adjusted association between OMV burden and proliferating alveolar macrophages described above. Canonical correlation analysis further demonstrated only weak multivariate covariance between inferred cell-type composition and the evaluated clinical variables (Supplementary Fig. S31). Overall, these analyses indicate that major BAL/MuSiC-inferred airway cell populations were not broadly altered according to HIV/COPD status or OMV strata. Nevertheless, covariate-adjusted analysis identified a selective positive association between OMV burden and proliferating alveolar macrophages. The larger HLCA-based analysis resolved modest disease-associated differences in selected cell populations and global composition, demonstrating that fine-grained deconvolution estimates are sensitive to reference choice. Together, these findings support selective cellular associations rather than wholesale restructuring of airway cellular composition and indicate that the broader disease- and microbiome-associated transcriptional programmes observed in induced sputum are unlikely to be explained solely by shifts in the relative abundance of major airway cell populations.

## Discussion

In this longitudinal cohort of rural Ugandan adults with and without HIV and COPD, we integrate multi-omics profiling to investigate relationships between airway microbiome dynamics, bacterial OMVs, and immune-transcriptional programmes during lung-function decline. Although HIV-positive participants exhibit modest unadjusted reductions in FEV₁/FVC, HIV and COPD status does not independently explain lung-function variation after adjustment for age, BMI, sex, and smoking. This is consistent with the multifactorial determinants of chronic airflow limitation, including host genetics, prior tuberculosis, biomass smoke exposure, environmental factors, and healthcare access^12,21–23^. The predominantly virologically suppressed HIV-positive cohort may also have attenuated HIV-associated inflammatory effects, as effective antiretroviral therapy partially restores mucosal immunity and reduces systemic inflammation^24^. However, because our study did not include untreated HIV-positive individuals, pre-ART lung-function measurements, or longitudinal assessment of immune reconstitution, we could not determine whether immune recovery influences subsequent lung-function trajectories.

COPD in low- and middle-income countries frequently arises from non-smoking exposures, including biomass smoke and chronic respiratory infections, and may follow a slower or more heterogeneous course than smoking-related disease^4,7,8,25^. Consequently, microbial perturbations and associated immune remodeling may precede measurable spirometric decline within the 2-year follow-up period. This may also explain why our findings differ from studies of HIV-associated lung-function decline conducted in populations with higher smoking prevalence, injection drug use, or uncontrolled viremia^12,26–28^. Consistent with our previous analysis of this cohort, prior tuberculosis, rather than HIV infection, remained the strongest predictor of COPD^29^. Together, these observations suggest that early airflow limitation in this rural Ugandan population is shaped by a combination of environmental exposures, prior tuberculosis, and host immune adaptation rather than HIV or COPD status alone.

Across complementary microbiome analyses, including PERMANOVA, mixed-effects modeling, and baseline-delta regression, time rather than HIV or COPD status was the principal determinant of longitudinal community composition. Approximately 5% of microbiome variation was attributable to time, whereas HIV and COPD explained only small, non-reproducible fractions. These findings contrast with reports of widespread HIV- or COPD-associated dysbiosis^11,30,31^ and suggest that, in predominantly virologically suppressed individuals, airway microbial communities are shaped primarily by longitudinal ecological turnover rather than persistent disease status^12,30,32^. Longitudinal microbial change was also evident in COPD⁻/HIV⁻ controls, who exhibited a significant decline in Shannon diversity between visits. This decline persisted after adjustment for visit-associated compositional drift, supporting a biological rather than purely technical origin. Age was independently associated with higher baseline diversity, consistent with previous observations in older adults^33^. The accompanying increase in *Actinomyces* and *Atopobium* and decline in *Prevotella, Haemophilus, and Veillonella* closely resemble reported age-related changes in the oral-respiratory microbiome^33^ rather than patterns expected from reagent contamination^34^. Importantly, these longitudinal changes were comparable across all four clinical groups, consistent with a shared temporal process operating independently of HIV or COPD. These findings provide a longitudinal description of airway microbiome dynamics in older adults from sub-Saharan Africa.

Yet the composition of ecological instability, rather than its magnitude, carried biological significance. *Haemophilus* selectively expanded in unstable microbiomes of HIV+/COPD+ participants, whereas Sphingomonas was enriched in stable microbiomes of HIV-/COPD- controls. Although neither microbiome stability tertiles nor continuous Bray–Curtis shift magnitude predicted 2-year changes in FEV₁, FVC, or FEV₁/FVC, these taxon-specific associations indicate that well-controlled HIV and COPD modify the ecological behavior of selected bacterial taxa without broadly restructuring the airway microbiome. Because microbiome stability was defined using cohort-specific tertiles rather than an absolute biological threshold, instability here should not be equated with a clinical state of dysbiosis; these findings should be interpreted as relative ecological states rather than absolute measures of dysbiosis. Together with the immune and transcriptomic analyses, these observations support a model in which HIV-associated COPD is characterized by selective host–microbe interaction modules rather than generalized microbial dysbiosis, with disease modifying host responses to specific bacterial taxa rather than global community diversity.

Despite detectable temporal shifts in microbiome composition, these changes did not predict lung function decline. Previous studies identified declining microbial diversity or shifts in keystone taxa as predictors of COPD progression^35–37^, yet these associations were absent here. Several factors likely account for this divergence. First, most participants had sustained viral suppression, whereas prior studies included individuals with uncontrolled HIV, advanced immunosuppression, and higher microbial disruption. Second, host-specific factors, such as immune tone, antibiotic exposure, and epithelial barrier integrity, may overshadow disease-group effects, obscuring population-level associations^31^. Third, a 2-year sampling interval may be insufficient to detect meaningful physiologic decline in a relatively stable cohort. Finally, compositional shifts may not reflect functional alterations if microbial metabolic redundancy preserves airway ecological function; metagenomic and metabolomic analyses will be necessary to evaluate this possibility. Together, these findings suggest that in well-controlled HIV, temporal microbiome change does not translate into short-term pulmonary decline and may be buffered by host or microbial resilience mechanisms.

OMV concentrations were not associated with microbial alpha or beta diversity and showed no genus-level associations, indicating that bulk sputum OMV abundance is a poor surrogate for overall microbial community composition. This is consistent with OMV biology, as vesicle production reflects bacterial metabolic activity, stress responses, quorum sensing, and host–microbe interactions rather than bacterial abundance alone^38,39^. Moreover, OMV production differs markedly between taxa, with Gram-negative bacteria, such as *Haemophilus, Pseudomonas*, and *Neisseria,* generally producing substantially more OMVs than Gram-positive organisms^40,41^. Host factors, including immune activation, antibiotic exposure, oxidative stress, and nutrient availability, also influence vesicle production and may contribute to the observed association between OMV burden and proliferating alveolar macrophages^20^. Technical factors may further weaken compositional associations because 16S rRNA sequencing lacks strain-level resolution, whereas OMV assays capture vesicles released by metabolically active, including low-abundance, organisms. Together, these findings suggest that bulk OMV abundance reflects functional microbial activity rather than community composition. Future studies integrating strain-resolved metagenomics with targeted vesicle, metabolomic, proteomic, and cargo analyses will be required to define the contribution of specific bacterial OMVs to airway immune regulation.

Integration of cytokine networks, CyTOF immune phenotyping, and airway transcriptomics revealed distinct immunologic correlates of airway inflammation. HIV-positive individuals exhibited reduced IL-12p40 and IL-2, consistent with lower mucosal immune activation in ART-treated HIV^42^. Healthy participants exhibited higher IL-3 and IL-2, markers of preserved T-cell homeostasis^43^. After adjustment, HIV/COPD comorbidity did not show clear cytokine signatures, consistent with immune exhaustion or compensatory regulation^12,44^. *Corynebacterium* showed broad negative cytokine associations, consistent with its commensal, immunomodulatory role^45,46^, while *Neisseria* was negatively associated with IL-12p40, congruent with its capacity to dampen Th1 responses^47–49^. PBMC profiling identified microbe-immune links across T-cell and innate-cell clusters, including Th2-like cells with *Prevotella 7*, effector-memory T cells with *Arylsiella*, and NK and monocyte subsets with *Patescibacteria and Firmicutes*. These associations persisted after deconfounding, suggesting that airway microbiota exert modular, selective tuning of systemic immune architecture.

Transcriptomic profiling provided evidence of disease-associated host remodeling. Across induced sputum, bronchoalveolar lavage (BAL), and peripheral blood mononuclear cells (PBMCs), HIV was associated with a dominant mucosal transcriptional programme enriched for antigen presentation, lysosomal biology, interferon signaling, and chemokine responses. These associations remained after adjustment for lung function and demographic factors, whereas COPD contributed a comparatively smaller transcriptional signature. The pronounced compartmental gradient (induced sputum > BAL > PBMC) indicates that, despite effective antiretroviral therapy, HIV-associated immune remodeling remains concentrated at the airway mucosal surface rather than the systemic circulation. Integration of transcriptomic and microbiome data identified selective host–microbe transcriptional modules rather than widespread microbiome-gene associations. *Treponema, Alloprevotella, and Peptostreptococcus* aligned with neutrophil and myeloid inflammatory programmes, whereas *Actinomyces, Rothia, and Patescibacteria* were associated with chemokine, lysosomal, stress-response, and autophagy pathways. Many of these relationships were non-linear, consistent with threshold-like host responses to specific bacterial taxa rather than proportional effects of microbial abundance. Subset analyses further showed that HIV status was not independently associated with broad differences in peripheral or airway immune-cell composition after multi-confounder adjustment, consistent with the predominantly ART-treated, virologically suppressed cohort. In contrast, a substantial HIV-associated transcriptional signal persisted in induced sputum among COPD-positive participants, with 452 genes remaining significantly associated with HIV after multi-confounder correction, 97% of which were classified as deconfounded. The absence of comparable signals in BAL and PBMCs is most likely explained by the reduced statistical power of the subset analyses rather than a definitive absence of biological effects. Together, these findings suggest that, in well-controlled HIV infection, persistent disease-associated immune remodeling is more readily detected at the transcriptional level than through broad changes in airway or circulating immune-cell composition.

Collectively, these findings support a model of HIV-associated COPD under ART in which the airway microbiome remains temporally dynamic yet broadly stable at the community level, while disease-associated changes are concentrated within airway immune and epithelial transcriptional programmes. Rather than generalized microbial dysbiosis, a limited number of bacterial taxa consistently aligned with coordinated inflammatory, immune, and epithelial responses, identifying reproducible host–microbe interaction modules. Bulk OMV abundance was not associated with overall microbial community structure but showed limited associations consistent with functional microbial activity rather than community composition. Overall, these findings suggest that HIV-associated COPD in ART-treated individuals is characterized primarily by localized immune-transcriptional remodeling accompanied by selective host–microbe interactions, rather than widespread restructuring of the airway microbiome. This framework provides a foundation for future mechanistic studies and may help inform microbiome-guided biomarkers and precision immunomodulatory strategies in HIV-endemic low- and middle-income settings. Because this cohort consisted predominantly of ART-treated, virologically suppressed individuals, the observed immune and microbial profiles likely reflect the combined effects of long-term antiretroviral therapy, immune reconstitution, and persistent low-grade immune dysregulation, and should not be assumed to represent untreated HIV infection.

### Limitations of the study

This study has several limitations. First, although the longitudinal multi-omics design enabled comprehensive characterization of host–microbe relationships, its observational nature precludes causal inference. Associations among microbial taxa, OMV burden, immune phenotypes and transcriptional programmes should therefore be considered hypothesis-generating and require experimental validation to establish directionality, necessity and sufficiency. Second, bacterial profiling relied on 16S rRNA gene sequencing, which provides limited strain-level and functional resolution. Although well suited to low-biomass respiratory specimens, such as induced sputum, future host-DNA-depleted metagenomic, metatranscriptomic, metabolomic, and targeted microbial-vesicle analyses will be important for defining the organisms, microbial products, and molecular mechanisms underlying the interaction networks identified here. Third, induced sputum represents a mixed airway compartment and may not fully capture the spatial and cellular heterogeneity of the lower respiratory tract. We addressed cellular heterogeneity using reference-guided deconvolution with a BAL snRNA-seq reference derived from participants in the same parent cohort and an independent HLCA reference. However, the BAL reference comprised only four donors and 5205 nuclei, and deconvolution-derived proportions represent model-based estimates rather than direct measurements of cell abundance. Moreover, the BAL/MuSiC and HLCA frameworks differed in reference tissue, cellular taxonomy, reference construction, and sample coverage, and consequently did not reproduce all disease-associated differences in inferred cell proportions. We therefore interpret fine-grained cell-proportion estimates cautiously and use the HLCA analysis as an external reference-sensitivity analysis rather than evidence of absolute concordance between deconvolution approaches. Importantly, downstream OMV- and microbiome-cell analyses were based on the BAL/MuSiC framework because it provided a biologically relevant, cohort-derived airway reference and explicitly incorporated between-donor variation. Fourth, several high-dimensional analyses, including BAL snRNA-seq and cytokine profiling, were performed in modest sample subsets, limiting statistical power for subgroup and interaction analyses. The absence of significant differences should therefore not be interpreted as evidence of equivalence, particularly for low-abundance cell populations. Likewise, the selective association between OMV burden and proliferating alveolar macrophages identified after covariate adjustment requires replication in larger cohorts and orthogonal validation using direct cellular measurements. An independent dataset containing matched bulk RNA sequencing and single-cell or cytometric measurements would provide a stronger validation of inferred cell proportions. Finally, because the cohort consisted predominantly of ART-treated, virologically suppressed adults from rural Uganda, the findings may not fully generalize to untreated or viremic HIV infection or to populations with different genetic, environmental, microbial, and healthcare exposures. Also, the available combined data on CyTOF and viral load do not permit a sufficiently powered direct comparison of peripheral immune profiles according to virological suppression status. Despite these limitations, the integration of longitudinal microbiome profiling with immune phenotyping, transcriptomics and complementary deconvolution frameworks provides a systems-level approach for investigating host–microbe interactions in HIV-associated COPD and establishes testable hypotheses for subsequent mechanistic and validation studies.

## Methods

### Ethics approval and consent to participate

This research was conducted in accordance with all relevant ethical regulations for research involving human participants. The study protocols were approved by the Mulago Hospital Research and Ethics Committee (MHREC), Kampala, Uganda (Nos. 1996 and 2152), and the Uganda National Council for Science and Technology (UNCST), Kampala, Uganda (Nos. HS2375 and HS2035ES). Written informed consent was obtained from all participants before participation in the study. Participants were compensated for their time participating in the study and reimbursed for transportation associated with study visits.

### Study design and participants

#### Study design

We designed a longitudinal study with a baseline and a 2-year follow-up in rural Uganda, conducted between February 2018 and January 2023. At baseline (from February 2018 to February 2021), we randomly recruited participants from two large, independent cohorts, namely LINK (Lung function In Kampala and Nakaseke) and HiLiNK (HIV in Lung function In Kampala and Nakaseke), in the same geographic location^50^. COPD screening was performed in both cohorts. In the rural Nakaseke communities, 656 HIV-negative individuals were screened, while 722 HIV-infected individuals from four HIV treatment centers within the Nakaseke district were screened. We randomly selected 50 HIV-positive individuals with COPD, 50 HIV-positive individuals without COPD, 50 HIV-negative individuals with COPD, and 50 HIV-negative individuals without COPD (total *n* = 200). Participant sex was recorded as male or female and was included as a matching variable and covariate in relevant analyses. All participants were aged ≥35 years at enrolment. The number, sex distribution and age distribution of participants contributing to each study group and assay are reported in Tables 1–4 and, where applicable, in the corresponding figure and Supplementary Fig. legends. Participants were eligible for inclusion if they resided within the Nakaseke district, were ≥35 years of age, had confirmed HIV serostatus and spirometry-based COPD status at enrollment, were capable of understanding study procedures, underwent successful sputum induction, and did not have contraindications for spirometry and sputum induction procedures. We excluded participants with grade C/D spirometry curves, a history of antibiotic use within 2 weeks before enrollment, asthma, or other respiratory diseases, as well as pregnant women. Matching was performed based on three participant characteristics (age, sex, and smoking status). However, it was limited by a small sampling frame of *n* = 50 individuals in the COPD+/HIV- group from the LiNK cohort, a small sampling frame of *n* = 50 individuals in the COPD+/HIV+ group from the HiLiNK cohort, and differences in sociodemographic and clinical characteristics between the LiNK and HiLiNK cohorts. To control for these matching limitations, we determined differences in the distribution of sociodemographic characteristics across groups (COPD+/HIV+, COPD-/HIV+, COPD+/HIV-, COPD-/HIV-). We employed post hoc testing to examine the role of these covariates using a confounder-aware statistical tool in all downstream analyses. During the follow-up period (April 2022 to January 2023), we successfully reviewed 160 participants (40 COPD+/HIV+, 40 COPD-/HIV+, 40 COPD+/HIV-, and 40 COPD-/HIV-) who underwent successful sputum induction and peripheral blood collection. A subset of these (11 COPD+/HIV+; 19 COPD+/HIV-; 17 COPD-/HIV+; and 14 COPD-/HIV-) underwent successful bronchoscopy and bronchoalveolar lavage for downstream analysis.

#### Sex and gender considerations

Sex was self-reported by participants as male or female. Sex was considered in the study design through matching of participants by sex and was included as a covariate in relevant multivariable analyses. We did not collect gender identity as a separate variable; therefore, analyses according to gender were not performed.

### Definition of COPD

We defined COPD as a post-bronchodilator (post-BD) FEV_1_/FVC ratio lower than the fifth percentile of the NHANES III African American reference population and used post-bronchodilator FEV1 values to define the severity of disease^29,51^.

### Study procedures

#### Spirometry

Participants were screened for COPD using spirometry. Spirometry was performed by trained research assistants using Easy On-PC spirometer (ndd Medical Technologies, Zurich, Switzerland) before and 15–20 min after administration of 400 mcg of salbutamol using a spacer, with predicted values generated using the NHANES III reference equations embedded within the device software^29^. The quality of spirometry test results was assessed by American Thoracic and European Respiratory Societies (ATS/ERS) guidelines. In this study, grades A and B were considered the highest-quality spirometry curves obtained according to the ATS/ERS guidelines. On the contrary, grade C/D curves were considered low-quality and excluded.

#### HIV testing and HIV-related characteristics

At recruitment, participants were retested for HIV according to ART clinic guidelines in Uganda. HIV-related medical history, including nadir CD4^+^ T cell count, viral load, Septrin use, ART drug use, and history of opportunistic infections or pulmonary tuberculosis, was recorded from patient medical records, including sociodemographic and other clinical characteristics.

#### Sputum induction and sample collection

Before the sputum induction procedure, the following three-step cleansing routine using sterile water was followed: rinsing the mouth (gargling and discarding) three times, clearing the back of the throat, then discarding, and blowing the nose to minimize contamination with saliva or postnasal drip. Each sputum sample was assessed for mucoid consistency during deep coughing and expectoration, and a Gram stain was performed for quality control. Sputum samples with less than ten squamous epithelial cells per low-power field (×10) microscopy (indicative of a lower airway sample) passed the quality control check. Otherwise, the sample was rejected, and the induction procedure was repeated. Each participant was instructed to inhale and exhale 3% nebulized hypertonic saline through the mouth with the nose clipped. Three 5-min nebulizing periods were used during the collection, with a 2-min rest period in between. During each rest, spirometry was repeated to determine the percentage fall in FEV_1_. Fifteen (15 min) post-nebulization, 2–4 mL of expectorates were collected in sterile sputum containers. For all successful inductions, after a quality check, a sputum sample was collected in a sputum DNA collection, preservation, and isolation kit (Norgen Biotek Corporation, Canada; catalog no RU46100) as per the manufacturer’s instructions for downstream 16S rRNA gene sequencing or a sterile sputum container for downstream OMV extraction.

#### Broncho-alveolar lavage collection

Elective bronchoscopy and bronchoalveolar lavage were successfully performed on 61 study participants (11 COPD+/HIV+; 19 COPD+/HIV-; 17 COPD-/HIV+; and 14 COPD-/HIV-) via the nasal route using single-use fiberoptic AMBUscopes following a standardized protocol. Nebulized lignocaine was used for nasopharyngeal anesthesia, while 3–5 sprays of 2% lignocaine were used for oropharyngeal and tracheal anesthesia during the bronchoscopy procedure. Briefly, we nebulized with 10 mls of 2% lignocaine until the drug was completed. We then used the “spray-as-you-go” technique for topical anesthesia of the vocal cords and trachea via the bronchoscope’s working channel, administering 1% lignocaine and ensuring the total dose was kept to the lowest possible level, with an upper limit of 7.0 mg/kg body weight. In all participants, BAL was performed from the right middle lobe (RML B4/5). At least 100 ml of normal saline was instilled during BAL, not exceeding 200 ml. The required amount of fluid was instilled in 2–5 aliquots, using smaller aliquots in COPD patients. BAL fluid was retrieved by application of a negative pressure manually using the hand-held syringe, providing a significantly higher percentage of BAL fluid return compared to wall suction, without affecting diagnostic yield. For safety considerations, we aimed for at least 70% fluid recovery.

#### Sample collection and processing

We collected about 100–160 mL of BAL in sterile 50 mL centrifuge tubes at room temperature following a successful bronchoscopy procedure, transferring it into three or four 50 mL conical tubes, and transported the sample immediately to the central processing laboratory at Makerere University School of Biomedical Sciences for processing. Briefly, in the laboratory, we spun the sample for 10 min at 800 × *g* at room temperature. We then harvested the supernatant, transferred it into 10 ml centrifuge tubes, and stored it at −20 °C for downstream applications or for storage at −20 °C. The cell pellet was resuspended in 1 ml of D10 (DMEM with 10% FBS and 5 μg of L-glutamate), ready for counting, downstream application, or storage at −80 °C (90% FBS, 10% DMSO).

#### Sputum processing

Using follow-up sputum samples designated for OMV extraction, they were processed as follows: upon receipt in a Biosafety level-3 laboratory under the cold chain, we added an equal volume of phosphate-buffered saline (PBS). The suspension was centrifuged at room temperature for 10 min at 160 × *g*. The sputum supernatant was harvested for downstream application. The remaining sputum cell pellet was counted and viability assessed using Trypan blue before adding freeze media, transferring to the −80 °C freezer in a Nalgene cryopreservation container (Mr. Frosty), and finally into liquid Nitrogen after 24 h. We used a 2 mL cryovial to store the harvested supernatant, which was then placed in the −80 °C freezer for downstream or validation assays. The remaining volume was used for OMV extraction as follows.

#### OMV extraction and LPS ELISA

OMVs were extracted from sputum supernatants using the Norgen Exosome extraction kit (catalog no. 65400, Norgen Biotek Corporation, Ontario, Canada), following the manufacturer’s instructions. Norgen’s Exosome extraction kit allows for the purification of exosomes from 500 µl to 2 ml sputum samples. The exosome extraction kit contained Slurry E, ExoC buffer, ExoR buffer, and Mini Filter Spin Columns, which were inserted into 2 mL tubes. To 3 mL of cell-free diluted sputum supernatant, we added 6 µL of ExoC Buffer, followed by 200 µL of Slurry E. The mixture was vortexed for 10 s and left to stand at room temperature for 10 min. The mixture was centrifuged for 2 min at 450 × *g*, and the resultant supernatant was discarded. Two hundred microliters of ExoR Buffer were added to the slurry pellet, and the mixture was vortexed for 10 s. The slurry pellet was resuspended in 200 µL ExoR Buffer and incubated at room temperature for 10 min. After incubation, the mixture was centrifuged for 2 min at 100 × *g*. The supernatant was then transferred to a Mini Filter Spin assembled with a 2 mL tube and centrifuged for 1 min at 4000 × *g*. The purified exosomes were then harvested in the flow-through. We performed a competitive LPS ELISA as a proxy measure for OMV concentration. We allowed all reagents and specimens to equilibrate to room temperature to perform the ELISA. We then documented the ELISA plate configuration and prepared our standards and wash buffer. We vortexed our samples and diluted them with PBS in a 1:1 ratio to make 50 µl of each mix. We then pipetted 50 µl of our standards and diluted participant samples into the appropriately labeled wells and mixed them with a microplate shaker. We added 50 µl of the detection reagent into each well, sealed the plate with adhesive plate films, and incubated it at 37 °C for 60 min. After 60 min of incubation, we removed the self-adhesive sealing film and aspirated the contents of the wells. We inverted the plate over a paper towel and firmly tapped it several times to remove the residual contents. We then washed the plate three times with the prepared wash buffer. We pipetted 100 µl of HRP conjugate into each well, sealed the plate with adhesive plate films, and incubated it at 37 °C for 30 min. After 30 min of incubation, we removed the self-adhesive sealing film, decanted the contents of the wells, and then washed the plate five times. We then added 90 µl of TMB to each well in the same order as the samples and incubated the plate for 20 min. We then added 50 µl of the stop solution in the same order as the sample was added and then read the plate at a wavelength of 450 nm.

16S rRNA gene sequencing. DNA was extracted from samples using a sputum DNA collection, preservation, and isolation kit (Norgen Biotek Corporation, Canada; catalog no. RU46100). DNA purity and concentration were determined using a spectrophotometer and a Qubit fluorescent-based kit. V3-V4 hypervariable region of the 16S rRNA gene was PCR-amplified using 16S rRNA-specific 341F (5′ NNNNNNNNNNCCTACGGGNGGCWGCAG) and 785R (5′ NNNNNNNNNNGACTACHVGGGTATCTAAKCC) primers. All samples were tagged with unique ten-nucleotide sequences (“barcodes”) incorporated into the forward primer. The PCRs included about 1–10 ng of DNA extract (total volume, 1 μL), 15 pmol of each forward primer, and reverse primer in 20 μL of 1× MyTaq buffer containing 1.5 units MyTaq DNA polymerase (Bioline GmbH, Luckenwalde, Germany), and 2 μL of BioStabII PCR Enhancer (Sigma-Aldrich Co). PCRs were carried out for 30 cycles. DNA concentration of amplicons of interest was assessed by gel electrophoresis. About 20 ng of amplicon DNA from each sample was pooled for up to 48 samples carrying different barcodes. The amplicon pools were purified with one volume of Agencourt AMPure XP beads (Beckman Coulter, Inc., IN, USA) to remove primer dimer and other small mispriming products, followed by an additional purification on MinElute columns (Qiagen GmbH, Hilden, Germany). About 100 ng of each purified amplicon pool DNA was used to construct Illumina libraries using the Ovation Rapid DR multiplex system 1–96 (NuGEN Technologies, Inc., CA, USA). Illumina libraries (Illumina, Inc., CA, USA) were pooled and size-selected by preparative gel electrophoresis. Bacterial 16S rRNA amplicons were sequenced targeting the V3-V4 (300-bp read length, paired-end protocol) region using the Illumina Miseq platform. The raw sequences were processed to remove potential human contamination through sequence processing and OTU classification. The human genome was masked with the ProGenomes2 microbial genome database. Raw reads were mapped to the human genome, discarded if they had 95% identity, masked, and filtered. Finally, we validated the human reads found by filtering out potential “human” contamination and aligned these against the NCBI nucleotide (nt) database, resulting in only the top human hits. After removing human contamination, the remaining raw reads were processed using LotuS (1.62). Poisson binomial model-based read filtering was applied. OTU clustering (UPARSE) was based on sequence similarity of 97%, while SILVA was incrementally used as a database for a taxonomic assignment using a Lambda taxonomic similarity search. The taxonomic classification (genus thresholded at 95% identity) was parsed using a custom Perl script, such that unassigned taxonomic levels were assigned to the last known taxonomic level and sequentially numbered. Normalization and computation of alpha diversity measures were performed using the rarefaction tool kit (RTK 0.93.1) with default settings. Sputum samples from Visit 1 (Baseline: February 2018– February 2021) and Visit 2 (Follow-up: April 2022–January 2023) were sequenced in two separate runs (NGS1969 in 2020 and NGS3581 in 2023, respectively) using identical laboratory protocols. Raw reads from both runs were pooled and rarefied together to 8278 reads per sample to standardize sequencing effort across participants, with prevalence and abundance filtering thresholds applied to the combined dataset, ensuring a common rarefaction depth and filtering criteria across both runs (Supplementary Table 12).

#### Quality control for 16S rRNA amplicons

All participants underwent sputum induction following a standard protocol to reduce sample variability. To avoid sample contamination, we used a single kit (Norgen Biotek sputum DNA isolation and preservation kit) that eliminated multiple steps of sputum processing, allowed transportation at room temperature, and inactivated microbial growth in the sample, thereby preserving microbial composition. During sample collection, stringent quality control measures were implemented to reduce contamination from saliva and postnasal drip. We also included negative controls (sputum kit with sterile water and buffer) during sample collection, DNA extraction, PCR amplification, and sequencing. Negative controls were negative for V3-V4 amplicons at PCR; no sequences were generated.

#### Sample size calculation

The sample size was estimated based on taxonomic profiles derived from Human Microbiome Project (HMP) data^52^. A conservative approximation of our power to detect microbiome associations at the genus level between groups was tested using a t-test. Given *n* = 50 individuals per group, assuming an effect size of 0.8 (Cohen’s *d*) and an alpha threshold of 0.05/30, reflecting a Bonferroni correction for the 30 main airway bacterial genera, power was approximately 80% to conclude significance for each accurate microbial biomarker. Clinically relevant microbial feature abundance effects that fall within the convention of a large effect size Cohen’s *d* ≥ 0.8 commonly used to define clinically relevant microbial abundance changes.

#### Profiling sputum cytokines using Luminex

We thawed induced sputum supernatants collected from participants and stored at −80 °C. Analytes were quantified in pg/mL using the BioRad human cytokine screening 48-plex kit (Catalog no. 12007283) following the manufacturer’s instructions on an LX-200 using Luminex xPONENT software. Analytes were quantified using the Bio-Rad Human Cytokine Screening 48-Plex Kit (Bio-Rad Laboratories, Cat. No. 12007283) according to the manufacturer’s instructions. Induced-sputum supernatants were diluted 1:4 before incubation with the kit-supplied antibody-coated magnetic beads, followed by the supplied secondary detection antibodies and streptavidin-PE. Antibody reagents were used at the manufacturer’s specified concentrations. Details of the assay reagents are provided in Supplementary Table 12. The assay involved incubating 1:4 diluted induced sputum supernatant with antibody-coated magnetic beads, automated magnetic plate washing, incubating the beads with secondary detection antibodies, and adding streptavidin-PE. Standard curves of known protein concentrations were used to quantify analytes. Samples below the detection limit were assigned the lowest detectable value, adjusted for a 1:4 dilution factor. Similarly, samples above the detection limit were assigned the maximum detectable value, adjusted for a 1:4 dilution factor. *Luminex analysis*. Using the Mann–Whitney *U* test, we compared cytokine concentrations between pairs within each of the four groups stratified by COPD/HIV dual status. We summarize results using box plots. Significant comparisons with *P* < 0.05, depicted with *, are shown. We further performed Spearman’s correlation analysis between airway pro-inflammatory cytokines, bacterial-derived OMVs, and Lung function parameters. Statistically significant correlations with adjusted *P* < 0.05 are depicted with *.

#### CyTOF staining and measurement

To conduct a comprehensive immune profiling of a Ugandan COPD cohort, we obtained paired induced sputum and peripheral blood samples from a subset of the cohort for Cytometry by Time-of-Flight (CyTOF). This subcohort comprised 26 COPD+/HIV- and 23 COPD+HIV+ cases, with 20 COPD-/HIV- and 21 COPD-/HIV+ as respective controls. Due to the limited number of viable cells in the induced sputum pellet, we obtained only PBMC samples during the assay. PBMCs were isolated from peripheral blood using density-gradient centrifugation, followed by freezing in 90% FBS/10% DMSO (freeze media). For CyTOF staining, the Maxpar Direct Immune Profiling Assay (MDIPA, Cat. No. 201325) from Standard BioTools (San Francisco, California) was used and performed according to the manufacturer’s instructions. The complete antibody panel, including antibody target, supplier, catalogue number, clone, metal conjugate, amount/dilution, and lot number, is provided in Supplementary Table 15. CD45 metal-conjugated antibodies (clone HI30; Standard BioTools) were used for sample barcoding as detailed in Supplementary Table 12. Frozen PBMCs were thawed in prewarmed RPMI, supplemented with 10% FBS, washed twice, and incubated with 25 units/mL benzonase for 20 min at 37 °C. Cells were then washed with Maxpar cell staining buffer, and a blocking step was performed by incubating the cells with Human TruStain FcX (BioLegend) for 10 min at RT. Afterward, samples were barcoded by incubating each with a different mixture of three of six CD45-metal conjugated isotopes for 30 min on ice (Supplementary Tables 13–15). After barcoding, samples were washed twice with Maxpar cell staining buffer and pooled afterward. Pooled cells were washed again with Maxpar cell staining buffer and then incubated with the antibody mix for cell-surface staining provided by the Maxpar Direct Immune Profiling Assay Kit for 30 min at RT. After incubation, cells were washed twice with Maxpar cell staining buffer. Cells were subsequently incubated overnight in 2% methanol-free formaldehyde solution (ThermoFisher Scientific). After fixation, cells were washed twice with Maxpar cell staining buffer and were incubated in Iridium intercalator solution (Standard BioTools) for 60 min. Cells were then washed twice with Maxpar cell staining buffer and kept on ice until CyTOF analysis. Data acquisition was performed on a CyTOF2 mass cytometer upgraded to Helios specifications (CyTOF2/Helios) (Standard BioTools). The instrument was tuned according to the manufacturer’s instructions. EQ Four Element Calibration Beads (Standard BioTools) were added to the sample to normalize signal intensity across the acquisition period.

#### Induced sputum RNA extraction and sequencing

Total RNA was extracted from 100 induced sputum samples (26 COPD+/HIV+; 23 COPD+/HIV-; 26 COPD-/HIV+ and 25 COPD-/HIV-) using the RNeasy Mini Kit (Qiagen, Catalogue no. 74104), following the manufacturer’s instructions. RNA quality was assessed using NanoDrop and Agilent Bioanalyzer 2100 platforms. We ensured an optimal RNA integrity number of 7 or above for high-quality downstream RNA sequencing. Extracted RNA samples were treated with DNase I to degrade any possible DNA contamination, followed by 3′ mRNA enrichment using the NEBNext Poly (A) mRNA Magnetic Isolation Module from the New England Biolabs (NEB, catalogue no.E7490) to generate direct RNA libraries following the manufacturer’s instructions. Following library fragmentation, end reparation, and 3′-end single nucleotide adenine addition, sequencing adapters were ligated to the fragments, followed by fragment enrichment by PCR amplification with 17 cycles. During the quality control (QC) step, Agilent 2100 Bioanalyzer and ABI StepOnePlus Real-Time PCR System were used to qualify and quantify the sample library. Fragments were sequenced from both ends, each 150 base pairs, using the Illumina NovaSeq 6000 system. We demultiplexed all libraries for each sequencing lane using Illumina bcl2fastq version 2.20. We allowed 1 or 2 mismatches or Ns in the barcode read when the barcode distances between all libraries on the lane allowed for it. We clipped sequence adapter remnants from all reads. Reads with a final length of less than 20 bases were discarded. We created FastQC reports for all FASTQ files. The median Phred score was at least 30 for our samples. After QC, clean reads were aligned to the human reference genome (GRCh38) using the STAR aligner (v2.7.9a) with default parameters. Gene-level counts were obtained using featureCounts (v2.0.1) with GENCODE v35 annotations (Supplementary Table 12).

#### BAL/PBMC RNA extraction and sequencing

Total RNA was successfully extracted from 61 BAL samples using the RNeasy Mini Kit (Qiagen, Catalogue no. 74104), following the manufacturer’s instructions. RNA quality was assessed using the NanoDrop and Agilent Bioanalyzer 2100 platforms, ensuring an RNA integrity number of 7 or higher for high-quality downstream RNA sequencing. Based on this criterion, we excluded six samples with a RIN value below 7, resulting in a final sequenced sample size of 55 (11 COPD+/HIV+; 18 COPD+/HIV-; 12 COPD-/HIV+; and 14 COPD-/HIV-). To allow for comparative analysis between BAL and blood, we extracted total RNA from a random subset of 24 PBMC samples (10 COPD+/HIV+; 7 COPD+/HIV-; 3 COPD-/HIV+; and 4 COPD-/HIV-), ensuring an RNA integrity number of 7 or higher for high-quality downstream RNA sequencing. Extracted RNA samples were treated with DNase I to degrade any possible DNA contamination, followed by 3′ mRNA enrichment using the NEBNext Poly (A) mRNA Magnetic Isolation Module from the New England Biolabs (NEB, catalogue no. E7490) to generate direct RNA libraries following the manufacturer’s instructions. Following library fragmentation, end reparation, and 3′-end single nucleotide adenine addition, sequencing adapters were ligated to the fragments, followed by fragment enrichment by PCR amplification with 17 cycles. During the quality control (QC) step, Agilent 2100 Bioanalyzer and ABI StepOnePlus Real-Time PCR System were used to qualify and quantify the sample library. Fragments were sequenced from both ends, each 150 base pairs, using the Illumina NovaSeq 6000 system. We demultiplexed all libraries for each sequencing lane using Illumina bcl2fastq version 2.20. We allowed 1 or 2 mismatches or Ns in the barcode read when the barcode distances between all libraries on the lane allowed for it. We clipped sequence adapter remnants from all reads. Reads with a final length of less than 20 bases were discarded. We created FastQC reports for all FASTQ files. The median Phred score was at least 30 for our samples. After QC, clean reads were aligned to the human reference genome (GRCh38) using the STAR aligner (v2.7.9a) with default parameters. Gene-level counts were obtained using featureCounts (v2.0.1) with GENCODE v35 annotations (Supplementary Tables 12 and 16).

### Statistical analysis

#### Missing data

Analyses were performed using participants with available data for the variables required for each analysis. No imputation of missing participant-level covariate, exposure, outcome, or follow-up data was performed. Consequently, analytical sample sizes varied across analyses according to data availability and are reported for the corresponding analyses, figures, and tables.

#### Lung function analysis

We evaluated whether changes in lung function from visit 1 (V1) to visit 2 (V2) differed by combined disease status (*DualStatus*: COPD-/HIV- [reference], COPD-/HIV+, COPD+/HIV-, COPD+/HIV+). Data preparation: lung function parameters included pre-bronchodilator best FEV₁ (L), pre-bronchodilator best FVC (L), post-bronchodilator best FEV₁ (L), post-bronchodilator best FVC (L), and post-bronchodilator FEV₁/FVC ratio. For each participant with paired measurements, the change (Δ) was calculated as V2-V1 for each parameter. Disease status and covariates (age, sex, BMI, and smoking status) were recorded from V1. Statistical analysis: separate linear models were fit for each lung function parameter with Δ as the outcome and *DualStatus* as the main predictor, adjusting for age, sex, BMI, and smoking status. The reference group was COPD-/HIV-. Results are reported as estimated differences in Δ between each disease group and the reference group, with 95% confidence intervals. *P*-values were adjusted for multiple testing across models using the Benjamini–Hochberg method.

#### 16S RNA sequencing data analysis (beta diversity analysis)

(Supplementary Table 16) Sample and data processing. Raw sequencing reads were rarefied to 8278 reads per sample to standardize sequencing effort across participants. Taxa were filtered based on two criteria: (1) prevalence threshold: a taxon had to be present in more than 10% of samples; and (2) abundance threshold: a taxon’s cumulative relative abundance across all samples had to exceed 0.0005% of the total read count. These filtering steps removed low-prevalence and low-abundance taxa to reduce noise and focus analyses on more prevalent community members. Participants were stratified into four disease status categories (*DualStatus*) based on their COPD/HIV diagnoses: COPD-/HIV-: healthy controls, COPD-/HIV+, COPD+/HIV- and COPD+/HIV+. *Bray–Curtis instability metric:* For participants with microbiome data from both visits, the Bray–Curtis distance between each follow-up sample and the individual’s own baseline profile (visit 1) was calculated at the OTU level. A higher Bray–Curtis value indicates greater microbiome instability relative to baseline. Linear modeling: To assess how much each predictor contributed to explaining microbiome instability, we fitted a linear model with Bray–Curtis distance to baseline (visit 1) as the outcome, and DualStatus (disease group), age, sex, BMI, and smoking status as predictors. From the model’s analysis of variance (ANOVA), we extracted the sum of squares for each predictor, divided by the total sum of squares, to obtain the percentage of variance explained. To estimate the average magnitude of microbiome change for each disease group while controlling for demographic factors, we calculated model-adjusted means from the linear model mentioned above using the emmeans package. Estimates were adjusted for age, sex, BMI, and smoking status, and are presented with 95% confidence intervals. *Stratification by microbiome stability*. Longitudinal microbiome instability was assessed only among participants with one valid OTU-level microbiome profile at both visit 1 and visit 2 (*n* = 101 participants; 202 microbiome samples). For each participant, we calculated the Bray–Curtis distance between their visit 1 and visit 2 OTU profiles; higher values indicate greater within-individual microbiome instability. Participants were ranked according to this distance and assigned to approximately equal-sized tertiles: stable (*n* = 34), medium (*n* = 34), and unstable (*n* = 33). The paired cohort comprised COPD-/HIV- (*n* = 26), COPD-/HIV+ (*n* = 28), COPD+/HIV- (*n* = 24), and COPD+/HIV+ (*n* = 23) participants. The numbers of participants within each stability × DualStatus subgroup are reported in Supplementary Table 17. We compared OTU- and genus-level relative abundances across stability groups using a three-step approach. Screening for differences: We applied Kruskal–Wallis tests at the OTU level to detect taxa with abundance differences between stability groups (FDR-adjusted *p*-values). Interaction testing: For the OTUs with the lowest *p*-values, we used Aligned Rank Transform (ART) models to test for stability-group × disease-status (DualStatus) interactions. This step identified OTUs for which the effect of stability on abundance varied across disease groups. Longitudinal taxon modeling: For genera with a significant *stability group × disease status (DualStatus)* interaction (FDR < 0.1) identified by the ART models, we performed post hoc pairwise comparisons using emmeans. Two sets of contrasts were extracted: Within-disease comparisons, comparing genera abundance between stability groups (stable, medium, and unstable) within each *DualStatus* category. Within-stability comparisons: comparing genus abundance between disease groups within each stability category. *P*-values were adjusted for multiple testing (Benjamini–Hochberg method). Effect estimates are expressed in ART rank units, with positive estimates indicating higher abundance in the first group of the contrast, and negative estimates indicating higher abundance in the second group. PERMANOVA: We evaluated changes in overall microbiome composition using Bray–Curtis dissimilarities computed from the rarefied, filtered OTU count table. Prior to distance calculation, samples with all zeros were removed, and remaining NAs were set to zero. Metadata were harmonized, and participants with paired measurements were retained. PERMANOVA models (adonis2, 999 permutations) were fit with participant ID used as a stratification factor (strata = ID) to respect within-subject pairing. We reported marginal (type I-like) sums of squares using by = margin to quantify the variance explained (*R*^2^) by each term, independent of the order of entry. Four models were specified: Model 1 (main effects): HIV status, COPD status, timepoint (visit), age, BMI, sex, and smoking. Model 2 (trajectory differences): full three-way interaction HIV × COPD × timepoint, plus age, BMI, sex, and smoking. Model 3 (binary disease): any disease (Dualdisease), timepoint, age, BMI, sex, smoking. Model 4 (age interaction): Dualdisease ×  age, plus timepoint, BMI, sex, smoking. For visualization, we converted *R*^2^ to percentages and summarized all terms across the four models in a grouped bar plot with significance annotations. We examined genus-level changes in relative abundance between visit 1 (V1) and visit 2 (V2) to identify taxa potentially influenced by microbiome instability, disease status, or other factors.

#### Genus level analysis: sample and data processing

We assessed within-participant changes in genus-level relative abundance between visit 1 (V1) and visit 2 (V2) using paired data from the rarefied and filtered genus-level relative abundance table, matched to participant metadata. Only participants with microbiome data available for both visits were included. Linear modeling (Unadjusted analysis): For descriptive purposes, we performed paired Wilcoxon signed-rank tests separately within each *DualStatus* category. For each genus, we calculated the log₂ fold change (V2 vs V1) and adjusted *p*-values using the Benjamini–Hochberg false discovery rate (FDR) procedure. Results were visualized as volcano plots stratified by *DualStatus*, with dashed thresholds indicating |log₂ fold change| > 1 and FDR < 0.1. Adjusted mixed-effects modeling: We next applied linear mixed-effects models (lmer) to account for covariates and repeated measures. Two model structures were fit for each genus. In the no-interaction model, we tested the average change from V1 to V2 across all participants: Fixed effects: visit (V1 vs V2), *DualStatus*, age, sex, BMI, smoking status. Random effects: subject ID to account for repeated measures. Genera with an FDR-adjusted *p* < 0.1 for the visit term (visit 2) were considered significant. In the Interaction model, we tested whether the change from V1 to V2 differed by *DualStatus*. Fixed effects included a visit × *DualStatus* interaction, plus the same covariates as above. The main effect for visit 2 represents the change in the reference group (COPD-/HIV-). Interaction terms (visit 2:DualStatusCOPD+/HIV+) represent differences in the time effect across disease groups relative to the reference group. Significant main or interaction effects were defined as FDR-adjusted *p* < 0.1. Baseline Abundance Predicting Change Over Time. We investigated whether the baseline abundance of each genus predicted its change over time (Δ = V2-V1), and whether this relationship varied by disease status (*DualStatus*). For each genus, we retained only participants with paired samples from both visits. Model setup: Baseline abundance (V1) was used as the main predictor of delta (Δ). Covariates included age, sex, BMI, and smoking status. Interaction term: V1 × *DualStatus* tested whether the slope of the baseline-delta relationship differed by disease group. Interpretation of coefficients: V1 term: slope of the baseline-delta relationship in the reference group (COPD-/HIV-). V1 × DualStatusX terms: difference in slope for a disease group compared to the reference. Negative slopes indicate regression to the mean (higher baseline abundance: larger decrease over time). Positive slopes indicate that higher baseline abundance predicted a greater increase (or smaller decrease). We compared a model without interaction to a model with interaction using likelihood ratio tests (LRTs). Genera with significant LRT results (FDR < 0.1) were further examined in the full interaction model.

#### Microbiome-host immune crosstalk analysis

To interrogate airway microbiome dynamics driving immune responses in a rural Ugandan HIV-COPD cohort, we conducted comprehensive immune profiling of a Ugandan HIV-COPD cohort using Luminex assay targeting 48 pro-inflammatory cytokines in induced sputum obtained from 8 COPD+/HIV+, 10 COPD+/HIV-, 10 COPD-/HIV+ and 9 COPD-/HIV- participants (*n* = 37) coupled with mass flow cytometry targeting 37 immune cell populations in peripheral blood obtained from 26 COPD+/HIV-, 23 COPD+/HIV+, 20 COPD-/HIV- and 21 COPD-/HIV+ participants (*n* = 90).

### CyTOF analysis

Resulting flow cytometry standard (FCS) files were normalized, and gating of single, live cells and CD45+ cells was performed using Cytobank (www.cytobank.org) and FlowJo. Then, files were downloaded and subsequently analyzed in *R* using the CATALYST and diffcyt packages. Briefly, samples were de-barcoded to obtain individual files, and normalization between different measurement batches was performed using the BatchAdjust function. Subsequently, cluster identification was performed using FlowSOM/ConsensusClusterPlus (used all defaults with max*K* = 80). For visualization, UMAP plots were generated. First, the main lineages in PBMCs were identified by performing clustering with 12 markers (CD16, CD56, TCRgd, CD66b, CD3, CD8a, CD11c, HLA-DR, CD14, CD4, CD19, and CD20). Subsequently, T and non-T cell clusters were merged, and subcluster analysis of T and non-T cells was performed. For the clustering of T cells, 16 markers (CD3, CD45RO, CD127, CD28, CD27, CCR7, CD4, CD8a, CD45RA, CD194, CD161, CXCR3, CCR6, TCRgd, CD25, and CXCR5) and for the clustering of non-T cells 15 markers (CD38, CD11c, HLA-DR, CD294, CD19, CD20, IgD, CD123, CD16, CD14, CD56, CD8a, CD66b, CD57, and CD27) were used. Clusters were identified based on the guidelines within the Approach to Bivariate Analysis of Data Acquired Using the Maxpar Direct Immune Profiling Assay by Standard BioTools. The statistical significance of cluster abundances was determined using diffcyt-DA-edgeR and a Benjamini-Hochberg adjustment with a false discovery rate threshold of 5%. (Supplementary Table 16).

#### Induced sputum RNA sequence data analysis

We subjected the raw count data matrix to a filtering step to remove lowly expressed genes. Specifically, genes were retained if they had a count of at least ten in at least five samples. Filtered count data were then used to create a DESeqDataSet object. The design formula incorporated key covariates, allowing the analysis to account for potential confounders. Specifically, the variables included in the model were dual disease status, z-scored OMV levels, experimental status, smoking status, z-scored age, and sex. This comprehensive approach ensured that the effect of HIV/COPD status was adjusted for the influence of sex, smoking status, and other covariates. To examine the differential gene expression, we computed the log2-fold changes in gene expression counts. Upon successfully filtering the log-transformed adjusted *p*-values greater than 0.1 and Log2 fold changes greater than one, top-upregulated gene signatures in the HIV+COPD+ group versus the HIV-COPD- group were identified. We further performed Gene Ontology (GO) enrichment analysis on the differentially expressed genes (DEGs) identified in the context of dual disease status and visualized the enriched GO terms, summarizing key biological processes (BP), cellular components (CC), and molecular functions. We also performed a KEGG pathway enrichment analysis to identify pathways predominant in our study population. Finally, to examine individual pro-inflammatory gene signatures induced during chronic airway infection, we extracted genes relevant to inflammation from a list of differentially expressed genes in the COPD+HIV+ versus COPD-HIV- groups and further assessed their significance of differential expression by adjusting *p*-values for multiple testing using the Benjamini–Hochberg (BH) method, controlling the false discovery rate. For gene-microbiome association analysis, we first filtered the microbiome data to remove rare and low-abundance taxa. We calculated the relative abundance of each genus across all samples and its prevalence (percentage of samples in which the genus was present). Genera were retained if they had a relative abundance >0.05% across all samples and were present in >10% of samples. This filtering reduced the number of genera from 255 to 52. To account for the compositional nature of microbiome data, we applied a centered log-ratio (CLR) transformation to the filtered genus abundance data. For the gene-expression analysis, we focused on a subset of 18 pro-inflammatory genes identified among the differentially expressed genes in the COPD+/HIV+ versus COPD-/HIV- comparison. Normalized gene-expression counts were used for the selected genes: *RLF, DNTTIP2, CSF1, IL1R1, CXCR4, REL, B3GNT5, CCL3, TNFAIP6, CXCL8, PLPP3, IRAK2, CXCL9, CCL4, CCL4L2, CCL3L3, HIVEP2*, and *CXCL2*. Univariate analysis was performed using metadeconfoundR (version 1.0.4) to test for associations between microbial abundances and genes and the influence of dual disease status. We controlled for potential confounders such as age, sex, and smoking status. Due to the non-normal distribution of the data, we employed nonparametric statistical tests. For discrete predictors, we used the Wilcoxon test for binary variables and the Kruskal–Wallis test for variables with more than two categories. For continuous variables, we used Spearman’s rank correlation test. All *p*-values were adjusted for multiple testing using the Benjamini–Hochberg method to control the FDR at 10%. Effect sizes were calculated using Cliff’s delta on the filtered count data for bacterial abundances. Statistical tests were performed on CLR-transformed data to account for compositionality, while effect sizes were calculated on the original count data to provide interpretable measures of association strength.

#### BAL/PBMC bulk RNAseq analysis

Gene counts were imported into R (v4.3.1) and analyzed using the DESeq2 package (v1.38.3). Lowly expressed genes (row sums <10) were filtered prior to normalization. Variance-stabilizing transformation (VST) was applied for principal component analysis (PCA). Differential expression testing was performed using the Wald test, applying Benjamini–Hochberg correction to control the false discovery rate (FDR). Genes with FDR-adjusted *p* < 0.05 and absolute log₂ fold change (log₂FC) > 1 were considered significantly differentially expressed. PCA was performed on variance-stabilized data. Ellipses representing 95% confidence intervals were derived from multivariate *t*-distribution modeling. For cross-compartment comparisons (BAL vs. blood, BAL vs. sputum), scatter density plots of log₂FC values were generated using kernel density estimation. Shared and unique transcripts were identified using normalized expression thresholds and visualized via Venn diagrams. Differentially expressed gene sets were subjected to Gene Ontology (GO) and Kyoto Encyclopedia of Genes and Genomes (KEGG) enrichment analyses using the clusterProfiler package (v4.6.2). Hypergeometric tests were employed, and enrichment results were corrected for multiple testing using the Benjamini–Hochberg method (FDR-adjusted *p* < 0.05). We visualized enriched GO terms, summarizing key biological processes (BP), cellular components (CC), and molecular functions. Data visualization and statistical analysis: Volcano plots were generated with EnhancedVolcano (v1.16.0). All plots were produced in *R* using ggplot2 (v3.4.2) and ComplexHeatmap (v2.16.0). All reported *P* values are FDR-adjusted unless otherwise specified.

#### BAL scRNAseq analysis

We performed single-cell RNA sequencing (scRNA-seq) of bronchoalveolar lavage (BAL) samples from four participants spanning a range of HIV and COPD clinical phenotypes (Table 4). BAL cells were subjected to standard single-cell RNA sequencing (scRNA-seq) protocols as described elsewhere. Raw sequencing data were processed using Scanpy and Seurat v4. Cells were retained if they expressed ≥200 genes, had ≥500 UMI counts, and <5% mitochondrial gene content. Genes starting with "MT-" were used to calculate mitochondrial gene percentages. A total of 5205 high-quality cells were retained after filtering. To mitigate batch effects and enable integrated analysis across participants, raw count matrices were normalized using SCTransform and integrated using the Harmony algorithm. Batch variables were defined by original sample identity (orig.ident). Dimensionality reduction was performed using principal component analysis (PCA), followed by UMAP projection and Leiden clustering. Harmony-corrected principal components were used to construct a shared nearest-neighbor graph for downstream analysis. Cell type annotation was performed using a multi-level classification model that generated coarse-to-fine-grained cell labels (predicted.ann_finest_level). Annotated populations included alveolar macrophages (proliferating and standard), monocyte-derived macrophages, classical/non-classical monocytes, CD4/CD8^+^ T cells, Dendritic cells (DC2), AT1 epithelial, multiciliated (non-nasal), interstitial macrophages (perivascular), and EC general capillary cells.

#### BAL-derived single-cell RNA-Seq guided bulk RNA-Seq deconvolution of induced sputum samples with microbial integration. Data preparation and deconvolution. Bulk normalization

We normalized induced sputum bulk RNA seq data using edgeR with a model matrix (DualStatus + omv_z). Sputum bulk RNA-seq counts were processed with edgeR using TMM normalization and filterByExpr, with non-log CPM values supplied to MuSiC; BAL snRNA-seq reference comprised four donors and 5205 nuclei after QC, annotated into 12 major cell types; we constructed donor-specific pseudobulk profiles by summing counts per cell type × donor and used these as the reference, which reduces single-cell noise while preserving donor variability; we applied MuSiC with donor-aware weighting, using only genes shared between bulk and snRNA-seq and constraining fractions to sum to 1 per bulk sample. Four BAL donors and 5205 cells are insufficient for standalone single-cell discovery. In this study, however, the snRNA-seq is used solely as a reference for deconvolution of the larger sputum bulk RNA-seq cohort, not as an independent scRNA-seq discovery dataset. MuSiC is designed for deconvolution with small numbers of reference donors and many cells^53^, and the donor-specific pseudobulk profiles mitigate per-cell noise while retaining the between-donor variation that the algorithm exploits. To assess robustness to reference sampling, we implemented a pseudo-donor perturbation analysis. Briefly, we resampled snRNA-seq cells into 3, 5, 7, and 10 pseudo-donors per cell type, reran MuSiC for each setting, and computed cell-type-wise correlations of estimated fractions between settings. For major populations, such as alveolar macrophages, T cells, and AT1 epithelial cells, correlations between pseudo-donor settings were high, indicating that the MuSiC estimates are numerically stable despite the small reference. We explicitly acknowledge that an independent matched scRNA-bulk dataset would be the ideal validation; such data are not available for this rural HIV-COPD BAL cohort, and additional bronchoscopy was not feasible within this study.

We filtered genes with fewer than 10 counts in at least half the samples and extracted non-log CPM for MuSiC. Final matrix included only genes shared with scRNA-seq reference database. Univariate analyses. After successful deconvolution, we merged proportions with metadata (disease, OMV z-scores) and generated boxplots for DualStatus and OMV quartiles (omv_z divided into labeled quartiles, missing values removed). Using Spearman’s correlation analysis, we assessed the relationships between OMV z-scores and cell-type proportions. For modeling, a baseline linear model (LM) was fit for each cell type, i.e., proportion*∼* omv_z+Smoke_ciga+Age_actual+Sex+BMI. *P*-values for the *omv_z* term were adjusted for multiple testing using the Benjamini–Hochberg (BH) method. For a more flexible approach, we compared LMs to generalized additive models (GAMs) using the Akaike Information Criterion (AIC); a ΔAIC > 2 was taken to indicate a better fit for the GAM (non-linear effect). We then fit interaction models to test whether *omv_z* effects varied by DualStatus or Sex and Dualdisease or Sex, respectively. For GAM-preferred cell types, smooth terms were modeled separately by group (e.g., s(omv_z, by = DualStatus) or s(omv_z, by = Dualdisease)), whereas for LM-preferred cell types, interaction terms were included directly (e.g., omv_z * DualStatus)/ (e.g., omv_z * Dualdisease). Predictions were generated across the observed *omv_z* range for each group, holding confounders constant (smoking = 0, age and BMI at their means, sex at the reference category). Only cell types with a mean proportion >0.01 and a standard deviation >0.01 were included in prediction plots. Finally, we estimated the main effects of DualStatus/Dualdisease (excluding *omv_z*) to quantify group-level differences in cell-type proportions after adjusting for covariates. Multivariate and predictive analyses. To reduce collinearity, we first removed highly correlated cell types (|*r*| > 0.70), such as EC general capillary cells. Multivariate analysis of variance (MANOVA) with Pillai’s trace was then applied to test for overall differences in cell-type composition across disease groups (*DualStatus* or *Dualdisease*), adjusting for smoking status, age, sex, and BMI. For group-level contrasts, we conducted pairwise MANOVAs comparing each disease group to the COPD-/HIV- reference (DualStatus) or comparing *Dualdisease* = 1 vs 0. To explore how cell-type composition related to continuous clinical measures, we performed canonical correlation analysis (CCA) linking the reduced cell-type set to OMV z-scores, age, and BMI. Microbial Genus Integration. Data preprocessing. Genus-level counts were (i) rarefied to equal depth, (ii) zeros imputed with a small pseudocount, and (iii) transformed using the centered log-ratio (CLR) to respect compositional structure. The CLR matrix was then merged with cell-type proportions and metadata (sex, age, BMI, smoking status Smoke_ciga, and omv_z).

### Modeling scenarios

For each cell type-genus pair, we evaluated three questions:

Main effect: Is genus abundance associated with the cell-type proportion?

LM:proportion*∼*genus_abundance+Smoke_ciga+Age_actual+Sex+BMI+omv_z+genus_abundance:Sex

GAM:proportion∼s(genus_abundance)+covariates+s(genus_abundance,by=Sex).

DualStatus interaction: Does the association vary by COPD/HIV group?

LM:proportion∼genus_abundance×DualStatus+covariates+genus_abundance:Sex

GAM:proportion∼s(genus_abundance,by=DualStatus)+DualStatus+covariates+s(genus_abundance,by=Sex). OMV z score interaction: Does the association vary along the continuous OMV axis?

LM:proportion∼genus_abundance×omv_z+DualStatus+covariates+genus_abundance:Sex

GAM: *Covariates in all models:* Smoke_ciga, Age_actual, Sex, BMI. Sex interactions were included explicitly to allow sex-specific slopes/curves.

Inclusion criteria. Pairs were analyzed only if *n* ≥ 30 observations and variance ≥1 × 10^−6^.

#### Model selection and inference

For each pair and scenario, we fit both LM and GAM and compared AIC: ΔAIC = AIC(LM)-AIC(GAM). ΔAIC > 2 → prefer GAM (nonlinear), ΔAIC < − 2 → prefer LM (linear). |ΔAIC| ≤ 2 → tie (reported with LM for interpretability). Term-level *P* values from the preferred model were adjusted with Benjamini–Hochberg (BH); associations were considered significant at *q* < 0.10. For GAMs, significance was assessed on relevant smooth terms, such as (s(genus_abundance, by = DualStatus) or ti(genus_abundance, omv_z)).

#### Human Lung Cell Atlas-guided deconvolution and reference-sensitivity analysis of induced-sputum bulk RNA sequencing

To assess the sensitivity of inferred sputum cellular composition to the choice of single-cell reference, we independently repeated deconvolution using the publicly available HLCA^54^. The annotated HLCA single-cell reference was obtained from the CELLxGENE Human Lung Cell Atlas collection as an AnnData object and processed using Scanpy and AutoGeneS^55–57^. Single-cell counts were normalized to 10,000 counts per cell, and the 5000 most highly variable genes were identified using the Cell Ranger method. Fifteen annotated immune-cell populations, including macrophages, monocytes, T cells, natural killer cells, B cells, plasma cells, and dendritic-cell subsets, were retained to construct reference expression centroids. Cell-type-specific marker genes were selected using AutoGeneS, which applies multi-objective optimization to maximize cell-type specificity while minimizing similarity between reference profiles. Optimization was performed for 5000 generations (offspring size = 100; random seed = 0), yielding 400 informative marker genes. Marker genes shared between the HLCA reference and induced-sputum bulk RNA-sequencing dataset were retained for deconvolution. Cell-type proportions were estimated using ν-support vector regression (ν-SVR), with negative coefficients constrained to zero and estimated proportions normalized to sum to one within each sample. HLCA-derived proportions were obtained for all 100 induced-sputum samples. To compare disease-associated cellular composition under the two independent reference frameworks, HLCA-derived proportions (*n* = 100) and BAL snRNA-seq/MuSiC-derived proportions (*n* = 48) were analyzed separately across the four HIV/COPD groups. For each reference, differences in individual cell populations were assessed using Kruskal–Wallis tests, with Benjamini–Hochberg correction across testable cell populations and epsilon-squared (*ε*²) reported as the omnibus effect size. Where pairwise comparisons were examined, Dunn’s post-hoc tests were performed with Benjamini–Hochberg adjustment across all pairwise comparisons; comparisons of each disease group with the COPD-/HIV- reference group are displayed in Supplementary Fig. S24, S25 17D, E. Reference sensitivity at the level of overall cellular composition was further assessed using compositional multivariate analysis. For each deconvolution framework separately, zero values were accommodated by adding a data-derived pseudocount equal to one-half of the smallest positive inferred cell proportion in that dataset. The resulting compositions were re-closed to sum to one and transformed using the centered log-ratio (CLR) transformation. Principal-component analysis (PCA) was then used to visualize compositional structure. Global differences among the four HIV/COPD groups were tested separately within each reference framework using PERMANOVA with 9999 permutations. Homogeneity of multivariate dispersion was assessed using Euclidean distances in CLR space from each sample to its group centroid, followed by a median-centered Levene test.

#### Statistics and reproducibility

This was an observational longitudinal study of adults stratified by HIV and COPD status, with baseline and approximately 2-year follow-up assessments. Sample size for the microbiome component was predetermined based on taxonomic profiles from Human Microbiome Project data. With *n* = 50 participants per group, an assumed effect size of Cohen’s *d* = 0.8 and an *α* threshold of 0.05/30 to account for 30 major airway bacterial genera, the estimated power was approximately 80%. Sample sizes for individual analyses varied according to availability of specimens and assay-specific data and are reported in the corresponding figures, tables, and “Methods” sections.

Analyses were performed using participants with available data for the variables required for each analysis (Supplementary Table 17). No imputation of missing participant-level covariate, exposure, outcome, or follow-up data was performed. Samples or observations failing prespecified assay-specific quality-control criteria were excluded as described in the relevant “Methods” sections; no additional data were excluded on the basis of study outcomes. Statistical methods, including multiple-testing corrections where applicable, are described in the corresponding “Methods” sections and figure legends.

The study was observational, and participants were not allocated to experimental treatment groups; therefore, randomization and blinding to experimental allocation were not applicable. Investigators were not blinded to participant HIV or COPD status during outcome assessment and data analysis. Biological sample sizes and the distinction between independent participants and repeated measurements are reported for the corresponding analyses. Technical replicates were not treated as independent biological observations.

## Supplementary information

Supplementary File

Transparent Peer Review File

## Source data

Source data

## Data Availability

The data generated in this study have been deposited in public repositories. Single-cell and Bulk RNA-sequencing data are publicly available in the NCBI Gene Expression Omnibus (GEO) under accession numbers GSE326017 (https://www.ncbi.nlm.nih.gov/gds/?term=GSE326017) and GSE326058 (https://www.ncbi.nlm.nih.gov/gds/?term=GSE326058), respectively. Baseline raw 16S rRNA gene sequencing data are publicly available in the NCBI Sequence Read Archive (SRA) under BioProject accession PRJNA726058 (https://www.ncbi.nlm.nih.gov/sra/?term=PRJNA726058). Follow-up raw 16S rRNA gene sequencing data are publicly available in the NCBI Sequence Read Archive (SRA) under BioProject accession PRJNA1526773 (https://www.ncbi.nlm.nih.gov/sra/?term=PRJNA1526773). Processed datasets and supporting data necessary to reproduce the analyses are publicly available through Zenodo (https://zenodo.org/records/21473246), referenced through the following https://doi.org/10.5281/zenodo.21473246. The archive contains de-identified sample metadata, processed analysis inputs, primary microbiome analysis files, and source data underlying the transcriptomic, single-cell, CyTOF, and MetaDeconfoundR analyses. Analysis files supporting HLCA deconvolution with AutoGeneS and the HLCA-based versus MuSiC cell-type deconvolution sensitivity analyses are provided with this article as Source data files. These publicly deposited datasets contain no confidential patient information. Source data are provided with this paper.

## Code availability

Analysis code used to generate the results reported in this study is publicly available through Zenodo (https://zenodo.org/records/21473246), referenced through the following https://doi.org/10.5281/zenodo.21473246. The archive includes complete R scripts for preprocessing, alpha- and beta-diversity analyses, PERMANOVA, genus-level mixed models, batch/drift correction, genus-cell-fraction models, RNA-seq association analyses, gene-microbe linear models, MetaDeconfoundR analyses for the full and HIV-stratified cohorts, and lung-function analyses; Processed microbiome inputs, including rarefied and filtered OTU-, genus-, and phylum-level abundance tables, rarefied read tables, the OTU-genus mapping table, and sample metadata; Primary 16S microbiome analysis files, including the OTU BIOM table, representative sequences, taxonomic assignments, phylogenetic tree, contaminant information, LotuS logs, and higher-level taxonomic abundance tables. Code files supporting HLCA deconvolution with AutoGeneS and the HLCA-based versus MuSiC cell-type deconvolution sensitivity analyses are provided with this article as Supplementary Software 1.
