# Supplementary material for: Microbial ecological instability and non-linear host interactions shape airway inflammation in HIV-associated COPD
Source: Nat Commun. 2026 Oct 6;17:11543. doi: 10.1038/s41467-026-78400-4 (PMC13748602; doi:10.1038/s41467-026-78400-4)
Supplement: Supplementary file 1 [file 41467_2026_78400_MOESM1_ESM.pdf]

Supplementary Information

Microbial Ecological Instability And Non-Linear Host Interactions Shape Airway Inflammation In HIV-Associated COPD

Alex Kayongo[1,2*†], Theda Ulrike Patricia Bartolomaeus[3,4,5,6*], Julia Hecker[7*], Till Birkner [3,4,5,6*], Eremire Vila[3,4], Moses Levi Ntayi[1,2,8,9], Lajos Markó[3,4,5,6], Ulrike Löber[3,4,5,6], Geoffrey Olweny[1,2,10], Edward Kyalo[1,2], Daisy Kyamulabi[1], Josephine Ndawula[1,2], Willy Ssengooba[1,2], Rose Nabatanzi[1], Samuel Kyobe[11], Faith Nassali[1,2], Edgar Kigozi[1], Robert Kalyesubula[12,13], Richard Munana[12], Jesca Namaganda[1,2], Caroline J. Musiime[1,2], Rogers Sekibira[1,2], Bernard S. Bagaya[1], David P. Kateete[1], Moses L. Joloba[1], Daudi Jjingo[14,15], John Lusiba[2,16], William Worodria[16,17], Harriet Mayanja-Kizza[16], Rejani Lalitha[16,17], Wasswa Ham[18], Ingvar Sanyu[16,17], Josephine Zawedde[16,17], Nicole M. Robertson[19], Obondo James Sande[1], Carl Weidinger[7], Desiree Kunkel[20], Bruce J. Kirenga[2,16], Trishul Siddharthan[21], John Hurst[22], William Checkley[19,23], William B. Cookson[24], Miriam F. Moffatt[24], and Sofia K. Forslund-Startceva [4,5,6,19,25†].

Supplementary Figure S1. Impact of disease status on longitudinal changes in lung function. Changes (Δ) in lung-function measures between baseline and follow-up are shown across the four clinical groups (COPD+/HIV+, COPD-/HIV-, COPD+/HIV-, and COPD-/HIV+). Analyses included *n* = 40 biologically independent participants with paired baseline and follow-up measurements in each clinical group (total *n* = 160). Each point represents one participant, which constitutes the biological unit of study; no technical replicates were treated as independent observations. Boxplots show the median (centre line), interquartile range (box), and whiskers extending to 1.5 × the interquartile range. Between-group differences in longitudinal change were assessed using multivariable linear regression models adjusted for age, sex, body mass index (BMI), smoking status, and the corresponding baseline lung-function measure. Statistical significance was defined as $P < 0.05$. *p<0.05, **p<0.01, *** p<0.001. Source data are provided as a Source Data file.

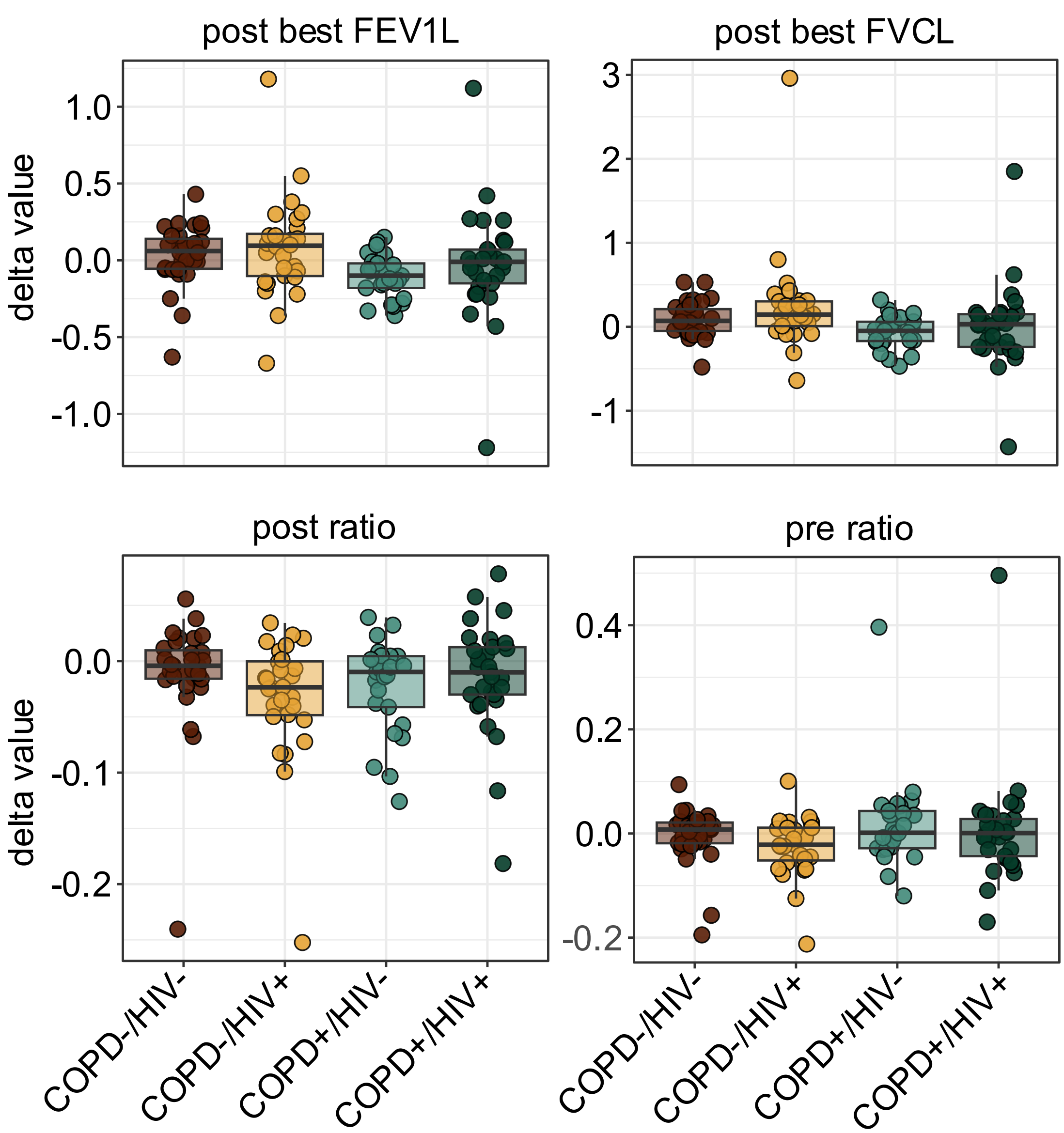

Supplementary Figure S2: **Change in lung function (Δ = V2-V1) by disease status (*DualStatus*), adjusted for age, sex, BMI, and smoking status.** Changes in lung function were evaluated among n = 160 biologically independent participants with paired baseline and follow-up measurements: COPD-/HIV-, *n* = 40; COPD-/HIV+, *n* = 40; COPD+/HIV-, *n* = 40; and COPD+/HIV+, *n* = 40. Points represent adjusted estimated differences in Δ for each disease group relative to the COPD-/HIV- reference group, and horizontal bars indicate 95% confidence intervals. Outcomes include pre- and post-bronchodilator FEV_1 (L), pre- and post-bronchodilator FVC (L), and post-bronchodilator FEV_1/FVC ratio. Models were adjusted for age, sex, body mass index (BMI), smoking status, and the corresponding baseline lung-function measure. Each participant constitutes the biological unit of study; no technical replicates were treated as independent observations. Source data are provided as a Source Data file.

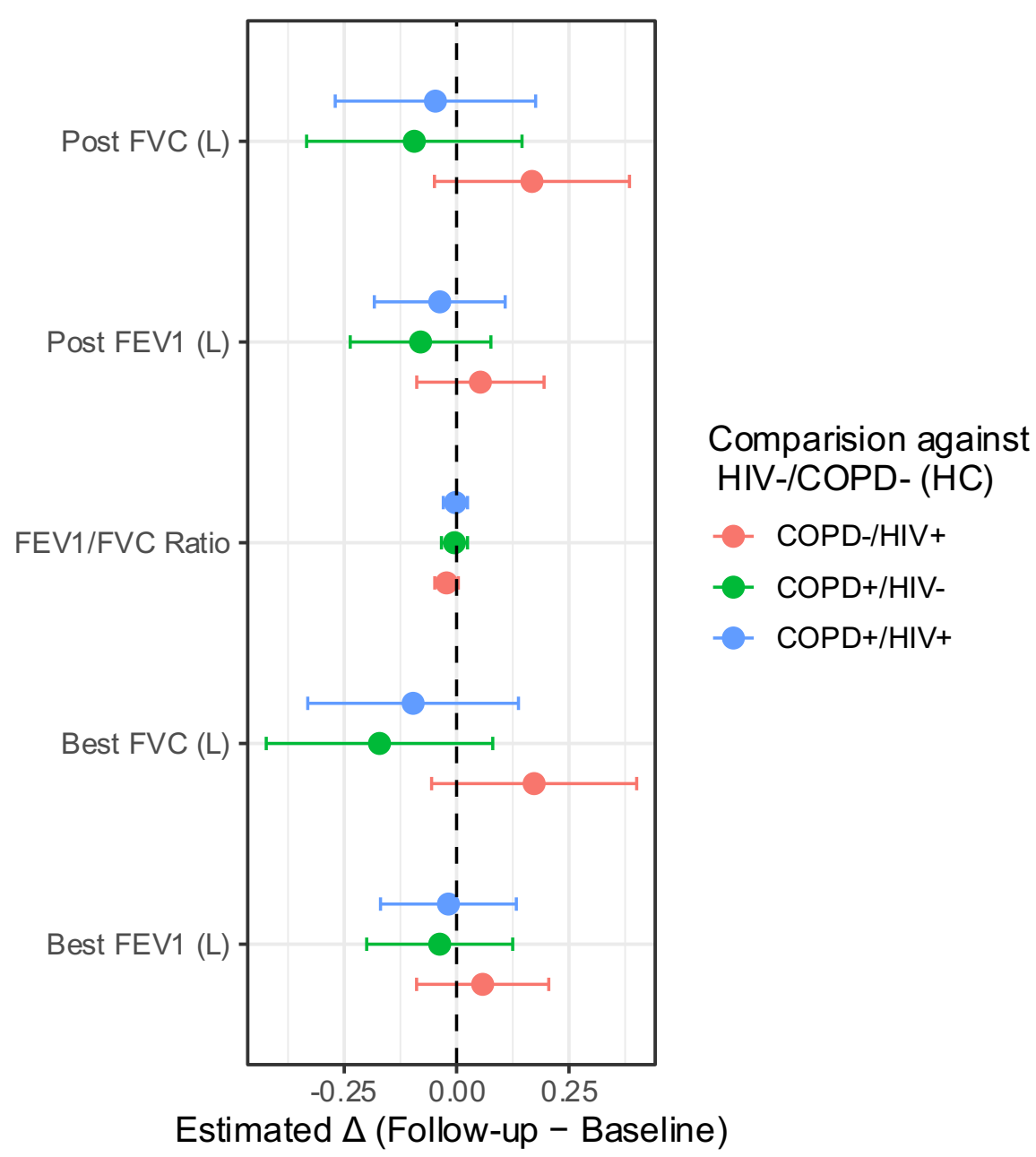

Supplementary Figure S3. Genus-level longitudinal changes in the induced sputum microbiome by clinical group from visit 1 to visit 2. Paired genus-level changes between visit 1 and visit 2 were evaluated among *n* = 101 biologically independent participants with paired microbiome profiles: COPD-/HIV-, *n* = 26; COPD-/HIV+, *n* = 28; COPD+/HIV-, *n* = 24; and COPD+/HIV+, *n* = 23. Each point represents a bacterial genus and shows the $\log_2$ fold change (visit 2 versus visit 1) and $-\log_{10}$ FDR-adjusted P value from paired Wilcoxon signed-rank tests within each clinical group. Dashed lines indicate $|\log_2 \text{ fold change}| > 1$ and FDR < 0.10. Each participant constitutes the biological unit of study; no technical replicates were treated as independent observations. Source data are provided as a Source Data file.

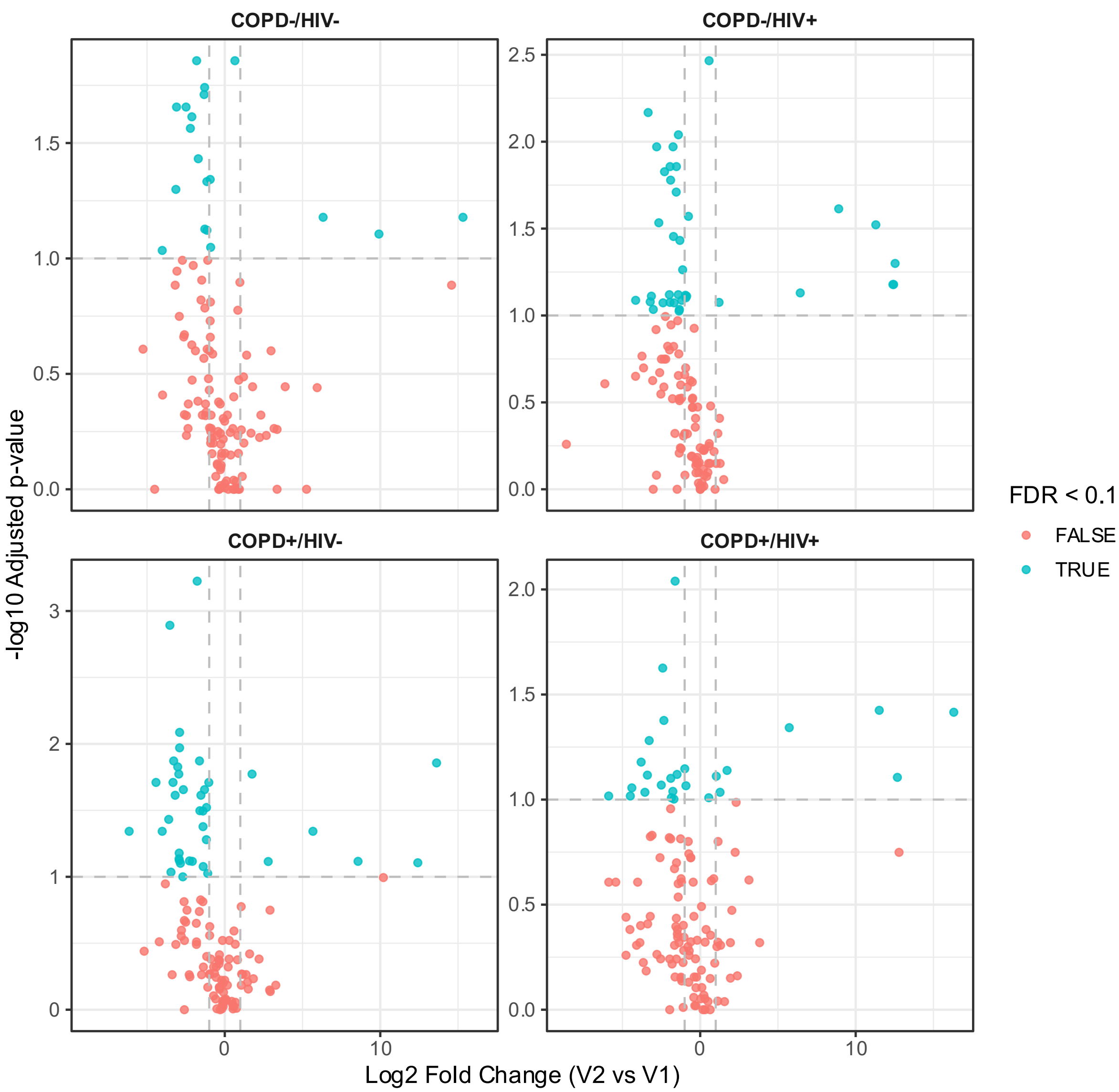

Supplementary Figure S4. Longitudinal changes in bacterial genera across visits. Mixed-effects model estimates of the average change in genus abundance from visit 1 to visit 2, adjusted for clinical group (COPD/HIV status), age, sex, body mass index (BMI), and smoking status. The analysis included 356 microbiome samples from 200 biologically independent participants: *n* = 200 at visit 1 and *n* = 156 at visit 2. Participant ID was included as a random effect to account for repeated measurements. Points show effect estimates (β) and horizontal bars indicate 95% confidence intervals; only genera with an FDR-adjusted *P* < 0.10 for the visit term are shown. Individual participants constitute the biological units of study; repeated measurements from the same participant are not treated as independent biological replicates, and no technical replicates were treated as independent observations. Source data are provided as a Source Data file.

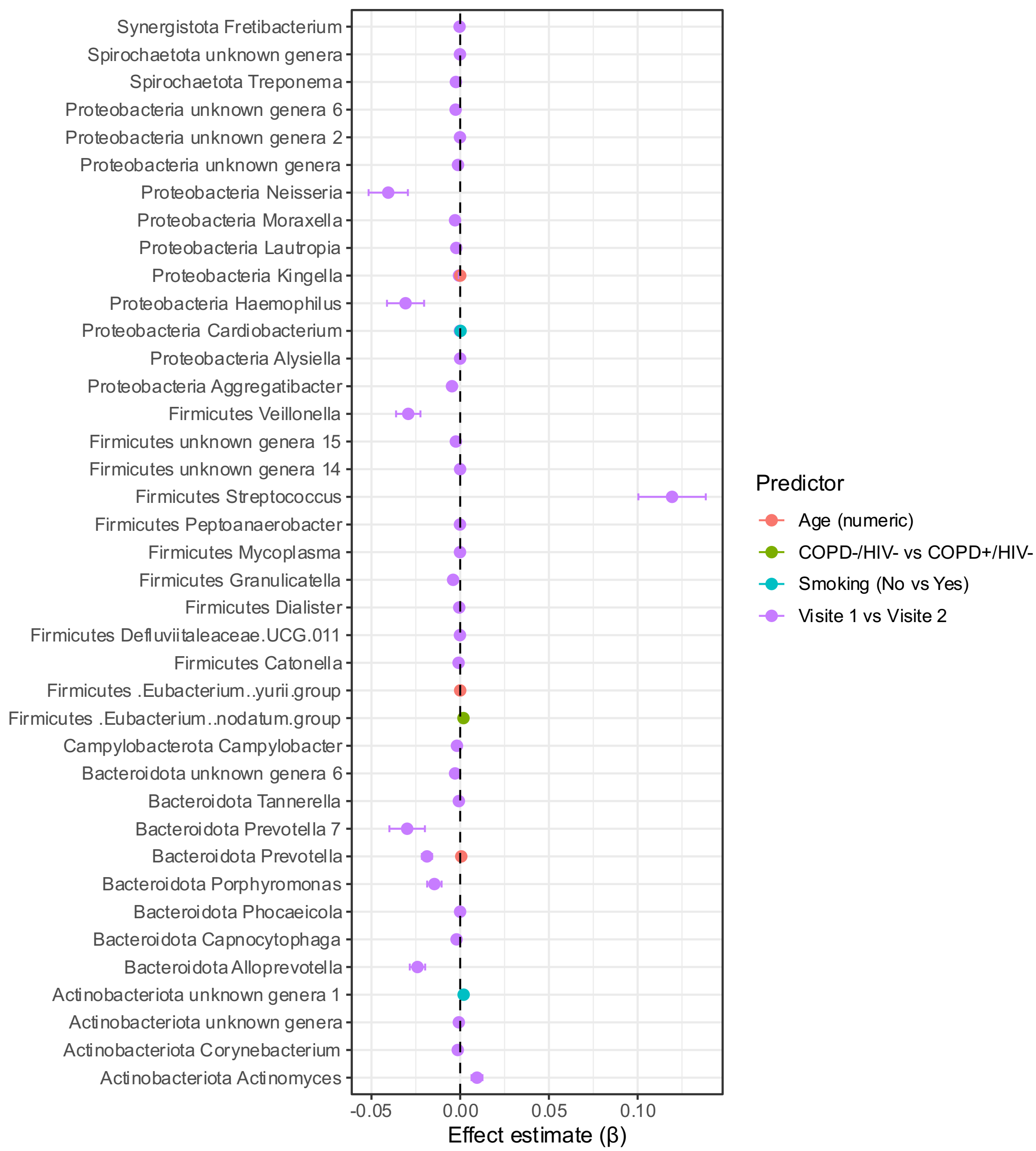

Supplementary Figure S5. Within-individual longitudinal changes in bloomer and decreaser bacterial genera among healthy controls. Summed relative abundances of bacterial genera classified as bloomers (left) or decreasers (right) are shown between baseline (visit 1) and follow-up (visit 2) in *n* = 26 biologically independent COPD-/HIV- participants with paired microbiome profiles. Bloomer and decreaser genera were defined as taxa exhibiting significant increases or decreases, respectively, in the longitudinal differential-abundance analysis. Each point represents an individual participant, and grey lines connect paired measurements from the same participant. Relative abundances were summed across genera within each category for each participant. Each participant constitutes the biological unit of study; repeated measurements from the same participant are not independent biological replicates, and no technical replicates were treated as independent observations. Source data are provided as a Source Data file.

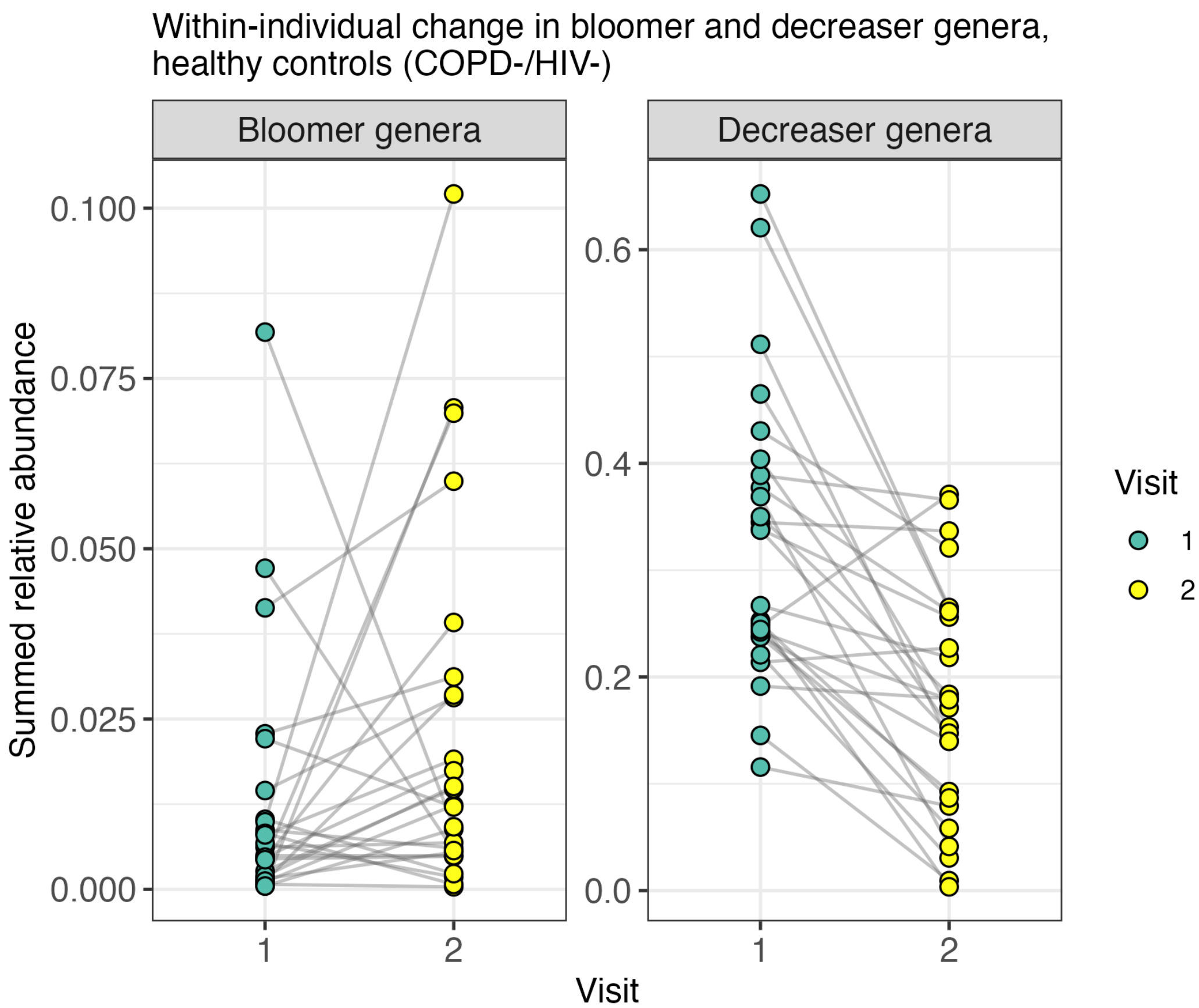

Supplementary Figure S6: Distribution of unadjusted *P* values for visit × clinical group interactions. Histograms show unadjusted P values for the visit × clinical group interaction term from longitudinal differential-abundance models comparing COPD−/HIV+, COPD+/HIV−, and COPD+/HIV+ with the COPD-/HIV- reference group. The longitudinal microbiome dataset comprised 356 samples from 200 biologically independent participants: at baseline, *n* = 50 participants per clinical group; at follow-up, COPD-/HIV- *n* = 36, COPD-/HIV+ *n* = 40, COPD+/HIV− *n* = 41, and COPD+/HIV+ *n* = 39. Participant ID was included as a random effect to account for repeated measurements. Under the null hypothesis, P values are expected to follow a uniform distribution; the dashed red line indicates the expected number of genera per histogram bin under this distribution. Individual participants constitute the biological units of study; repeated measurements from the same participant are not independent biological replicates, and no technical replicates were treated as independent observations. Source data are provided as a Source Data file.

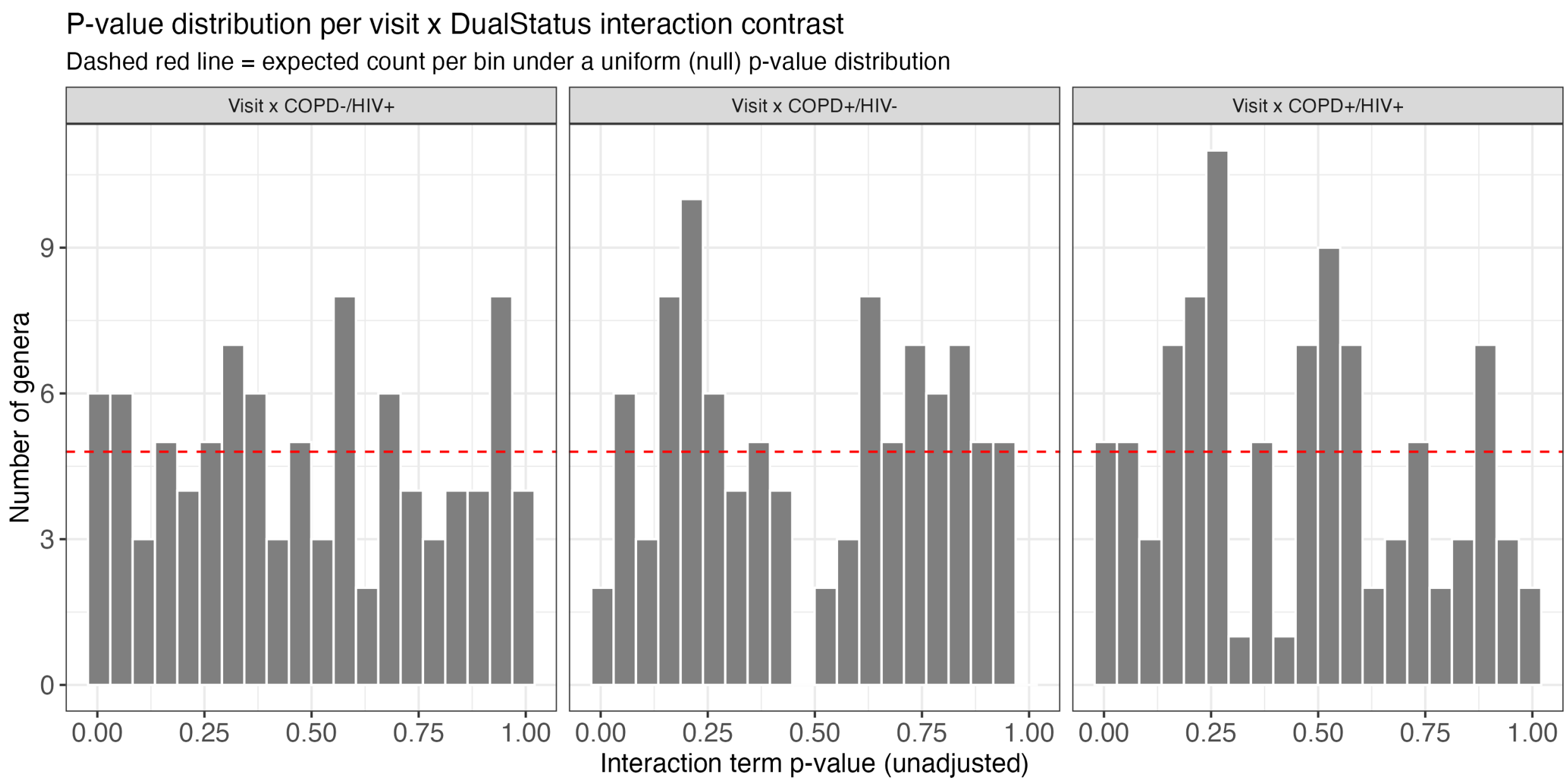

Supplementary Figure S7: Associations between differentially abundant bacterial genera and clinical metadata by HIV/COPD status. Heatmaps show multivariable-adjusted associations between bacterial genera (rows) and clinical variables (columns) for COPD+/HIV+ (left), COPD+/HIV- (middle), and COPD-/HIV+ (right), relative to the COPD-/HIV- reference group. The longitudinal microbiome dataset comprised 356 samples from 200 biologically independent participants: at baseline, *n* = 50 participants per clinical group; at follow-up, COPD-/HIV− *n* = 36, COPD-/HIV+ *n* = 40, COPD+/HIV− *n* = 41, and COPD+/HIV+ *n* = 39. Colours represent Cliff's *δ* effect sizes. Open circles denote associations attenuated after adjustment for potential confounders, whereas asterisks denote associations remaining significant after multivariable adjustment (*FDR < 0.10, **FDR < 0.01, ***FDR < 0.001). Individual participants constitute the biological units of study; repeated measurements from the same participant are not independent biological replicates, and no technical replicates were treated as independent observations. Source data are provided as a Source Data file.

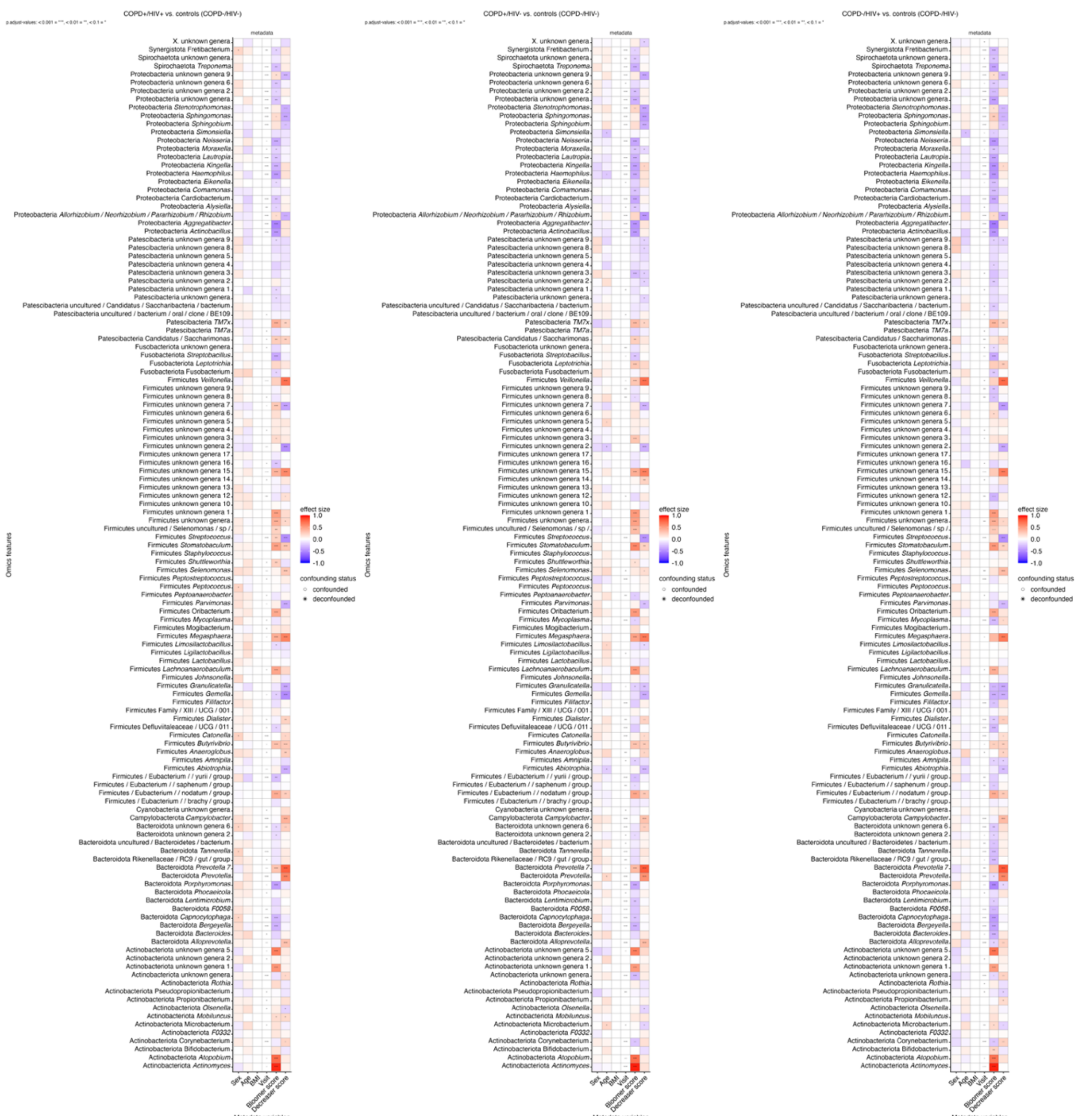

Supplementary Figure S8. **Effect of baseline genus abundance on change over time (*Δ* = V2 - V1) in healthy controls and disease groups.** Associations between baseline genus abundance and within-participant change (Δ = visit 2 − visit 1) were evaluated in n = 101 biologically independent participants with paired microbiome profiles: COPD-/HIV-, *n* = 26; COPD−/HIV+, *n* = 28; COPD+/HIV−, *n* = 24; and COPD+/HIV+, *n* = 23. Genera shown had significant baseline abundance × clinical group interactions (likelihood-ratio test, FDR < 0.10). Points represent model coefficient estimates (β), and horizontal bars indicate 95% confidence intervals. Models were adjusted for age, sex, body mass index (BMI), and smoking status. The baseline-abundance coefficient represents the slope in the COPD-/HIV- reference group; interaction coefficients represent differences in slope between each disease group and the reference group. Each participant constitutes the biological unit of study; no technical replicates were treated as independent observations. Source data are provided as a Source Data file.

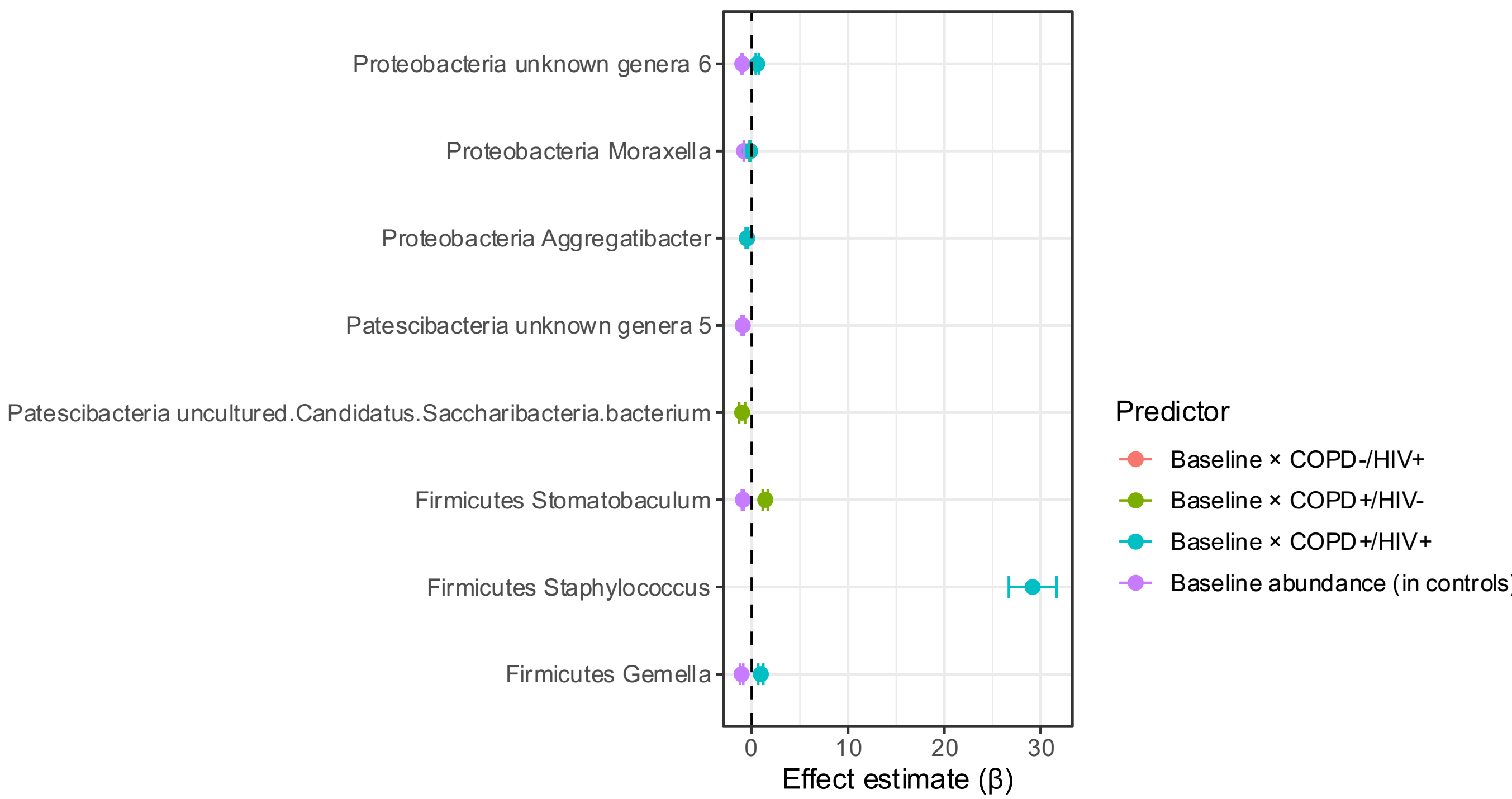

Supplementary Figure S9. Bray-Curtis distance to baseline across HIV/COPD clinical groups. Bray-Curtis distances between paired baseline and follow-up microbiome profiles were evaluated in *n* = 101 biologically independent participants: COPD-/HIV-, *n* = 26; COPD-/HIV+, *n* = 28; COPD+/HIV−, *n* = 24; and COPD+/HIV+, *n* = 23. Left, observed within-participant Bray-Curtis distances between baseline and follow-up. Boxplots show the median (centre line), interquartile range (box), and whiskers extending to 1.5 × the interquartile range; points represent individual participants. Right, observed Bray-Curtis distances with model-adjusted means (white circles) and 95% confidence intervals. Model estimates were adjusted for age, sex, body mass index (BMI), and smoking status. Each participant constitutes the biological unit of study; no technical replicates were treated as independent observations. Source data are provided as a Source Data file.

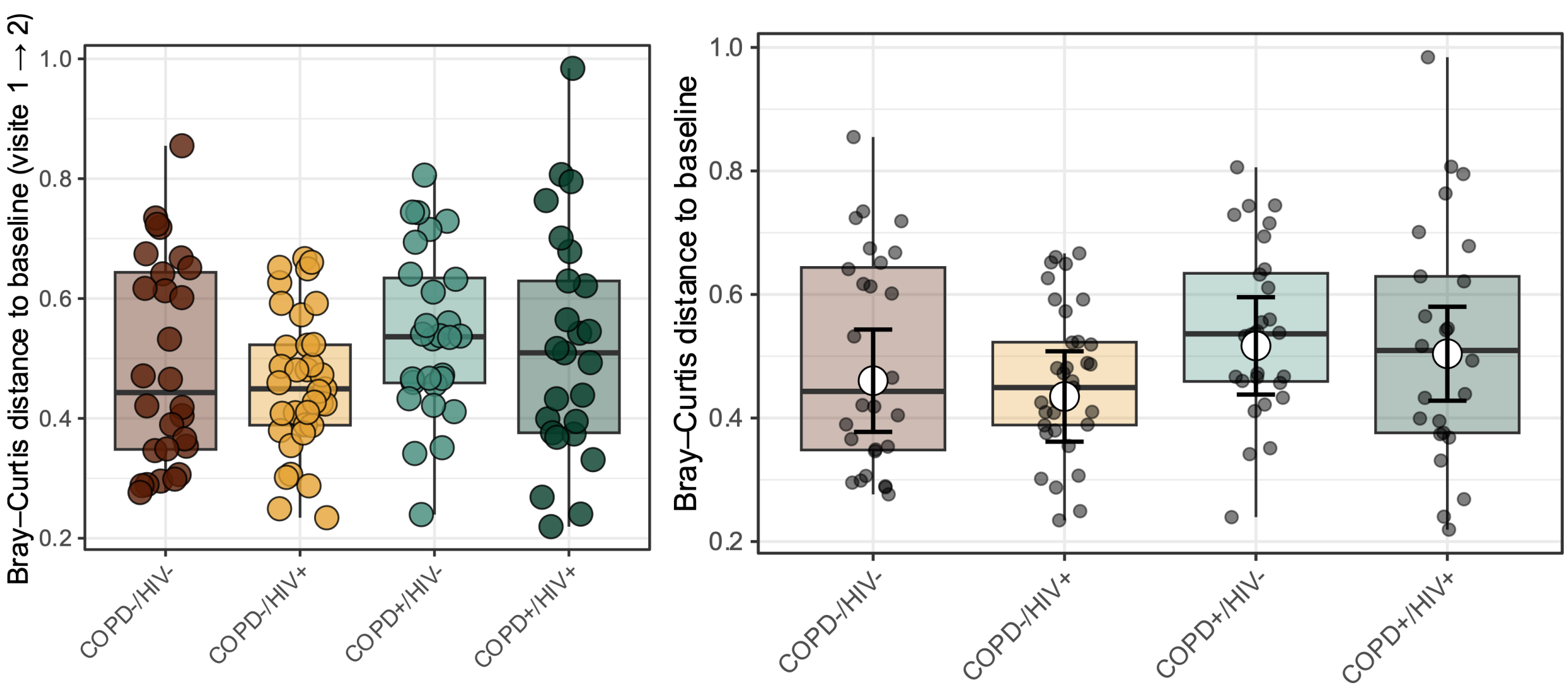

Supplementary Figure S10. Variance explained by PERMANOVA terms across four models. Bars show the proportion of variation in Bray-Curtis dissimilarity explained (R^2, %) by individual terms across four PERMANOVA models. Analyses included 202 microbiome samples from *n* = 101 biologically independent participants with paired visit 1 and visit 2 microbiome profiles: COPD-/HIV-, *n* = 26; COPD-/HIV+, *n* = 28; COPD+/HIV-, *n* = 24; and COPD+/HIV+, *n* = 23. Bray-Curtis dissimilarities were calculated from the rarefied and filtered OTU count table. PERMANOVA models used 999 permutations constrained within participant ID to account for paired measurements. Bars show R^2 as percentages, with significance indicated as $P \leq 0.05$ (*), $P \leq 0.01$ (**), $P \leq 0.001$ (***), and $P \leq 0.10$ (.) Each participant constitutes the biological unit of study; repeated measurements from the same participant are not independent biological replicates, and no technical replicates were treated as independent observations. Source data are provided as a Source Data file.

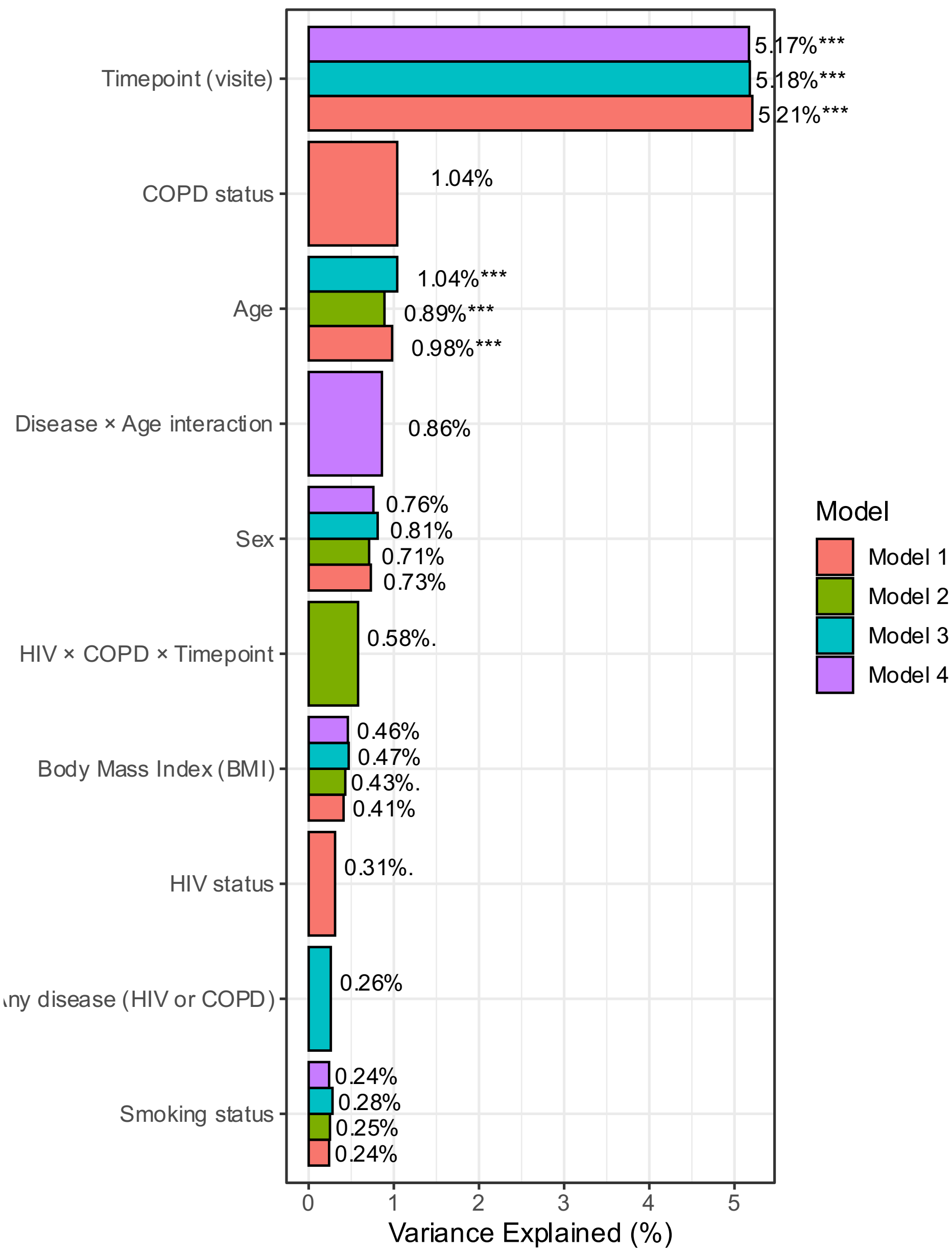

Supplementary Figure S11. Model-adjusted associations between airway microbiota and clinical covariates. Heatmaps show associations between airway bacterial genera (rows) and clinical or demographic covariates (columns) across three modeling frameworks: naïve (left), participant ID as a random effect (ID_random; middle), and participant ID as a fixed effect (ID_fixed; right). Analyses included 202 microbiome samples from *n* = 101 biologically independent participants with paired visit 1 and visit 2 microbiome profiles: COPD-/HIV-, *n* = 26; COPD-/HIV+, *n* = 28; COPD+/HIV−, *n* = 24; and COPD+/HIV+, *n* = 23. Colours indicate the direction of association (orange, positive; blue, negative), and asterisks denote statistically significant associations after multiple-testing correction (FDR < 0.05). Each participant constitutes the biological unit of study; repeated measurements from the same participant are not independent biological replicates, and no technical replicates were treated as independent observations. Source data are provided as a Source Data file.

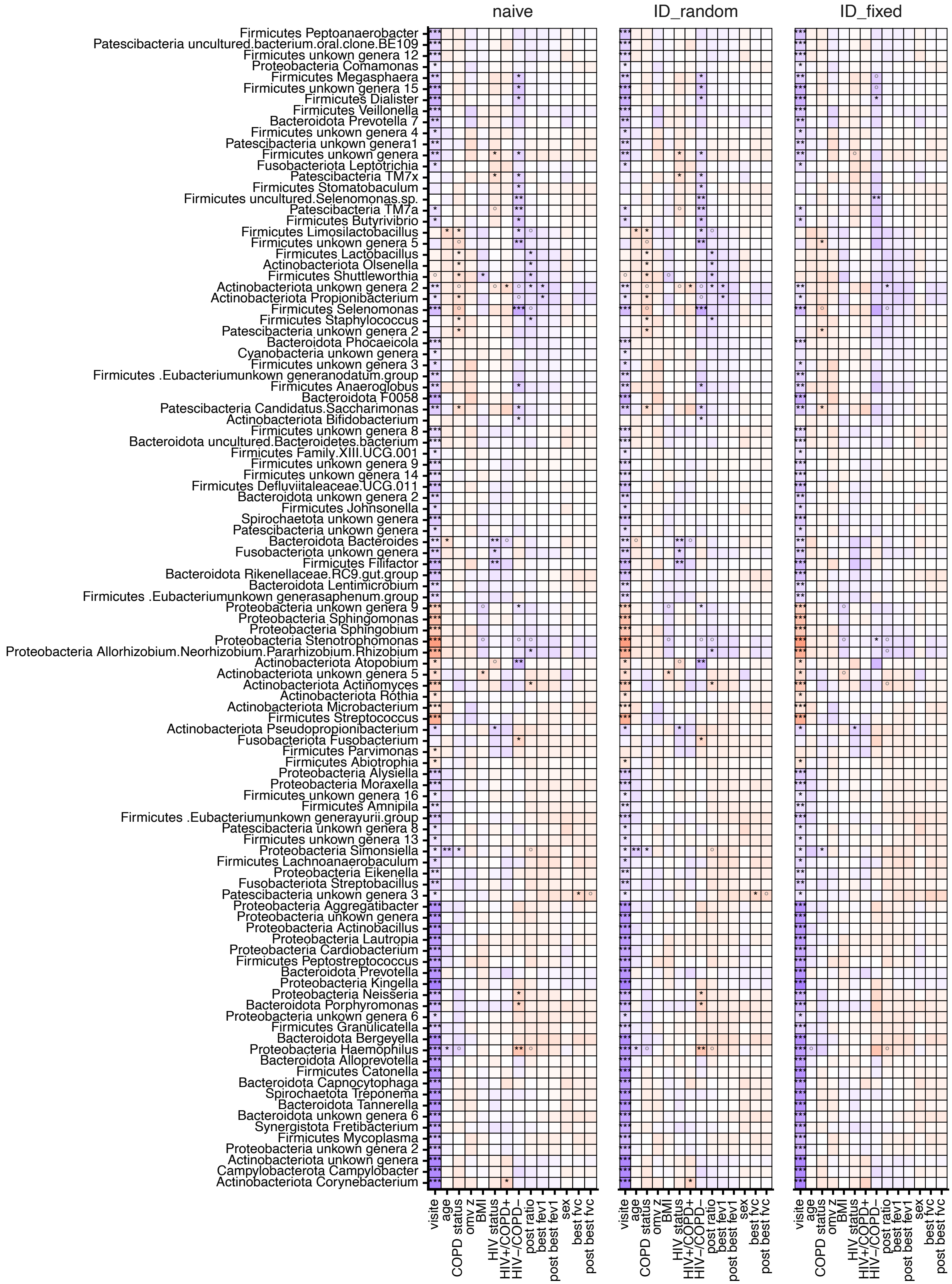
naive
ID_random
ID_fixed
Firmicutes Peptoanaerobacter
Patescibacteria uncultured.bacterium.oral.clone.BE109
Firmicutes unkown genera 12
Proteobacteria Comamonas
Firmicutes Megasphaera
Firmicutes unkown genera 15
Firmicutes Dialister
Firmicutes Veillonella
Bacteroidota Prevotella 7
Firmicutes unkown genera 4
Patescibacteria unkown genera1
Firmicutes unkown genera
Fusobacteriota Leptotrichia
Patescibacteria TM7x
Firmicutes Stomatobaculum
Firmicutes uncultured.Selenomonas.sp.
Patescibacteria TM7a
Firmicutes Butyrivibrio
Firmicutes Limosilactobacillus
Firmicutes unkown genera 5
Firmicutes Lactobacillus
Actinobacteriota Olsenella
Firmicutes Shuttleworthia
Actinobacteriota unkown genera 2
Actinobacteriota Propionibacterium
Firmicutes Selenomonas
Firmicutes Staphylococcus
Patescibacteria unkown genera 2
Bacteroidota Phocaeicola
Cyanobacteria unkown genera
Firmicutes unkown genera 3
Firmicutes .Eubacteriumunkown generanodatum.group
Firmicutes Anaeroglobus
Bacteroidota F0058
Patescibacteria Candidatus.Saccharimonas
Actinobacteriota Bifidobacterium
Firmicutes unkown genera 8
Bacteroidota uncultured.Bacteroidetes.bacterium
Firmicutes Family.XIII.UCG.001
Firmicutes unkown genera 9
Firmicutes unkown genera 14
Firmicutes Defluviitaleaceae.UCG.011
Bacteroidota unkown genera 2
Firmicutes Johnsonella
Spirochaetota unkown genera
Patescibacteria unkown genera
Bacteroidota Bacteroides
Fusobacteriota unkown genera
Firmicutes Filifactor
Bacteroidota Rikenellaceae.RC9.gut.group
Bacteroidota Lentimicrobium
Firmicutes .Eubacteriumunkown generasaphenum.group
Proteobacteria unkown genera 9
Proteobacteria Sphingomonas
Proteobacteria Sphingobium
Proteobacteria Stenotrophomonas
Proteobacteria Allorhizobium.Neorhizobium.Pararhizobium.Rhizobium
Actinobacteriota Atopobium
Actinobacteriota unkown genera 5
Actinobacteriota Actinomyces
Actinobacteriota Rothia
Actinobacteriota Microbacterium
Firmicutes Streptococcus
Actinobacteriota Pseudopropionibacterium
Fusobacteriota Fusobacterium
Firmicutes Parvimonas
Firmicutes Abiotrophia
Proteobacteria Alysiella
Proteobacteria Moraxella
Firmicutes unkown genera 16
Firmicutes Amnipila
Firmicutes .Eubacteriumunkown generayurii.group
Patescibacteria unkown genera 8
Firmicutes unkown genera 13
Proteobacteria Simonsiella
Firmicutes Lachnoanaerobaculum
Proteobacteria Eikenella
Fusobacteriota Streptobacillus
Patescibacteria unkown genera 3
Proteobacteria Aggregatibacter
Proteobacteria unkown genera
Proteobacteria Actinobacillus
Proteobacteria Lautropia
Proteobacteria Cardiobacterium
Firmicutes Peptostreptococcus
Bacteroidota Prevotella
Proteobacteria Kingella
Proteobacteria Neisseria
Bacteroidota Porphyromonas
Proteobacteria unkown genera 6
Firmicutes Granulicatella
Bacteroidota Bergeyella
Proteobacteria Haemophilus
Bacteroidota Alloprevotella
Firmicutes Catonella
Bacteroidota Capnocytophaga
Spirochaetota Treponema
Bacteroidota Tannerella
Bacteroidota unkown genera 6
Synergistota Fretibacterium
Firmicutes Mycoplasma
Proteobacteria unkown genera 2
Actinobacteriota unkown genera
Campylobacterota Campylobacter
Actinobacteriota Corynebacterium
visite
age
COPD status
omv z
BMI
HIV status
HIV+/COPD+
HIV−/COPD−
post ratio
best fev1
post best fev1
sex
best fvc
post best fvc

Supplementary Figure S12. Associations between longitudinal changes in lung function and microbial diversity stratified by disease status. Heatmap shows associations between changes (Δ = visit 2 − visit 1) in lung-function measures and changes in microbial diversity, including Shannon diversity and principal components of beta diversity. Analyses included *n* = 101 biologically independent participants with paired longitudinal microbiome profiles: COPD-/HIV-, *n* = 26; COPD-/HIV+, *n* = 28; COPD+/HIV-, *n* = 24; and COPD+/HIV+, *n* = 23. Values within cells represent regression coefficients. Linear regression models were used to evaluate associations between longitudinal changes in lung function and microbial diversity, adjusting for sex and clinical group (COPD/HIV status). Each participant constitutes the biological unit of study; no technical replicates were treated as independent observations. Source data are provided as a Source Data file.

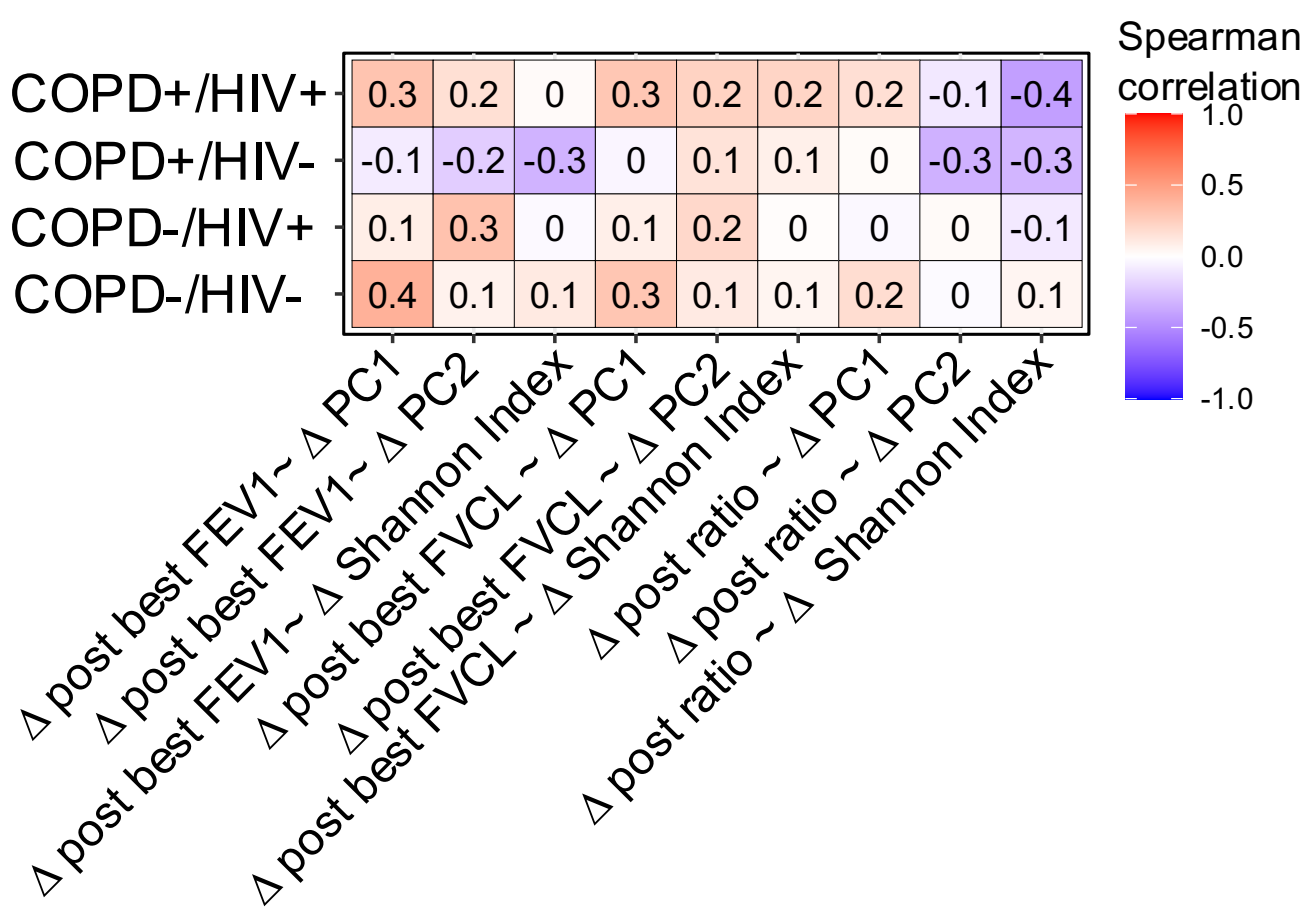

Supplementary Figure S13. OTUs with significant microbiome stability × disease-group interactions (q < 0.1). Relative abundances of representative OTUs are shown across longitudinal microbiome-stability strata, defined using tertiles of each participant's within-individual OTU-level Bray-Curtis dissimilarity between visit 1 and visit 2. Points represent individual participants; boxplots show the median and interquartile range. Stability-stratified analyses included 101 participants with paired microbiome profiles: Stable (n = 34), Medium (n = 34), and Unstable (n = 33). Within disease groups, the Stable/Medium/Unstable sample sizes were COPD-/HIV-: n = 12/4/10; COPD-/HIV+: n = 10/11/7; COPD+/HIV-: n = 4/12/8; and COPD+/HIV+: n = 8/7/8, respectively. Colours indicate DualStatus. Because some Stability × DualStatus subgroups were small, notably COPD-/HIV- Medium (n = 4) and COPD+/HIV- Stable (n = 4), these stability-stratified taxon-level associations should be interpreted as exploratory. Source data are provided as a Source Data file.

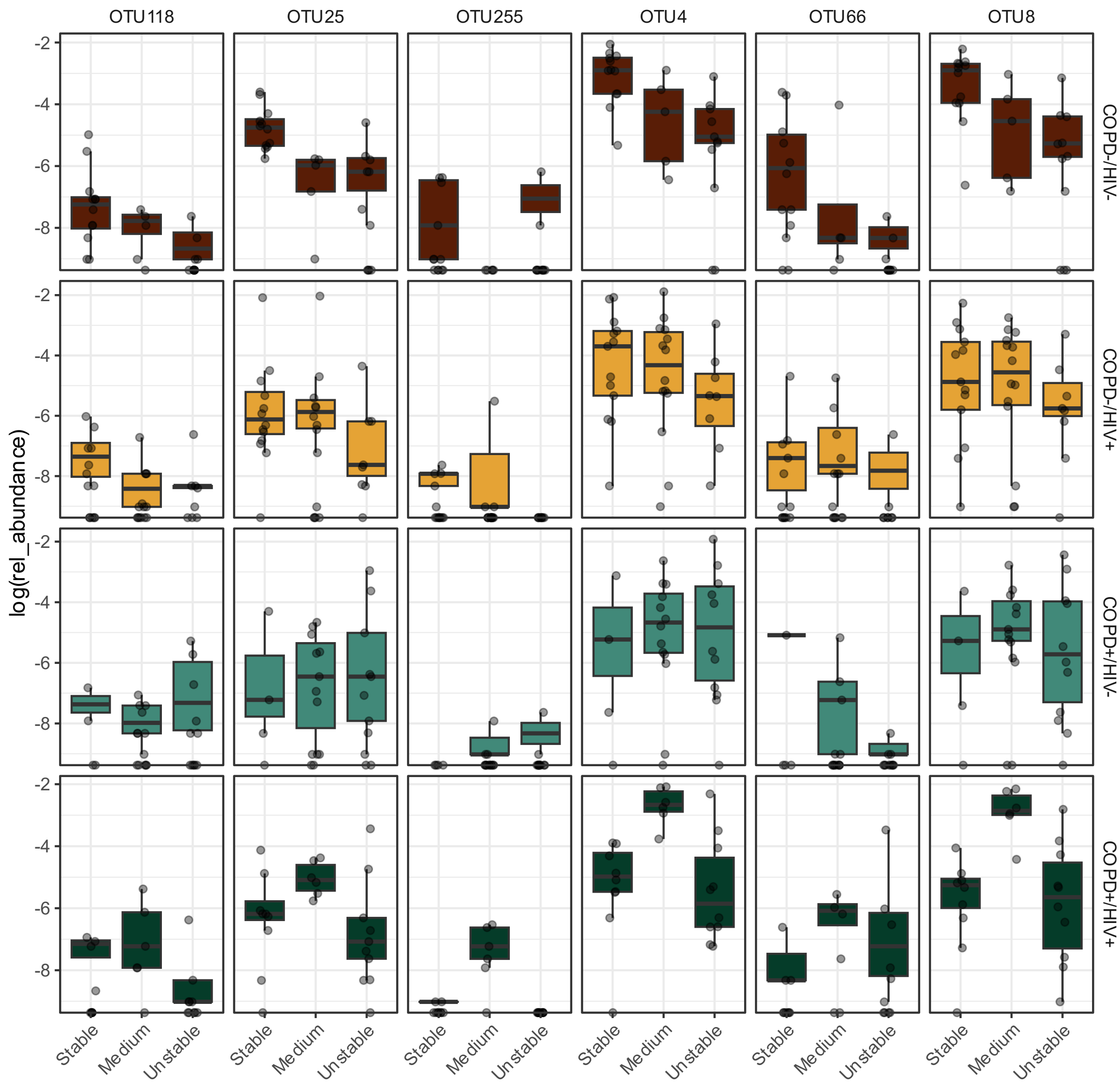

Supplementary Figure S14. Bacterial-derived OMVs and variance in microbiome diversity. (a) Scatter plots showing Spearman correlation analysis between ranked outer membrane vesicle (OMV) concentration and microbiome diversity (Shannon index) across patient subgroups: COPD-/HIV− (brown; *n* = 26), COPD+/HIV- (yellow; *n* = 24), COPD-/HIV+ (light green; *n* = 28), and COPD+/HIV+ (dark green; *n* = 23). Blue lines represent fitted regression lines with 95% confidence intervals (shaded areas). Spearman's rank correlation coefficient (*ρ*) and *P* values are displayed for each subgroup. (b) Boxplots depicting OMV concentration according to HIV and COPD status. Linear models were applied to test the significance of disease status on OMV concentration, using sex and age as covariates. Boxplots show the median (centre line), interquartile range (box), and whiskers extending to the most extreme data points within 1.5 × the interquartile range. (c) Heatmap showing bacterial taxa significantly associated with participants' clinical characteristics. Effect sizes (Cliff's *δ* for categorical factors and Spearman's ρ for continuous features) are represented by cell colours. Statistical significance: FDR < 0.1 (*), FDR < 0.01 (**), and FDR < 0.001 (***). Black stars indicate associations that remained significant after multi-confounder testing (strictly deconfounded [SD], laxly deconfounded [LD], or no other covariates [NC]). Grey circles represent confounded signals. (d) Bray-Curtis distance at visit 1 (V1) for different OMV concentration groups (Low, Medium, High), stratified by dual disease status: COPD-/HIV- (brown; *n* = 26), COPD+/HIV− (yellow; *n* = 24), COPD-/HIV+ (light green; *n* = 28), and COPD+/HIV+ (dark green; *n* = 23). OMV groups were defined based on quartiles of OMV concentration. Brackets demarcate results for Low versus Medium versus High OMV categories. Analyses included *n* = 101 biologically independent participants with paired microbiome profiles. Each participant constituted the biological unit of study; repeated measurements from the same participant were not considered independent biological replicates, and no technical replicates were treated as independent observations. Source data are provided as a Source Data file.

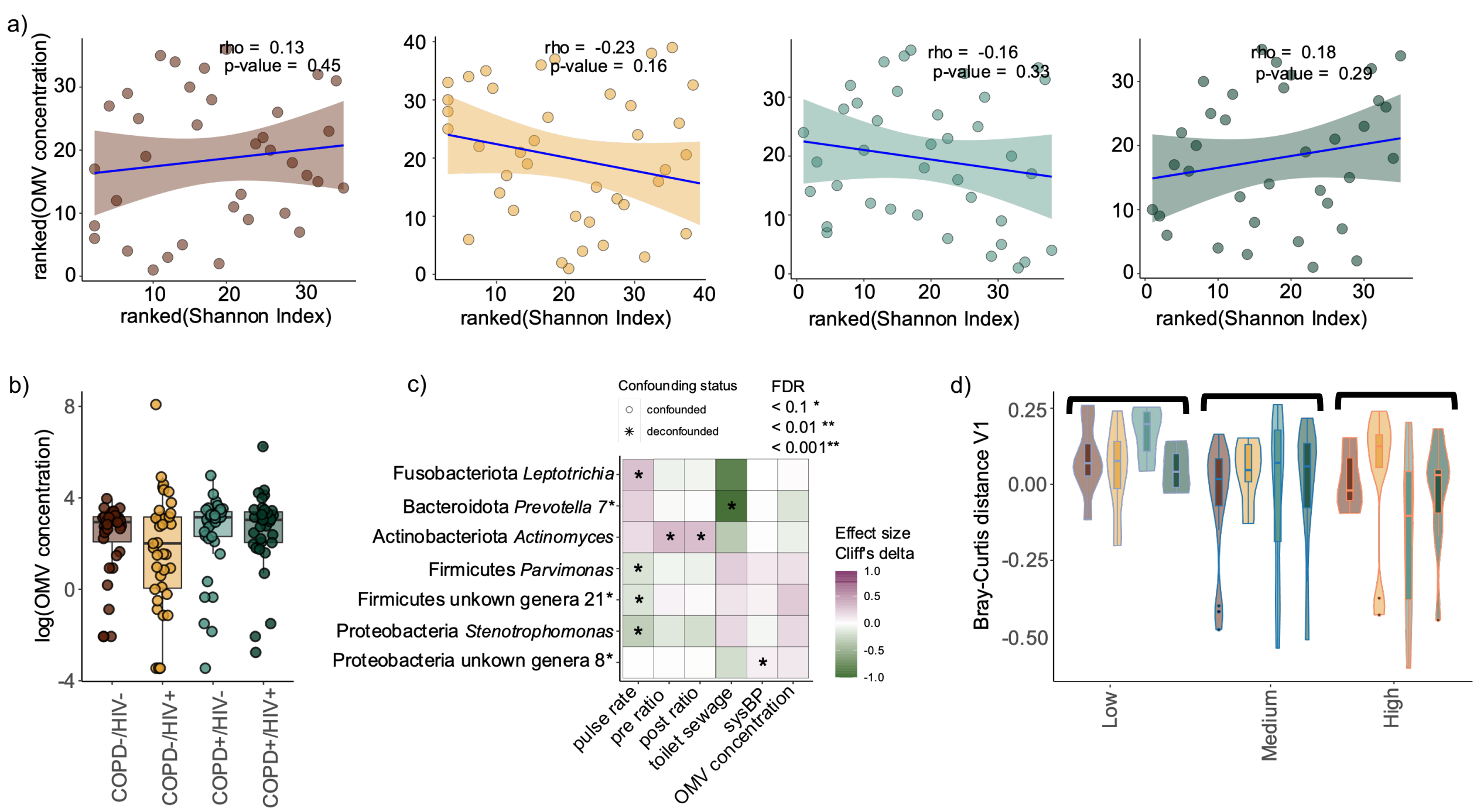

Supplementary Figure S15. Pregating and CD45+ immune-cell cluster analysis. (a) Gating strategy for the identification of single, live CD45+ cells used for subsequent CyTOF analysis. (b) Heatmap showing the expression of 12 selected markers used for clustering of CD45+ peripheral blood mononuclear cells (PBMCs). (c) UMAP representation of CD45+ PBMCs coloured according to the eight identified immune-cell clusters. (d) UMAP plots coloured by the expression intensity of selected markers used to characterize the immune-cell clusters. CyTOF analyses included *n* = 90 biologically independent participants: COPD+/HIV- (*n*=26), COPD+HIV+ (*n*=23), COPD-/HIV- (*n*=20) and COPD-/HIV+ (*n*=21). Each participant constituted the biological unit of study, and no technical replicates were treated as independent observations. Source data are provided as a Source Data file.

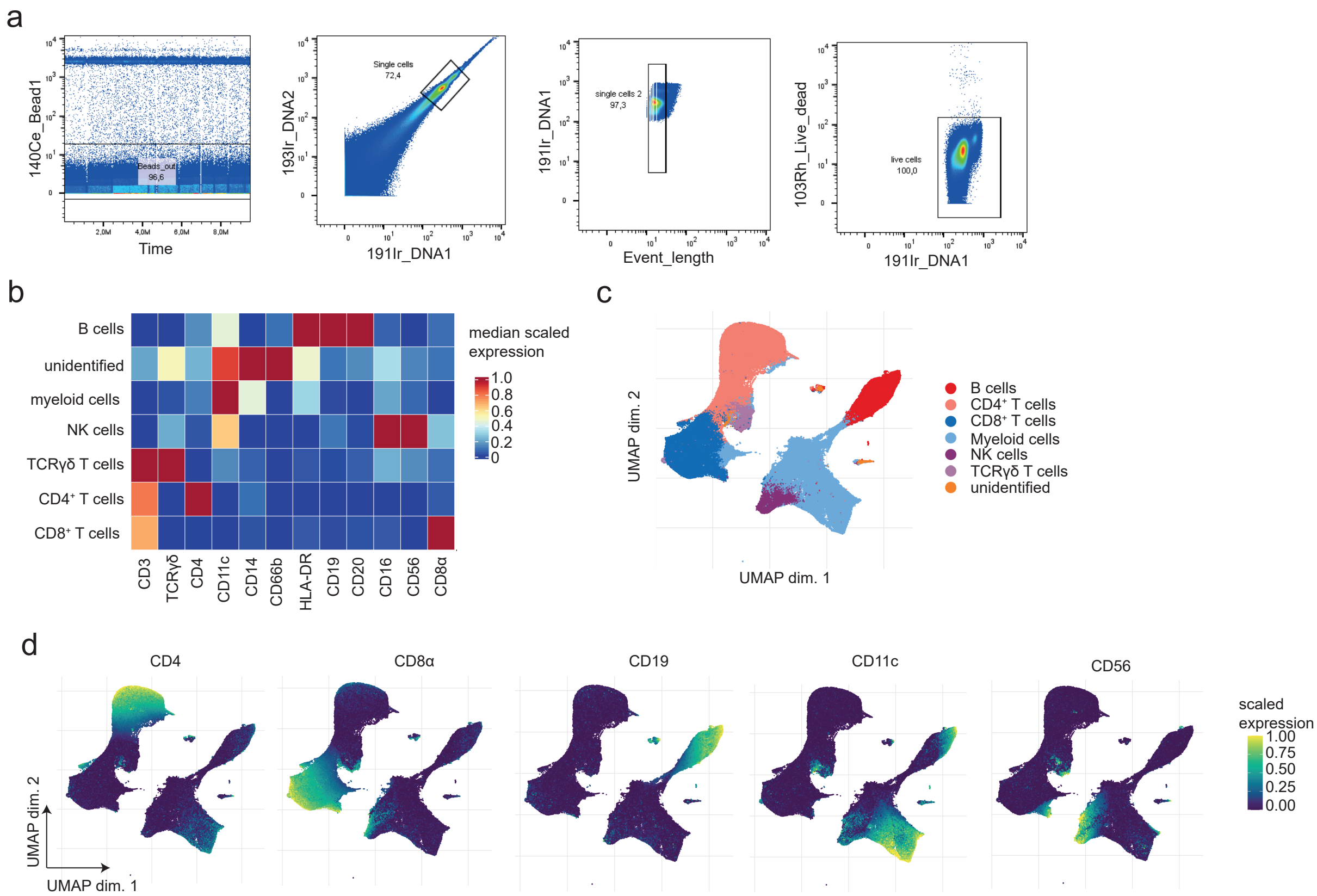

Supplementary Figure S16. Peripheral immune-cell composition according to HIV status. (a) Frequencies of major CD45^{+} immune-cell populations, including B cells, CD4^{+} T cells, CD8^{+} T cells, myeloid cells, NK cells, and TCRγδ^{+} T cells, comparing HIV-negative (n = 46) and HIV-positive (n = 44) participants. (b) Frequencies of high-resolution T-cell subsets identified by CyTOF, including CD4^{-}CD8^{-} T cells, CD4^{+} effector memory, naïve, terminal effector, and regulatory T-cell populations; CD8^{+} central memory, effector, effector memory I and II, naïve, and terminal effector cells; MAIT/NKT cells; TCRγδ^{+} T cells; and Th1-like, Th2-like, and Th17-like populations. (c) Frequencies of non-T-cell populations, including B cells, classical and non-classical monocytes, early and late NK-cell subsets, IgD^{+} B cells, myeloid dendritic cells (mDCs), plasmacytoid dendritic cells (pDCs), plasma cells, transitional monocytes, and an unidentified population. Analyses included n = 90 biologically independent participants (HIV-, n = 46; HIV+, n = 44). Each point represents one participant, which constituted the biological unit of study; no technical replicates were treated as independent observations. Boxplots show the median (centre line) and interquartile range (box), with whiskers extending to the most extreme data points within 1.5 × the interquartile range. Comparisons between HIV-negative and HIV-positive participants were performed using multivariable linear models with Benjamini-Hochberg correction for multiple testing. Significant differences are indicated by asterisks (adjusted $P < 0.05$); ns denotes non-significant comparisons. Source data are provided as a Source Data file.

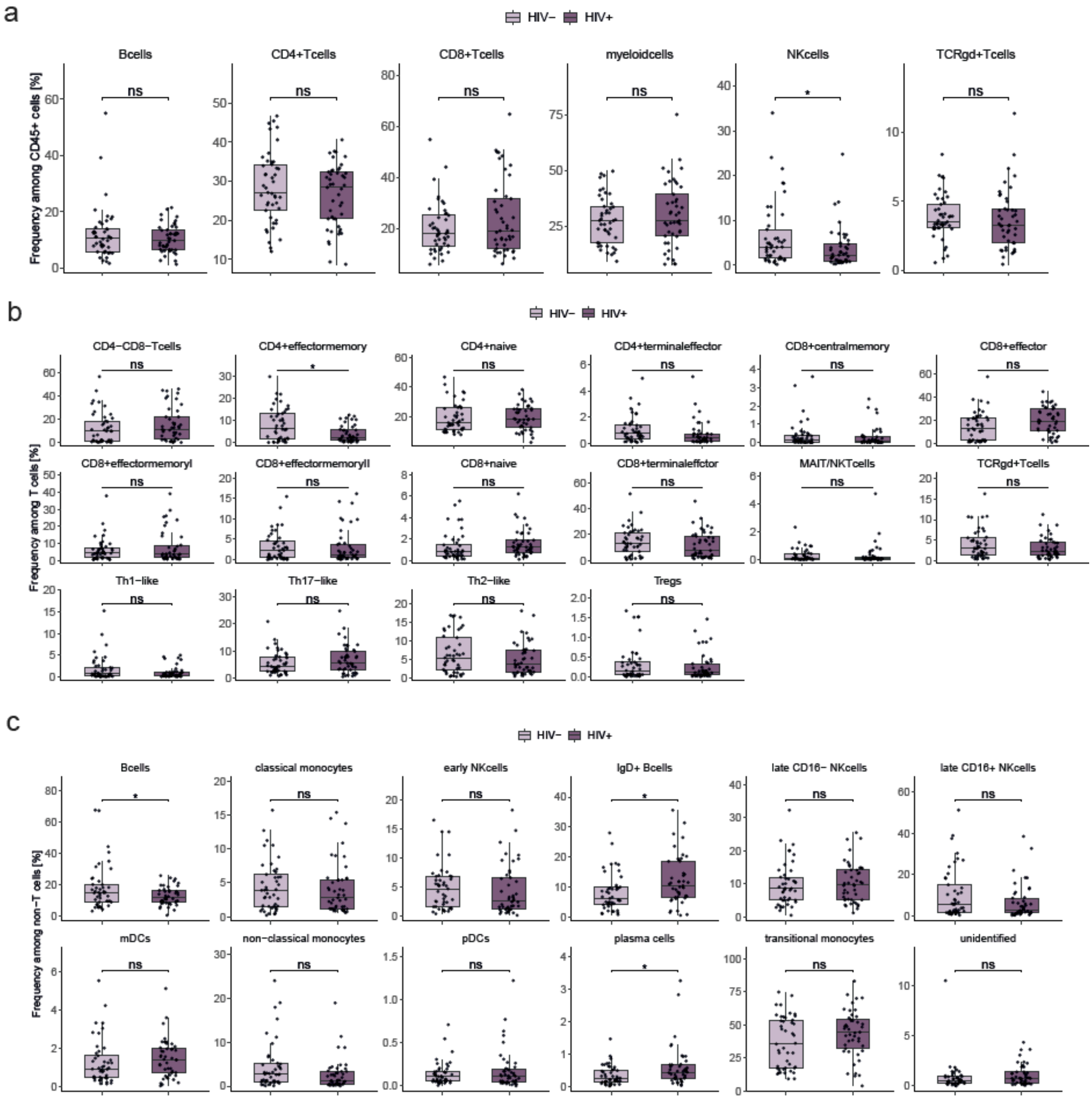

a
HIV− HIV+
Frequency among CD45+ cells [%]
Bcells
CD4+Tcells
CD8+Tcells
myeloidcells
NKcells
TCRgd+Tcells
ns
*
b
HIV− HIV+
Frequency among T cells [%]
CD4−CD8−Tcells
CD4+effectormemory
CD4+naive
CD4+terminaleffector
CD8+centralmemory
CD8+effector
CD8+effectormemoryI
CD8+effectormemoryII
CD8+naive
CD8+terminaleffctor
MAIT/NKTcells
TCRgd+Tcells
Th1−like
Th17−like
Th2−like
Tregs
c
HIV− HIV+
Frequency among non−T cells [%]
Bcells
classical monocytes
early NKcells
IgD+ Bcells
late CD16− NKcells
late CD16+ NKcells
mDCs
non−classical monocytes
pDCs
plasma cells
transitional monocytes
unidentified

Supplementary Figure S17. Peripheral immune-cell composition according to COPD status. (a) Frequencies of major $CD45^+$ immune-cell populations, including B cells, $CD4^+$ T cells, $CD8^+$ T cells, myeloid cells, NK cells, and $TCR\gamma\delta^+$ T cells, comparing COPD-negative (n = 41) and COPD-positive (n = 49) participants. (b) Frequencies of high-resolution T-cell subsets identified by CyTOF, including $CD4^-CD8^-$ T cells, $CD4^+$ effector memory, naïve, and terminal effector populations; $CD8^+$ central memory, effector, effector memory I and II, naïve, and terminal effector populations; MAIT/NKT cells; $TCR\gamma\delta^+$ T cells; Th1-like, Th2-like, Th17-like, and regulatory T-cell (Treg) populations. (c) Frequencies of non-T-cell populations, including B cells, classical and non-classical monocytes, early and late NK-cell subsets, IgD^+ B cells, myeloid dendritic cells (mDCs), plasmacytoid dendritic cells (pDCs), plasma cells, transitional monocytes, and an unidentified population. Analyses included n = 90 biologically independent participants (COPD-, n = 41; COPD+, n = 49). Each point represents one participant, which constituted the biological unit of study; no technical replicates were treated as independent observations. Boxplots show the median (centre line) and interquartile range (box), with whiskers extending to the most extreme data points within 1.5 × the interquartile range. Comparisons between COPD-negative and COPD-positive participants were performed using multivariable linear models with Benjamini-Hochberg correction for multiple testing. Significant differences are indicated by asterisks (adjusted $P < 0.05$); ns denotes non-significant comparisons. Source data are provided as a Source Data file.

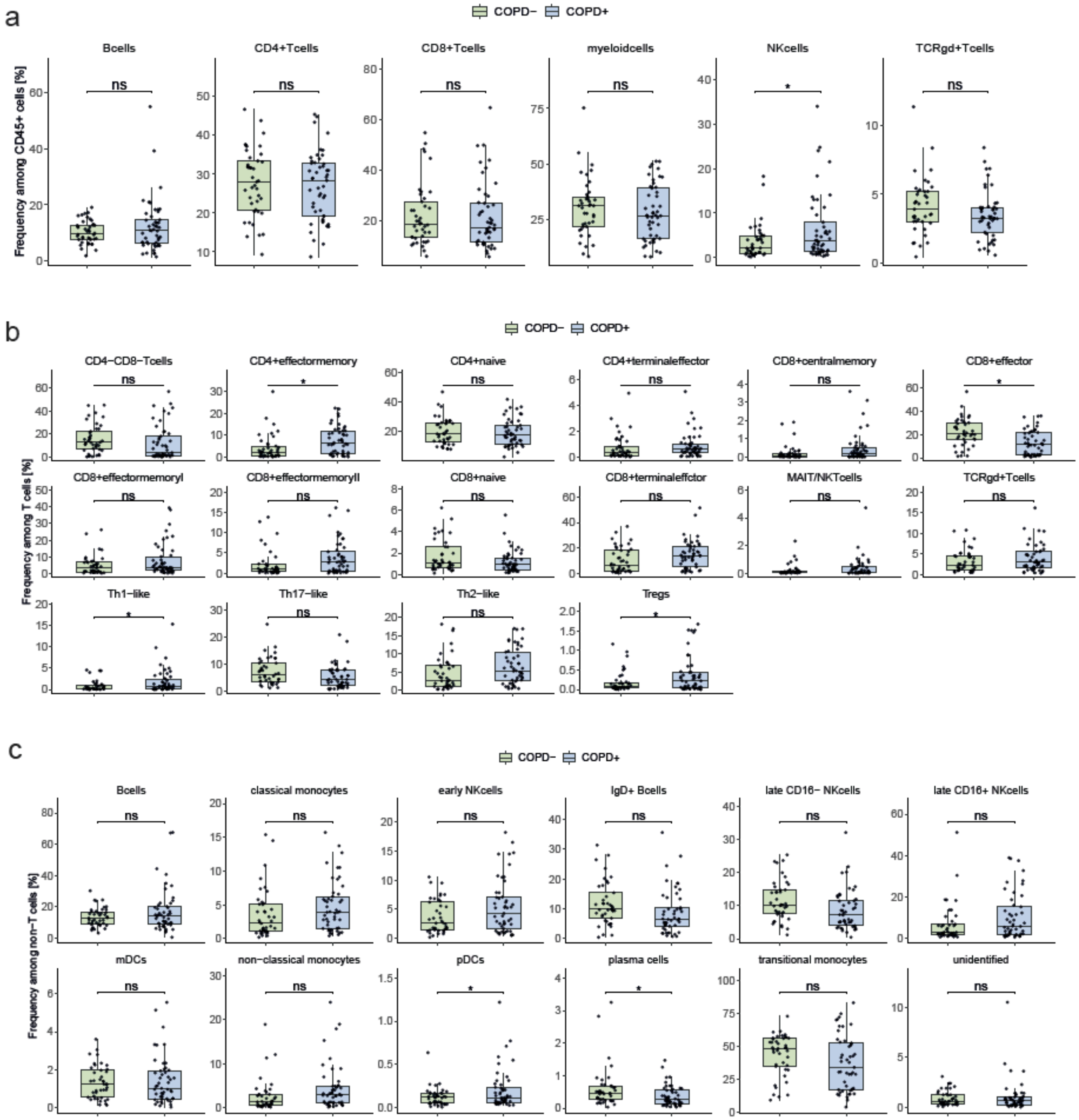
a
COPD− COPD+
Frequency among CD45+ cells [%]
Bcells
CD4+Tcells
CD8+Tcells
myeloidcells
NKcells
TCRgd+Tcells
b
COPD− COPD+
Frequency among T cells [%]
CD4−CD8−Tcells
CD4+effectormemory
CD4+naive
CD4+terminaleffector
CD8+centralmemory
CD8+effector
CD8+effectormemoryI
CD8+effectormemoryII
CD8+naive
CD8+terminaleffctor
MAIT/NKTcells
TCRgd+Tcells
Th1−like
Th17−like
Th2−like
Tregs
c
COPD− COPD+
Frequency among non−T cells [%]
Bcells
classical monocytes
early NKcells
IgD+ Bcells
late CD16− NKcells
late CD16+ NKcells
mDCs
non−classical monocytes
pDCs
plasma cells
transitional monocytes
unidentified

Supplementary Figure S18. Associations between peripheral immune-cell populations and microbiota across disease-group comparisons. Heatmaps show CyTOF immune-cell associations with clinical metadata and bacterial genera across four pairwise disease-group analyses: HIV-/COPD- (*n* = 22) versus HIV+/COPD- (*n* = 23); HIV-/COPD- (*n* = 22) versus HIV-/COPD+ (*n* = 22); HIV+/COPD- (*n* = 23) versus HIV+/COPD+ (*n* = 22); and HIV-/COPD+ (*n* = 22) versus HIV+/COPD+ (*n* = 22). Cell colours represent effect sizes, with the direction and magnitude indicated by the colour scale. Symbols denote confounder-aware associations, with circles indicating confounded associations and diamonds indicating deconfounded associations. Statistical significance is indicated as $P < 0.05$ (*), $P < 0.01$ (**), and $P < 0.001$ (***). Microbial genera were excluded as potential confounders. Participants constituted the biological unit of study; no technical replicates were treated as independent observations. Source data are provided as a Source Data file.

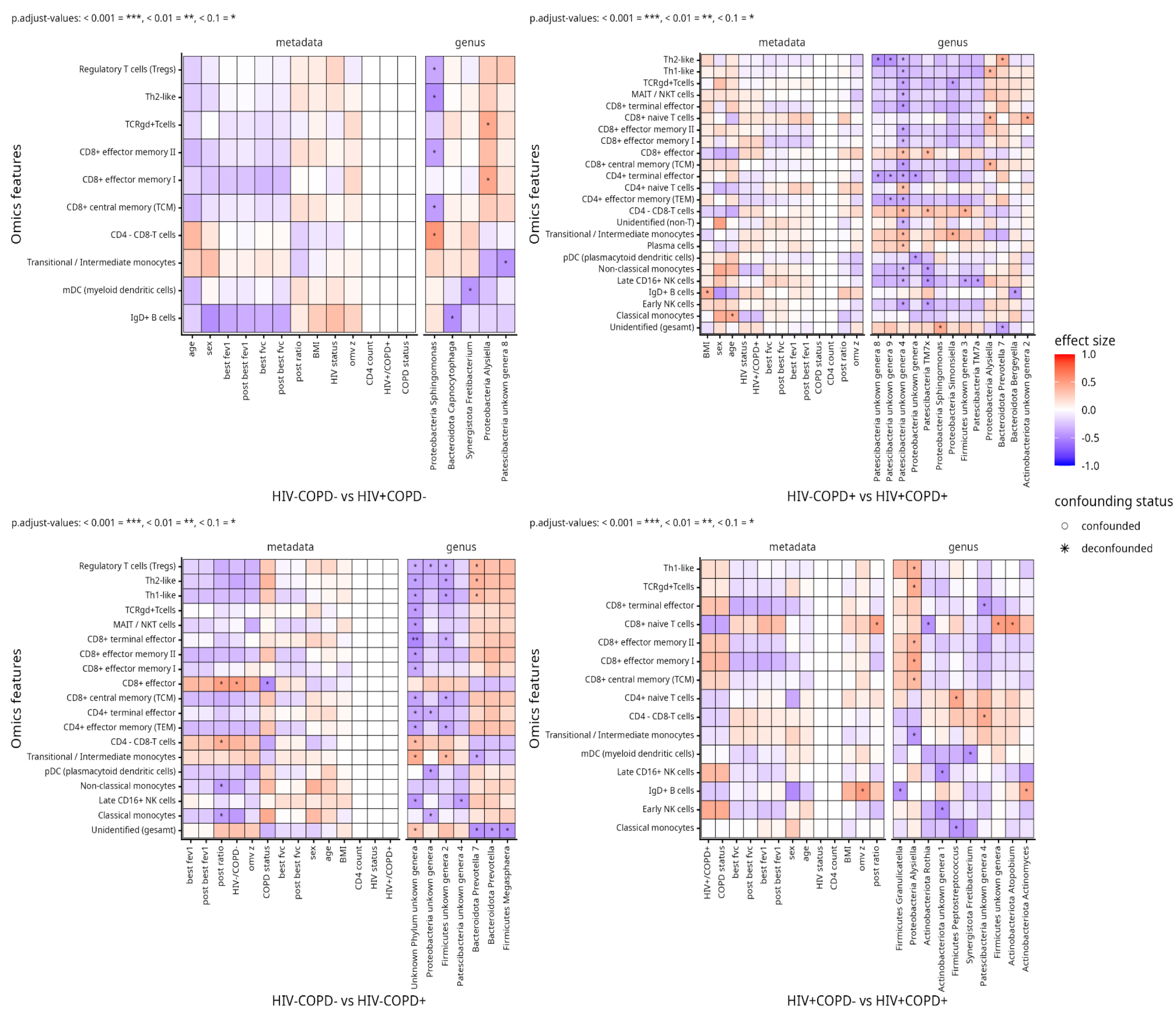

Supplementary Figure S19. Microbial features align with coordinated airway transcriptional programmes. Heatmap showing associations between filtered airway bacterial genera and host gene-expression features in induced sputum. Microbial genera were filtered for prevalence and abundance and centred log-ratio transformed before analysis. RNA features were prefiltered for associations with dual HIV/COPD status, after which microbiome-transcript associations were tested while accounting for HIV status, COPD status, lung function, age, sex, and BMI. Analyses included *n* = 80 biologically independent participants: HIV-/COPD− (*n* = 19), HIV+/COPD− (*n* = 23), HIV−/COPD+ (*n* = 20), and HIV+/COPD+ (*n* = 18). To provide a complete visualization of the significant microbiome-host associations, all host transcripts with at least one microbial association meeting Benjamini-Hochberg false discovery rate (BH-FDR) < 0.10 are displayed across 17 consecutive heatmap panels. Microbial features are shown as rows and host transcripts as columns; the same microbial features are retained across panels to facilitate comparison of transcriptional association patterns. Microbial taxa are labelled at the genus level where available; taxa lacking genus-level assignment are displayed at the highest informative taxonomic level. Colours indicate effect sizes; open circles denote associations attenuated after adjustment for potential confounders, whereas asterisks denote associations remaining significant after adjustment (FDR < 0.10, *FDR < 0.01, **FDR < 0.001; Benjamini-Hochberg correction). Each participant constitutes the biological unit of study; no technical replicates were treated as independent observations. Host transcripts are ordered reproducibly according to the number and strength of significant microbial associations and distributed sequentially across the 17 panels for readability. Source data are provided as a Source Data file.

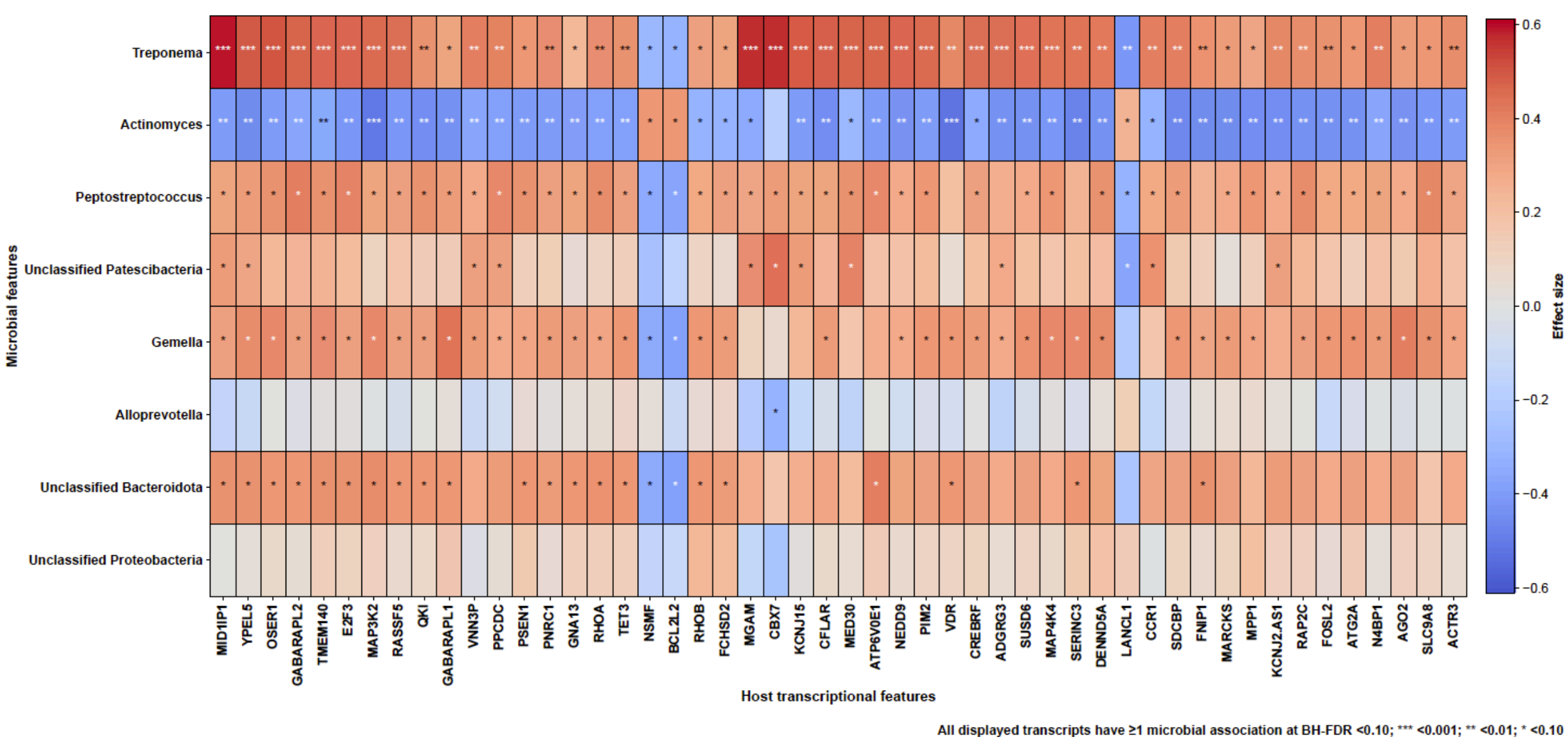

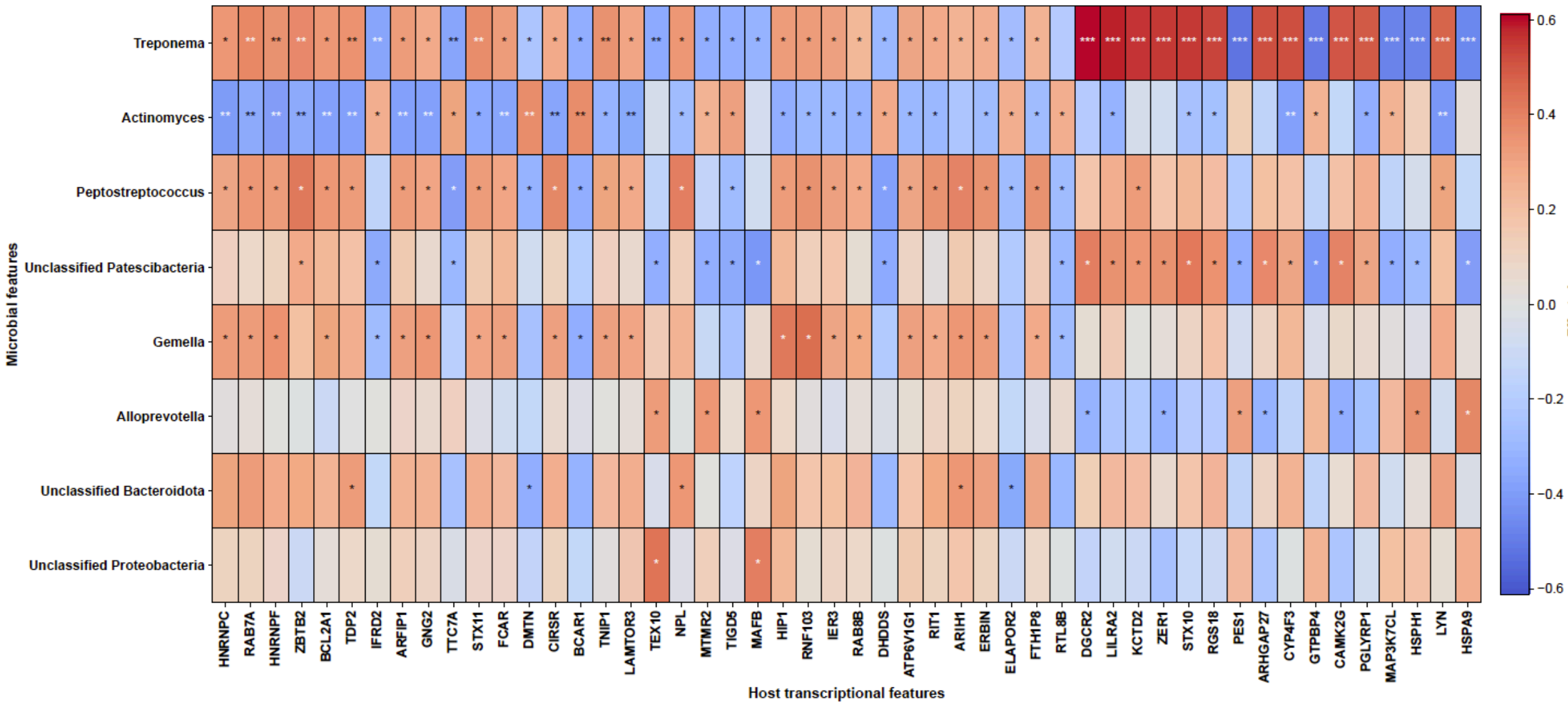

All displayed transcripts have ≥1 microbial association at BH-FDR <0.10; *** <0.001; ** <0.01; * <0.10

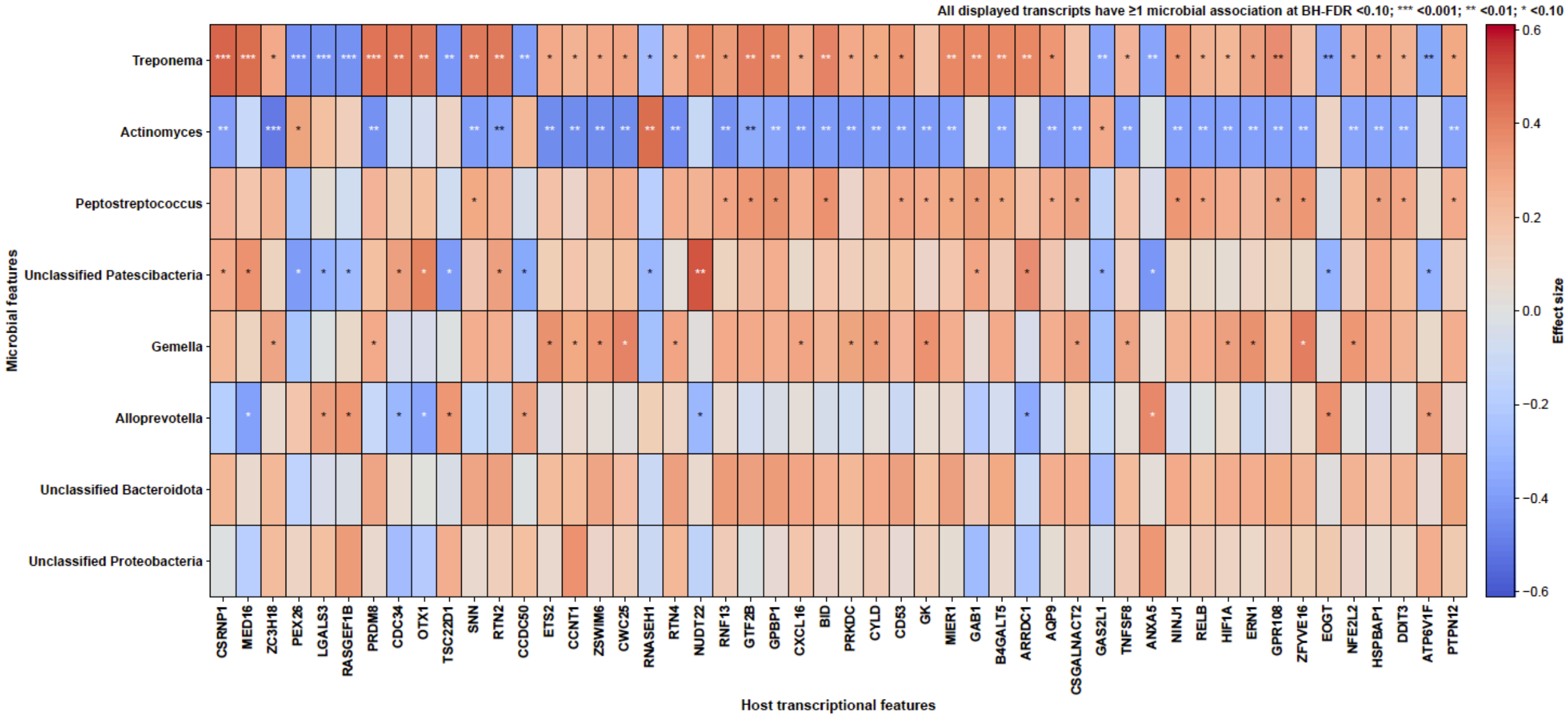

All displayed transcripts have ≥1 microbial association at BH-FDR <0.10; *** <0.001; ** <0.01; * <0.10

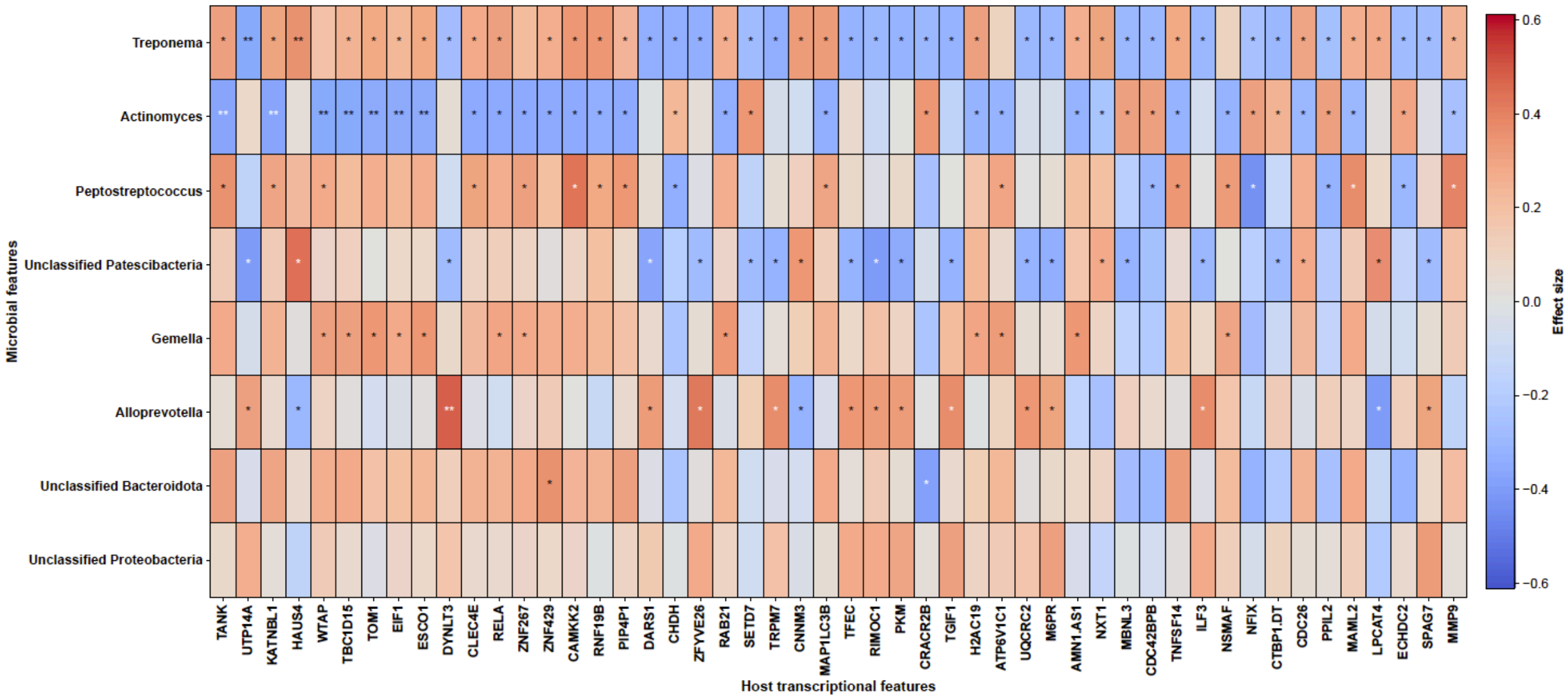

All displayed transcripts have ≥1 microbial association at BH-FDR <0.10; *** <0.001; ** <0.01; * <0.10

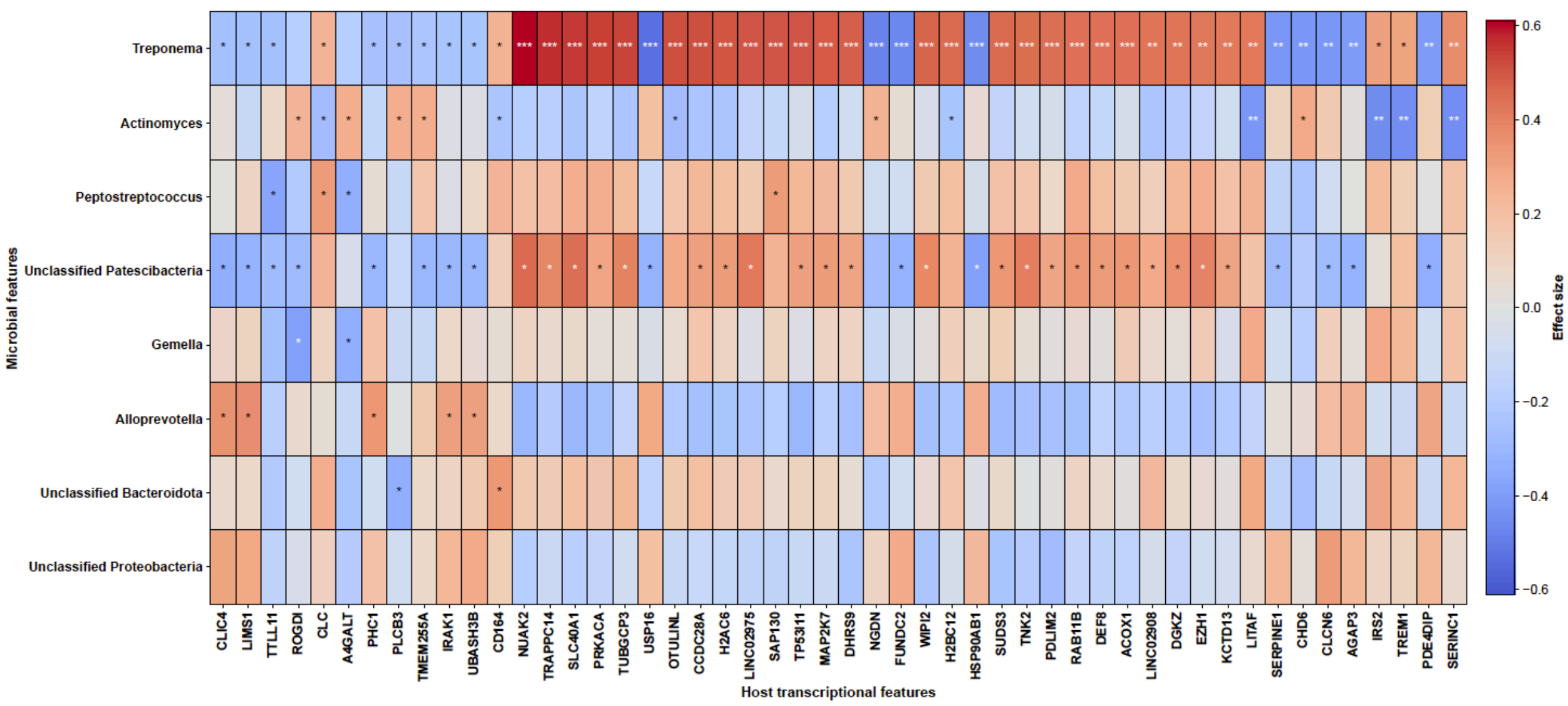

All displayed transcripts have ≥1 microbial association at BH-FDR <0.10; *** <0.001; ** <0.01; * <0.10

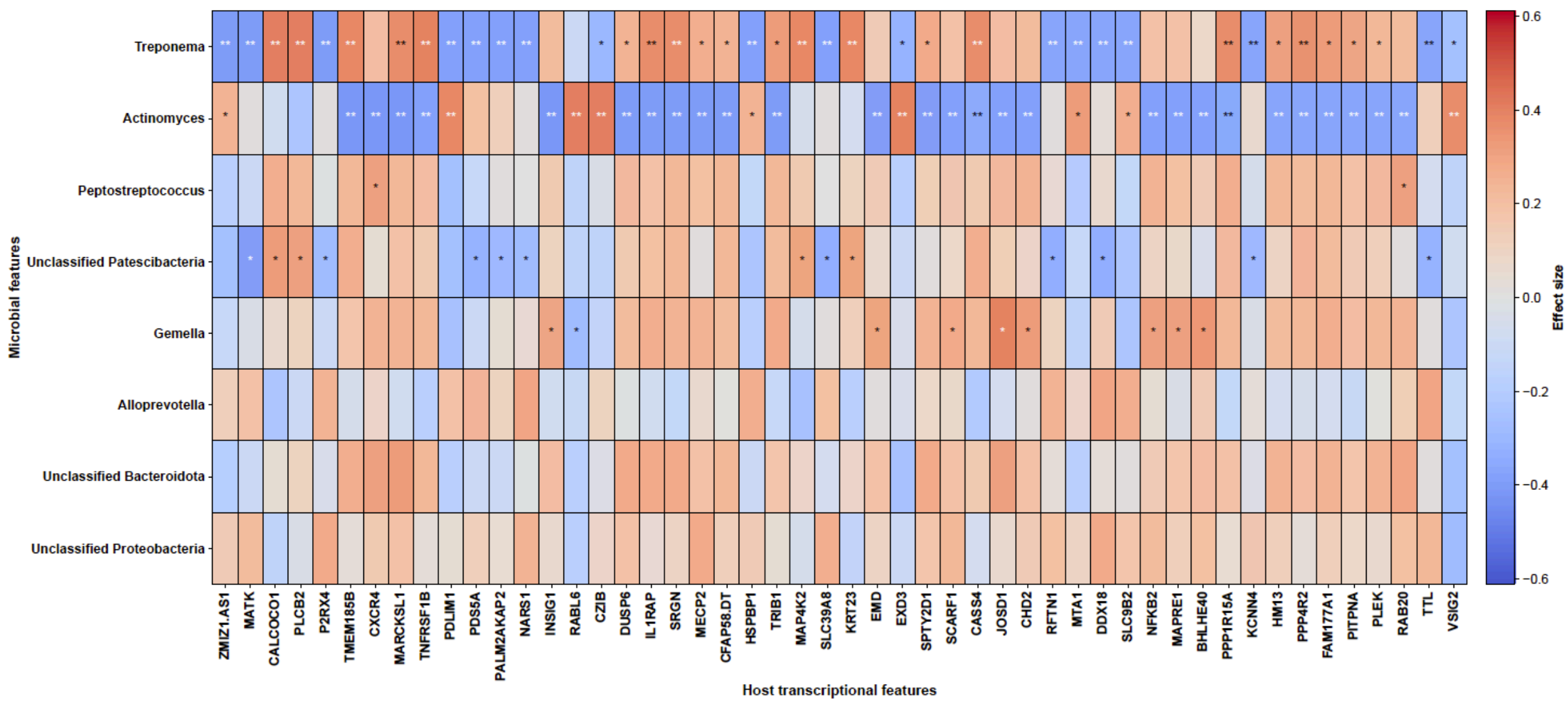

All displayed transcripts have ≥1 microbial association at BH-FDR <0.10; *** <0.001; ** <0.01; * <0.10

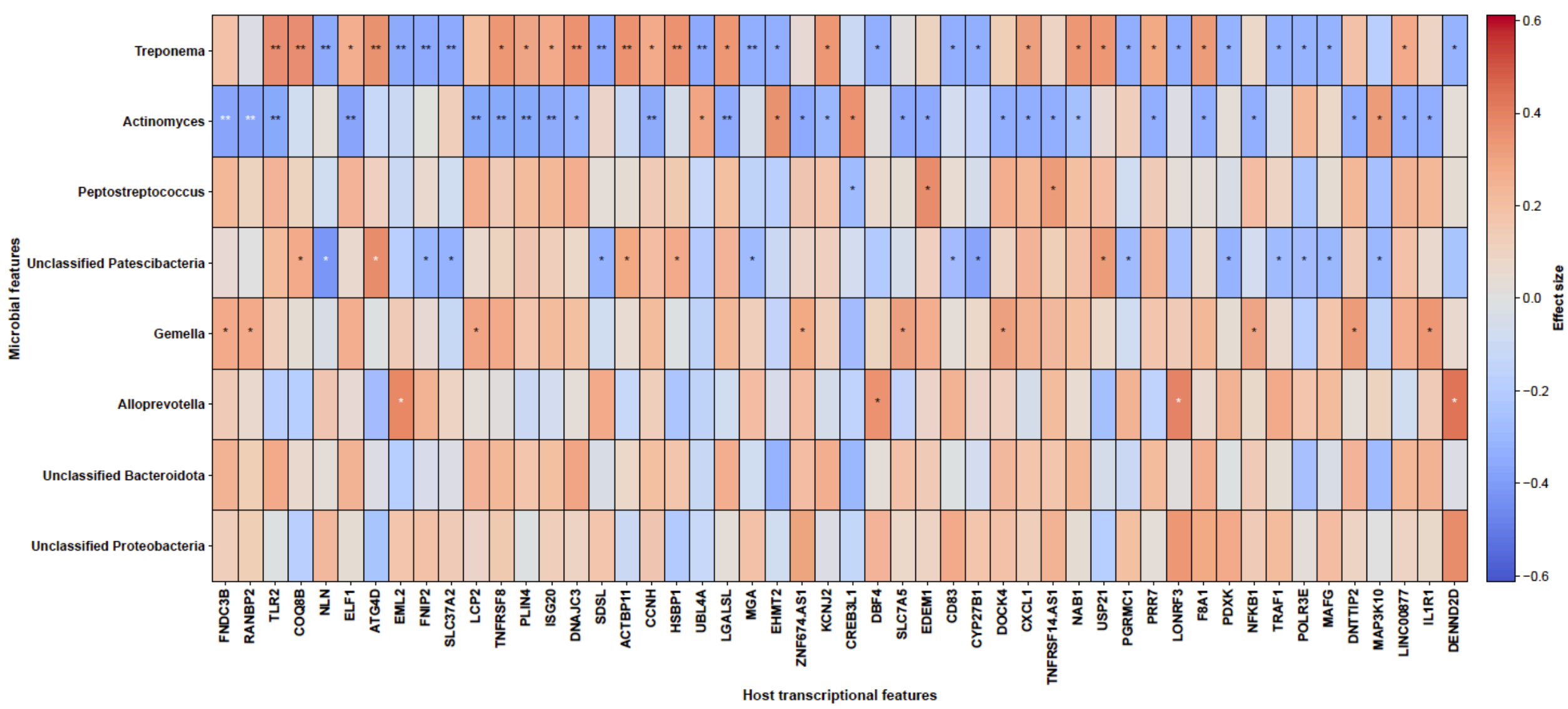

All displayed transcripts have ≥1 microbial association at BH-FDR <0.10; *** <0.001; ** <0.01; * <0.10

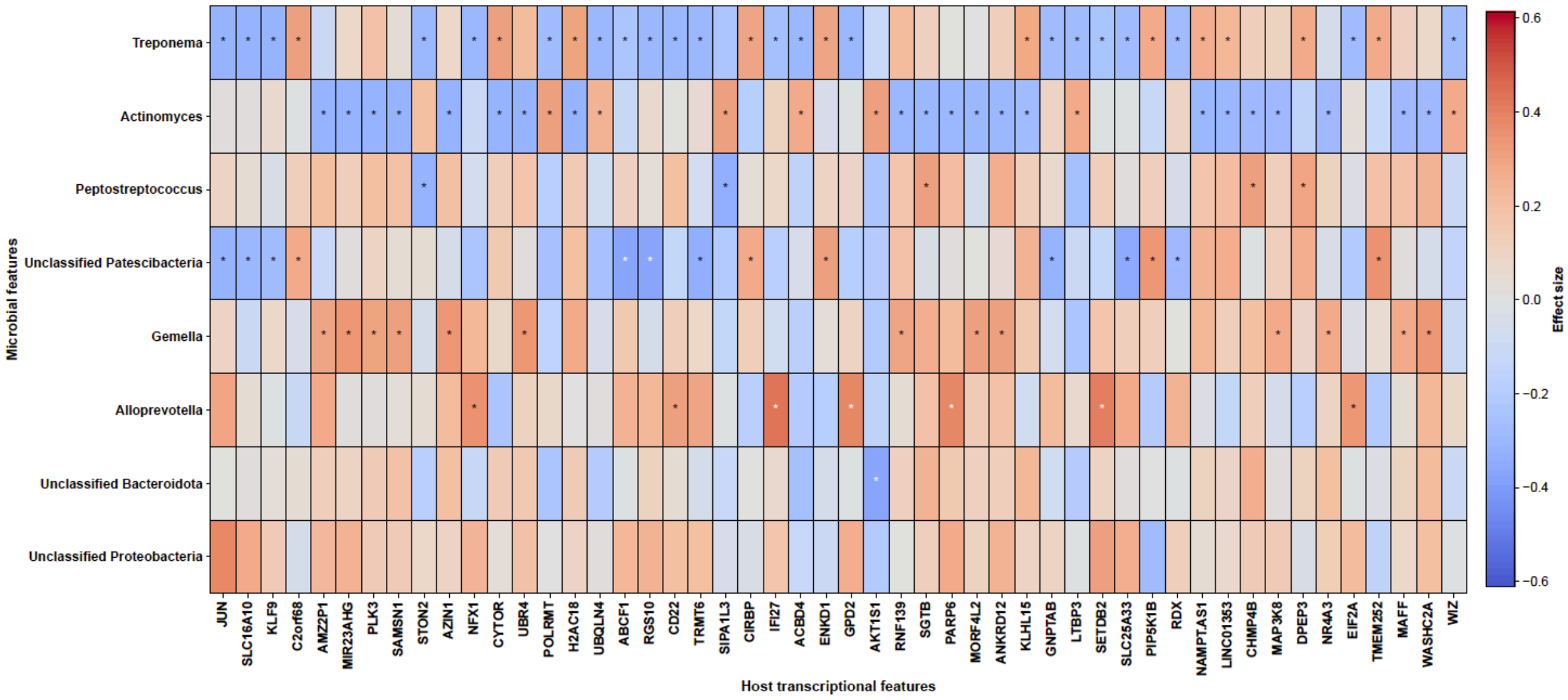

All displayed transcripts have ≥1 microbial association at BH-FDR <0.10; *** <0.001; ** <0.01; * <0.10

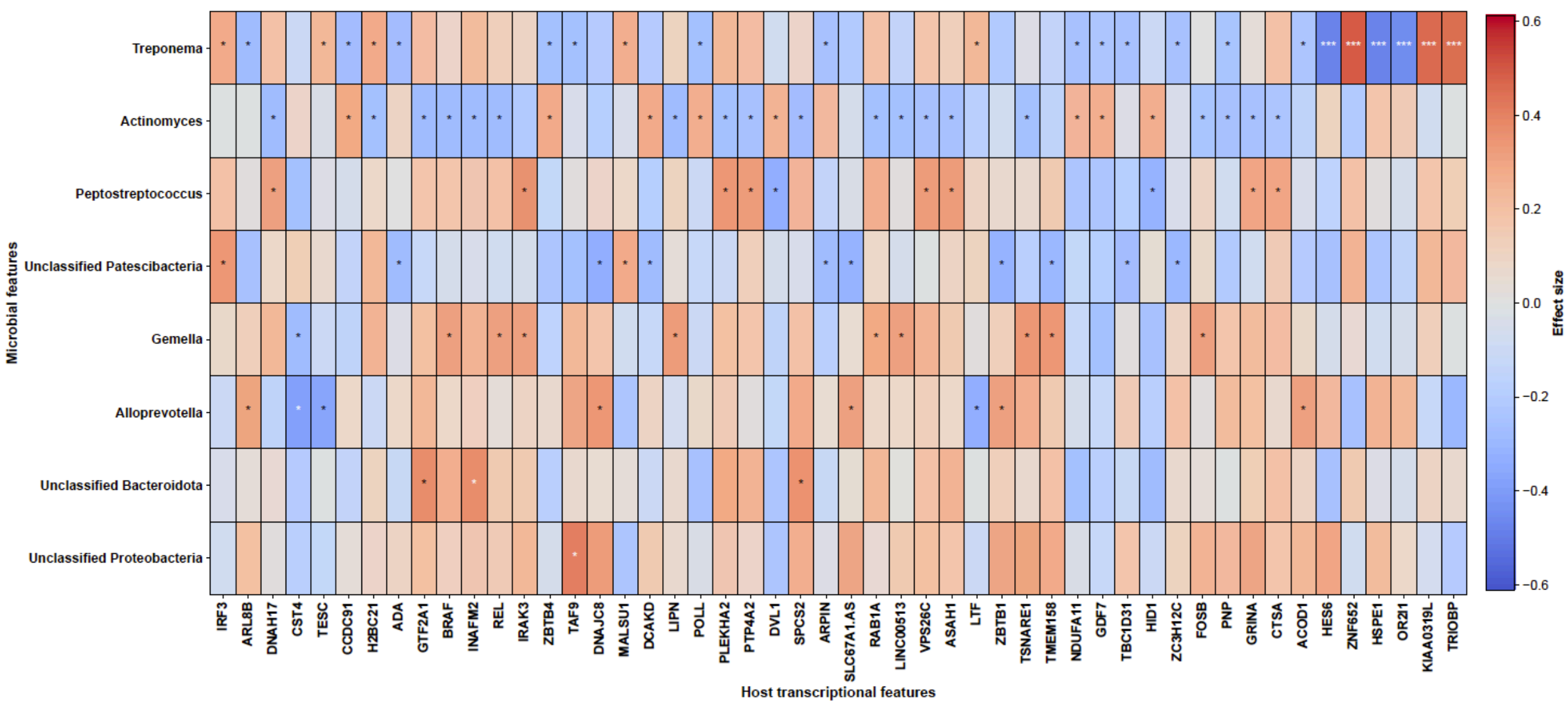

All displayed transcripts have ≥1 microbial association at BH-FDR <0.10; *** <0.001; ** <0.01; * <0.10

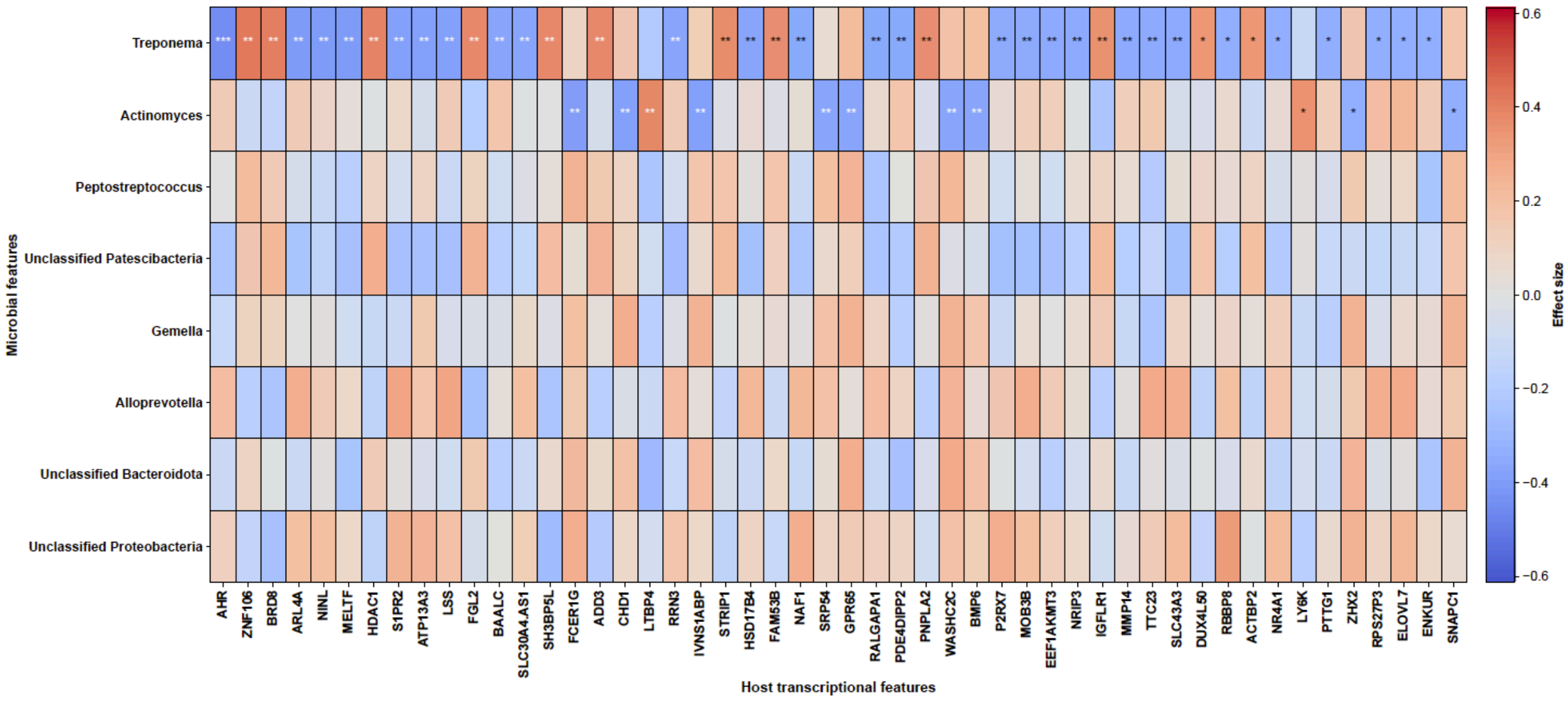

All displayed transcripts have ≥1 microbial association at BH-FDR <0.10; *** <0.001; ** <0.01; * <0.10

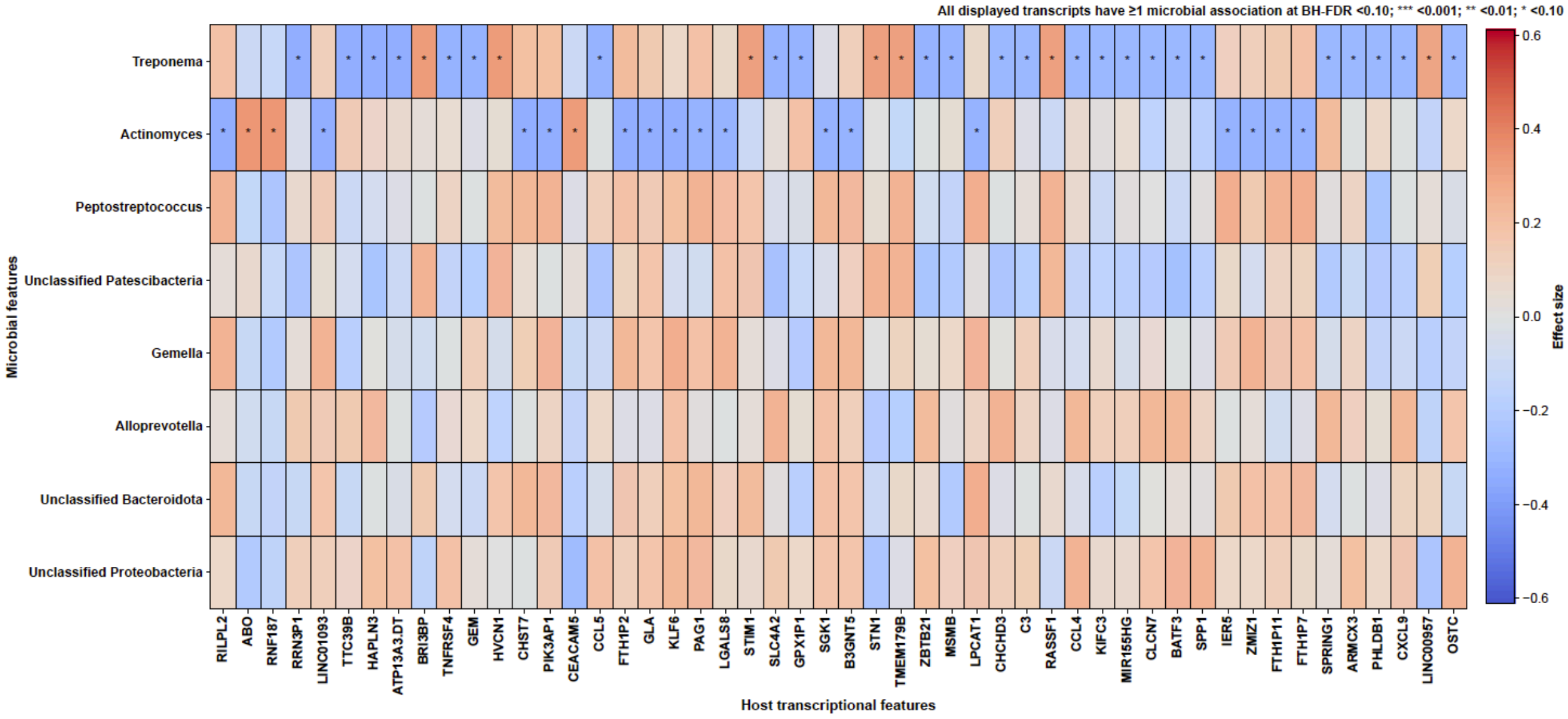

All displayed transcripts have ≥1 microbial association at BH-FDR <0.10; *** <0.001; ** <0.01; * <0.10

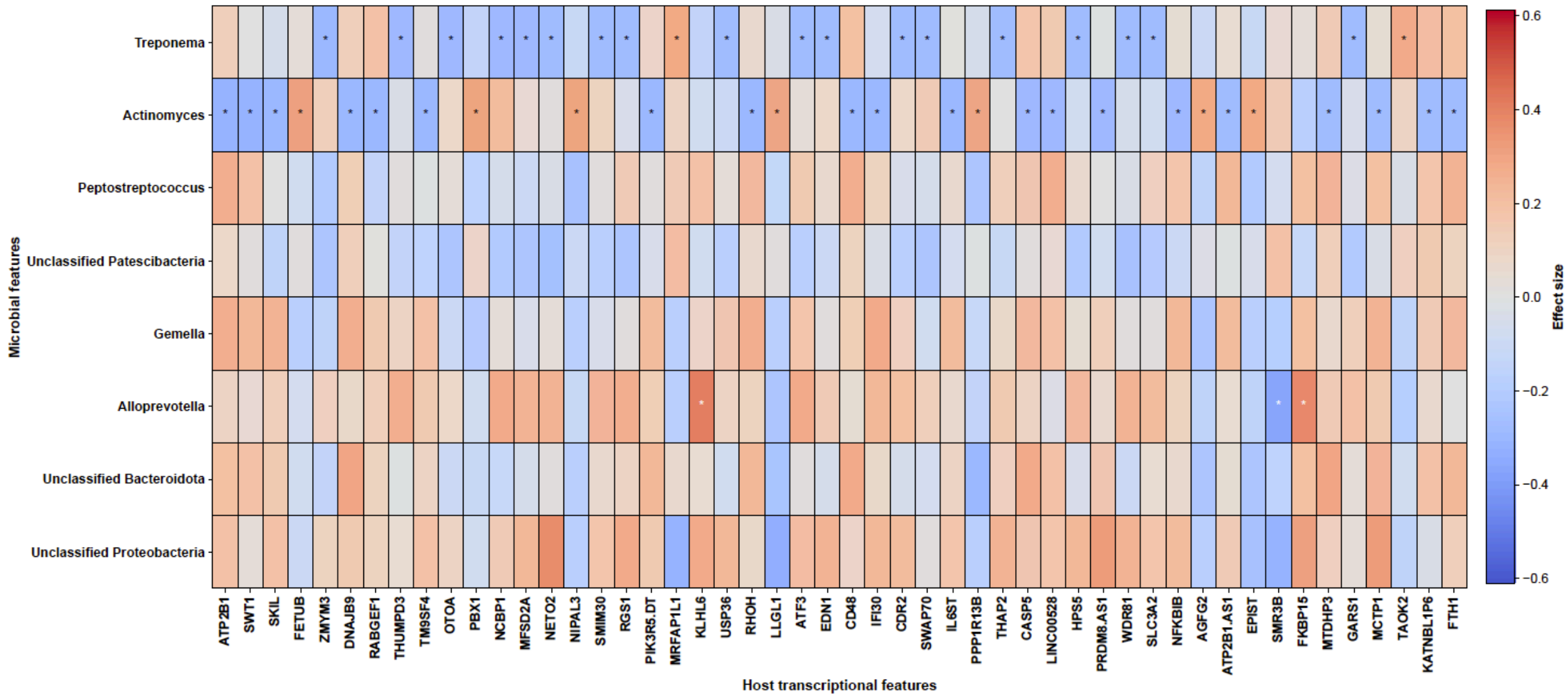

All displayed transcripts have ≥1 microbial association at BH-FDR <0.10; *** <0.001; ** <0.01; * <0.10

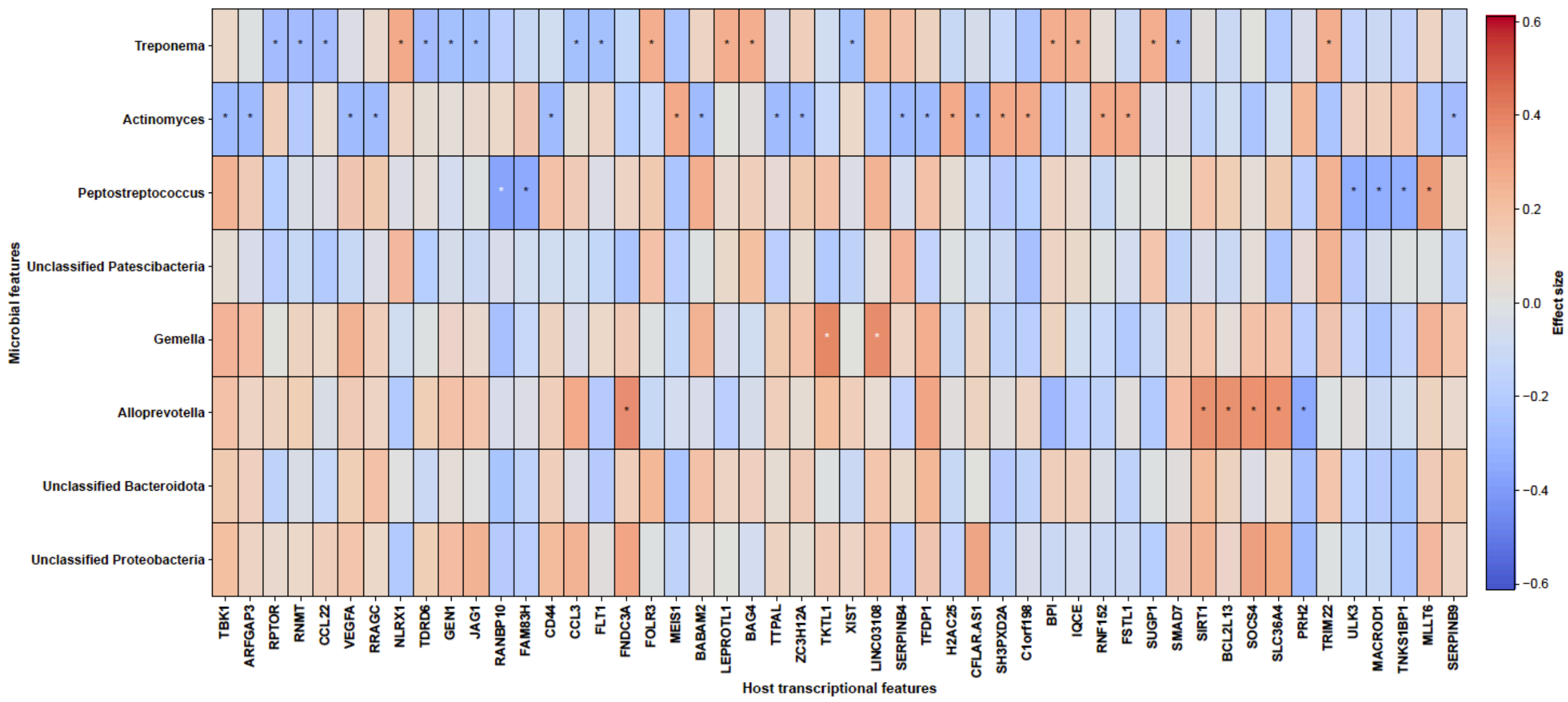

All displayed transcripts have ≥1 microbial association at BH-FDR <0.10; *** <0.001; ** <0.01; * <0.10

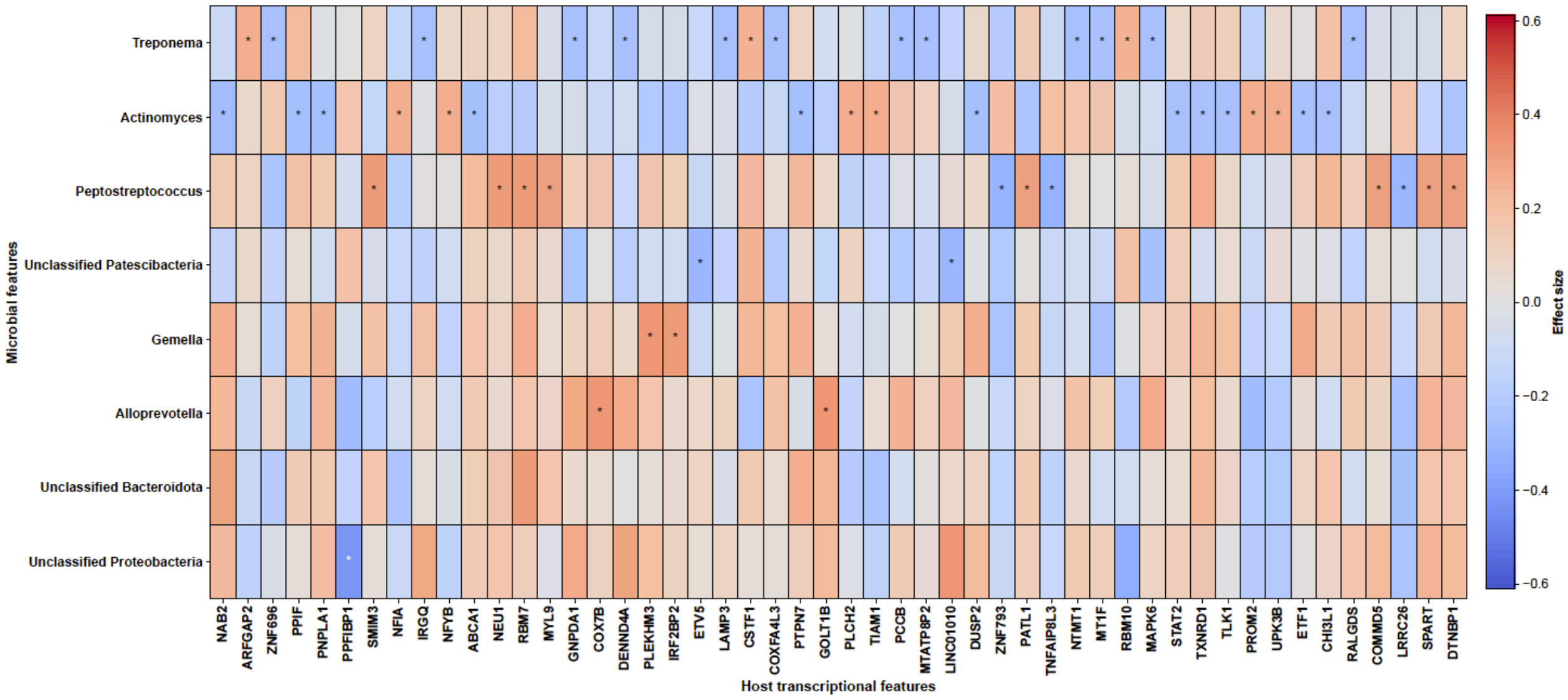

All displayed transcripts have ≥1 microbial association at BH-FDR <0.10; *** <0.001; ** <0.01; * <0.10

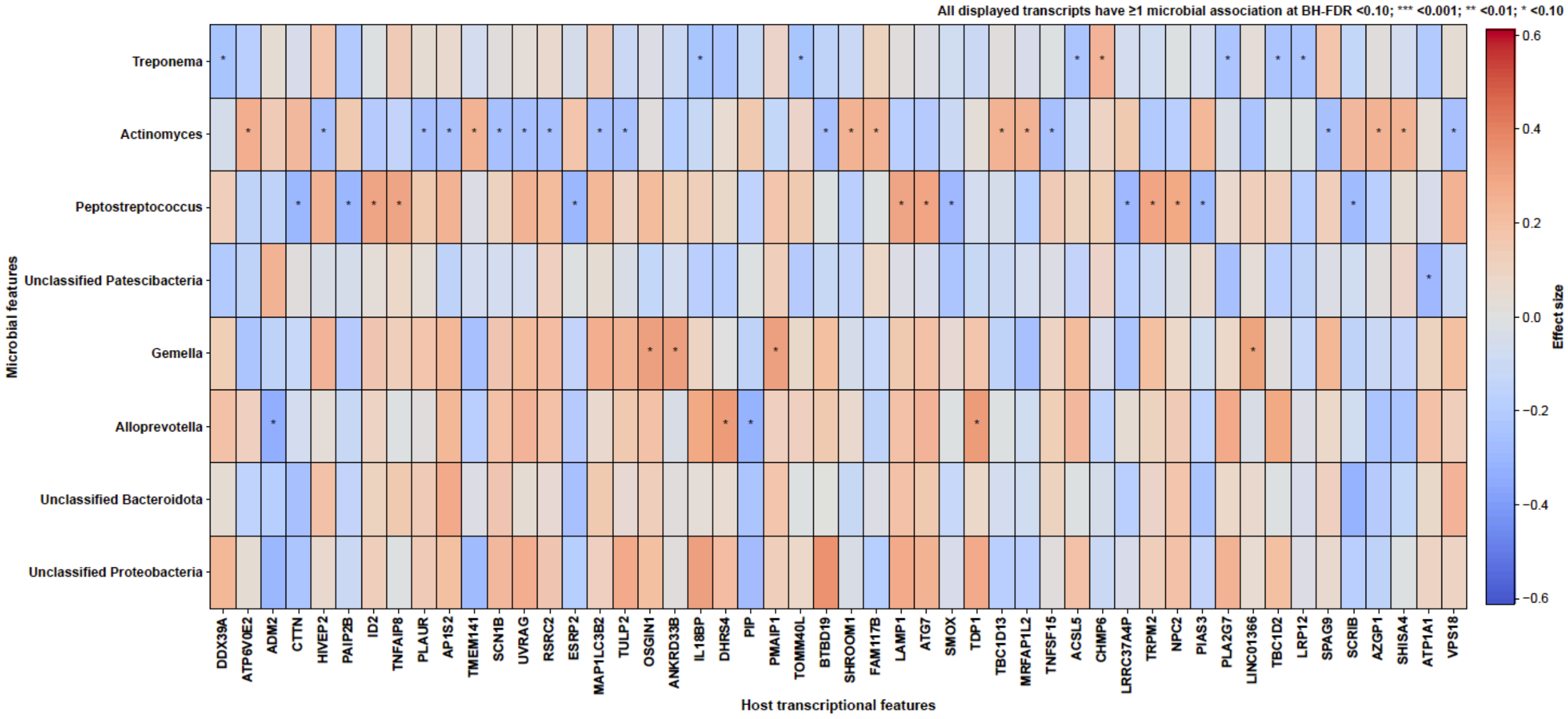

All displayed transcripts have ≥1 microbial association at BH-FDR <0.10; *** <0.001; ** <0.01; * <0.10

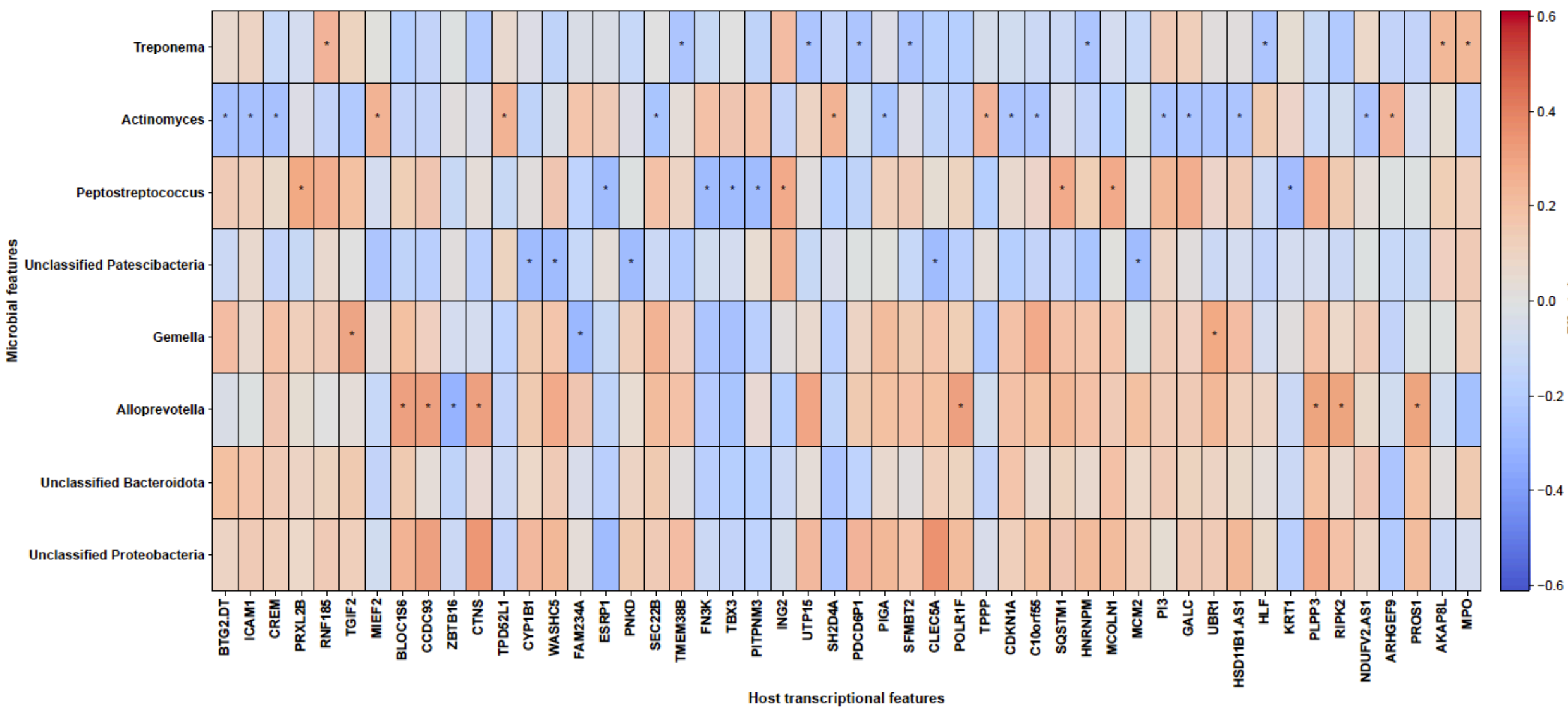

All displayed transcripts have ≥1 microbial association at BH-FDR <0.10; *** <0.001; ** <0.01; * <0.10

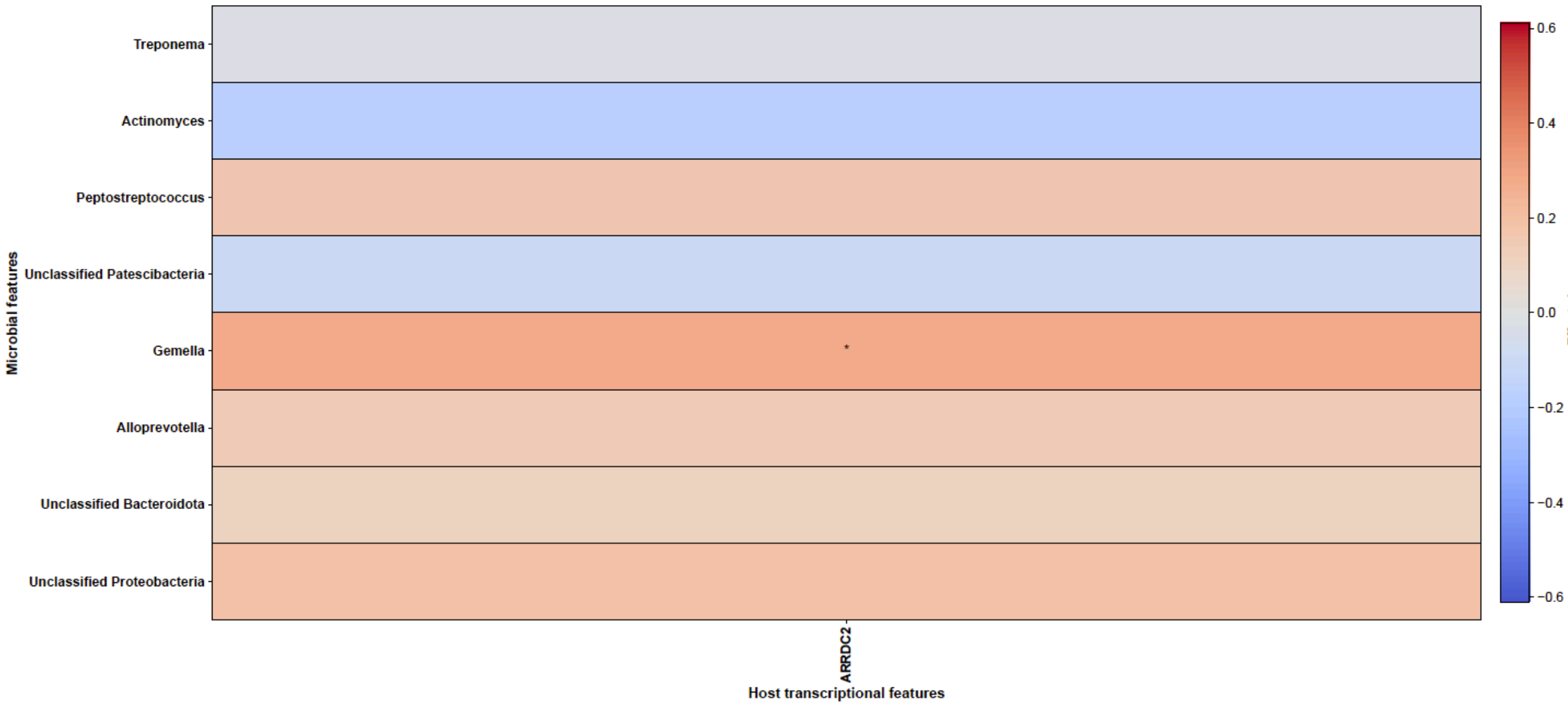

All displayed transcripts have ≥1 microbial association at BH-FDR <0.10; *** <0.001; ** <0.01; * <0.10

Supplementary Figure S20: Bacterial-derived OMVs on airway microenvironment and gene expression. (a) Shannon diversity index comparison across OMV concentration groups (high, low, medium). Box plots show no significant difference between groups (Wilcox test, BH-FDR adjusted). (b) Principal Component Analysis (PCA) of gene expression profiles. Each point represents a sample, colored by OMV group (high, low, medium). PC1 accounts for 31% variance, PC2 for 19% variance. PERMANOVA test performed to assess group differences. (c) Volcano plot of differential gene expression analysis. X-axis: Log2 Fold Change; Y-axis: -Log10(adjusted P). Red points indicate significantly differentially expressed genes (adjusted p-value < 0.1); grey points are non-significant. Significant genes are labeled. (d) Gene Ontology enrichment analysis of differentially expressed genes. A bar plot shows enriched pathways on the Y-axis and -log10(q-value) on the X-axis. Color intensity indicates significance (BH-FDR adjusted q-value). Top enriched pathways include "zinc ion binding," "transition metal ion binding," and "metal ion binding." (e) Expression levels of significant differentially expressed genes across OMV groups. Boxplots for genes BOLA2B, CNNM3, ENSG00000240103, ENSG00000256148, and MTCYBP18. Colors represent OMV groups (High, Low, Medium). Asterisks indicate significance levels (* $p < 0.1$, ** $p < 0.01$, *** $p < 0.001$, BH-FDR adjusted). Analyses included $n = 80$ biologically independent participants. Each participant constituted the biological unit of study; no technical replicates were treated as independent observations. Source data are provided as a Source Data file.

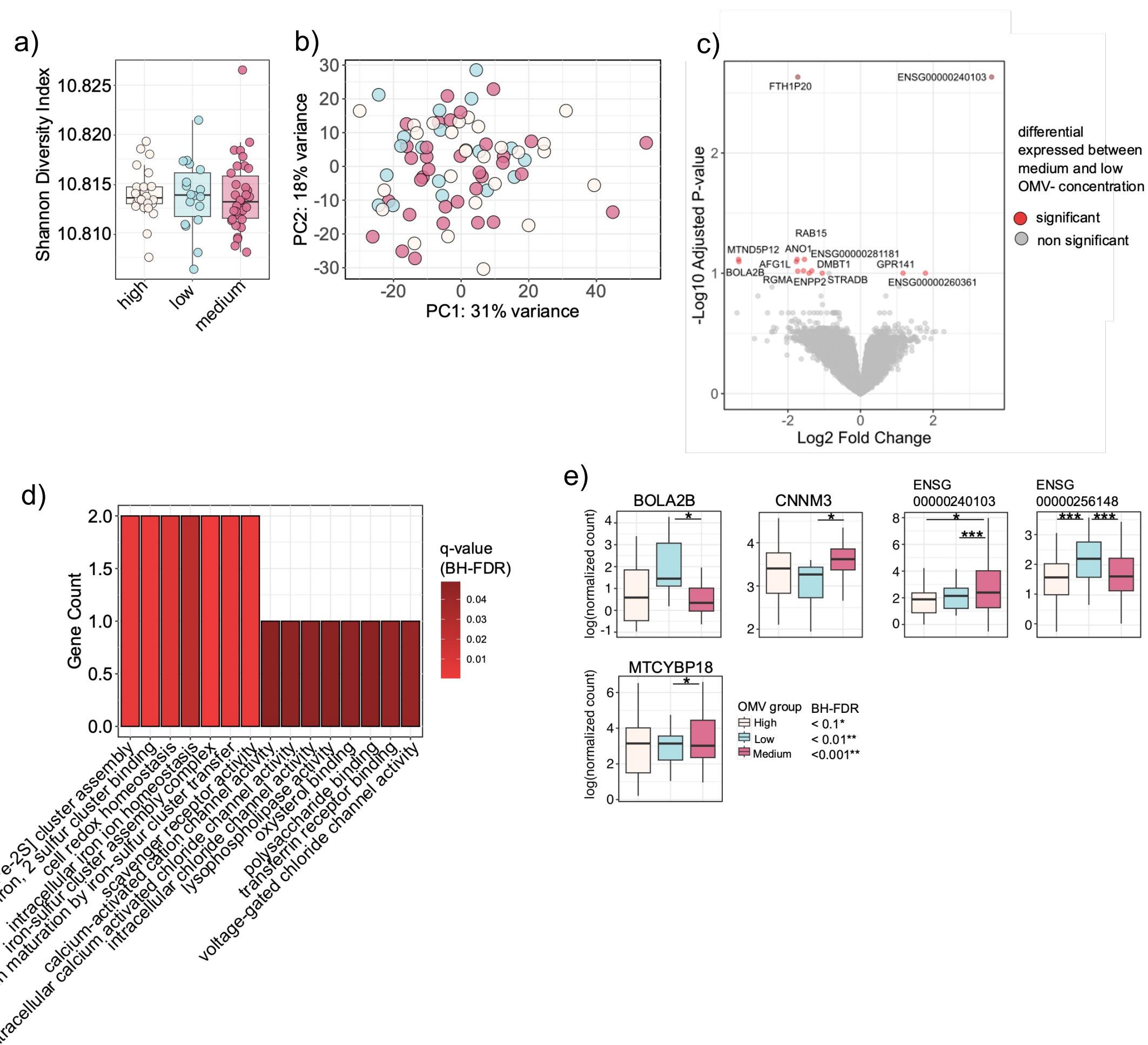

Supplementary Figure S21. BAL single-cell reference for airway cell-type deconvolution. Relative cellular composition of the bronchoalveolar lavage (BAL) single-cell RNA-sequencing reference generated from *n* = 4 biologically independent participants, comprising 5,205 nuclei after quality control and annotated into 12 major cell populations. Stacked bars show the relative proportion of annotated cell populations within each BAL sample, with each bar representing one participant. Participants constituted the biological units of study; individual nuclei represent cells sampled within participants and were not treated as independent biological replicates. No technical replicates were treated as independent observations. Source data are provided as a Source Data file.

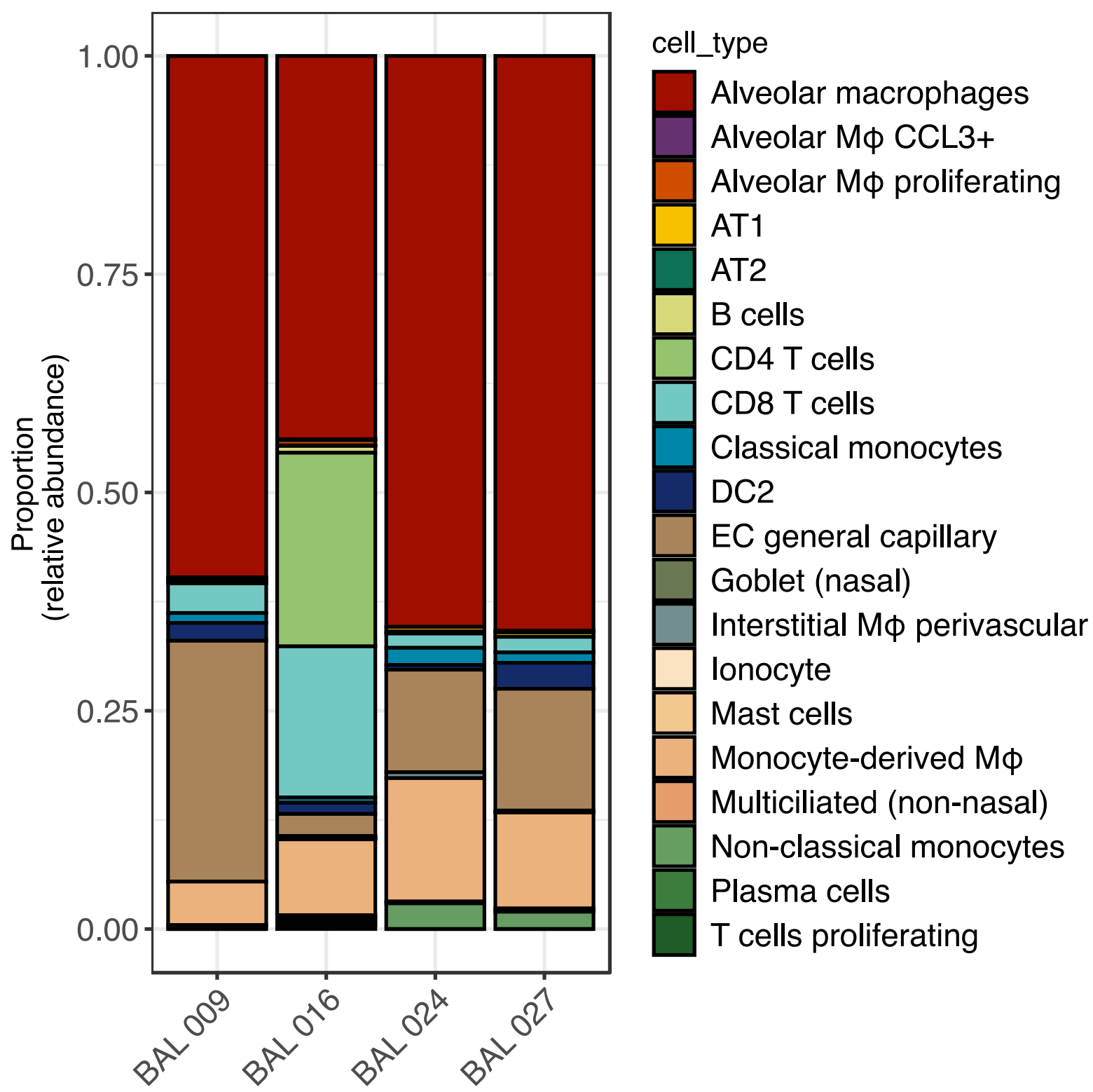

Supplementary Figure S22. BAL single-cell-guided deconvolution of induced-sputum cellular composition according to HIV/COPD status and OMV burden. MuSiC-derived proportions of 12 major airway cell populations estimated using the cohort-derived BAL single-cell reference. Top, estimated cell-type proportions across the four HIV/COPD groups (COPD-/HIV-, COPD+/HIV-, COPD−/HIV+, and COPD+/HIV+). Bottom estimated cell-type proportions across quartiles of standardized outer membrane vesicle (OMV) concentration. Analyses included *n* = 48 biologically independent participants. Boxplots show the median (centre line), interquartile range (box), and whiskers extending to the most extreme data points within 1.5 × the interquartile range. Differences across the four HIV/COPD groups were assessed independently for each cell population using Kruskal-Wallis tests, with Benjamini-Hochberg correction for multiple testing across the 12 cell populations. Each participant constituted the biological unit of study; deconvolution-derived cell proportions are model-based estimates, and no technical replicates were treated as independent observations. Source data are provided as a Source Data file.

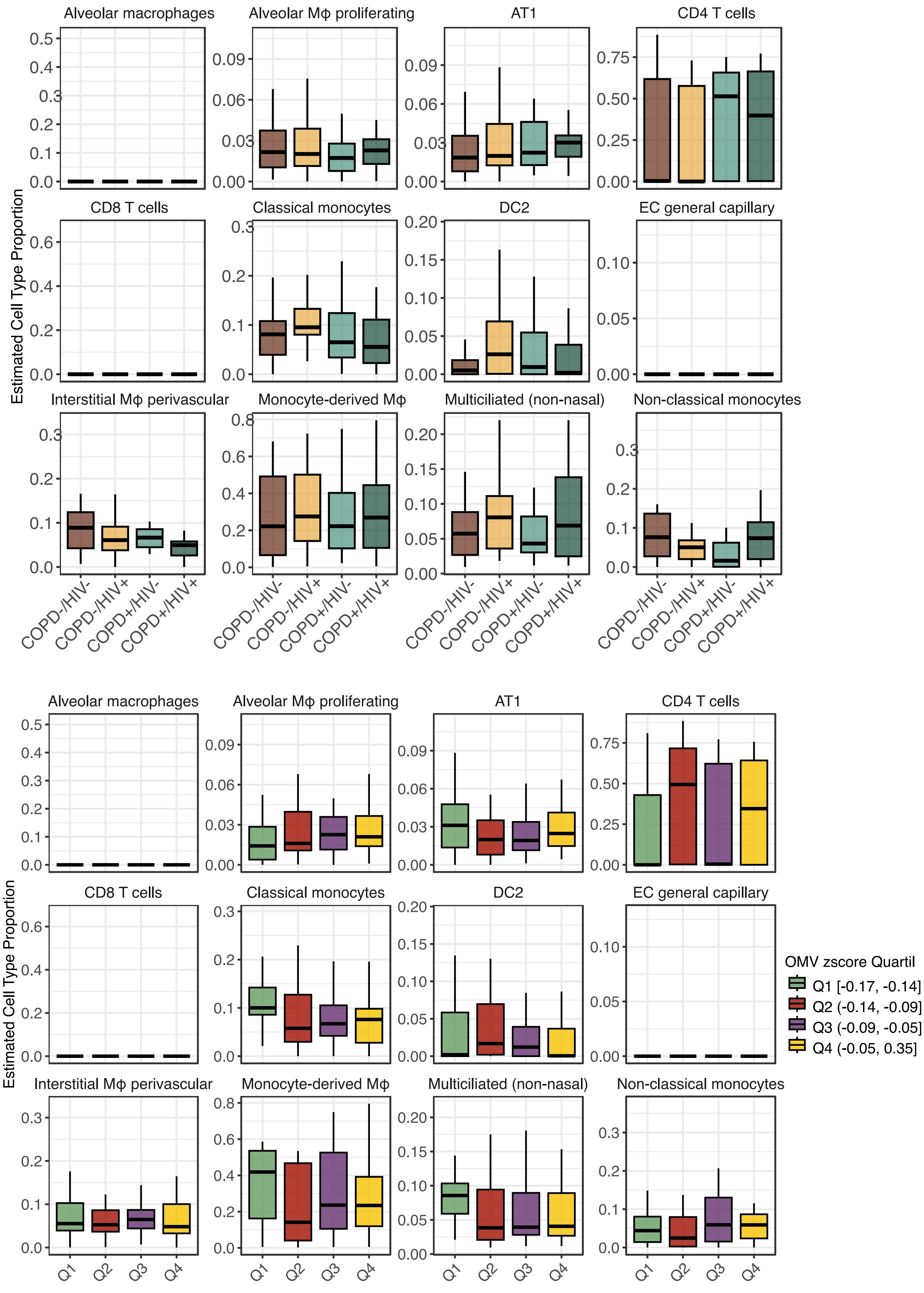
Alveolar macrophages
Alveolar Mϕ proliferating
AT1
CD4 T cells
CD8 T cells
Classical monocytes
DC2
EC general capillary
Interstitial Mϕ perivascular
Monocyte-derived Mϕ
Multiciliated (non-nasal)
Non-classical monocytes
Estimated Cell Type Proportion
COPD-/HIV-
COPD-/HIV+
COPD+/HIV-
COPD+/HIV+
Q1
Q2
Q3
Q4
OMV zscore Quartil
Q1 [-0.17, -0.14]
Q2 (-0.14, -0.09]
Q3 (-0.09, -0.05]
Q4 (-0.05, 0.35]

Supplementary Figure S23. Lung Cell Atlas-guided deconvolution of induced-sputum cellular composition according to HIV/COPD status. Estimated cell-type proportions in induced sputum obtained using the Lung Cell Atlas as the reference, stratified by HIV/COPD status. Analyses included *n* = 100 biologically independent participants: COPD-/HIV- (*n* = 25), COPD−/HIV+ (*n* = 26), COPD+/HIV− (*n* = 23), and COPD+/HIV+ (*n* = 26). Each stacked bar represents one participant and shows the inferred relative proportions of the indicated cell populations. Each participant constituted the biological unit of study; deconvolution-derived cell proportions are model-based estimates, and no technical replicates were treated as independent observations. Source data are provided as a Source Data file.

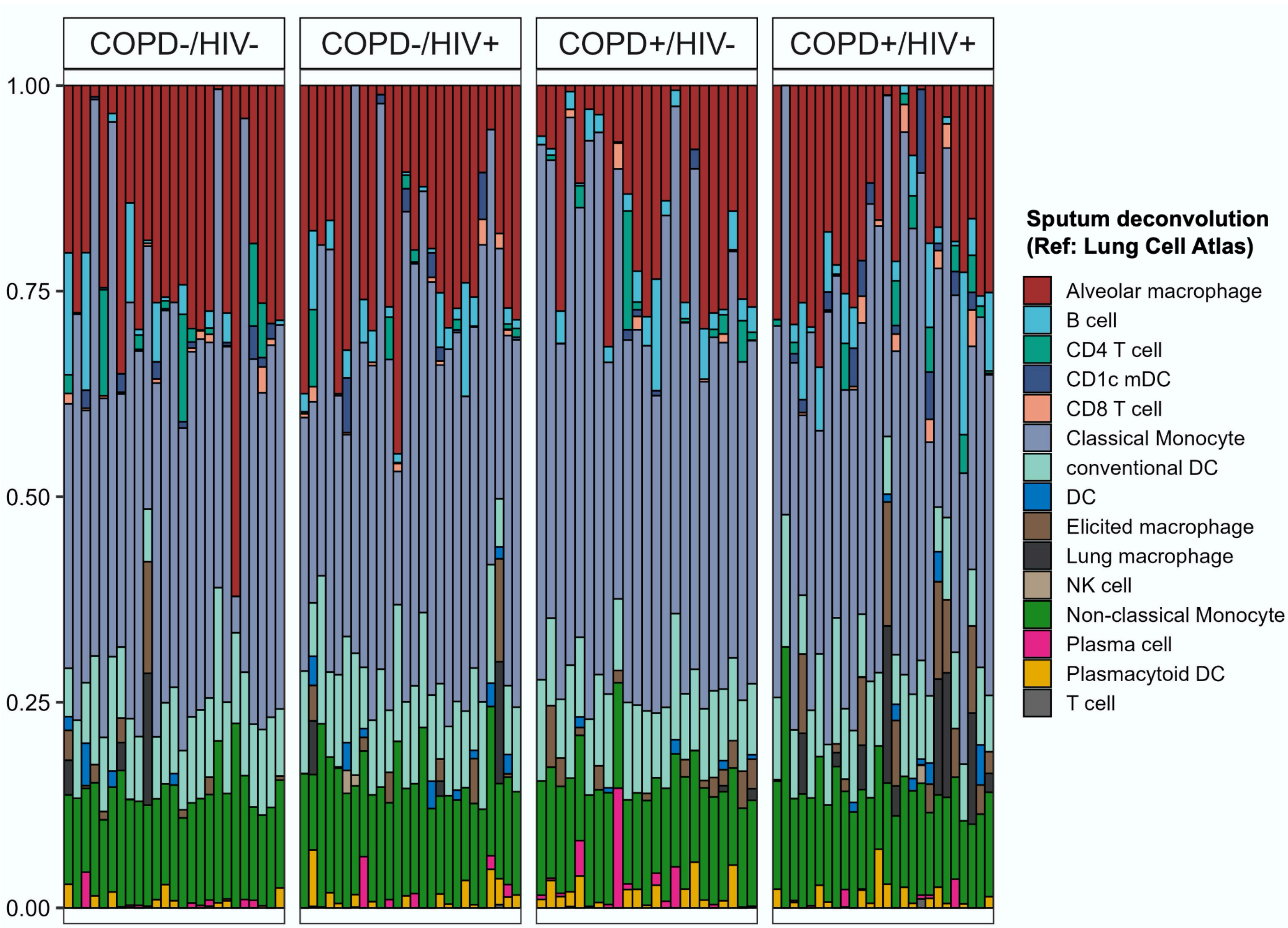

Supplementary Figure S24. Disease-associated differences in HLCA-inferred sputum cellular composition. Cell-type proportions were estimated from induced-sputum bulk RNA-sequencing data from *n* = 100 biologically independent participants using the Human Lung Cell Atlas (HLCA) as an external reference. Boxplots show inferred proportions across four disease groups: COPD-/HIV− (*n* = 25), COPD-/HIV+ (*n* = 26), COPD+/HIV- (*n* = 23), and COPD+/HIV+ (*n* = 26). Points represent individual participants; centre lines indicate medians, boxes indicate the interquartile range, and whiskers extend to the most extreme data points within 1.5 × the interquartile range. Differences across the four groups were assessed independently for each cell population using Kruskal-Wallis tests, with Benjamini-Hochberg correction across the 12 testable cell populations; adjusted *P* values (BH-FDR) and Kruskal-Wallis epsilon-squared (ε^2) effect sizes are shown above each panel. Brackets show post-hoc Dunn comparisons of each disease group against the COPD-/HIV- reference group, with *P* values adjusted for multiple pairwise comparisons; * $P < 0.05$; ns, not significant. Each participant constituted the biological unit of study; deconvolution-derived cell proportions are model-based estimates, and no technical replicates were treated as independent observations. DC, dendritic cell; HLCA, Human Lung Cell Atlas; BH-FDR, Benjamini-Hochberg false-discovery rate. Source data are provided as a Source Data file.

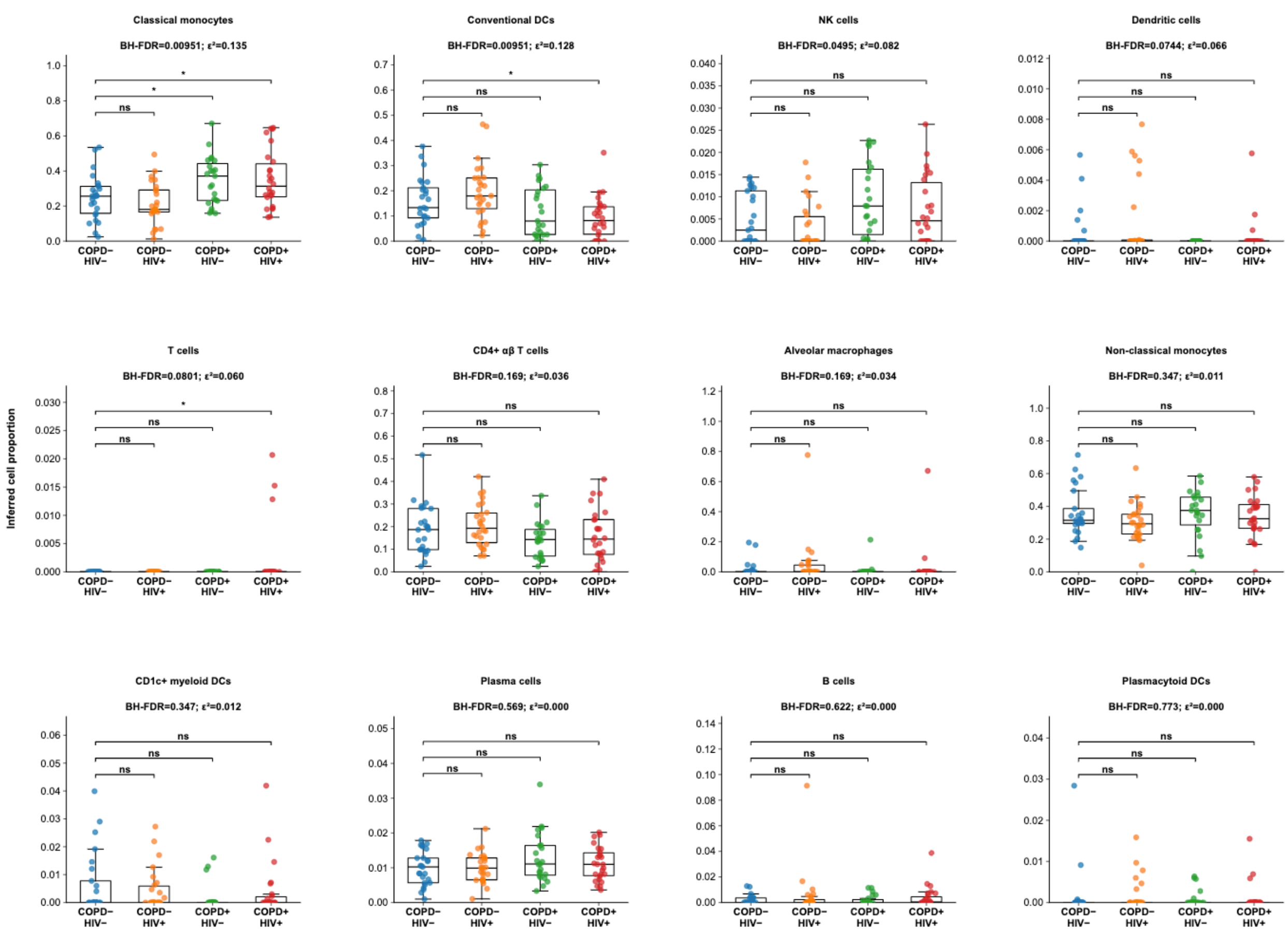

Supplementary Figure S25. Disease-associated differences in BAL snRNA-seq/MuSiC-inferred sputum cellular composition. Cell-type proportions were independently estimated from induced-sputum bulk RNA-sequencing data from n = 48 biologically independent participants using MuSiC with BAL single-nucleus RNA-sequencing profiles as the reference. Boxplots show inferred proportions across four disease groups: COPD-/HIV- (n = 12), COPD−/HIV+ (n = 12), COPD+/HIV- (n = 13), and COPD+/HIV+ (n = 11). Points represent individual participants; centre lines indicate medians, boxes indicate the interquartile range, and whiskers extend to the most extreme data points within 1.5 × the interquartile range. Twelve cell populations inferred using the BAL reference are shown, including alveolar macrophage, T-cell, monocyte, dendritic-cell, epithelial and endothelial populations. Differences across the four disease groups were assessed independently for each cell population using Kruskal-Wallis tests, with Benjamini-Hochberg correction across the 12 populations; adjusted P values (BH-FDR) and Kruskal-Wallis epsilon-squared (ε^2) effect sizes are shown above each panel. Brackets show post-hoc Dunn comparisons of each disease group against the COPD-/HIV- reference group, with P values adjusted across pairwise comparisons using the Benjamini-Hochberg procedure; $P < 0.05$; ns, not significant. Each participant constituted the biological unit of study; deconvolution-derived cell proportions are model-based estimates, and no technical replicates were treated as independent observations. Mϕ, macrophage; DC, dendritic cell; AT1, alveolar type 1 epithelial cell; MuSiC, Multi-subject Single Cell deconvolution; BH-FDR, Benjamini-Hochberg false-discovery rate. Source data are provided as a Source Data file.

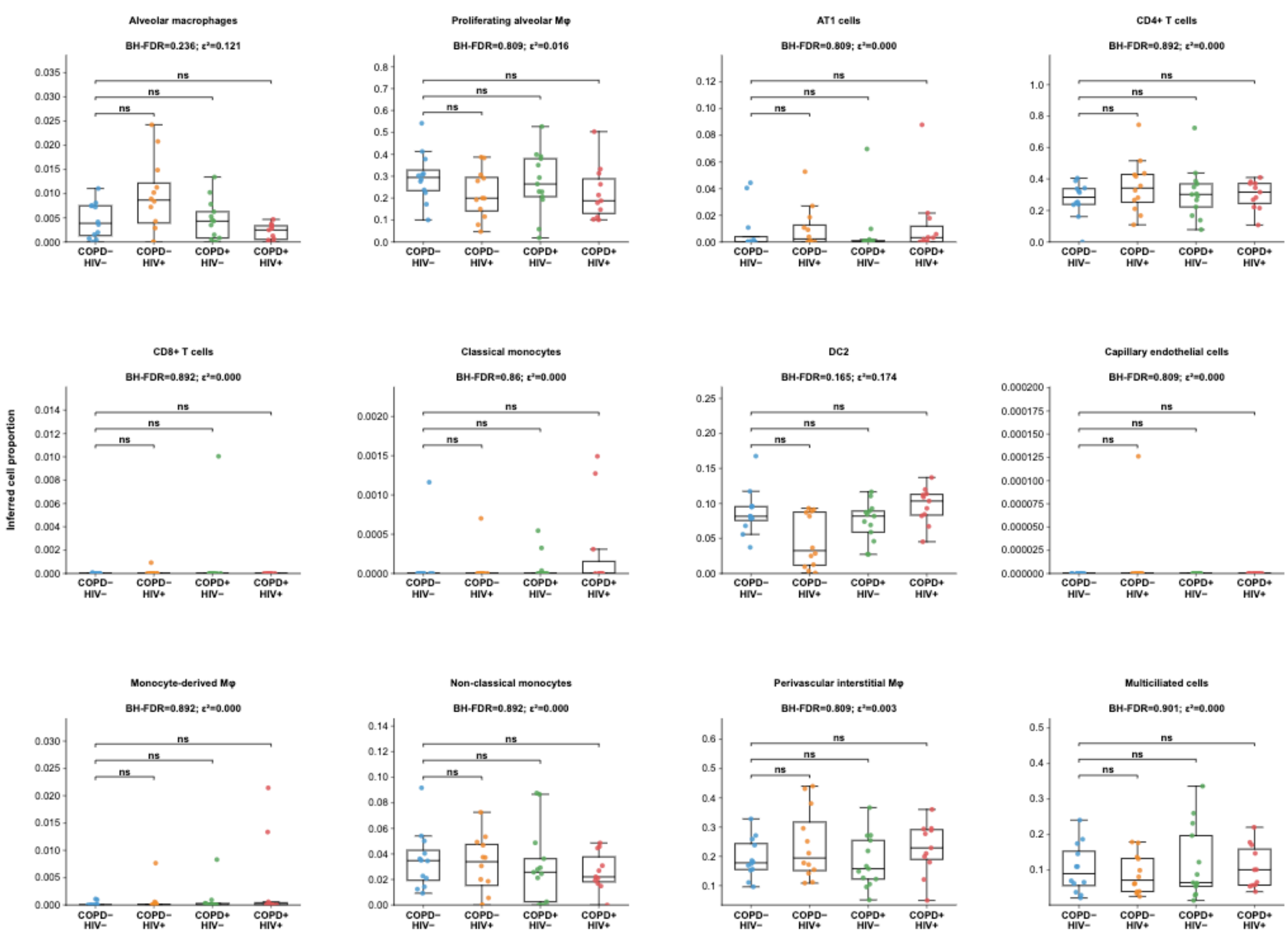

Supplementary Figure S26. Reference sensitivity of inferred sputum cellular composition across HIV/COPD groups. Principal-component analysis (PCA) of centred log-ratio (CLR)-transformed inferred cell-type proportions was used to compare the global structure of sputum cellular composition obtained using two independent deconvolution references. (A) HLCA-reference deconvolution included *n* = 100 biologically independent participants: COPD-/HIV- (*n* = 25), COPD−/HIV+ (*n* = 26), COPD+/HIV− (*n* = 23), and COPD+/HIV+ (*n* = 26). (B) BAL snRNA-seq/MuSiC-reference deconvolution included *n* = 48 biologically independent participants: COPD-/HIV- (*n* = 12), COPD−/HIV+ (*n* = 12), COPD+/HIV- (*n* = 13), and COPD+/HIV+ (*n* = 11). Points represent individual participants and colours denote the four HIV/COPD groups. Differences in global inferred cellular composition among disease groups were assessed using PERMANOVA; *P* values and R^2 effect sizes are shown within each panel. These analyses assess the sensitivity of disease-associated global compositional structure to the choice of deconvolution reference. Each participant constituted the biological unit of study; deconvolution-derived cell proportions are model-based estimates, and no technical replicates were treated as independent observations. HLCA, Human Lung Cell Atlas; snRNA-seq, single-nucleus RNA sequencing; MuSiC, Multi-subject Single Cell deconvolution; CLR, centred log-ratio; PCA, principal-component analysis; PERMANOVA, permutational multivariate analysis of variance. Source data are provided as a Source Data file.

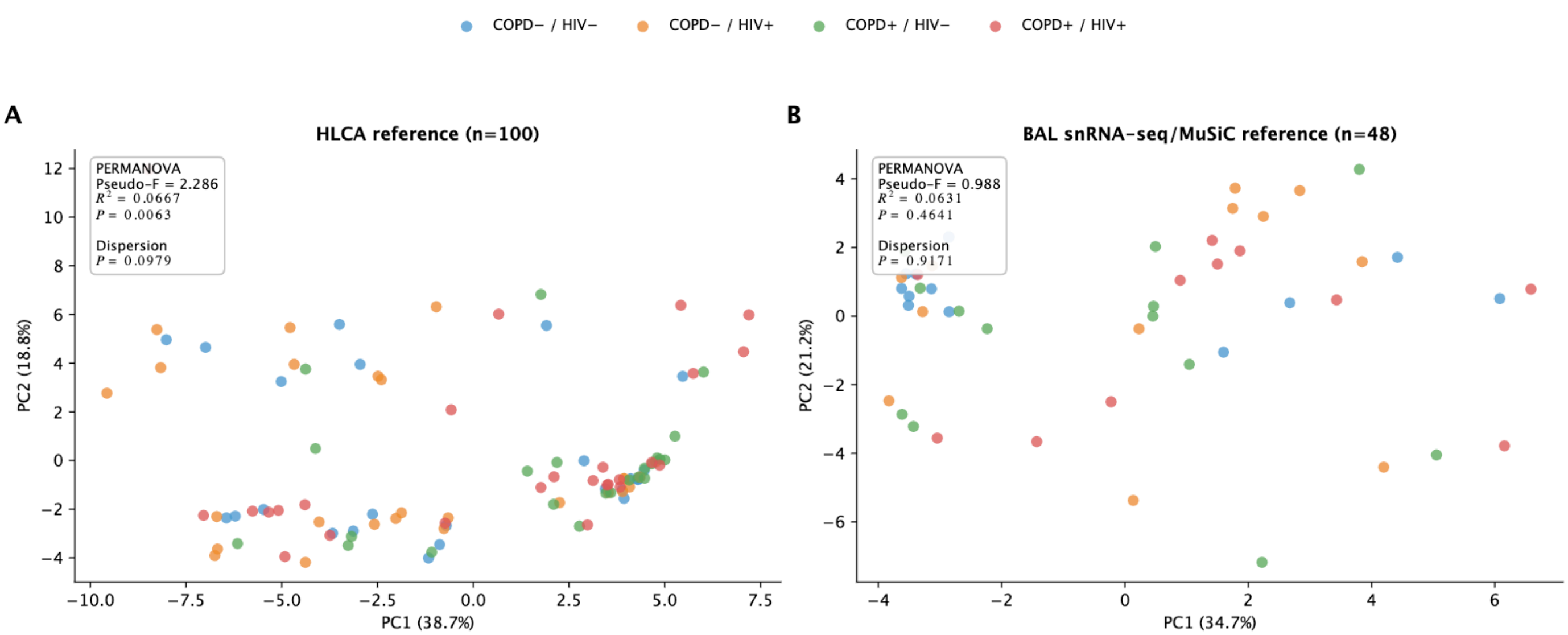

Supplementary Figure S27. OMV × HIV/COPD status modelling of BAL/MuSiC-inferred sputum cell-type proportions. Prediction plots show associations between standardized outer membrane vesicle (OMV) concentration and BAL snRNA-seq/MuSiC-inferred cell-type proportions, stratified by HIV/COPD status. Analyses were based on *n* = 48 biologically independent participants: COPD-/HIV− (*n* = 12), COPD−/HIV+ (*n* = 12), COPD+/HIV- (*n* = 13), and COPD+/HIV+ (*n* = 11). Points represent observed participant-level inferred proportions, lines represent model-predicted relationships, and shaded ribbons indicate 95% confidence intervals. Linear models (LMs) and generalized additive models (GAMs) were compared using the Akaike Information Criterion, with *Δ*AIC > 2 indicating preference for the GAM; facet labels indicate the preferred model for each cell type. Models evaluated OMV × HIV/COPD status interactions while accounting for smoking, age, sex and BMI. Predictions were generated across the observed OMV range while holding covariates constant. Each participant constituted the biological unit of study; deconvolution-derived cell proportions are model-based estimates, and no technical replicates were treated as independent observations. OMV, outer membrane vesicle; LM, linear model; GAM, generalized additive model; MuSiC, Multi-subject Single Cell deconvolution. Source data are provided as a Source Data file.

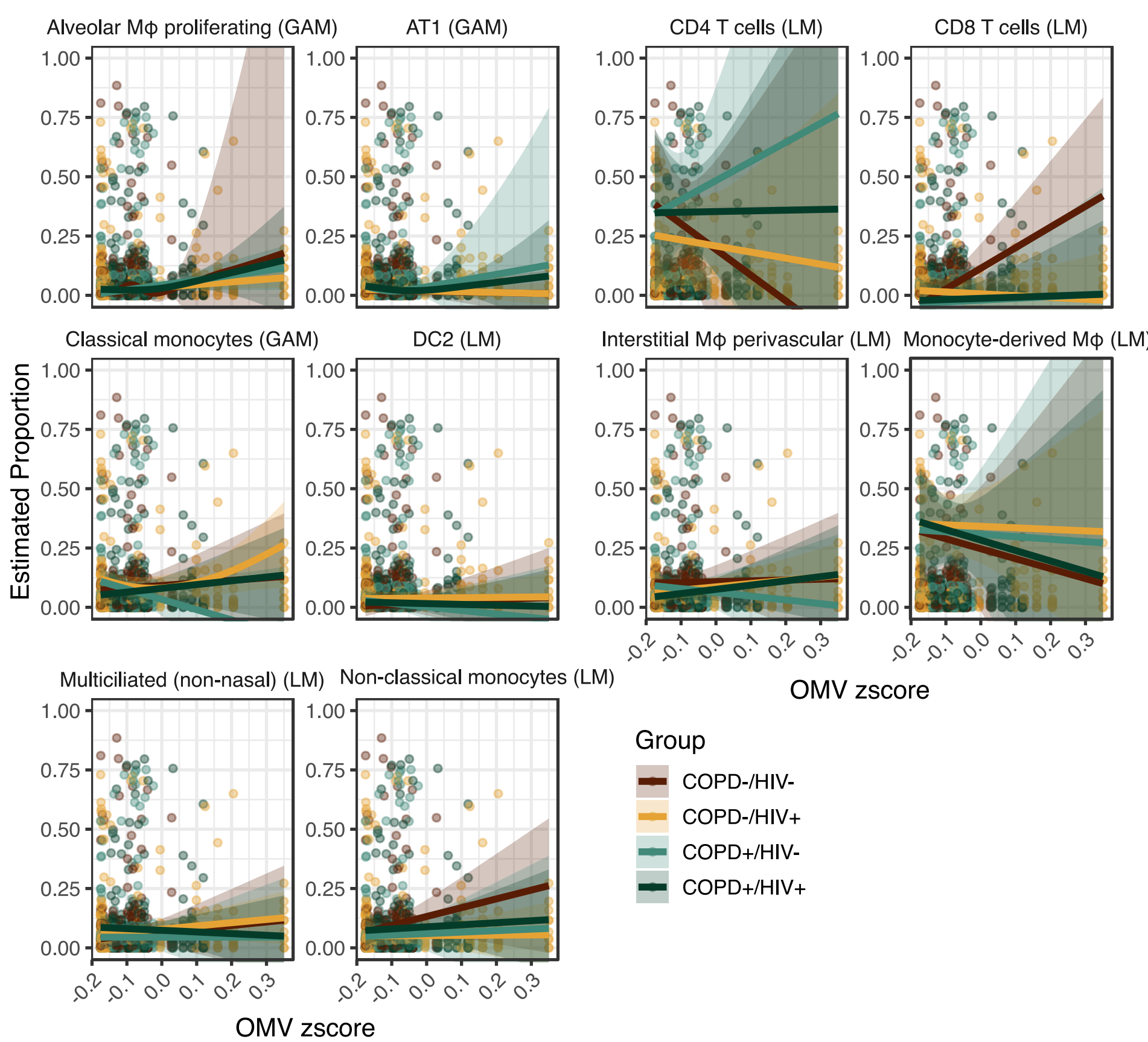

Supplementary Figure S28. Adjusted associations between HIV/COPD status and BAL/MuSiC-inferred sputum cell-type proportions. Point estimates and 95% confidence intervals show adjusted differences in inferred cell-type proportions for COPD-/HIV+ (*n* = 12), COPD+/HIV- (*n* = 13), and COPD+/HIV+ (*n* = 11), relative to the COPD-/HIV- reference group (*n* = 12). Analyses included *n* = 48 biologically independent participants. Cell type-wise models were adjusted for smoking, age, sex and BMI. Points represent adjusted effect estimates and error bars represent 95% confidence intervals. No disease-group differences remained statistically significant after Benjamini-Hochberg false discovery rate correction. Each participant constituted the biological unit of study; deconvolution-derived cell proportions are model-based estimates, and no technical replicates were treated as independent observations. MuSiC, Multi-subject Single Cell deconvolution. Source data are provided as a Source Data file.

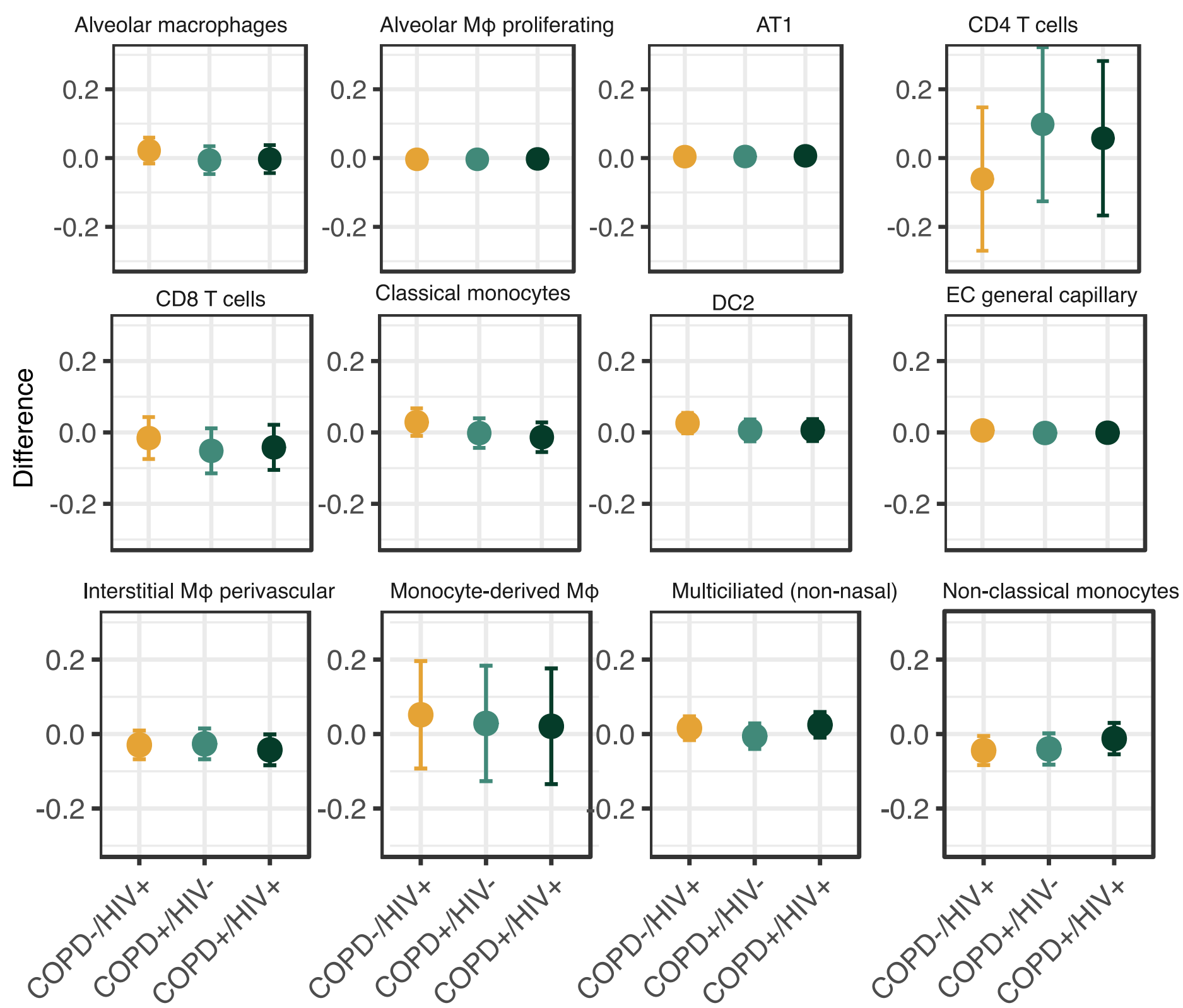

Supplementary Figure S29. OMV × dual-disease modelling of BAL/MuSiC-inferred sputum cell-type proportions. Prediction plots show associations between standardized outer membrane vesicle (OMV) concentration and BAL snRNA-seq/MuSiC-inferred cell-type proportions, stratified by dual-disease status. Analyses included *n* = 48 biologically independent participants: dual-disease negative (Dualdisease = 0; HIV-COPD-, *n* = 37) and dual-disease positive (Dualdisease = 1; HIV+/COPD+; *n* = 11). Points represent observed participant-level inferred cell proportions, lines represent model-predicted relationships, and shaded ribbons indicate 95% confidence intervals. Linear models (LMs) and generalized additive models (GAMs) were compared using the Akaike Information Criterion, with *Δ*AIC > 2 indicating preference for the GAM; facet labels indicate the preferred model for each cell type. Models evaluated OMV × dual-disease interactions while accounting for smoking, age, sex and BMI. Predictions were generated across the observed OMV range while holding covariates constant. No OMV × dual-disease interaction remained statistically significant after correction for multiple testing. Each participant constituted the biological unit of study; deconvolution-derived cell proportions are model-based estimates, and no technical replicates were treated as independent observations. OMV, outer membrane vesicle; LM, linear model; GAM, generalized additive model; MuSiC, Multi-subject Single Cell deconvolution. Source data are provided as a Source Data file.

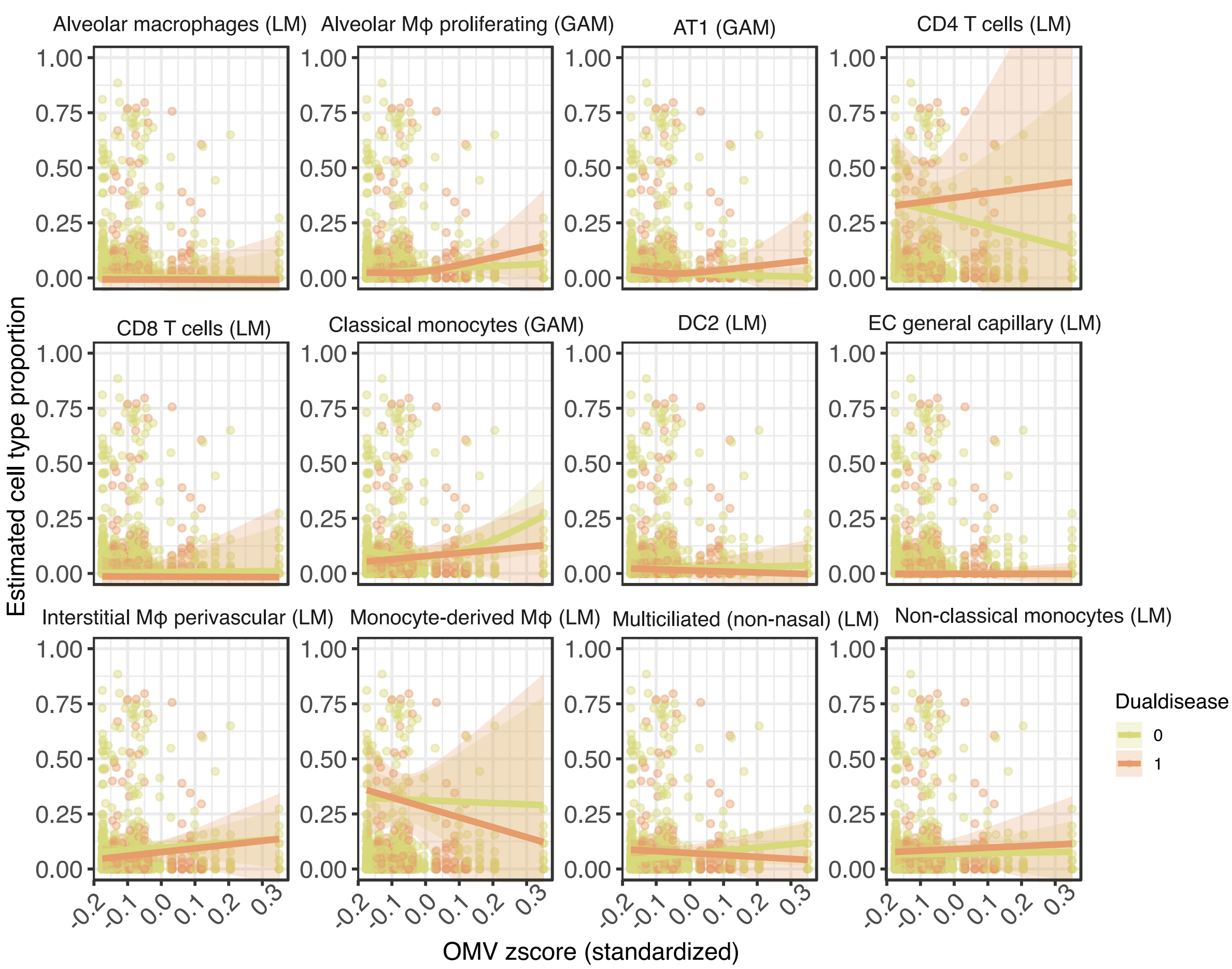

Supplementary Figure S30. Adjusted associations between dual-disease status and BAL/MuSiC-inferred sputum cell-type proportions. Point estimates and 95% confidence intervals show adjusted differences in inferred cell-type proportions between dual-disease-positive participants (Dualdisease = 1; HIV+/COPD+; *n* = 11) and dual-disease-negative participants (Dualdisease = 0; HIV-COPD-, *n* = 37). Analyses included *n* = 48 biologically independent participants. Cell type-wise models were adjusted for smoking, age, sex and BMI. Points represent adjusted effect estimates for the Dualdisease 1 versus 0 contrast, and error bars represent 95% confidence intervals. No differences remained statistically significant after Benjamini-Hochberg false discovery rate correction. Each participant constituted the biological unit of study; deconvolution-derived cell proportions are model-based estimates, and no technical replicates were treated as independent observations. MuSiC, Multi-subject Single Cell deconvolution. Source data are provided as a Source Data file.

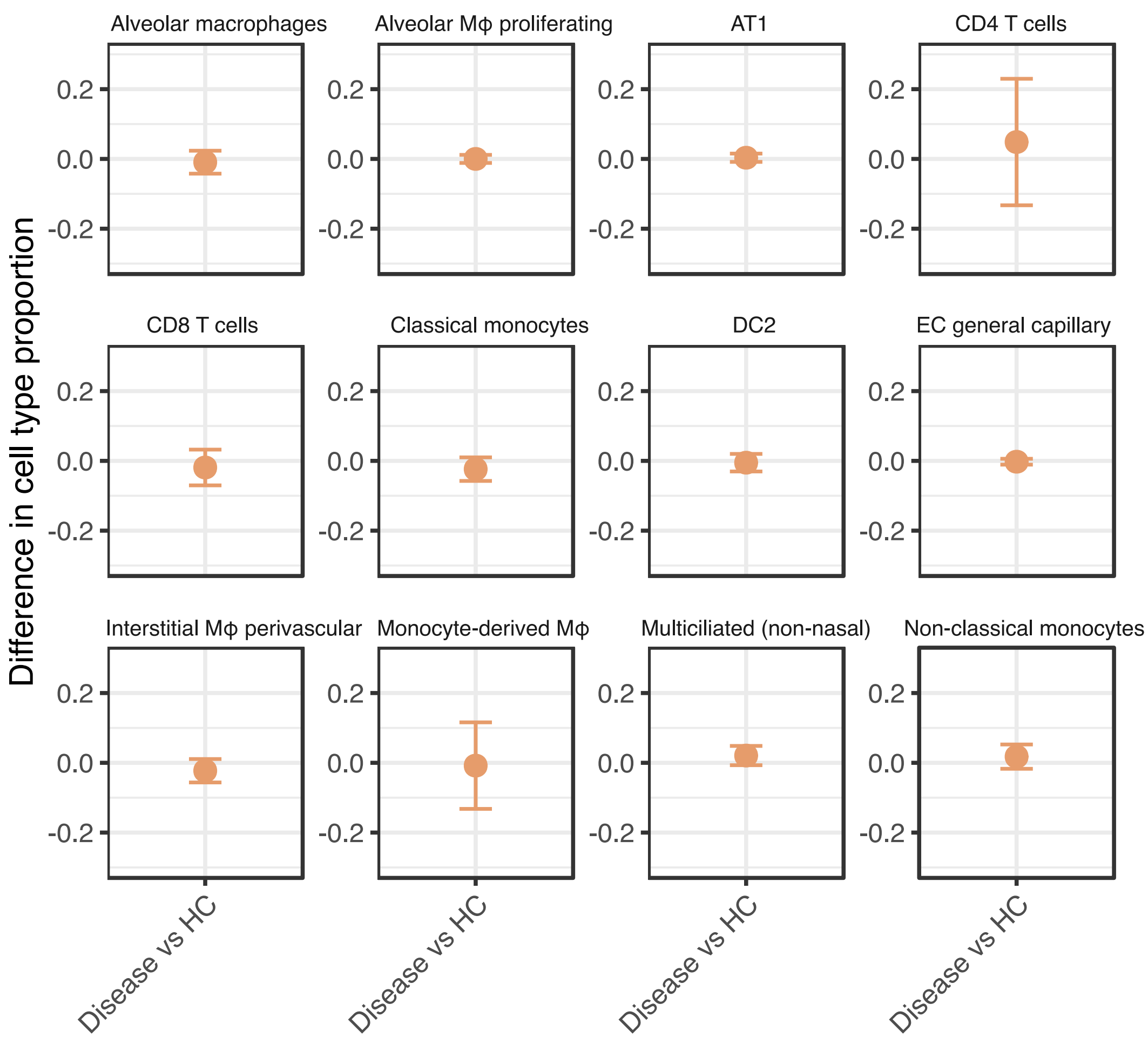

Supplementary Figure S31. Canonical correlation analysis linking BAL/MuSiC-inferred cell-type composition to clinical variables. Canonical correlation analysis (CCA) was used to assess multivariate covariance between inferred sputum cell-type composition and clinical variables. Analyses included *n* = 48 biologically independent participants. Each point represents one participant, plotted according to the first canonical variate for cell-type composition (U_1; x-axis) and the first canonical variate for clinical variables (V_1; y-axis). (A) CCA projections coloured by HIV/COPD status: COPD-/HIV- (*n* = 12), COPD-/HIV+ (*n* = 12), COPD+/HIV- (*n* = 13), and COPD+/HIV+ (*n* = 11). (B) The same CCA projections coloured by quartiles of standardized outer membrane vesicle (OMV) concentration (Q1-Q4). (C) The same CCA projections coloured by dual-disease status: dual-disease negative (Dualdisease = 0; *n* = 37) and dual-disease positive (Dualdisease = 1; HIV+/COPD+; *n* = 11). Each participant constituted the biological unit of study; deconvolution-derived cell proportions are model-based estimates, and no technical replicates were treated as independent observations. CCA, canonical correlation analysis; OMV, outer membrane vesicle; MuSiC, Multi-subject Single Cell deconvolution. Source data are provided as a Source Data file.

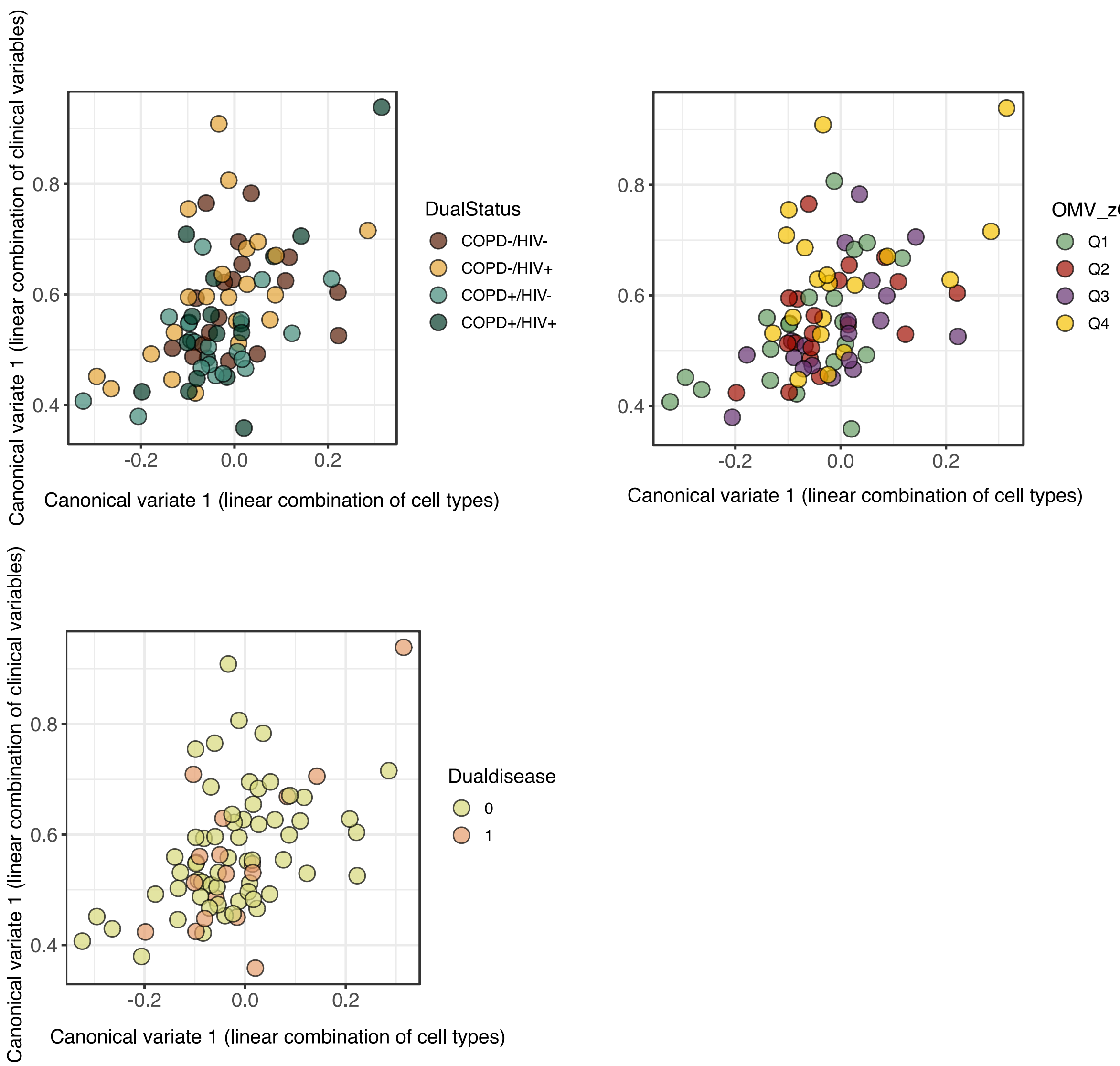

Supplementary Table ST1: Bloomer-decreaser taxonomy table. Bacterial genera were classified as bloomers or decreasers according to the direction of change in mean relative abundance between visit 1 (V1) and visit 2 (V2) among *n* = 26 biologically independent COPD-/HIV- control participants with paired samples (52 samples in total). Each participant constituted the biological unit of study, with V1 and V2 representing repeated measurements from the same participant. genus, bacterial genus identified by 16S rRNA gene sequencing; category, classification as a bloomer (increased from V1 to V2 in controls) or decreaser (decreased from V1 to V2 in controls); mean_relAb1, mean relative abundance at V1; mean_relAb2, mean relative abundance at V2; $\log_2$ ratio of mean relative abundance at V2 relative to V1. No statistical hypothesis testing was performed for this descriptive classification. Source data are provided as a Source Data file.

Genus	Category	mean_relAb1	mean_relAb2	$\log_2$ ratio
Actinobacteriota_Atopobium	Bloomer (increased V1→V2 in controls)	0.0011	0.00305	1.022
Actinobacteriota_Actinomyces	Bloomer (increased V1→V2 in controls)	0.00766	0.01874	0.894
Proteobacteria_Aggregatibacter	Decreaser (decreased V1→V2 in controls)	0.00926	0.00351	−0.97
Bacteroidota_Prevotella	Decreaser (decreased V1→V2 in controls)	0.04399	0.01788	−0.90
Firmicutes_unknown_genera_15	Decreaser (decreased V1→V2 in controls)	0.0028	0.00121	−0.839
Proteobacteria_Haemophilus	Decreaser (decreased V1→V2 in controls)	0.08978	0.04224	−0.754
Proteobacteria_TMTx	Decreaser (decreased V1→V2 in controls)	0.00751	0.00367	−0.717
Proteobacteria_unknown_genera	Decreaser (decreased V1→V2 in controls)	0.00208	0.00113	−0.61
Bacteroidota_Prevotella_7	Decreaser (decreased V1→V2 in controls)	0.11429	0.06287	−0.603
Firmicutes_Megasphaera	Decreaser (decreased V1→V2 in controls)	0.00223	0.00122	−0.602
Firmicutes_Veillonella	Decreaser (decreased V1→V2 in controls)	0.07006	0.03917	−0.581
Campylobacterota_Campylobacter	Decreaser (decreased V1→V2 in controls)	0.00218	0.00124	−0.563

Supplementary Table ST2: Paired t-test, linear mixed-effects model (LMM), and likelihood-ratio test (LRT) results for alpha diversity. Longitudinal changes in OTU-level alpha diversity were evaluated using a two-sided paired t-test comparing visit 2 (V2) with visit 1 (V1) among *n* = 26 biologically independent COPD-/HIV- control participants with paired samples. Linear mixed-effects models (LMMs) were fitted to the paired longitudinal cohort (*n* = 101 biologically independent participants) with participant ID included as a random effect to account for repeated measurements. LMM results are shown for the visit and age effects in unadjusted and drift-adjusted models. The visit × DualStatus interaction was evaluated using a likelihood-ratio test (LRT) comparing models with and without the interaction term. Each participant constituted the biological unit of study, with V1 and V2 representing repeated measurements from the same participant. finding, analysis or model term evaluated; estimate, estimated mean paired difference or LMM regression coefficient, as applicable; se_or_stat, standard error (SE) of the estimate or LRT chi-square statistic and degrees of freedom (df), as applicable; p_value, P value for the corresponding statistical test. Tests of individual LMM coefficients were two-sided. No multiple-comparison adjustment was applied to the results presented in this table. Source data are provided as a Source Data file.

Finding	Estimate	se_or_stat	*P* value
Paired t-test: controls V2 vs V1	−0.4279	CI (−0.726, −0.13)	0.00672
LMM controls: visit coefficient (UNADJUSTED)	−0.4884	SE=0.0967	2.44×10^{-6}
LMM controls: visit coefficient (DRIFT-ADJUSTED)	−0.3251	SE=0.1026	0.04116
LMM controls: age effect (UNADJUSTED)	0.0147	SE=0.0051	0.00486
LMM controls: age effect (DRIFT-ADJUSTED)	0.0121	SE=0.0051	0.0199
LRT: visit × DualStatus interaction	NA	χ^2=0.941, *df*=3	0.816

Data file: Supplementary Table ST3: Visit × DualStatus interaction analysis of longitudinal bacterial genus abundance. Linear mixed-effects models (LMMs) were used to test whether changes in bacterial genus abundance between visit 1 (V1) and visit 2 (V2) differed according to HIV/COPD status (DualStatus) among *n* = 101 biologically independent participants with paired longitudinal microbiome data (COPD-/HIV-, *n* = 26; COPD-/HIV+, *n* = 28; COPD+/HIV−, *n* = 24; COPD+/HIV+, *n* = 23). Participant ID was included as a random effect to account for repeated measurements, with age, sex, BMI and smoking status included as covariates. COPD-/HIV- was the reference group. Each participant constituted the biological unit of study, with V1 and V2 representing repeated measurements from the same participant. term, visit × DualStatus interaction term tested; Estimate, estimated interaction coefficient relative to the reference group; Std. Error, standard error of the coefficient; df, degrees of freedom; t value, t-statistic for the coefficient; pval, two-sided P value for the coefficient; sample_n, number of observations included in the corresponding model; q_value, Benjamini-Hochberg FDR-adjusted *P* value. Statistical significance was defined as FDR-adjusted $q < 0.10$. Source data are provided as a Source Data file.

Supplementary Table ST4: OTU-level sensitivity analysis for temporal microbiome drift. OTU-level interaction results were compared between unadjusted and drift-adjusted linear mixed-effects models (LMMs) among *n* = 101 biologically independent participants with paired longitudinal microbiome data (COPD-/HIV-, *n* = 26; COPD-/HIV+, *n* = 28; COPD+/HIV-, *n* = 24; COPD+/HIV+, *n* = 23) to assess the robustness of associations to temporal microbiome drift. Each participant constituted the biological unit of study, with visit 1 (V1) and visit 2 (V2) representing repeated measurements from the same participant. OTU, operational taxonomic unit identifier; interaction_p_unadj_ART, P value for the interaction term in the unadjusted analysis; interaction_p_adj_LM, P value for the interaction term in the drift-adjusted linear model; survives_at_p05, indicates whether the interaction remained significant at $P < 0.05$ after drift adjustment; survives_at_p10, indicates whether the interaction remained significant at $P < 0.10$ after drift adjustment; note, model-fitting status. Source data are provided as a Source Data file.

OTU	Unadjusted ART interaction P value	Drift-adjusted LM interaction P value	P < 0.05 after adjustment	P < 0.10 after adjustment	Model status
OTU118	0.000416	0.00379	Yes	Yes	Model fitted
OTU25	0.00206	0.00875	Yes	Yes	Model fitted
OTU255	0.0117	0.0283	Yes	Yes	Model fitted
OTU4	8.62×10^{-8}	0.000150	Yes	Yes	Model fitted
OTU66	3.94×10^{-5}	0.119	No	No	Model fitted
OTU8	6.48×10^{-7}	0.000852	Yes	Yes	Model fitted

Supplementary Table ST5: Sensitivity analysis of *Haemophilus* and *Sphingomonas* associations after adjustment for temporal microbiome drift. Linear models were used to evaluate associations of stability group, DualStatus, and their interaction with the relative abundance of Haemophilus and Sphingomonas among *n* = 101 biologically independent participants with paired longitudinal microbiome data (COPD-/HIV-, *n* = 26; COPD-/HIV+, *n* = 28; COPD+/HIV-, *n* = 24; COPD+/HIV+, *n* = 23), comparing unadjusted and drift-adjusted models. Each participant constituted the biological unit of study, with visit 1 (V1) and visit 2 (V2) representing repeated measurements from the same participant. curve, model term evaluated; term, predictor or interaction term; *Df*, degrees of freedom; Df.res, residual degrees of freedom; Sum Sq, sum of squares; Sum Sq.res, residual sum of squares; *F* value, F-statistic for the corresponding model term; Pr(>F), *P* value from the F-test; genus, bacterial genus analysed; model, unadjusted or drift-adjusted model; Mean Sq, mean square for the corresponding model term. Source data are provided as a Source Data file.

Genus	Model	Interaction F	Interaction P value	Interpretation
Proteobacteria_Haemophilus	Unadjusted ART (rank)	3.38	0.0043	Stability × DualStatus interaction significant
Proteobacteria_Haemophilus	Drift-adjusted LM (log relative abundance)	3.02	0.0094	Stability × DualStatus interaction remains significant after drift adjustment
Proteobacteria_Sphingomonas	Unadjusted ART (rank)	0.58	0.75	No Stability × DualStatus interaction; main effect only
Proteobacteria_Sphingomonas	Drift-adjusted LM (log relative abundance)	0.58	0.75	No Stability × DualStatus interaction after drift adjustment

Supplementary Table ST6: Associations between microbiome stability and longitudinal changes in lung function. Linear regression models were used to assess associations between microbiome stability and changes in lung function from baseline to follow-up among *n* = 113 biologically independent participants with longitudinal lung-function data included in each model. Each participant constituted the biological unit of study, with longitudinal change calculated from the participant's baseline and follow-up measurements. outcome, longitudinal change in the specified lung function measure (*Δ* = follow-up − baseline); n_obs, number of biologically independent participants included in the model; stability_F, F-statistic for the microbiome stability term; stability_p, *P* value from the F-test for the stability term; R^2_adj, adjusted coefficient of determination (adjusted R^2) for the model; stability_p_BH, Benjamini-Hochberg (BH)-adjusted P value for the stability term. ΔFEV_1, change in forced expiratory volume in one second; ΔFVC, change in forced vital capacity; Δratio, change in FEV_1/FVC ratio. Source data are provided as a Source Data file.

Outcome	n_obs	Stability F	Stability P	Adjusted R^2	BH-adjusted P
ΔFEV_1	113	1.328	0.269	0.039	0.414
*Δ*FVC	113	0.585	0.559	0.002	0.559
ΔFEV_1/FVC ratio	113	1.304	0.276	−0.009	0.414

Data file: Supplementary Table ST7: Confounder-aware associations between HIV status and induced sputum gene expression among COPD-positive participants. Associations between HIV status and gene expression in induced sputum were assessed among COPD-positive participants (*n*=38), comparing COPD+/HIV+ with COPD+/HIV-, using two-sided Wilcoxon tests within the metadeconfoundR framework, with P values adjusted for multiple comparisons using the Benjamini-Hochberg (BH) procedure. All genes are displayed. feature, gene analysed; metaVariable, clinical variable tested; PS, nominal P value for the association; Qs, BH-adjusted *P* value (FDR); Ds, Cliff's *δ* effect size; status, significance/confounding classification assigned by the metadeconfoundR analysis. NS, not significant; OK, significant association retained after confounder assessment; QC, association affected by confounding. Source data are provided as a Source Data file.

Data file: Supplementary Table ST8: Confounder-aware associations between HIV status and PBMC gene expression among COPD-positive participants. Associations between HIV status and gene expression in peripheral blood mononuclear cells (PBMCs) were assessed among COPD-positive participants (*n*=14), comparing COPD+/HIV+ with COPD+/HIV-, using two-sided Wilcoxon tests within the metadeconfoundR framework, with P values adjusted for multiple comparisons using the Benjamini-Hochberg (BH) procedure. All genes are displayed. feature, gene analysed; metaVariable, clinical variable tested; PS, nominal P value for the association; Qs, BH-adjusted P value (FDR); Ds, Cliff's δ effect size; status, significance/confounding classification assigned by the metadeconfoundR analysis. Source data are provided as a Source Data file.

Data file: Supplementary Table ST9: Confounder-aware associations between HIV status and BAL gene expression among COPD-positive participants. Associations between HIV status and gene expression in bronchoalveolar lavage (BAL) were assessed among COPD-positive participants (*n*=24), comparing COPD+/HIV+ with COPD+/HIV-, using two-sided Wilcoxon tests within the metadeconfoundR framework, with P values adjusted for multiple comparisons using the Benjamini-Hochberg (BH) procedure. All genes are displayed. feature, gene analysed; metaVariable, clinical variable tested; PS, nominal *P* value for the association; Qs, BH-adjusted *P* value (FDR); Ds, Cliff's δ effect size; status, significance/confounding classification assigned by the metadeconfoundR analysis. Source data are provided as a Source Data file.

Supplementary Table ST10: Correlations between inferred BAL cell-type proportions and OMV levels. Two-sided Spearman rank correlations between inferred cell-type proportions and omv_z were assessed among *n* = 48 biologically independent participants. Each participant constituted the biological unit of study, and cell-type proportions were model-derived estimates from BAL transcriptomic data. P values were adjusted for multiple comparisons using the Benjamini-Hochberg (BH) procedure; all adjusted P values (padj) were >0.1. ρ, Spearman's rank correlation coefficient. Source data are provided as a Source Data file.

Cell Type	ρ	p-value	p_{adj}
Alveolar Mφ proliferating	0.192	0.0915	0.549
Classical monocytes	-0.209	0.0665	0.549
Multiciliated (non-nasal)	-0.155	0.177	0.707
CD8 T cells	-0.135	0.24	0.719
CD4 T cells	0.118	0.305	0.732
Non-classical monocytes	0.102	0.374	0.748
Monocyte-derived Mφ	-0.0865	0.452	0.774
AT1	-0.0371	0.747	0.897
DC2	-0.0398	0.729	0.897
Interstitial Mφ perivascular	-0.0451	0.695	0.897
Alveolar macrophages	0.00759	0.947	0.947
EC general capillary	0.00759	0.947	0.947

Supplementary Table ST11: Adjusted associations between OMV levels and inferred BAL cell-type proportions. Cell type-wise linear regression models were used to assess associations between OMV levels and inferred cell-type proportions among *n* = 48 biologically independent participants, adjusting for smoking, age, sex, and BMI. Each participant constituted the biological unit of study, and cell-type proportions were model-derived estimates from BAL transcriptomic data. Statistical significance of regression coefficients was assessed using two-sided t-tests. P values were adjusted for multiple comparisons using the Benjamini-Hochberg (BH) procedure. Estimate, regression coefficient; SE, standard error; t, t-statistic; padj, BH-adjusted P value. Source data are provided as a Source Data file.

Cell Type	Estimate	SE	t	p	p_{adj}
Alveolar Mφ proliferating	0.0678	0.0251	2.71	0.00852	0.00852
Interstitial Mφ perivascular	0.0547	0.0771	0.71	0.48	0.48
Monocyte-derived Mφ	-0.188	0.282	-0.668	0.506	0.506
CD4 T cells	0.157	0.413	0.38	0.705	0.705
Classical monocytes	-0.0265	0.0779	-0.34	0.735	0.735
CD8 T cells	-0.0384	0.117	-0.328	0.744	0.744
EC general capillary	-0.00487	0.0193	-0.253	0.801	0.801
Alveolar macrophages	-0.019	0.075	-0.253	0.801	0.801
DC2	-0.0132	0.0573	-0.23	0.819	0.819
AT1	-0.00416	0.0272	-0.153	0.879	0.879
Multiciliated (non-nasal)	0.00822	0.0639	0.129	0.898	0.898
Non-classical monocytes	0.00634	0.0799	0.0794	0.937	0.937

Supplementary Table ST12. Key Resources Table

REAGENT or RESOURCE	SOURCE	IDENTIFIER
**Microbiome analysis**		
Sputum DNA collection, preservation, and isolation kit	Norgen Biotek, Canada	Catalog no. RU46100
16S rRNA-specific 341F primer	LGC Biosearch, Berlin	LGC-Berlin Germany
16S rRNA-specific 785R primer	LGC Biosearch, Berlin	LGC-Berlin Germany
MyTaq DNA polymerase	Bioline, Luckenwalde GmbH	BIO21105
BioStabII PCR Enhancer	Sigma-Aldrich Co, Berlin	Catalog no. 53833
Agencourt AMPure XP beads	Beckman Coulter, Inc., IN, USA	Catalog no. A63882
Deposited data	SRA	
**Bulk RNA-seq analysis**		
RNeasy Mini Kit	Qiagen GmbH, Hilden	Catalogue no. 74104
MinElute columns	Qiagen GmbH, Hilden Germany	Catalogue no. 28004
NEBNext Poly (A) mRNA Magnetic Isolation	New England Biolabs (NEB)	Catalogue no. E7490
DNase I	New England Biolabs (NEB)	Catalogue no. M0303S
**Single cell RNA-seq analysis**		
Chromium Next GEM Single Cell 5’ RNA library v2	10X Genomics	PN-1000265
Chromium Next GEM Single Cell V(D)J library v2	10X Genomics	PN-1000252
**CyTOF analysis**		
Human TruStain FcX™	Biolegend	422301
Formaldehyde 16%	Thermofischer scientific	28906
Benzonase nuclease	Thermofischer scientific	
MDIPA kit	Standard BioTools	201325
Anti-Human CD45 (HI30)-111Cd	Standard BioTools	3111001B
Anti-Human CD45 (HI30)-113Cd	Standard BioTools	3113001B
Anti-Human CD45 (HI30)-114Cd	Standard BioTools	3114001B
Anti-Human CD45 (HI30)-116Cd	Standard BioTools	3116001B
Anti-Human CD45 (HI30)-194Pt	Standard BioTools	3194001B
Anti-Human CD45 (HI30)-195Pt	Standard BioTools	3195001B
Anti-Human CD45 (HI30)-196Pt	Standard BioTools	3196001B
Anti-Human CD45 (HI30)-198Pt	Standard BioTools	3198001B
Maxpar Cell Staining Buffer	Standard BioTools	
Anti-human CCR6	Standard BioTools	201325 (MDIPA)
Anti-human CD123	Standard BioTools	201325 (MDIPA)
Anti-human CD19	Standard BioTools	201325 (MDIPA)
Anti-human CD4	Standard BioTools	201325 (MDIPA)
Anti-human CD8a	Standard BioTools	201325 (MDIPA)
Anti-human CD11c	Standard BioTools	201325 (MDIPA)
Anti-human CD45RO	Standard BioTools	201325 (MDIPA)
Anti-human CD45RA	Standard BioTools	201325 (MDIPA)
Anti-human CD161	Standard BioTools	201325 (MDIPA)
Anti-human CD194/CCR4	Standard BioTools	201325 (MDIPA)
Anti-human CD25	Standard BioTools	201325 (MDIPA)

Anti-human CD27	Standard BioTools	201325 (MDIPA)
Anti-human CD57	Standard BioTools	201325 (MDIPA)
Anti-human CD183/CXCR3	Standard BioTools	201325 (MDIPA)
Anti-human CD185/CXCR5	Standard BioTools	201325 (MDIPA)
Anti-human CD28	Standard BioTools	201325 (MDIPA)
Anti-human CD38	Standard BioTools	201325 (MDIPA)
Anti-human CD56/NCAM	Standard BioTools	201325 (MDIPA)
Anti-human TCRgd	Standard BioTools	201325 (MDIPA)
Anti-human CD294	Standard BioTools	201325 (MDIPA)
Anti-human CD197/CCR7	Standard BioTools	201325 (MDIPA)
Anti-human CD14	Standard BioTools	201325 (MDIPA)
Anti-human CD3	Standard BioTools	201325 (MDIPA)
Anti-human CD20	Standard BioTools	201325 (MDIPA)
Anti-human CD66b	Standard BioTools	201325 (MDIPA)
Anti-human HLA-DR	Standard BioTools	201325 (MDIPA)
Anti-human IgD	Standard BioTools	201325 (MDIPA)
Anti-human CD127/IL-7R	Standard BioTools	201325 (MDIPA)
Live/dead intercalator	Standard BioTools	201325 (MDIPA)
**48-Bioplex Assay**		
IL-1β	BIORAD	BIORAD (#12007283)
IL-1ra	BIORAD	BIORAD (#12007283)
IL-2	BIORAD	BIORAD (#12007283)
IL-4	BIORAD	BIORAD (#12007283)
IL-5	BIORAD	BIORAD (#12007283)
IL-7	BIORAD	BIORAD (#12007283)
IL-8	BIORAD	BIORAD (#12007283)
IL-9	BIORAD	BIORAD (#12007283)
IL-10	BIORAD	BIORAD (#12007283)
IL-12p70	BIORAD	BIORAD (#12007283)
IL-13	BIORAD	BIORAD (#12007283)
IL-15	BIORAD	BIORAD (#12007283)
IL-17	BIORAD	BIORAD (#12007283)
IL-1α	BIORAD	BIORAD (#12007283)
IL-3	BIORAD	BIORAD (#12007283)
IL-16	BIORAD	BIORAD (#12007283)
CTACK	BIORAD	BIORAD (#12007283)
HGF	BIORAD	BIORAD (#12007283)
Eotaxin	BIORAD	BIORAD (#12007283)
Basic FGF	BIORAD	BIORAD (#12007283)
G-CSF	BIORAD	BIORAD (#12007283)
GM-CSF	BIORAD	BIORAD (#12007283)
IFNγ	BIORAD	BIORAD (#12007283)
IP-10	BIORAD	BIORAD (#12007283)
MCP-1	BIORAD	BIORAD (#12007283)
MIP-1α	BIORAD	BIORAD (#12007283)

MIP-1β	BIORAD	BIORAD (#12007283)
PDGF-BB	BIORAD	BIORAD (#12007283)
RANTES	BIORAD	BIORAD (#12007283)
TNF-α	BIORAD	BIORAD (#12007283)
VEGF	BIORAD	BIORAD (#12007283)
IL-2Rα	BIORAD	BIORAD (#12007283)
IL-12p40	BIORAD	BIORAD (#12007283)
IL-18	BIORAD	BIORAD (#12007283)
GRO-α	BIORAD	BIORAD (#12007283)
IFN-α2	BIORAD	BIORAD (#12007283)

Supplementary Table ST13. Annotation for CD45+ clustering

Subset	Marker
B cells	$CD19^+$ $CD20^+$
$CD4^+$ T cells	$CD3^+$ $CD4^+$
$CD8^+$ T cells	$CD3^+$ $CD8^+$
Myeloid cells	$CD14^+$ $CD11c^+$
NK cells	$CD56^+$ $CD16^+$
TCRgd T cells	$CD3^+$ $TCRgd^+$

Supplementary Table ST14. Annotation for T cell clustering

Subset	Marker
$CD4^+$ naïve	$CD4^+$ $CD45RA^+$ $CCR7^+$
$CD4^+$ effector memory	$CD4^+$ $CCR7^-$ $CD45RO^+$
$CD4^+$ terminal effector	$CD4^+$ $CCR7^-$ $CD45RA^+$
Tregs	$CD4^+$ $CD127^-$ $CD25^+$
TH1-like	$CD4^+$ $CD45RO^+$ $CXCR3^+$
TH2-like	$CD4^+$ $CD45RO^+$ $CCR4^+$ $CXCR3^-$ $CCR6^-$
TH17-like	$CD4^+$ $CD45RO^+$ $CCR4^+$ $CCR6^+$
$CD8^+$ naïve	$CD8^+$ $CD45RA^+$ $CCR7^+$
$CD8^+$ effector memory I	$CD8^+$ $CCR7^-$ $CD45RO^+$
$CD8^+$ effector memory II	$CD8^+$ $CCR7^-$ $CD45RO^+$ $CD27^+$
$CD8^+$ terminal effector	$CD8^+$ $CCR7^-$ $CD45RA^+$
$CD8^+$ central memory	$CD8^+$ $CCR7^+$ $CD45RO^+$
$CD8^+$ effector	$CD8^+$ $CCR4^+$ $CCR6^+$
$CD4^-$ $CD8^-$ T cells	$CD3^+$ $CD4^-$ $CD8^-$
$TCRgd^+$ T cells	$CD3^+$ $TCRgd^+$
MAIT/NKT cells	$CD3^+$ $CD161^+$

Supplementary Table ST15. Annotation for non-T cell clustering

**Subset**	**Marker**
Classical monocytes	$CD11c^+$ $HLA\text{-}DR^+$ $CD14^+$
Non-classical monocytes	$CD11c^+$ $HLA\text{-}DR^+$ $CD16^+$
Transitional monocytes	$CD11c^+$ $HLA\text{-}DR^+$ CD14low CD16low
Myeloid dendritic cells (mDCs)	$CD11c^+$ $HLA\text{-}DR^+$
Plasmacytoid dendritic cells (pDCs)	$HLA\text{-}DR^+$ $CD123^+$
Early NK cells	$CD56^+$ $CD57^-$
Late CD16- NK cells	$CD56^+$ $CD57^-$ $CD16^-$
LateCD16+ NK cells	$CD56^+$ $CD57^+$ CD16+
B cells	$CD19^+$ $CD20^+$
IgD^+ B cells	$CD19^+IgD^+$
Plasma cells	$CD19^+$ $CD27^+$ $CD38^+$

Supplementary Table ST16. Software and algorithms used.

**Software and algorithms**		
**Microbiome analysis**		
ProGenomes2 microbial genome database	Mende et al, 2020	Reference[58]
LotuS version 1.62.	Hildebrand et al, 2014	Reference[59]
UPARSE	Edgar et al, 2013	Reference[60]
SILVA	Quast, et al, 2012	Reference[61]
GreenGenes	Molano, et al, 2024	Reference[62]
HITdb	Molano, et al, 2024	Reference[62]
RTK version 0.93.1	Saary et al, 2017	Reference [63]
DESeq2 version 1.38.3	Wirbel, J., et al, 2024	Reference [64]
metadeconfoundR version 1.0.4	Forslund et al, 2021 & 2022	Reference[65,66]
**CyTOF analysis**		
FlowSOM	Van Gassen, et al, 2015	Reference[67]
CATALYST	Nowicka M, 2019	Reference[68]
diffcyt	Weber et al., 2019	Reference[69]
BatchAdjust	Schuyler et al., 2019	Reference[70]
**48-Bioplex Assays Analysis**		
Luminex xPONENT software version 1.0		
**Bulk RNA-seq analysis**		
R version 4.3.1	R Core Team	Reference[71]
DESeq2 version 1.38.3	Elahimanesh M, 2024	Reference[72]
STAR version 2.7.9a	Du, Yuheng, 2020	Reference[73]
featureCounts version 2.0.1	Wei, 2024	Reference[74]
GENCODE version 35	Frankish, 2021	Reference[75]
clusterProfiler version 4.6.2	Lu, 2024	Reference[76]
EnhancedVolcano version 1.16.0	Guarnieri, 2024	Reference[77]
ggplot2 version 3.4.2	Wickham, 2023	Reference[78]
ComplexHeatmap version 2.16.0	Lyu, 2025	Reference[79]
FastQC	Metge BJ,2024	Reference[80]
bcl2fastq software version 2.20	Metge BJ,2024	Reference[80]
MSigDB	Metge BJ,2024	Reference[80]
GRCh38 human reference genome	Guo, Yan, 2017	Reference[81]
ENSEMBL	Dyer, Sarah C., et al, 2025	Reference[82]
ENTREZ	Kans, J., 2024	Reference[83]
**Single-cell RNA-seq analysis**		
Scanpy	Wolf, 2018	Reference[84]
Seurat version 4	Arbatsky,2025	Reference[85]
SCTransform	Ge,2025	Reference[86]
Harmony	Korsunsky, 2019	Reference[87]
UMAP	Becht,2019	Reference[88]
Leiden clustering	Netskar,2024	Reference[89]
**Bulk RNA-seq deconvolution and sensitivity Analysis**		
edgeR	Chen,2024	Reference[90]
MuSiC	Wang, X., et al, 2019	Reference[53]
HLCA (Human Lung Cell Atlas)	Sikkema, L., et al, 2023	Reference[54]
AutoGeneS	Aliee, H. and F.J. Theis, 2021, Xu, X., et al, 2025, Wolfram-Schauerte, M., et al, 2025	Reference [55-57]

Supplementary Table ST17. Sample sizes for longitudinal 16S microbiome analyses and stability stratification. A. All available 16S samples; B. Longitudinal sample availability; C. Stability strata in the paired cohort; D. Stability strata by disease group.

A. All available 16S samples

**DualStatus**	**Visit 1 samples**	**Visit 2 samples**	**Total samples**
COPD−/HIV−	50	36	86
COPD−/HIV+	50	40	90
COPD+/HIV−	50	41	91
COPD+/HIV+	50	39	89
**Total**	**200**	**156**	**356**

B. Longitudinal sample availability

**DualStatus**	**Valid paired participants**	**Visit 1 only**	**Visit 2 only**	**Participants with any microbiome sample**
COPD−/HIV−	26	24	10	60
COPD−/HIV+	28	22	12	62
COPD+/HIV−	24	26	17	67
COPD+/HIV+	23	27	16	66
**Total**	**101**	**99**	**55**	**255**

C. Stability strata in the paired cohort

**Stability group**	**Paired participants**	**Microbiome samples contributing**
Stable	34	68
Medium	34	68
Unstable	33	66
**Total**	**101**	**202**

D. Stability strata by disease group

**DualStatus**	**Stable**	**Medium**	**Unstable**	**Total paired participants**
COPD−/HIV−	12	4	10	26
COPD−/HIV+	10	11	7	28
COPD+/HIV−	4	12	8	24
COPD+/HIV+	8	7	8	23
**Total**	**34**	**34**	**33**	**101**

58 Mende, D. R. *et al.* proGenomes2: an improved database for accurate and consistent habitat, taxonomic and functional annotations of prokaryotic genomes. *Nucleic Acids Research* **48**, D621-D625 (2020).
59 Hildebrand, F., Tadeo, R., Voigt, A. Y., Bork, P. & Raes, J. LotuS: an efficient and user-friendly OTU processing pipeline. *Microbiome* **2**, 30 (2014).
60 Edgar, R. C. UPARSE: highly accurate OTU sequences from microbial amplicon reads. *Nature methods* **10**, 996-998 (2013).
61 Quast, C. *et al.* The SILVA ribosomal RNA gene database project: improved data processing and web-based tools. *Nucleic acids research* **41**, D590-D596 (2012).
62 Molano, L.-A. G., Vega-Abellaneda, S. & Manichanh, C. GSR-DB: a manually curated and optimized taxonomical database for 16S rRNA amplicon analysis. *MSystems* **9**, e00950-00923 (2024).
63 Saary, P., Forslund, K., Bork, P. & Hildebrand, F. RTK: efficient rarefaction analysis of large datasets. *Bioinformatics* **33**, 2594-2595 (2017).
64 Wirbel, J., Essex, M., Forslund, S. K. & Zeller, G. A realistic benchmark for differential abundance testing and confounder adjustment in human microbiome studies. *Genome Biology* **25**, 247 (2024).
65 Forslund, S. K. *et al.* Combinatorial, additive and dose-dependent drug–microbiome associations. *Nature* **600**, 500-505 (2021).
66 Fromentin, S. *et al.* Microbiome and metabolome features of the cardiometabolic disease spectrum. *Nature medicine* **28**, 303-314 (2022).
67 Van Gassen, S. *et al.* FlowSOM: using self-organizing maps for visualization and interpretation of cytometry data. *Cytometry Part A* **87**, 636-645 (2015).
68 Nowicka, M. *et al.* CyTOF workflow: differential discovery in high-throughput high-dimensional cytometry datasets. *F1000Research* **6**, 748 (2019).
69 Weber, L. M., Nowicka, M., Soneson, C. & Robinson, M. D. diffcyt: Differential discovery in high-dimensional cytometry via high-resolution clustering. *Communications biology* **2**, 183 (2019).
70 Schuyler, R. P. *et al.* Minimizing Batch Effects in Mass Cytometry Data. *Front Immunol* **10**, 2367, doi:10.3389/fimmu.2019.02367 (2019).
71 Team, R. C. (The Comprehensive R Archive Network, 2023).
72 Elahimanesh, M. & Najafi, M. Differentially expressed genes of RNA-seq data are suggested on the intersections of normalization techniques. *Biochem Biophys Rep* **37**, 101618, doi:10.1016/j.bbrep.2023.101618 (2024).
73 Du, Y., Huang, Q., Arisdakessian, C. & Garmire, L. X. Evaluation of STAR and Kallisto on Single Cell RNA-Seq Data Alignment. *G3 (Bethesda)* **10**, 1775-1783, doi:10.1534/g3.120.401160 (2020).
74 Wei, W. *et al.* Single-cell multiomics analysis reveals cell/tissue-specific associations in bipolar disorder. *Translational psychiatry* **14**, 323 (2024).
75 Frankish, A. *et al.* GENCODE 2021. *Nucleic acids research* **49**, D916-D923 (2021).
76 Lu, Z., Saddozai, U. A. K., Fu, S., Zhu, L. & Wang, J. Bioinformatics analysis of therapeutic targets for idiopathic pulmonary fibrosis and exploration of immune cell infiltration patterns. *Gene & Protein in Disease* **3**, 4101 (2024).
77 Guarnieri, J. W. *et al.* Mitochondrial antioxidants abate SARS-COV-2 pathology in mice. *Proceedings of the National Academy of Sciences* **121**, e2321972121 (2024).
78 Wickham, H. *et al.*
79 Lyu, J., Zhang, H., Zhong, J. & Feng, Z. The dysregulation score method identifies epigenetic regulator genes that predict cancer prognosis and efficiency of cancer immunotherapy. *Molecular Therapy Nucleic Acids* **36** (2025).
80 Metge, B. J. *et al.* Targeting EMT using low-dose Teniposide by downregulating ZEB2-driven activation of RNA polymerase I in breast cancer. *Cell Death & Disease* **15**, 322 (2024).
81 Guo, Y. *et al.* Improvements and impacts of GRCh38 human reference on high throughput sequencing data analysis. *Genomics* **109**, 83-90 (2017).
82 Dyer, S. C. *et al.* Ensembl 2025. *Nucleic acids research* **53**, D948-D957 (2025).

83 Kans, J. " Entrez Direct: e-Utilities on the UNIX Command Line". Entrez Programming Utilities Help. National Center for Biotechnology Information (US). (2024).
84 Wolf, F. A., Angerer, P. & Theis, F. J. SCANPY: large-scale single-cell gene expression data analysis. *Genome biology* **19**, 15 (2018).
85 Arbatsky, M. *et al.* Seurat function argument values in scRNA-seq data analysis: potential pitfalls and refinements for biological interpretation. *Frontiers in Bioinformatics* **5**, 1519468 (2025).
86 Ge, Q., Sheng, Y., Lu, J., Yang, Y. & Pan, M. Single-cell RNA-seq data normalization: A benchmarking study. *Plos one* **20**, e0335102 (2025).
87 Korsunsky, I. *et al.* Fast, sensitive and accurate integration of single-cell data with Harmony. *Nature methods* **16**, 1289-1296 (2019).
88 Becht, E. *et al.* Dimensionality reduction for visualizing single-cell data using UMAP. *Nature biotechnology* **37**, 38-44 (2019).
89 Netskar, H. *et al.* Pan-cancer profiling of tumor-infiltrating natural killer cells through transcriptional reference mapping. *Nature immunology* **25**, 1445-1459 (2024).
90 Chen, Y., Lun, A. T. & Smyth, G. K. Differential expression analysis of complex RNA-seq experiments using edgeR. *Statistical analysis of next generation sequencing data*, 51-74 (2014).
91 Kayongo A, Bartolomaeus TUP, Hecker J, Birkner T. Microbial Ecological Instability And Non-Linear Host Interactions Shape Airway Inflammation In HIV-Associated COPD. *Zenodo*; (2026) Jul. doi:10.5281/zenodo.21473246
