# Supplementary material for: Microbial ecological instability and non-linear host interactions shape airway inflammation in HIV-associated COPD
Source: Nat Commun. 2026 Oct 6;17:11543. doi: 10.1038/s41467-026-78400-4 (PMC13748602; doi:10.1038/s41467-026-78400-4)
Supplement: Supplementary file 2 [file 41467_2026_78400_MOESM2_ESM.pdf]

nature portfolio

Peer Review File

Microbial Ecological Instability And Non-Linear Host Interactions Shape Airway Inflammation In HIV-Associated COPD.

Corresponding Author: Dr Alex Kayongo

This file contains all reviewer reports in order by version, followed by all author rebuttals in order by version.

Version 0:

Reviewer comments:

Reviewer #1

(Remarks to the Author)
In this manuscript, Alex et al. perform a longitudinal multi-omics analysis investigating the relationship between microbial ecological instability and airway inflammation in HIV-associated COPD. The authors integrate multiple layers of data, including the airway microbiome, outer membrane vesicles (OMVs), cytokines, and bronchoalveolar lavage fluid (BALF) cell populations. They conclude that HIV-associated COPD reflects immune-skewed mucosal remodeling, wherein the airway microbiome acts as a selective, temporally dynamic modulator of inflammatory states rather than a globally disrupted community.
While this study addresses an important question regarding the interactions between the respiratory microbiome and the immune status in a specific clinical population, the manuscript suffers from several significant limitations. Specifically, the small sample size, the reliance on 16S rRNA sequencing rather than higher-resolution metagenomics, and the over-reliance on simple correlational analyses lead to an overstatement of the main conclusions. The majority of the findings (e.g., correlations between cytokines/immune cells and microbiota) are purely associative, without sufficient exploration of their clinical significance or underlying biological mechanisms. Furthermore, the lack of an independent validation cohort severely undermines the reliability of the conclusions. My major and minor concerns are detailed below.

Major Concerns:
1. Lines 171-173, Figure 3A: Were the samples from the two visits collected using the exact same Standard Operating Procedures (SOPs) and sequenced in the same batch? The authors need to rule out the possibility that the observed temporal shifts are artifacts of a batch effect. Furthermore, how do the authors explain the significant decline in richness observed in the control group (COPD-/HIV-)? If these are healthy individuals, there is no apparent biological rationale for such a drastic decrease.
2. Lack of Validation and Mechanistic Insight: The study utilizes a small sample size without validation in an independent, larger cohort. For instance, Pages 251-254 present associations between the microbiota and specific cytokines—how do the authors interpret these associations? Do they hold any clinical significance? Similarly, Pages 265-269 detail correlations between immune cells and microbiota. The manuscript lacks an explanation on the clinical relevance or the biological mechanisms driving these observations.
3. Pages 261-285: I am particularly interested in the impact of HIV infection on immune cell populations and their relationship with the microbiota. The authors should expand their analysis and discussion on this specific interplay.
4. Pages 286-303: It is unsurprising that gene expression differs significantly between the dual-positive (COPD+/HIV+) and dual-negative (COPD-/HIV-) groups, given that HIV infection itself robustly activates these immune responses. To accurately assess the specific impact of HIV infection on COPD patients, the more appropriate comparison would be between the COPD+/HIV+ group and the COPD+/HIV- group.
5. Pages 321-324: Statements such as "discrete microbial shifts, rather than overall diversity, may influence local immune tone" and "suggested that discrete microbial or microbial-derived activities may drive airway immune tone independently of global diversity" are overly definitive and overstated. The evidence provided is based solely on simple correlations within a small cohort, lacking support from independent validation cohorts or robust longitudinal/temporal causal evidence.
6. Rationale for OMV Analysis: Why do the authors assume that "OMVs contribute additional transcriptional modulation"? This premise is not convincing. Numerous other microbial factors, including cell wall components and potential metabolites, can equally modulate the immune system. Furthermore, the authors do not identify which bacterial taxa these OMVs

originate from. This omission weakens the logic and relevance of the subsequent downstream analyses linking OMVs to immune cells.
7. Pages 355-356: I strongly question the conclusion that "Pairwise PERMANOVA and univariate ANCOVA similarly detected no disease-dependent cell-type differences after FDR correction." Both HIV and COPD are diseases known to profoundly impact immune cell populations and their activation states. The assertion that there are no cell-type differences between these patients and healthy controls contradicts established pathophysiological facts.
8. Pages 428-430: The conclusion stating "Together, these findings show that airway mucosal gene expression is shaped far more by microbial community structure than by HIV or COPD status alone..." is unsupported. The manuscript only provides simple associative analyses, which cannot establish causality. It is impossible to determine from these data whether the microbial community structure is the cause or the consequence of the altered mucosal gene expression.
9. Figure Consolidation: The inclusion of 12 main figures is excessive. The authors should consider merging related panels to create fewer, more comprehensive multi-panel figures.

Minor Concerns:
1. Abstract: The abstract should explicitly state the sample size (number of human subjects) included in the analysis.
2. Figure 1: This figure appears to be an AI-generated illustration. It is overly complex and does not seem highly relevant to the specific scientific content of the paper.
3. Figure 3: For Figure 3B, a statistical analysis (e.g., ADONIS/PERMANOVA) should be performed and reported. For Figure 3C, is the observed difference statistically significant? Please provide the appropriate p-values.

(Remarks on code availability)

Reviewer #2

(Remarks to the Author)
This ambitious manuscript integrates sputum microbiome, transcriptomics, and immune cell profiling to understand HIV-associated COPD. The authors should be commended for building one of the most deeply phenotyped HIV lung cohorts utilizing an array of sequencing platforms. Their longitudinal approach is unique and extremely valuable to better understand disease pathogenesis in this population. Also, this cohort brings forth new data from HIV populations living in Africa which have often been overlooked in HIV lung studies.

The authors found that 1) time influences the sputum microbiome more so than disease status; 2) certain genera like Haemophilus and Sphingomonas drive microbiome instability in disease-specific ways; 3) bacterial-derived OMVs have little relationship between disease states and microbiome structures; 4) there were few microbiome-cytokine interactions; 5) certain microbial features are associated in the blood with both T-cell and non-T-cell populations; 6) HIV+/COPD+ is associated with numerous transcriptionally dysregulated genes in sputum, with only a few of these genes associated with microbial taxa; 7) OMVs have a subtle influence on sputum transcriptomics; 8) HIV has a stronger effect on sputum gene expression compared to COPD, with this effect observed less in BAL; 9) Sputum transcriptomics are influenced more by specific microbes rather than by global microbial disruptions or disease status.

I think there are a few limitations to the work that need to be addressed. First, my impression from this paper is that ultimately the microbiome does not really explain the pathogenesis of dual disease status. If anything, the microbiome's influence on sputum transcriptomics and sputum cell composition are subtle. While the transcriptome may be substantially dysregulated in HIV+COPD+, I am less convinced that dysbiotic features are the driver of this based on the data. OMVs for example appear to exert little influence on disease outcomes here. Therefore, as an explanation for HIV+COPD+, I think the authors do not necessarily substantiate their hypothesis. If the influence of disease status on the microbiome or the host response is minimal, then the next question is what is the significance of these findings?

Second, I think a major gap is the use of a deconvoluted bulk to single cell RNA sequencing pathway that lacks necessary detail. I question the validity of their approach and further substantiation that these methods are robust is required before many of the downstream analyses generated from this deconvolution step are to be believed.

Methods
1. The definition of COPD utilized in this study should be provided.
2. Line 637: sentence appears to be cut off.
3. The scRNASeq analysis only comes from 4 participants. Even for a scRNASeq analysis, this number is quite low. With only 5205 cells reported, I would find this analysis very difficult to interpret. Can the authors justify this sample size? If only 4 participants are then used to inform the bulk seq deconvolution, this could lead to significant downstream bias. How were these deconvolution methods ultimately validated? One would want to see the methods applied to a separate matched scRNAseq-bulk seq dataset. Greater detail needs to be provided as to how these deconvolution methods were obtained.

Results
1. Table 1. Further clarification on the other category in Biomass exposure would be helpful. Inhaled prednisone use is confusing – do you mean oral prednisone or inhaled corticosteroids? Please provide FVC % predicted.
2. Much is made in the first part of the results of this concept of microbiome instability. Is this a microbiome feature, though, that is clinically important? i.e. does someone with microbiome instability over time have worse lung function outcomes, or disease outcomes?

3. Figure 6D could be clarified. It is hard to know which of the plots belong to which low/medium/high category and the colors should be explicitly defined in the legend.
4. Lines 247-248 and Figure 7. This needs to be clarified. I'm confused about the statement that HIV-negative status was associated with reduced IL-12p40 and IL2. In the figure. It looks as though HIV status (which I interpreted as HIV+) is more negative (green) compared to HIV-negative. The text and the figure don't seem congruent and it may mean that you need to specifically state what you mean by HIV status in the figure.
5. Lines 304-306. I think the statement here that the links between airway dysbiosis and inflammatory transcription is a "strong association" is a reach. One sees many transcription disruptions in dual disease status, but many of these are not explained by the microbial taxa.
6. Minor point: on Figure 11 many people would not know what Ekyoto is so it should be defined in the legend.
7. Demographic tables should be provided of the subsets who underwent bronchoscopy, CyTOF analysis, Bioplex assay, and bulk RNASeq. Were these subsets representative of the larger cohort?
8. As a stylistic point, as you have both sputum and BAL samples reported here, it can be confusing to the reader which compartment is referred to when the only term used is "airway" which could refer to either compartment. Would clarify in the figure legends and in the text which samples are being addressed.

Discussion
1. Lines 438-441. I'm not sure you've shown that restoration of mucosal immunity has attenuated the magnitude of lung function decline (i.e. you don't have a comparison group where immune reconstitution has not occurred or you don't have pre-ART lung function trajectory).

(Remarks on code availability)

Version 1:

Reviewer comments:

Reviewer #1

(Remarks to the Author)
This revised manuscript has addressed most of the issues I previously raised and included additional analyses, thereby strengthening the reliability of the conclusion that "HIV-associated COPD is characterized primarily by localized immune-transcriptional remodeling accompanied by selective host-microbe interactions, rather than widespread restructuring of the airway microbiome". However, before the conclusions can be accepted, the authors must address the critical issues of the reproducibility of their analytical results and the "Data and Code Availability", which, in its current state, is not qualified.
As noted in my previous comments, the sample size of this study is relatively small, especially for certain assays such as the BALF scRNA-seq, which only includes 4 cases. Furthermore, the main conclusions of the article rely on complex, multi-layered statistical analyses without independent cohort validation or experimental verification. Therefore, an essential way to evaluate the validity of the conclusions is through the public availability of the data and analytical code, including the availability of the input files for the code. However, upon reviewing the GitHub repository provided by the authors, I found only a few lines of code with no annotations. The repository fails to provide abundance tables for the transcriptome, cytokines, and microbiome, nor does it provide patient metadata (patient privacy information can be removed, but unique identifiers are needed to link the omics data with patient grouping information). There is a complete absence of the formulas used for core analyses, such as differential gene expression analysis, deconvolution analysis, and various statistical analyses, as well as the crucial input files for these analyses. Without the public availability of these codes and data, it is exceedingly difficult for readers to evaluate the reliability of this paper.
Other Comments:
1. Figure 1: The study design figure should clearly state and illustrate the exact timeline of the longitudinal follow-up.
2. Figure 3B: In the PCoA plot, different shape morphologies (e.g., triangles and squares) should be used to represent HIV+/HIV-, and empty or filled shapes should be used to represent COPD+/COPD-, to simultaneously reflect the grouping characteristics of both HIV and COPD.
3. Lines 180-181 and Figure 3C: The text states, "Despite this temporal turnover, within-individual microbial communities remained consistently more similar to themselves over time than to those of other participants, indicating preservation of individual-specific microbial signatures". However, Figure 3C shows that the BC distance within the "same individual" is higher than that in "different individuals," which implies the similarity within the same individual is lower than that between different individuals. This contradicts the conclusion drawn in the text.
4. Line 289: This section suggests that the lack of differences in peripheral immunity associated with HIV status might be related to viral suppression in the patients. Since 86% of the patients in this cohort achieved virological suppression, does this mean that 14% were not effectively suppressed? If so, are there any differences in the peripheral immune-cell composition and sputum inflammatory cytokines between the 86% virologically suppressed patients and the 14% unsuppressed patients?
5. Lines 342-344: "we performed confounder-aware transcriptomic analyses of induced sputum, bronchoalveolar lavage (BAL), and peripheral blood mononuclear cells (PBMCs) (Figure 5)". Please specify the exact number of samples included for each group within each compartment category.
6. Line 365: "Selective microbial features align with coordinated airway transcriptional programmes". Consider visualizing the results of this section and placing them in the supplementary figures.
7. Lines 941-989: This section involves numerous subgroup classifications and inter-group difference analyses, such as the

stability stratification. In these analyses, the sample size is crucial to the reliability of the results. Please explicitly state the sample size included for each group in each type of analysis, which could be listed as "(n=x)" next to each group.

(Remarks on code availability)
This code is completely inadequate for evaluating the reliability of the paper. Only a few trivial snippets of code have been uploaded, and it lacks a README file, the core analytical code of the paper, and the input source data used for the analyses. It lacks any reproducibility whatsoever.

Reviewer #2

(Remarks to the Author)
I appreciate the efforts by the authors to shift the messaging of the paper away from the microbiome and more towards transcriptional dysregulation. I am still puzzled though by the deconvolution methods. The authors state validation using the Human Lung Cell Atlas but when I review Supplementary figures 17B and 17C, there appears to be quite substantial differences in cell proportions. I still find this aspect of the paper to have questionable validity.

(Remarks on code availability)

Version 2:

Reviewer comments:

Reviewer #1

(Remarks to the Author)
The authors have addressed my previous comments regarding code availability and the reproducibility of the results. I have no further comments.

(Remarks on code availability)

Reviewer #2

(Remarks to the Author)
Thank you for the clarification and the further description of the limitations of your methods when deconvoluting the single cell data.

(Remarks on code availability)

Open Access This Peer Review File is licensed under a Creative Commons Attribution 4.0 International License, which permits use, sharing, adaptation, distribution and reproduction in any medium or format, as long as you give appropriate credit to the original author(s) and the source, provide a link to the Creative Commons license, and indicate if changes were made.
In cases where reviewers are anonymous, credit should be given to 'Anonymous Referee' and the source.
The images or other third party material in this Peer Review File are included in the article's Creative Commons license, unless indicated otherwise in a credit line to the material. If material is not included in the article's Creative Commons license and your intended use is not permitted by statutory regulation or exceeds the permitted use, you will need to obtain permission directly from the copyright holder.
To view a copy of this license, visit https://creativecommons.org/licenses/by/4.0/

HELMHOLTZ

MDC MAX-DELBRÜCK-CENTRUM FÜR MOLEKULARE MEDIZIN IN DER HELMHOLTZ-GEMEINSCHAFT

MDC • Robert-Rössle-Straße 10 • 13125 Berlin

Dr. Sofia Forslund
Wirt-Mikrobiom Faktoren in Herz-Kreislauferkrankungen

Robert-Rössle-Straße 10
13125 Berlin

Tel.: +49 (0)30 450540649
Fax: +49 (0)30 450540945

sofia.forslund@mdc-berlin.de
www.mdc-berlin.de

July 21, 2026

Nature Communications
Springer Nature

Dear Editor,
We are pleased to submit our revised manuscript NCOMMS-26-019313, entitled "***Microbial Ecological Instability and Non-Linear Host Interactions Shape Airway Inflammation in HIV-Associated COPD***" for consideration as an Article in Nature Communications. We have extensively revised the manuscript as per reviewers' comments. Please find point-by-point responses.

Reviewer #1 (Remarks to the Author):
In this manuscript, Alex et al. perform a longitudinal multi-omics analysis investigating the relationship between microbial ecological instability and airway inflammation in HIV-associated COPD. The authors integrate multiple layers of data, including the airway microbiome, outer membrane vesicles (OMVs), cytokines, and bronchoalveolar lavage fluid (BALF) cell populations. They conclude that HIV-associated COPD reflects immune-skewed mucosal remodeling, wherein the airway microbiome acts as a selective, temporally dynamic modulator of inflammatory states rather than a globally disrupted community. While this study addresses an important question regarding the interactions between the respiratory microbiome and the immune status in a specific clinical population, the manuscript suffers from several significant limitations. Specifically, the small sample size, the reliance on 16S rRNA sequencing rather than higher-resolution metagenomics, and the over-reliance on simple correlational analyses lead to an overstatement of the main conclusions. The majority of the findings (e.g., correlations between cytokines/immune cells and microbiota) are purely associative, without sufficient exploration of their clinical significance or underlying biological mechanisms. Furthermore, the lack of an independent validation cohort severely undermines the reliability of the conclusions. My major and minor concerns are detailed below.

Response:
We thank the reviewer for bringing this to our attention.
Sample size issue: A sample size and power estimation was included in the methods section of our manuscript. We estimated sample size based on taxonomic profiles derived from Human Microbiome Project (HMP) data. A conservative approximation of our power to detect microbiome associations at the genus level between groups was tested using a t-test. At baseline (visit 1), given n = 50 individuals per group, assuming an effect size of 0.8 (Cohen's d) and an alpha threshold of 0.05/30, reflecting a Bonferroni correction for the 30 main airway bacterial genera, power was approximately 80% to conclude significance for each accurate microbial biomarker. Clinically relevant microbial feature abundance effects that fall within the convention of a "large" effect size Cohen's d ≥ 0.8 are commonly used to define clinically relevant microbial abundance changes. We agree that several high-dimensional modalities, particularly microbiome analysis, bulk and single-cell RNA sequencing and CyTOF, were

performed on subsets of the cohort because of sample availability and the logistical constraints of deep multi-omic profiling. We have revised the manuscript to better acknowledge this limitation. However, the central conclusions do not rely on any single dataset. Rather, they are supported by convergent evidence across multiple independent platforms, including longitudinal microbiome profiling, cytokine measurements, CyTOF immune phenotyping, bulk transcriptomics, and BAL-guided deconvolution, each identifying consistent patterns of immune remodeling and taxon-specific host interactions.

Reliance on 16S rRNA sequencing rather than higher-resolution metagenomics. We agree that shotgun metagenomic sequencing provides higher taxonomic and functional resolution than 16S rRNA amplicon sequencing and would enable strain-level characterization together with direct inference of microbial functional capacity. We have revised the Discussion accordingly and now explicitly state that the taxon-specific associations identified in this study should be interpreted at the genus level and that future metagenomic, metatranscriptomic, and metabolomic studies will be required to define the underlying functional mechanisms. We also note, however, that induced sputum represents a low-biomass respiratory specimen with a high proportion of host DNA, making shotgun metagenomic sequencing technically challenging and often substantially reducing microbial sequencing depth without extensive host-DNA depletion. Under these circumstances, 16S rRNA amplicon sequencing remains a widely used and appropriate approach for robust characterization of bacterial community structure in airway microbiome studies. Because the primary objective of our study was to define longitudinal ecological organization and integrate bacterial community profiles with immune and transcriptomic phenotypes, rather than to identify strain-level functions, 16S sequencing provided an appropriate platform for addressing the study aims. To acknowledge this limitation explicitly, we have revised our limitation section as follows. "Bacterial profiling was performed using 16S rRNA amplicon sequencing, which provides genus-level resolution but does not capture strain-level diversity or microbial functional capacity. While 16S sequencing remains well suited to low-biomass respiratory specimens such as sputum, future host-DNA-depleted metagenomic, metatranscriptomic, and metabolomic studies will be important for defining the functional mechanisms underlying the interaction networks identified here."

Over-reliance on simple correlational analyses lead to an overstatement of conclusions. We thank the reviewer for this observation. We clarify that correlation analyses were used as an initial exploratory step. The principal findings are further supported by multiple complementary statistical frameworks, including longitudinal paired analyses, mixed-effects models, generalized additive models, aligned rank transform interaction models, PERMANOVA, multivariable regression, and confounder-aware modeling. We have revised the Results and Methods to distinguish exploratory association analyses more clearly from the hypothesis-driven inferential analyses that underpin our conclusions.

The majority of the findings (e.g., correlations between cytokines/immune cells and microbiota) are purely associative, without sufficient exploration of their clinical significance or underlying biological mechanisms. We thank the reviewer for this valuable comment. We agree that the original manuscript focused primarily on reporting statistically significant associations and did not sufficiently discuss their biological or clinical significance. We have substantially revised both the Results and Discussion to provide a more balanced biological interpretation while avoiding causal overstatement.

For the cytokine analyses, we now emphasize that the principal finding is not widespread microbial regulation of airway inflammation, but rather that only a restricted number of bacterial genera remained associated with specific inflammatory mediators after rigorous confounder adjustment. In particular, Corynebacterium showed reproducible inverse associations with IL-6, MIF, RANTES, PDGF-BB, IL-1Ra and IL-4, while Neisseria was selectively associated with IL-12p40. We interpret these findings as evidence that discrete microbial community states align with selective inflammatory pathways rather than generalized cytokine activation. Because these associations persisted after adjustment for HIV status, COPD, demographic variables and other measured confounders, they identify biologically coherent host-microbe association patterns. However, we now explicitly state that these findings do not establish direct microbial regulation of cytokine production, and instead represent hypothesis-generating observations requiring mechanistic validation.

Similarly, we substantially expanded the interpretation of the CyTOF analyses. Rather than simply presenting microbe-immune correlations, we now highlight that several immune populations associated with airway microbial features, including Th2-like cells, effector-memory T cells, non-classical monocytes and early NK cells, also showed significant associations with post-bronchodilator FEV_1/FVC. This provides important clinical context by linking microbial features with immune phenotypes associated with airflow limitation. At the same time, we emphasize that these analyses identify host-microbe-immune interaction modules, not causal pathways. The observed relationships may reflect microbial influences on immunity, immune-mediated ecological selection, or shared responses to other biological processes that cannot be distinguished in an observational study.

We have also expanded the Discussion to place these findings within the context of a predominantly ART-treated and virologically suppressed HIV cohort. We now discuss that the dominant biological signal was selective functional immune remodeling rather than generalized immune activation, consistent with contemporary understanding of treated HIV infection. Accordingly, we interpret the cytokine and CyTOF findings as evidence that specific microbial states reproducibly align with defined inflammatory and immune programmes, while acknowledging that experimental perturbation studies will be required to establish the underlying mechanisms. To reflect this revised interpretation, we have modified the relevant sections of the Results and Discussion throughout the manuscript. The revised text consistently replaces mechanistic language with terms such as "associated with," "aligned with," "host-microbe interaction modules," and "hypothesis-generating," thereby providing biological context without overstating causal inference.

My major and minor concerns are detailed below.
Major Concern 1: Lines 171-173, Figure 3A: Were the samples from the two visits collected using the exact same Standard Operating Procedures (SOPs) and sequenced in the same batch? The authors need to rule out the possibility that the observed temporal shifts are artifacts of a batch effect.

Response:
We thank the reviewer for raising this important point. Both visits were processed using identical laboratory SOPs and sequenced in two runs three years apart (2020, 2023), using identical primers, DNA input, and pooling strategy. Raw reads from both runs were pooled and processed jointly through a single bioinformatic pipeline, including a common rarefaction depth and filtering thresholds applied to the combined dataset (see Methods). We agree that the main effect of visit is structurally confounded with sequencing run. However, all primary disease-related inferences rely on the visit × DualStatus interaction, which is estimable and robust to any additive run-level shift, since such a shift is shared across all four disease groups and cancels in the interaction contrast. Using COPD⁻/HIV⁻ participants as sentinels, we found a significant compositional shift between visits (PERMANOVA, R^2 = 0.091, p = 0.001), characterised by two genera increasing (*Atopobium, Actinomyces*) and ten decreasing (including *Prevotella, Haemophilus, Campylobacter, Veillonella, Megasphaera*; full list in Supplementary Table SR1). Linear mixed models testing whether this shift differed by disease group found no evidence that it did (bloomer interaction: χ^2=6.64, df=3, p=0.084; decreaser interaction: χ^2=6.82, df=3, p=0.078), indicating a cohort-wide temporal pattern rather than a disease-specific one (Supplementary Figure SR2). We therefore included *bloomer* and *decreaser* abundance scores as covariates in all per-genus mixed models. Two complementary checks confirmed that visit did not distort our disease-group findings. First, testing whether each genus's Visit 1→Visit 2 change differed by disease group (96 genera passing our abundance filter; 288 visit × DualStatus interaction tests) identified no significant interactions after FDR correction (Supplementary Table SR4; p-value distribution in Supplementary Figure SR3), indicating that where genera changed over time, they did so similarly across all disease groups rather than in a disease-specific manner.

Second, as an independent check in the opposite direction, whether disease-group associations were themselves artifacts of visit, metadeconfoundR analysis performed separately within each DualStatus pairing (COPD⁻/HIV⁻ vs. each disease group; 125 genera × 3 pairings = 375 tests) did not identify visit as a confounder for any tested disease-group association (Supplementary Figure SR5, Supplementary Table SR5). Together, these results indicate that visit-driven temporal drift neither produced disease-specific trajectories nor accounted for the disease-group differences reported in this study. We have added these new analyses in our manuscript results, methods and discussion sections.

How do the authors explain the significant decline in richness observed in the control group (COPD-/HIV-)? If these are healthy individuals, there is no apparent biological rationale for such a drastic decrease.

Response: We thank the reviewer for this observation. The decline in alpha diversity in COPD⁻/HIV⁻ controls is statistically robust, taxonomically characterised, and biologically explicable given the characteristics of this cohort. We address each dimension below.

<u>Cohort context</u>. Our participants are adults aged 50-80 years, resident in Uganda, followed over a 2-year interval. This is not a stable, low-exposure Western population. Background infectious burden, antibiotic exposure, dietary shifts, and progressive immune senescence over a 2-year interval are all plausible drivers of respiratory microbiome change even in individuals who remain clinically classified as healthy by spirometry criteria and HIV-seronegative status at both visits. To our knowledge, published longitudinal data on respiratory microbiome aging trajectories specifically in Sub-Saharan African adults over 50 are not yet available; our findings represent novel longitudinal evidence in this population context.

<u>Taxonomic characterisation of the shift</u>. We characterised the visit-associated compositional shift using COPD⁻/HIV⁻ participants as internal sentinels (Supplementary Table SR1). Taxa that declined between visits

included *Prevotella, Haemophilus, Aggregatibacter, Campylobacter, and Veillonella*, canonical members of the healthy lower respiratory and oral microbiome. Taxa that increased included *Actinomyces, Atopobium, Abiotrophia, and Shuttleworthia*, oral anaerobes associated with age-related changes in oral ecosystem composition and reduced mucosal immune clearance. This pattern is biologically coherent with known aging trajectories of the oral-respiratory microbiome (Liu et al. 2020, doi:10.18632/aging.103108; Maciejewska et al. 2019, PMID 30308473). Critically, none of the increasing taxa are consistent with reagent or environmental contamination, which characteristically produces environmental Proteobacteria absent from the human respiratory tract (Salter et al. 2014, doi:10.1186/s12915-014-0087-z).

<u>Statistical confirmation in healthy controls</u>. Alpha diversity (Shannon index) declined significantly in paired healthy controls between visits (paired t-test: $t=-2.96$, $df=25$, $p=0.007$; mean difference=−0.43, 95% CI: −0.73 to −0.13; n=26). In the full control group adjusted for age, the LMM estimated a visit coefficient of −0.49 (SE=0.10, $p=2.4\times10^{-6}$); age was independently and positively associated with baseline diversity ($\beta=+0.015$, $p=0.005$). Critically, this decline remained significant after adjustment for the drift covariates identified in our batch-effect analysis (bloomer_sum and decreaser_sum): the visit coefficient attenuated to −0.33 (SE=0.13, $p=0.012$) and the age effect remained significant ($\beta=+0.012$, $p=0.020$; Supplementary Table SR6). This ~33% attenuation indicates that a portion of the raw signal reflects the shared compositional drift already characterised in our response to Major Concern 1, while a substantial residual decline persists after this adjustment. The finding is therefore not an artifact of the visit-associated technical/compositional shift.

<u>Uniformity across disease groups is evidence against technical artifacts</u>. A likelihood ratio test found no evidence of group-specific trajectories ($\chi^2=0.94$, $df=3$, $p=0.816$; Supplementary Table SR6). The decline was statistically indistinguishable across all four groups, a pattern with no plausible technical explanation but consistent with a shared biological aging process operating under common demographic and environmental conditions.

<u>Analytical control</u>. The visit-associated compositional shift was accounted for in all longitudinal analyses via bloomer/decreaser covariates in the per-genus mixed models (see response to Major Concern 1), preferable to data-level batch correction, which would require unverifiable assumptions about which groups are biologically stable (Leek et al. 2010, Nat Rev Genet).

<u>Manuscript changes</u>: We have incorporated these changes in results and discussions sections of our manuscript.

Major Concern 2. Lack of Validation and Mechanistic Insight: The study utilizes a small sample size without validation in an independent, larger cohort.

Response: Validation remains as a study limitation; we will acknowledge the absence of an independent validation cohort and revise the manuscript to frame findings as internally consistent, multi-layered associations requiring external validation.

For instance, Pages 251-254 present associations between the microbiota and specific cytokines, how do the authors interpret these associations? Do they hold any clinical significance? Similarly, Pages 265-269 detail correlations between immune cells and microbiota. The manuscript lacks an explanation on the clinical relevance or the biological mechanisms driving these observations.

Response: We thank the reviewer for this valuable comment. We agree that the original manuscript focused primarily on reporting statistically significant associations and did not sufficiently discuss their biological or clinical significance. We have substantially revised both the Results and Discussion to provide a more balanced biological interpretation while avoiding causal overstatement.

For the cytokine analyses, we now emphasize that the principal finding is not widespread microbial regulation of airway inflammation, but rather that only a restricted number of bacterial genera remained associated with specific inflammatory mediators after rigorous confounder adjustment. In particular, *Corynebacterium* showed reproducible inverse associations with IL-6, MIF, RANTES, PDGF-BB, IL-1Ra and IL-4, while *Neisseria* was selectively associated with IL-12p40. We interpret these findings as evidence that discrete microbial community states align with selective inflammatory pathways rather than generalized cytokine activation. Because these associations persisted after adjustment for HIV status, COPD, demographic variables and other measured confounders, they identify biologically coherent host-microbe association patterns. However, we now explicitly state that these findings do not establish direct microbial regulation of cytokine production, and instead represent hypothesis-generating observations requiring mechanistic validation.

Similarly, we substantially expanded the interpretation of the CyTOF analyses. Rather than simply presenting microbe-immune correlations, we now highlight that several immune populations associated with airway microbial

features, including Th2-like cells, effector-memory T cells, non-classical monocytes and early NK cells, also showed significant associations with post-bronchodilator FEV_1/FVC. This provides important clinical context by linking microbial features with immune phenotypes associated with airflow limitation. At the same time, we emphasize that these analyses identify host-microbe-immune interaction modules, not causal pathways. The observed relationships may reflect microbial influences on immunity, immune-mediated ecological selection, or shared responses to other biological processes that cannot be distinguished in an observational study.

We have also expanded the Discussion to place these findings within the context of a predominantly ART-treated and virologically suppressed HIV cohort. We now discuss that the dominant biological signal was selective functional immune remodeling rather than generalized immune activation, consistent with contemporary understanding of treated HIV infection. Accordingly, we interpret the cytokine and CyTOF findings as evidence that specific microbial states reproducibly align with defined inflammatory and immune programmes, while acknowledging that experimental perturbation studies will be required to establish the underlying mechanisms. To reflect this revised interpretation, we have modified the relevant sections of the Results and Discussion throughout the manuscript. The revised text consistently replaces mechanistic language with terms such as "associated with," "aligned with," "host-microbe interaction modules," and "hypothesis-generating," thereby providing biological context without overstating causal inference.

Major concern 3. Pages 261-285: I am particularly interested in the impact of HIV infection on immune cell populations and their relationship with the microbiota. The authors should expand their analysis and discussion on this specific interplay.

Response: We thank the reviewer for the interest in this specific interplay. To address this comment directly, we performed four additional metadeconfoundR analyses restricted to two-group subsets of the cohort, each isolating the HIV or COPD contrast against either healthy controls or double-positive participants: (i) HIV−/COPD− vs HIV+/COPD−, (ii) HIV−/COPD− vs HIV−/COPD+, (iii) HIV−/COPD+ vs HIV+/COPD+, and (iv) HIV+/COPD− vs HIV+/COPD+. Within each subset, we tested associations between immune cell populations (CyTOF) and microbial genera, as well as age, sex, BMI, and lung function metrics. Microbial genera were excluded as potential confounders. No immune cell population reached significant association with HIV status itself in any of the four subsets. The associations that did reach significance were with COPD-related variables (post-bronchodilator FEV_1, FEV_1/FVC ratio, COPD status) rather than with the HIV variable, and specific microbial genera (notably *Proteobacteria Sphingomonas*, Bacteroidota Prevotella, Firmicutes Megasphaera) associated with defined T cell subsets in some subsets but did so in a manner not attributable to HIV status.

We interpret this null result cautiously and consider two contributing factors. First, all HIV-positive participants in this cohort were virologically suppressed on antiretroviral therapy, a clinical state known to substantially normalise peripheral and mucosal immune populations. Only a subtle residual HIV-specific immunological signal is therefore expected. Second, sample sizes in the two-group subsets (n ~ 40-50 per subset for CyTOF × microbiota) are modest for detecting more subtle associations. Together, these considerations mean that the null observed here is consistent with, but does not prove, an absence of HIV-microbiota-immune interaction; it reflects both the well-controlled clinical status of this cohort and the statistical limits of the subset design. We have added an explicit sentence in the Results and a short interpretive paragraph in the Discussion and provide the four-subset heatmap for the Reviewer to inspect (Supplementary Figure for Reviewer SR10).

Major comment 4. Pages 286-303: It is unsurprising that gene expression differs significantly between the dual-positive (COPD+/HIV+) and dual-negative (COPD-/HIV-) groups, given that HIV infection itself robustly activates these immune responses. To accurately assess the specific impact of HIV infection on COPD patients, the more appropriate comparison would be between the COPD+/HIV+ group and the COPD+/HIV- group.

Response: We thank the reviewer for this important suggestion. We agree that isolating the HIV effect within COPD-positive participants is a more specific test than comparing the dual-positive to the dual-negative group. We therefore repeated our confounder-aware metadeconfoundR analysis of gene expression restricted to COPD-positive participants, contrasting COPD+/HIV+ against COPD+/HIV-, for each of the three transcriptomic compartments (sputum, BAL, PBMC).

<u>Sputum RNA</u>. Of 1,127 genes tested for HIV status within COPD-positive participants, roughly a third remained significant at BH-adjusted q<0.1 and not confounded by any of the other included metavariables. Full results are provided as Supplementary Table SR11-13 for the reviewers only.

<u>BAL and PBMC RNA</u>. The same analysis in BAL and PBMC yielded zero significant HIV-associated gene-expression associations at q<0.1 (0/1,163 features tested in BAL; 0/1,271 in PBMC). We attribute this to sample size: subsetting to COPD-positive participants substantially reduces the effective n for these compartments, which had smaller baseline cohorts than sputum. These null results are consistent with underpowered detection rather than evidence

of no biological effect. We have retained the BAL and PBMC subset results as Supplementary Tables SR12 and SR13 for transparency, but do not interpret them substantively. We have added a paragraph to the Results and discussion sections describing this subset analysis, interpreting the sputum finding and acknowledging the BAL/PBMC underpowering

Supplementary Table for Reviewers SR11: metadeconf_COPD_pos_sputumRNA.tsv; Supplementary Table for Reviewers SR12: metadeconf_COPD_pos_PBMCRNA.tsv and Supplementary Table for Reviewers SR13: metadeconf_COPD_pos_BALRNA.tsv).

Major comment 5. Pages 321-324: Statements such as "discrete microbial shifts, rather than overall diversity, may influence local immune tone" and "suggested that discrete microbial or microbial-derived activities may drive airway immune tone independently of global diversity" are overly definitive and overstated. The evidence provided is based solely on simple correlations within a small cohort, lacking support from independent validation cohorts or robust longitudinal/temporal causal evidence.

Response: We thank the reviewer for this important comment and agree that our original wording could be interpreted as implying causality beyond what our observational data support. We have therefore revised the manuscript throughout to distinguish clearly between association and mechanism. Specifically, we no longer state that discrete microbial taxa or microbial-derived activities drive airway immune tone. Instead, the revised Results consistently describe these findings as selective host-microbe associations or host-microbe interaction modules, emphasizing that specific bacterial taxa were associated with inflammatory cytokines, immune-cell populations, and transcriptional programmes after adjustment for measured clinical confounders, while explicitly avoiding causal language.

Major comment 6. Rationale for OMV Analysis: Why do the authors assume that "OMVs contribute additional transcriptional modulation"? This premise is not convincing. Numerous other microbial factors, including cell wall components and potential metabolites, can equally modulate the immune system. Furthermore, the authors do not identify which bacterial taxa these OMVs originate from. This omission weakens the logic and relevance of the subsequent downstream analyses linking OMVs to immune cells.

Response: We thank the reviewer for this thoughtful comment and agree that bacterial outer membrane vesicles (OMVs) represent only one of several potential mechanisms through which the airway microbiome may influence host immunity. Our intention was not to imply that OMVs are the principal or exclusive microbial mediators of immune modulation. We have therefore revised the Introduction, Results, and Discussion to clarify this point throughout the manuscript.

Specifically, we now describe OMVs as one functional microbial feature examined alongside microbial community composition, cytokine profiles, immune-cell populations, and host transcriptomic responses, rather than as a presumed mechanistic driver. The Introduction now explicitly acknowledges that numerous microbial products, including cell-wall components, metabolites, and other extracellular molecules, may contribute to host immune regulation, and that OMVs were investigated because experimental studies have demonstrated their immunomodulatory capacity, while their relevance in human HIV-associated COPD remains largely unexplored. Importantly, our revised Results do not support a dominant role for bulk OMV burden. We found that OMV concentrations were not significantly associated with microbial alpha diversity, beta diversity, HIV status, COPD status, or individual bacterial genera after multiple-testing correction, and only modest transcriptional changes enriched for metal-ion pathways were observed across OMV strata. Likewise, among estimated airway cell populations, only proliferating alveolar macrophages showed an independent association with OMV burden after multivariable adjustment.

We also agree that our study cannot determine the bacterial origin of the recovered OMVs. Because OMVs were quantified from unfractionated sputum, the assay measures total extracellular vesicle burden and does not permit taxonomic attribution. We now explicitly acknowledge this limitation in the Discussion and emphasize that bulk OMV abundance should not be interpreted as a surrogate for vesicle production by specific bacterial taxa. Instead, we state that strain-specific OMV cargo, vesicles derived from particular organisms, or other microbial products including metabolites and cell-wall components may represent more informative functional mediators and should be investigated using targeted metagenomic, metabolomic, proteomic, or vesicle-characterization approaches.

Accordingly, we have revised the narrative to avoid implying that OMVs explain the downstream immune and transcriptomic findings. Instead, the manuscript concludes that the strongest and most reproducible biological signals arose from selective taxon-specific host-microbe association networks, whereas bulk OMV burden showed comparatively limited explanatory value. This interpretation more accurately reflects the data and avoids overstating the biological role of OMVs in this cohort.

Major Comment 7. Pages 355-356: I strongly question the conclusion that "Pairwise PERMANOVA and univariate ANCOVA similarly detected no disease-dependent cell-type differences after FDR correction." Both HIV and COPD are diseases known to profoundly impact immune cell populations and their activation states. The assertion that there are no cell-type differences between these patients and healthy controls contradicts established pathophysiological facts.

Response: We thank the reviewer for this comment. In our study, adjusted cell-type models demonstrated no significant main effects of either DualStatus or DualDisease on the relative abundance of estimated airway cell populations following FDR correction (Supplementary Figures S19 and S21). Multivariate analyses were consistent with these observations. MANOVA detected no significant overall effect of disease status on joint airway cellular composition (P = 0.158), while PERMANOVA and cell-type-specific ANCOVA similarly did not identify broad disease-associated shifts in estimated airway immune and epithelial cell proportions after adjustment for age, sex, BMI, smoking status, and correction for multiple testing. These findings should not be interpreted as evidence that HIV or COPD lack immunological effects. Indeed, complementary CyTOF analyses identified selective HIV- and COPD-associated differences in several circulating immune-cell subsets, while confounder-aware integration linked discrete airway microbial taxa with defined immune-cell populations, inflammatory cytokines, and lung-function measures. Rather, our deconvolution analyses indicate that, within this predominantly ART-treated and virologically suppressed cohort, HIV-associated COPD was not accompanied by large-scale remodeling of overall airway cellular composition. This interpretation is consistent with the clinical characteristics of the cohort, in which 98% of participants living with HIV were receiving antiretroviral therapy and 86% were virologically suppressed, conditions expected to attenuate broad alterations in immune-cell composition while allowing persistent functional immune remodeling.
Together, these findings suggest that the dominant biological signal in HIV-associated COPD resides in selective host-microbe-immune interaction networks rather than wholesale redistribution of airway immune-cell populations.

Major comment 8. Pages 428-430: The conclusion stating "Together, these findings show that airway mucosal gene expression is shaped far more by microbial community structure than by HIV or COPD status alone..." is unsupported. The manuscript only provides simple associative analyses, which cannot establish causality. It is impossible to determine from these data whether the microbial community structure is the cause or the consequence of the altered mucosal gene expression.

Response: We thank the reviewer for this important critique and agree that the original wording overstated the strength of inference from our data. Our analyses identify associations between microbial features and airway gene-expression modules, but they do not establish causality, directionality, or whether microbial community differences are a cause or consequence of altered mucosal transcriptional states.

We have therefore revised this conclusion to remove causal and comparative language. We now describe these findings as associations between specific microbial features and mucosal transcriptional programs after adjustment for measured confounders as follows:

These analyses therefore identified reproducible transcriptional modules that consistently aligned with specific bacterial taxa rather than widespread microbiome-transcriptome relationships. However, these associations alone do not indicate whether they arise from extensive restructuring of the airway microbiome or from selective variation within otherwise stable microbial communities. It is worth mentioning that, because these analyses are observational, they cannot determine whether microbial community structure contributes to, results from, or co-varies with altered mucosal transcriptional states. This warrants further investigation. We therefore next examined the broader ecological context of these transcriptional programmes by integrating global microbial diversity, differential gene expression, and pathway enrichment analyses across the clinical groups.

Major Comment 9. Figure Consolidation: The inclusion of 12 main figures is excessive. The authors should consider merging related panels to create fewer, more comprehensive multi-panel figures.

Response: We thank the reviewers for this suggestion. We have merged figures into 5 comprehensive figures. All other figures can be found in supplementary file.

Minor comment 1. Abstract: The abstract should explicitly state the sample size (number of human subjects) included in the analysis.

Response: This has been done, see revised abstract,
Chronic obstructive pulmonary disease (COPD) is increasingly recognized among people living with HIV in sub-Saharan Africa, yet the mechanisms linking airway microbial ecology to chronic inflammation remain poorly

understood. We studied 200 adults stratified by HIV and COPD status, including 160 participants with two-year longitudinal follow-up, using an integrated multi-omics framework combining sputum 16S rRNA profiling, bacterial outer membrane vesicle quantification, airway cytokine profiling, CyTOF immune phenotyping, bulk transcriptomics of induced sputum, bronchoalveolar lavage and peripheral blood, and bronchoalveolar lavage single-cell RNA sequencing-guided deconvolution. Sputum microbial communities were shaped predominantly by temporal ecological turnover rather than HIV or COPD status. Although microbial diversity, microbiome instability, outer membrane vesicle burden and inferred airway cellular composition remained largely preserved, HIV-associated COPD exhibited disease-specific microbial signatures together with extensive inflammatory transcriptional remodeling concentrated in induced sputum. Integrated analyses identified selective host-microbe interaction networks linking discrete microbial taxa with inflammatory, immune and transcriptional programmes.

Minor comment 2. Figure 1: This figure appears to be an AI-generated illustration. It is overly complex and does not seem highly relevant to the specific scientific content of the paper.

Response: This study schematic has been revised, see Figure 1.

Minor comment 3. Figure 3: For Figure 3B, a statistical analysis (e.g., ADONIS/PERMANOVA) should be performed and reported. For Figure 3C, is the observed difference statistically significant? Please provide the appropriate p-values.

Response: We thank the reviewer for this observation. PERMANOVA analysis was performed, adjusting for participant ID, age, sex, and dual disease status. Results show a significant effect of visit on community composition ($F = 2.001$, $R^2 = 0.03251$, $p = 0.001$), indicating temporal changes in the microbiome structure. (Figure 3B). This has been added to the manuscript.

Within-individual microbial communities remained consistently more similar to themselves over time than to those of other participants, indicating preservation of individual-specific microbial signatures (Figure 3C). Statistical comparisons using the *Wilcoxon rank-sum test*, with Benjamini-Hochberg FDR correction, revealed a significant difference in sample similarity for time-point comparisons (adjusted p-value < 0.001), and for within-individual comparisons over time (adjusted p-value = 0.1). This has been added to the manuscript.

Reviewer #2 (Remarks to the Author):
This ambitious manuscript integrates sputum microbiome, transcriptomics, and immune cell profiling to understand HIV-associated COPD. The authors should be commended for building one of the most deeply phenotyped HIV lung cohorts utilizing an array of sequencing platforms. Their longitudinal approach is unique and extremely valuable to better understand disease pathogenesis in this population. Also, this cohort brings forth new data from HIV populations living in Africa which have often been overlooked in HIV lung studies. The authors found that 1) time influences the sputum microbiome more so than disease status; 2) certain genera like Haemophilus and Sphingomonas drive microbiome instability in disease-specific ways; 3) bacterial-derived OMVs have little relationship between disease states and microbiome structures; 4) there were few microbiome-cytokine interactions; 5) certain microbial features are associated in the blood with both T-cell and non-T-cell populations; 6) HIV+/COPD+ is associated with numerous transcriptionally dysregulated genes in sputum, with only a few of these genes associated with microbial taxa; 7) OMVs have a subtle influence on sputum transcriptomics; 8) HIV has a stronger effect on sputum gene expression compared to COPD, with this effect observed less in BAL; 9) Sputum transcriptomics are influenced more by specific microbes rather than by global microbial disruptions or disease status. I think there are a few limitations to the work that need to be addressed.

First, my impression from this paper is that ultimately the microbiome does not really explain the pathogenesis of dual disease status. If anything, the microbiome's influence on sputum transcriptomics and sputum cell composition are subtle. While the transcriptome may be substantially dysregulated in HIV+COPD+, I am less convinced that dysbiotic features are the driver of this based on the data. OMVs for example appear to exert little influence on disease outcomes here. Therefore, as an explanation for HIV+COPD+, I think the authors do not necessarily substantiate their hypothesis. If the influence of disease status on the microbiome or the host response is minimal, then the next question is what is the significance of these findings?

Response: We thank the reviewer for this important and constructive critique. We agree that the data do not support a model in which broad microbiome dysbiosis or OMV burden is the primary driver of HIV+/COPD+ pathogenesis. Our original framing placed too much emphasis on microbial mechanisms, and we have revised the manuscript accordingly.

The revised interpretation is that HIV+/COPD+ disease in this cohort is characterized predominantly by host mucosal immune and transcriptional dysregulation, whereas microbiome-associated effects are modest, selective, and context-dependent. Specifically, global microbiome diversity and OMV burden showed limited association with

disease status, lung-function measures, or broad cellular composition. In contrast, host transcriptional programs showed more substantial disease-associated remodeling.

We have therefore revised the Abstract, Results, and Discussion to make clear that the microbiome does not explain the full pathogenesis of dual HIV/COPD disease. Instead, the significance of our findings lies in identifying a negative result with important biological implications: in this ART-treated rural Ugandan cohort, HIV+/COPD+ is not accompanied by broad airway microbial disruption or a dominant OMV-linked inflammatory phenotype. Rather, disease-associated inflammation appears to be driven largely by host mucosal immune programs, with microbial features acting, at most, as selective correlates of specific inflammatory modules.

We believe this reframing strengthens the manuscript. It moves the central conclusion away from a dysbiosis-driven model and toward a more nuanced model in which treated HIV-associated COPD reflects immune-dominant mucosal remodeling, while the airway microbiome provides limited but potentially informative context-specific signals. This distinction is clinically relevant because it suggests that broad microbiome-targeted interventions may be less appropriate than strategies focused on defining and modifying host inflammatory endotypes. It also identifies specific microbial taxa and OMV-associated patterns as candidate markers for future validation rather than as established causal drivers.

We have also expanded the Limitations section to explicitly state that microbial associations were modest, observational, and insufficient to establish causality, and that independent cohorts, metagenomics/metabolomics, and perturbation experiments will be needed to determine whether any of the observed microbial features contribute mechanistically to airway immune remodeling.

Second, I think a major gap is the use of a deconvoluted bulk to single cell RNA sequencing pathway that lacks necessary detail. I question the validity of their approach and further substantiation that these methods are robust is required before many of the downstream analyses generated from this deconvolution step are to be believed.

Response: We agree that the original submission did not provide sufficient methodological detail for the deconvolution pipeline and that the small size of the BAL single-cell reference is a key limitation. We have therefore expanded the Online Methods section ("BAL-derived snRNA-seq-guided deconvolution of sputum bulk RNA-seq") to describe the workflow in more detail. Specifically, we now state: (i) that sputum bulk RNA-seq counts were processed with edgeR using TMM normalization and filterByExpr, with non-log CPM values supplied to MuSiC; (ii) that the BAL snRNA-seq reference comprised four donors and 5,205 nuclei after QC, annotated into 12 major cell types; (iii) that we constructed donor-specific pseudobulk profiles by summing counts per cell type × donor and used these as the reference, which reduces single-cell noise while preserving donor variability; and (iv) that we applied MuSiC with donor-aware weighting, using only genes shared between bulk and snRNA-seq and constraining fractions to sum to 1 per bulk sample

We fully agree that four BAL donors and 5,205 cells are insufficient for standalone single-cell discovery. In this study, however, the snRNA-seq is used solely as a reference for deconvolution of the larger sputum bulk RNA-seq cohort, not as an independent scRNA-seq discovery dataset. MuSiC is designed for deconvolution with small numbers of reference donors and many cells, and the donor-specific pseudobulk profiles mitigate per-cell noise while retaining the between-donor variation that the algorithm exploits. We now state explicitly in the Discussion that this small reference limits resolution of rare cell types and the precision of estimated fractions, and that all deconvolution-based findings should be regarded as hypothesis-generating.

To assess robustness to reference sampling, we implemented a pseudo-donor perturbation analysis, which is now described in the Online Methods and summarized in a new Supplementary Table. Briefly, we resampled snRNA-seq cells into 3, 5, 7, and 10 pseudo-donors per cell type, reran MuSiC for each setting, and computed cell-type-wise correlations of estimated fractions between settings. For major populations such as alveolar macrophages, T cells, and AT1 epithelial cells, correlations between pseudo-donor settings were high, indicating that the MuSiC estimates are numerically stable despite the small reference. We explicitly acknowledge that an independent matched scRNA–bulk dataset would be the ideal validation; such data are not available for this rural HIV-COPD BAL cohort, and additional bronchoscopy was not feasible within this study. We therefore treat all deconvolution-based results as exploratory and state this explicitly in the revised Discussion.

Importantly, after multiple-testing correction we did not detect statistically robust COPD/HIV group differences in deconvolved sputum cell-type fractions: MANOVA on the joint composition was non-significant (Pillai's trace ≈ 0.52, p = 0.158), and univariate ANCOVAs and PERMANOVAs likewise yielded no FDR-significant disease effects. OMV burden showed no consistent monotonic association with composition beyond a single association with proliferating alveolar macrophages that survived FDR correction. In the revised Results and Discussion, we now emphasize that these null results most likely reflect limited power and deconvolution noise rather than proving absence of disease-linked compositional shifts. We have therefore softened any mechanistic language based on

the deconvolution, and we frame the genus-cell-type associations as exploratory, non-causal signals requiring replication.

We independently repeated the analysis using the publicly available Human Lung Cell Atlas reference as an external validation (Supplementary Figure S17C). Across both reference datasets, airway cellular composition remained remarkably stable. Deconvolution based on the BAL reference identified no significant differences in the estimated proportions of immune and epithelial cell populations across HIV/COPD groups or OMV quartiles (Supplementary Figure S17B; Supplementary Tables ST1-ST2). Similarly, deconvolution using the independent Human Lung Cell Atlas demonstrated highly comparable immune-cell compositions across all four HIV/COPD groups without detectable group-specific compositional differences (Supplementary Figure S17C).

Methods 1. The definition of COPD utilized in this study should be provided.

Response:
We defined COPD as a post-bronchodilator (post-BD) FEV1/FVC ratio lower than the fifth percentile of the NHANES III African-American reference population and used post-bronchodilator FEV1 values to define the severity of disease. (Reference: PMID: 32462945 PMCID: PMC8126339 DOI: 10.1080/15412555.2020.1769583).

Methods 2. Line 637: sentence appears to be cut off.
Response: We have revised this sentence.
Method 3. The scRNASeq analysis only comes from 4 participants. Even for a scRNASeq analysis, this number is quite low. With only 5205 cells reported, I would find this analysis very difficult to interpret. Can the authors justify this sample size? If only 4 participants are then used to inform the bulk seq deconvolution, this could lead to significant downstream bias. How were these deconvolution methods ultimately validated? One would want to see the methods applied to a separate matched scRNAseq-bulk seq dataset. Greater detail needs to be provided as to how these deconvolution methods were obtained.

Response: See response for single cell RNA seq-based deconvolution above.

Results 1. Table 1. Further clarification on the other category in Biomass exposure would be helpful. Inhaled prednisone use is confusing – do you mean oral prednisone or inhaled corticosteroids? Please provide FVC % predicted.

Response: We thank the reviewer for these helpful suggestions.
First, we have clarified the biomass exposure category. The category "Other" refers to non-wood biomass fuels, including wood, charcoal, cow dung as fuel sources.

Second, we apologize for the ambiguity regarding prednisone use. This variable refers to oral prednisone use rather than inhaled corticosteroid therapy. We have revised Table 1 to explicitly indicate "Oral Prednisone Use." See Table 1a/b

Third, we agree that inclusion of FVC % predicted improves characterization of baseline lung function. Spirometry was performed using the Easy One-PC spirometer (ndd Medical Technologies, Zurich, Switzerland), with predicted values generated using the NHANES III reference equations embedded within the device software. We have therefore added post-bronchodilator FVC % predicted to Table 1 and updated the Methods accordingly.

Methods Revision
Spirometry was performed by trained research assistants using Easy On-PC spirometer (ndd Medical Technologies, Zurich, Switzerland) before and 15 min after administration of 400 mcg of salbutamol using a spacer, with predicted values generated using the NHANES III reference equations embedded within the device software. The quality of spirometry test results was assessed by ATS/ERS guidelines.

Result 2. Much is made in the first part of the results of this concept of microbiome instability. Is this a microbiome feature, though, that is clinically important? i.e. does someone with microbiome instability over time have worse lung function outcomes, or disease outcomes?

Response: Response: We thank the reviewer for this important question. We agree that establishing clinical relevance is essential for interpreting the microbiome instability concept, and we tested it directly. We regressed the change in three post-bronchodilator lung function measures (ΔFEV_1, ΔFVC, and $\Delta FEV_1/FVC$ ratio) on stability tertile (Stable, Medium, Unstable), adjusting for age, sex, BMI, and smoking status (n=113 participants with paired spirometry and microbiome data). None of the three outcomes was significantly associated with stability tertile (ΔFEV_1: F=1.33, p=0.27; ΔFVC: F=0.59, p=0.56; $\Delta FEV_1/FVC$: F=1.30, p=0.28; all BH-adjusted p>0.4; Supplementary Table SR7). The direction of effect was also not consistent with an 'instability predicts worse outcomes' hypothesis:

mean ΔFEV$_1$ was slightly higher in the Stable group (+0.055 L) than in Medium (-0.052 L) or Unstable (+0.008 L), though these differences were not significant.

Two considerations frame the interpretation of this null result. First, stability was defined as a within-cohort ranking (tertile split of Bray-Curtis distance to baseline), not an absolute measure of microbiome instability. Consequently, participants in the 'Unstable' tertile shifted more than others in this specific cohort, but this does not translate directly to a clinical threshold or reflect a pre-defined biological state of dysbiosis. Second, this null adds to a consistent pattern already reported in the manuscript: neither continuous Bray-Curtis shift magnitude nor ΔShannon predicted changes in lung function across the cohort (Supplementary Figures S9), and disease status did not predict the magnitude of microbiome shift (Figure 3, Supplementary Figures S9).

Taken together, our data indicate that in this cohort microbiome instability itself is not a clinically informative biomarker for two-year lung function trajectories. What is biologically meaningful, however, is the taxonomic composition of that instability, the disease-specific enrichment of *Haemophilus* in unstable microbiomes of COPD$^+$/HIV$^+$ participants, and the *Sphingomonas* association with more stable microbiomes across strata (Figures 3). This distinction that the total magnitude of shift is not clinically predictive, but the specific taxa driving that shift differ by disease status is now stated explicitly in the Results and Discussion.

Result 3. Figure 6D could be clarified. It is hard to know which of the plots belong to which low/medium/high category and the colors should be explicitly defined in the legend.
Response: This has been addressed. The figure has been moved to supplementary figure S11

Result 4. Lines 247-248 and Figure 7. This needs to be clarified. I'm confused about the statement that HIV-negative status was associated with reduced IL-12p40 and IL2. In the figure. It looks as though HIV status (which I interpreted as HIV+) is more negative (green) compared to HIV-negative. The text and the figure don't seem congruent and it may mean that you need to specifically state what you mean by HIV status in the figure.

Response: We thank the reviewer for identifying this ambiguity. We agree that the original figure labeling could be misinterpreted. In this Figure, (now figure 4H in the revised muniscript), the "HIV status" coefficient represents the contrast of HIV-positive participants relative to the HIV-negative reference group. Thus, negative coefficients indicate lower cytokine levels among HIV-positive participants compared with HIV-negative participants. The original text incorrectly referred to HIV-negative status as being associated with reduced IL-12p40 and IL-2. We have corrected the Results text to state that HIV-positive status was associated with lower IL-12p40 and IL-2 relative to HIV-negative status, and we have revised the figure label and legend to make the contrast explicit.

Results 5. Lines 304-306. I think the statement here that the links between airway dysbiosis and inflammatory transcription is a "strong association" is a reach. One sees many transcription disruptions in dual disease status, but many of these are not explained by the microbial taxa.

Response:
We thank the reviewer for this thoughtful observation and agree that the original wording overstated the extent to which microbial variation accounted for the observed transcriptional changes. While we identified statistically significant associations between specific microbial taxa and selected inflammatory gene-expression programs, we did not intend to imply that microbial features explained the majority of transcriptional perturbations observed in participants with dual HIV/COPD disease. We agree that the transcriptional alterations associated with dual disease status were substantially broader than those linked to individual microbial taxa. Indeed, many differentially expressed genes were not associated with the microbial features evaluated in this study, indicating that additional host, environmental, clinical, and immunological factors likely contribute to the observed transcriptional landscape. This section has been revised.

Result 6. Minor point: on Figure 11 many people would not know what Ekyoto is so it should be defined in the legend.

Response: This has been revised to wood stove.

Result 7. Demographic tables should be provided of the subsets who underwent bronchoscopy, CyTOF analysis, Bioplex assay, and bulk RNASeq. Were these subsets representative of the larger cohort?

This has been addressed. See table 2. There were no statistically significant differences in socio-demographic and clinical characteristics across subsets.

Result 8. As a stylistic point, as you have both sputum and BAL samples reported here, it can be confusing to the reader which compartment is referred to when the only term used is "airway" which could refer to either compartment. Would clarify in the figure legends and in the text which samples are being addressed.

Response: This has been addressed throughout the manuscript.

Discussion 1
Lines 438-441. I'm not sure you've shown that restoration of mucosal immunity has attenuated the magnitude of lung function decline (i.e. you don't have a comparison group where immune reconstitution has not occurred or you don't have pre-ART lung function trajectory).

Response: We thank the reviewer for this important point and agree that our original wording implied a causal interpretation that is not supported by the available data. Our study did not include pre-ART lung-function measurements, untreated HIV-positive participants, or longitudinal assessment of immune reconstitution. Consequently, we cannot determine whether restoration of mucosal immunity attenuated lung-function decline.

We have therefore revised the Discussion to remove this causal inference. The revised text now emphasizes that the observed immunological and microbiome profiles were identified in a cohort of ART-treated individuals and may reflect the consequences of long-term HIV treatment, immune reconstitution, persistent immune dysregulation, or other factors. We now explicitly acknowledge that longitudinal studies will be required to determine whether immune restoration influences subsequent lung-function trajectories.

We hope you find our responses satisfactory and the quality of our revised manuscript up to the standards of Nature Communications Journal.

Thank you for your consideration.

Sincerely,

Sofia Kirke Forslund-Startceva,
Group Leader, Host-microbiome factors in cardiovascular disease,
Professor, Charité - Universitätsmedizin Berlin & Max Delbrück Center for Molecular Medicine, Experimental and Clinical Research Center (ECRC), Berlin-Buch,
Robert-Rössle-Street 10, 13125 Berlin, Germany,
Contact: +49 30 450540649,
Email address: Sofia.Forslund@mdc-berlin.de ,
https://www.mdc-berlin.de/forslund,
(on behalf of all authors)

Körperschaft des öffentlichen Rechts

Vorstand:
Prof. Dr. Martin Lohse
Prof. Dr. Heike Graßmann

Berliner Sparkasse –
Niederlassung der Landesbank Berlin AG

BLZ: 100 500 00 / Kto. 195 323 1140
IBAN: DE38 1005 0000 1953 2311 40
BIC: BELADEBE
VAT: DE811261930

Zertifikat seit 2009
audit berufundfamilie

REVIEWER COMMENTS

Reviewer 1 (Remarks to the Author):

This revised manuscript has addressed most of the issues I previously raised and included additional analyses, thereby strengthening the reliability of the conclusion that "HIV-associated COPD is characterized primarily by localized immune-transcriptional remodeling accompanied by selective host-microbe interactions, rather than widespread restructuring of the airway microbiome". However, before the conclusions can be accepted, the authors must address the critical issues of the reproducibility of their analytical results and the "Data and Code Availability", which, in its current state, is not qualified. As noted in my previous comments, the sample size of this study is relatively small, especially for certain assays such as the BALF scRNA-seq, which only includes 4 cases. Furthermore, the main conclusions of the article rely on complex, multi-layered statistical analyses without independent cohort validation or experimental verification. Therefore, an essential way to evaluate the validity of the conclusions is through the public availability of the data and analytical code, including the availability of the input files for the code. However, upon reviewing the GitHub repository provided by the

authors, I found only a few lines of code with no annotations. The repository fails to provide abundance tables for the transcriptome, cytokines, and microbiome, nor does it provide patient metadata (patient privacy information can be removed, but unique identifiers are needed to link the omics data with patient grouping information). There is a complete absence of the formulas used for core analyses, such as differential gene expression analysis, deconvolution analysis, and various statistical analyses, as well as the crucial input files for these analyses. Without the public availability of these codes and data, it is exceedingly difficult for readers to evaluate the reliability of this paper.

Reviewer 1 (Remarks on code availability):
This code is completely inadequate for evaluating the reliability of the paper. Only a few trivial snippets of code have been uploaded, and it lacks a README file, the core analytical code of the paper, and the input source data used for the analyses. It lacks any reproducibility whatsoever.

Response:
Thank you for bringing this to our attention. We would like to clarify that the link provided in the previous submission referred to an older, incomplete version of the repository. We apologize that this did not provide sufficient material to assess the reproducibility of our analyses. We have now deposited the complete analysis package in a public Zenodo archive: https://zenodo.org/records/21473246 . The archive contains a detailed README file, complete analysis scripts, processed analysis inputs, de-identified sample metadata, primary microbiome analysis files, and source data for the transcriptomic, single-cell, CyTOF, and MetaDeconfoundR analyses.

Specifically, the Zenodo archive includes:

- **Complete R scripts for preprocessing, alpha- and beta-diversity analyses, PERMANOVA, genus-level mixed models, batch/drift correction, genus-cell-fraction models, RNA-seq association analyses, gene-microbe linear models, MetaDeconfoundR analyses for the full and HIV-stratified cohorts, and lung-function analyses.**
- **Processed microbiome inputs, including rarefied and filtered OTU-, genus-, and phylum-level abundance tables, rarefied read tables, the OUT-genus mapping table, and sample metadata.**
- **Primary 16S microbiome analysis files, including the OTU BIOM table, representative sequences, taxonomic assignments, phylogenetic tree, contaminant information, LotuS logs, and higher-level taxonomic abundance tables.**
- **Bulk RNA-seq count matrices for BAL and PBMC samples, merged count matrices, quality-control data, normalized expression matrices, and the corresponding sample metadata.**
- **Single-cell BAL data in both .h5ad and Seurat .rds formats, together with the Harmony integration workflow and single-cell metadata.**
- **CyTOF cell-frequency tables and the input files used for deconvolution and MetaDeconfoundR analyses.**
- **De-identified clinical and assay metadata required to link samples across modalities and assign participants to the clinical and longitudinal study groups**

Other Comments:
1. Figure 1: The study design figure should clearly state and illustrate the exact timeline of the longitudinal follow-up.

Response: **We thank the reviewer for this helpful suggestion. We have revised Figure 1 to clearly illustrate the exact timeline of the longitudinal follow-up. The corresponding Figure 1 legend has also been revised to explicitly describe the follow-up timeline, ensuring that the longitudinal study design and timing of study procedures are clear to the reader.**

2. Figure 3B: In the PCoA plot, different shape morphologies (e.g., triangles and squares) should be used to represent HIV+/HIV-, and empty or filled shapes should be used to represent COPD+/COPD-, to simultaneously reflect the grouping characteristics of both HIV and COPD.

Response:
We thank the reviewer for this helpful suggestion. We have revised Figure 3B so that HIV and COPD status are now simultaneously encoded by symbol morphology and fill. Specifically, squares represent HIV− and triangles represent HIV+, while filled symbols represent COPD- and open symbols represent COPD+. Study visit remains distinguished

by color. The Figure 3 legend has also been revised to explicitly define these graphical encodings, allowing the four HIV/COPD groups and longitudinal visit structure to be interpreted simultaneously.

3. Lines 180-181 and Figure 3C: The text states, "Despite this temporal turnover, within-individual microbial communities remained consistently more similar to themselves over time than to those of other participants, indicating preservation of individual-specific microbial signatures". However, Figure 3C shows that the BC distance within the "same individual" is higher than that in "different individuals," which implies the similarity within the same individual is lower than that between different individuals. This contradicts the conclusion drawn in the text.

Response: We thank the reviewer for identifying this inconsistency. The reviewer is correct that our previous interpretation of Figure 3C was not supported by the direction of the Bray-Curtis distances. Because lower Bray-Curtis distance indicates greater community similarity, the distributions shown in Figure 3C do not support our previous statement that microbial communities from the same individual remained consistently more similar to themselves over time than to those of other participants. We have therefore removed this conclusion from the manuscript and revised the corresponding Results text to state that Bray-Curtis dissimilarity across visits was comparable between samples obtained from the same individual and those compared between different individuals, providing little evidence for preservation of individual-specific microbial signatures over the follow-up period.

4. Line 289: This section suggests that the lack of differences in peripheral immunity associated with HIV status might be related to viral suppression in the patients. Since 86% of the patients in this cohort achieved virological suppression, does this mean that 14% were not effectively suppressed? If so, are there any differences in the peripheral immune-cell composition and sputum inflammatory cytokines between the 86% virologically suppressed patients and the 14% unsuppressed patients?

Response: We thank the reviewer for this important point. The 86% figure refers to virological suppression among participants with available viral-load information and should not be interpreted as providing a sufficiently powered suppressed-versus-unsuppressed subgroup for the immune analyses. In particular, viral-load measurements were not available for all participants with CyTOF data. Among participants for whom both CyTOF and viral-load information were available, classification using detectable versus undetectable viral load yielded only 9 participants with undetectable viral load and 6 with detectable viral load. The remaining participants lacked contemporaneous viral-load measurements. We therefore considered this subset too small to support a statistically reliable comparison of high-dimensional peripheral immune-cell populations, particularly given the number of cell populations tested and the consequent multiple-testing burden.

We have consequently not undertaken an additional suppressed-versus-detectable CyTOF analysis, as we believe that an inadequately powered subgroup analysis would risk generating unstable or misleading estimates. We similarly avoid extending the interpretation of virological suppression to subgroup comparisons for which adequate paired viral-load and immune measurements are unavailable.

Importantly, we have revised the manuscript to temper our original interpretation. Rather than suggesting that the absence of marked peripheral immune differences was attributable to virological suppression, we now state that this finding may be consistent with the predominantly ART-treated and virologically suppressed nature of the cohort, but that the available data do not permit a sufficiently powered direct comparison of peripheral immune profiles according to virological suppression status. We have also acknowledged this limitation in the revised manuscript.

5. Lines 342-344: "we performed confounder-aware transcriptomic analyses of induced sputum, bronchoalveolar lavage (BAL), and peripheral blood mononuclear cells (PBMCs) (Figure 5)". Please specify the exact number of samples included for each group within each compartment category.

Response: We thank the reviewer for requesting this clarification. We have revised the Results and Figure 5 legend to provide the exact sample numbers included in the confounder-aware transcriptomic analyses for each compartment and disease grouping. For induced sputum (n=80), 39 participants were HIV- and 41 HIV+, 42 were COPD- and 38 COPD+, and 62 were Dualdisease- and 18 Dualdisease+. For BAL (n=48), 26 participants were HIV- and 22 HIV+, 24 were COPD- and 24 COPD+, and 38 were Dualdisease- and 10 Dualdisease+. For PBMCs (n=21), 10 participants were HIV- and 11 HIV+, 7 were COPD- and 14 COPD+, and 13 were Dualdisease- and 8

Dualdisease+. These sample numbers are now explicitly reported in the revised manuscript and Figure 5 legend to clarify the sample sizes underlying each compartment-specific comparison.

6. Line 365: "Selective microbial features align with coordinated airway transcriptional programmes". Consider visualizing the results of this section and placing them in the supplementary figures.

<u>Response</u>: We thank the reviewer for this helpful suggestion. We have now added a new Extended Data Figure 1 to directly visualize the microbiome-host transcriptional associations described in this section. The new figure displays the complete set of host transcripts with at least one microbial association meeting BH-FDR < 0.10, distributed across 17 sequential heatmap panels for readability. Microbial features are shown as rows and host transcripts as columns, with cell color representing effect size and asterisks denoting associations that remain significant after multiple-testing correction. The same microbial features are retained across panels to facilitate comparison of coordinated transcriptional patterns, and taxa lacking genus-level annotation are displayed at the highest informative taxonomic level. We have also revised the corresponding Results text to explicitly refer readers to Extended Data Figure 1, while retaining the complete numerical association results in Supplementary Table ST1. This addition provides a direct visual summary of the selective microbial features that align with coordinated airway transcriptional programmes and addresses the reviewer's request for visualization of this analysis.

7. Lines 941-989: This section involves numerous subgroup classifications and inter-group difference analyses, such as the stability stratification. In these analyses, the sample size is crucial to the reliability of the results. Please explicitly state the sample size included for each group in each type of analysis, which could be listed as "(n=x)" next to each group.

<u>Response</u>: **Thank you for this comment. We agree that the sample sizes underlying the longitudinal and stability-stratified analyses should be stated explicitly. We have therefore revised the Methods, figure legends, and Supplementary Tables to report both the number of microbiome samples and the number of participants included in each analysis. The longitudinal 16S microbiome dataset comprised 356 samples from 255 participants. At visit 1, 200 samples were available, with 50 samples in each DualStatus group. At visit 2, 156 samples were available: COPD-/HIV- (n=36), COPD-/HIV+ (n=40), COPD+/HIV- (n=41), and COPD+/HIV+ (n=39). Longitudinal Bray-Curtis instability and stability-stratified analyses were restricted to the 101 participants with one valid microbiome sample at both visits. This paired cohort comprised 26 COPD-/HIV-, 28 COPD-/HIV+, 24 COPD+/HIV-, and 23 COPD+/HIV+ participants. Participants were classified as Stable (n=34), Medium (n=34), or Unstable (n=33) according to tertiles of their within-individual OTU-level Bray-Curtis distance between visit 1 and visit 2. We now report the exact Stability × DualStatus sample size for all subgroup analyses in Supplementary Table, ST5. Given the small size of several Stability × DualStatus cells, we have clarified that stability-stratified taxon-level findings are exploratory and should be interpreted cautiously.**

Reviewer 2

"I am still puzzled though by the deconvolution methods. The authors state validation using the Human Lung Cell Atlas but when I review Supplementary figures 17B and 17C, there appears to be quite substantial differences in cell proportions. I still find this aspect of the paper to have questionable validity."

<u>Response</u>

We thank the reviewer for highlighting this important issue. We agree that our previous description of the Human Lung Cell Atlas (HLCA)-based analysis as an independent "validation" was imprecise and could imply that the two approaches were expected to reproduce identical absolute cell fractions. We have therefore substantially revised the deconvolution analysis, its presentation in the manuscript, and our interpretation of these results.

First, we now explicitly distinguish the two deconvolution frameworks. Our primary analysis used a BAL snRNA-seq reference comprising four donors from the same parent cohort that contributed the induced-sputum samples and 5,205 nuclei after quality control, annotated into 12 major cell populations. Donor-specific pseudobulk profiles were constructed for each cell type × donor and used with MuSiC, retaining the donor-aware weighting intrinsic to the algorithm. The alternative analysis used the publicly available HLCA reference, from which 15 immune-cell populations were retained; AutoGeneS was used to select 400 informative marker genes, followed by ν-support vector regression to estimate cell fractions. The two approaches therefore differ in tissue source, cellular taxonomy, reference construction and deconvolution algorithm and should not be regarded as technical replicates from which identical absolute fractions are expected. We have consequently replaced the term "validation" throughout the

manuscript with "external reference-sensitivity analysis." This distinction is now made explicitly in the revised Methods, Results and Supplementary Figure S17.

Second, we have substantially expanded the reference-sensitivity analysis rather than relying on visual comparison of the original stacked proportion plots. We now formally test disease-associated differences in individual inferred cell populations under each reference framework. In the complete HLCA-derived sputum dataset (n=100), classical monocytes and conventional dendritic cells remained significant after multiple-testing correction (both BH-FDR=0.0095), with natural killer cells also significant (BH-FDR=0.0495) across the four HIV/COPD groups (new Supplementary Fig. S17D; SR15). In contrast, in the 48 sputum samples retained in the BAL-reference/MuSiC workflow, none of the 12 inferred cell populations differed significantly among HIV/COPD groups after multiple-testing correction (new Supplementary Fig. S17E; SR16).

Third, we have added a formal global compositional reference-sensitivity analysis (new Supplementary Fig. S17F; SR17). Because estimated cell fractions are compositional, proportions were zero-adjusted, re-closed and centered log-ratio transformed before multivariate analysis. HLCA-derived cellular composition differed significantly among HIV/COPD groups (PERMANOVA: pseudo-F=2.286, R^2=0.0667, P=0.0063; 9,999 permutations), without significant heterogeneity of multivariate dispersion (P=0.0979). By contrast, BAL-reference/MuSiC-derived composition showed no significant global separation (pseudo-F=0.988, R^2=0.0631, P=0.4641), again without evidence of heterogeneous dispersion (P=0.9171). We additionally visualize both analyses in CLR-PCA space. Importantly, although statistical significance differed, the proportion of compositional variance attributable to HIV/COPD status was similar in magnitude between the two frameworks (R^2=0.067 for HLCA versus 0.063 for BAL/MuSiC). We therefore now interpret these data as evidence for modest, reference-sensitive disease-associated compositional structure, rather than either complete concordance or broad reference-independent cellular restructuring.

Fourth, we have clarified why the BAL/MuSiC framework remains the primary dataset for the downstream OMV- and microbiome-cell analyses. This choice was based on biological and study-design considerations rather than on which framework produced statistically significant disease-group differences. The BAL snRNA-seq reference was generated from participants within the same parent cohort as the induced-sputum samples, providing a cohort-derived airway reference that is more closely aligned with the population under study than an external atlas. MuSiC also explicitly incorporates between-donor variation during estimation. The HLCA analysis is therefore used to assess sensitivity to an independent external reference rather than to replace the cohort-derived framework. We now state this rationale explicitly in the revised Results.

Finally, we have expanded the Limitations of the Study to address the reviewer's concern directly. We now acknowledge that the BAL reference contains only four donors and 5,205 nuclei; that deconvolution-derived fractions are model-based estimates rather than direct measurements of cell abundance; that the BAL/MuSiC and HLCA frameworks differ in tissue source, cellular taxonomy, reference construction and sample coverage; and that fine-grained cell-proportion estimates should therefore be interpreted cautiously. We further acknowledge that an independent dataset containing matched bulk RNA sequencing and direct single-cell or cytometric measurements would provide stronger validation of inferred cellular proportions.

Accordingly, we no longer claim that the HLCA analysis validates the absolute cell fractions obtained using the BAL reference. Instead, the revised manuscript presents the two approaches explicitly as complementary cohort-derived and external-reference frameworks, quantifies their agreement and divergence at both individual-cell and global compositional levels, and retains the observed differences rather than treating them as methodological concordance. Our revised conclusion is therefore more circumscribed: major BAL/MuSiC-inferred cell populations were not broadly altered by HIV/COPD status or OMV strata; the HLCA framework identified modest disease-associated differences in selected populations and global composition; and fine-grained deconvolution estimates are sensitive to reference choice. We believe these additional analyses, revised figures, methodological clarifications and more conservative interpretation directly address the reviewer's concern regarding the validity and reproducibility of the deconvolution analysis.

We hope you find our responses satisfactory and the quality of our revised manuscript up to the standards of Nature Communications Journal.

Thank you for your consideration.

Sincerely,

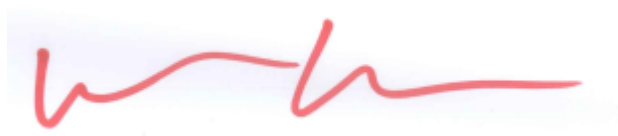

Sofia Kirke Forslund-Startceva,
Group Leader, Host-microbiome factors in cardiovascular disease,
Professor, Charité - Universitätsmedizin Berlin & Max Delbrück Center for Molecular Medicine, Experimental and Clinical Research Center (ECRC), Berlin-Buch,
Robert-Rössle-Street 10, 13125 Berlin, Germany,
Contact: +49 30 450540649,
Email address: Sofia.Forslund@mdc-berlin.de ,
https://www.mdc-berlin.de/forslund,
(on behalf of all authors)

Körperschaft des öffentlichen Rechts

Vorstand:
Prof. Dr. Martin Lohse
Prof. Dr. Heike Graßmann

Berliner Sparkasse –
Niederlassung der Landesbank Berlin AG

BLZ:	100 500 00 / Kto. 195 323 1140
IBAN:	DE38 1005 0000 1953 2311 40
BIC:	BELADEBE
VAT:	DE811261930

Zertifikat seit 2009
audit berufundfamilie
